# Synthesis of a glycan hairpin

**DOI:** 10.1038/s41557-023-01255-5

**Published:** 2023-07-03

**Authors:** Giulio Fittolani, Theodore Tyrikos-Ergas, Ana Poveda, Yang Yu, Nishu Yadav, Peter H. Seeberger, Jesús Jiménez-Barbero, Martina Delbianco

**Affiliations:** 1 Department of Biomolecular Systems, Max Planck Institute of Colloids and Interfaces, Potsdam, Germany; 2 Department of Chemistry and Biochemistry, Freie Universität Berlin, Berlin, Germany; 3 CICbioGUNE, Basque Research and Technology Alliance, Derio, Spain; 4 Ikerbasque, Basque Foundation for Science, Bilbao, Spain; 5 Department of Organic Chemistry II, Faculty of Science and Technology, University of the Basque Country, Leioa, Spain; 6 Centro de Investigación Biomedica en Red de Enfermedades Respiratorias, Madrid, Spain; 7 Present Address: Department of Chemistry, Massachusetts Institute of Technology, Cambridge, MA USA; 8 Present Address: Department of Chemistry, University of Illinois, Urbana, IL USA; 9 Present Address: Simpson Querrey Institute, Northwestern University, Evanston, IL USA

**Keywords:** Supramolecular chemistry, Carbohydrate chemistry, Solution-state NMR

## Abstract

The primary sequence of a biopolymer encodes the essential information for folding, permitting to carry out sophisticated functions. Inspired by natural biopolymers, peptide and nucleic acid sequences have been designed to adopt particular three-dimensional (3D) shapes and programmed to exert specific functions. In contrast, synthetic glycans capable of autonomously folding into defined 3D conformations have so far not been explored owing to their structural complexity and lack of design rules. Here we generate a glycan that adopts a stable secondary structure not present in nature, a glycan hairpin, by combining natural glycan motifs, stabilized by a non-conventional hydrogen bond and hydrophobic interactions. Automated glycan assembly enabled rapid access to synthetic analogues, including site-specific ^13^C-labelled ones, for nuclear magnetic resonance conformational analysis. Long-range inter-residue nuclear Overhauser effects unequivocally confirmed the folded conformation of the synthetic glycan hairpin. The capacity to control the 3D shape across the pool of available monosaccharides has the potential to afford more foldamer scaffolds with programmable properties and functions.

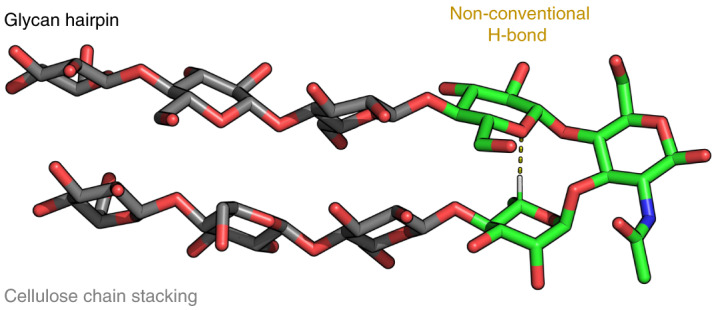

## Abstract (web summary)

Inspired by the design of peptide and nucleic acid sequences to adopt particular three-dimensional shapes, natural glycan motifs have now been combined to construct a glycan that adopts a hairpin conformation in water. Thus a designed glycan can now autonomously fold into a stable secondary structure absent in nature.

## Main

Linear amino acid sequences encode the information needed to generate three-dimensional (3D) motifs such as helices, sheets and turns, which can combine to give complex functional macromolecules (that is, proteins). Using the same alphabet of 20 α-amino acids, it is possible to design molecules with engineered 3D structures^1^. The development of unnatural amino acids expanded the catalogue even further, and molecules with size, shape and arrangement beyond the natural ones could be generated (that is, foldamers)^2–5^. Following the principle ‘form follows function’, foldamers were designed to recognize specific ligands^6^ or penetrate cell membranes^4^ as well as to perform catalysis^7,8^ or undergo supramolecular assembly^9^. Moreover, these designed systems largely improved our understanding of the factors influencing the stability of protein folding^1,10^.

In contrast, glycan structures capable of folding into defined 3D shapes have never been designed, despite the potential numerous advantages. Glycans are constructed from a vast alphabet of more than 100 natural monosaccharides that generates linear or branched polymers, suggesting an enormous potential to access new conformations. The presence of many hydroxyl groups with well-defined orientations provides opportunities for site-specific functionalization. The tendency of naturally occurring polysaccharides to self-assemble into hierarchically organized materials is another attractive feature^11^. However, so far, the complex chemical synthesis of glycans and the lack of design principles have prevented access to glycans with predictable 3D shapes. Glycans are historically considered flexible molecules rarely exhibiting stable secondary structures^12^, with few exceptions for polysaccharides capable of adopting helix- (for example, amylose) or ribbon-like (for example, cellulose) conformations in solution^13^. Nevertheless, the central role of glycans in a biological setting is often tightly related to the conformation and presentation they adopt^14^. Recent studies report increasing evidence that even small glycans can adopt relatively well-defined conformation in solution, often stabilized by intramolecular hydrogen bonds^15–25^. The increased understanding of glycan behaviour suggests that glycans capable of adopting stable 3D architectures can be created.

In this Article, we rationally designed a glycan capable of folding autonomously into a secondary structure motif to challenge the common view that sees glycans exclusively as flexible molecules^12,26^. Inspired by peptide model systems, we constructed a glycan that adopts a hairpin secondary structure motif in aqueous solution. The design was based on the combination of natural glycan structural elements and aided by molecular dynamics (MD) simulations. Automated glycan assembly (AGA) provided rapid access to a series of well-defined glycan sequences, including ^13^C-labelled analogues to facilitate the structural and conformational analysis. Nuclear magnetic resonance (NMR) spectroscopy detected long-range inter-residue nuclear Overhauser effects (NOEs) that unequivocally confirmed the folded conformation of the synthetic glycan hairpin. This work demonstrates that it is possible to program glycans adopting defined conformation in aqueous solution^27^, opening opportunities for endowing glycans with new properties and functions. Analogous to the discovery of peptide-based foldamers, we envision applications of folded glycans in several areas, including materials science^28,29^, biology^30^ and catalysis^31^.

## Results and discussion

### Rational design of a glycan hairpin

**Fig. 1:**
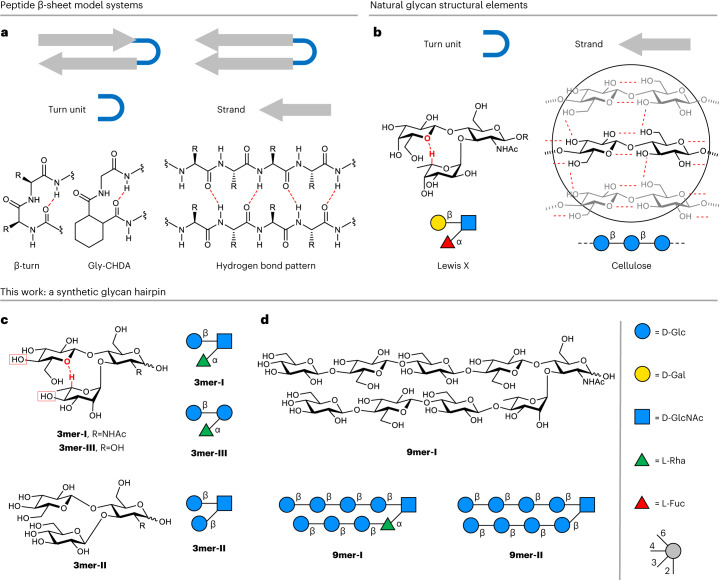
Design of a glycan hairpin. **a**, Autonomously folding antiparallel or parallel peptide β-sheet model systems are composed of a turn unit (for example, β-turn or Gly-CHDA)^32^ and two strands interacting via hydrogen bonds (red dotted lines, exemplified for parallel β-sheets). **b**, Naturally occurring glycans with turn-like conformation (that is, LeX trisaccharide) and strand-like conformation (that is, cellulose) inspired the design of an artificial glycan hairpin. **c**, The glycan turn unit **3mer-I**, and **3mer-III**, was designed to preserve the non-conventional hydrogen bond (red dotted line) and optimally align two hydroxyl groups (red boxes) to elongate the strands of the hairpin. **d**, Synthetic glycan hairpin model system composed of two cellulose strands attached as branches to a trisaccharide turn unit. The following abbreviations are used for monosaccharides: Glc, glucose; Gal, galactose; GlcNAc, *N*-acetyl glucosamine; Rha, rhamnose; Fuc, fucose. The monosaccharide residues are represented following the Symbol Nomenclature for Glycans (SNFG) graphical representation^74^.

Peptide model systems have largely improved our understanding of the factors influencing protein folding and stability^1,10^. This is especially true in the case of protein β-sheets, for which folding rules could be deduced from model systems consisting of a hairpin (that is, strand–loop–strand). In a hairpin, the β-turn acts as a β-sheet nucleator, placing the two strands adjacent to each other and favouring inter-strand interactions in a parallel^32^ or antiparallel^33^ fashion (Fig. 1a). Intramolecular hydrogen bonds and side-chain interactions stabilize the turn unit and hold the two strands in a closed hairpin conformation (Fig. 1a). Model systems such as double-^34,35^ or triple-stranded^36,37^ β-hairpins served as blueprints to gain molecular insights into protein folding^32,33,38–40^ and have proven essential to study structural aspects of amyloid aggregation^41^. Beyond applications in biology, these systems have been employed to generate self-assembling nanomaterials^42–44^ and hydrogels^45,46^, or as catalysts for asymmetric transformations^47,48^.

Given the breadth of the research on peptide hairpins, we decided to explore the design of an analogous secondary structure motif in glycans. The molecular nature of glycans, composed of hydroxyl-rich backbones, makes the design of a glycan folded secondary structure highly challenging in comparison with peptides and imposes the exploration of new design rules. Interactions with water are strong and drastically impact the conformation in solution, disrupting intramolecular hydrogen bonds. Long-range inter-residue interactions are scarce, and the absence of hydrophobic side chains limits the stability of glycan secondary structures^12^. Still, within the glycan realm, subtle non-covalent interactions can take place (Fig. 1b), which have a large impact on macroscopic properties^49^ or bioactivity^14^. These non-covalent interactions, combined with design rules developed for peptides, served as inspiration for the creation of a glycan hairpin.

A parallel glycan hairpin structure, composed of two strands attached to a turn, is a secondary structure motif absent in nature. The design requires (1) a suitable glycan loop adopting a turn conformation and (2) two stacking strands^50^. The similarity of the Lewis X (Le^X^, α-l-Fuc-(1,3)-[β-d-Gal-(1,4)]-d-GlcNAc) trisaccharide conformation to that of naturally occurring peptide β-turns prompted us to explore this glycan motif as a starting point for the design of the turn unit. Le^X^ adopts a fairly rigid closed conformation in aqueous solution stabilized by hydrophobic interactions between the methyl group of L-Fuc and the β-face of Gal. Further stabilization arises due to a non-conventional CH···O hydrogen bond^51^ between the H-5 of L-Fuc (l-Fuc-5) and the O-5 of Gal branches (Fig. 1b)^19,20,24^. This CH···O hydrogen bond forms a ten-membered atom ring analogous to naturally occurring peptide β-turns. Looking at the 3D shape of Le^X^, we noticed that further monosaccharide extensions at the OH-4 positions of the stacked residues could keep a parallel arrangement between the two new moieties, provided that OH-4 displays an equatorial orientation^20^. Therefore, the canonical Le^X^ trisaccharide loop was modified by converting β-1,4-Gal into β-1,4-Glc and α-1,3-l-Fuc into α-1,3-l-Rha, both presenting OH-4 in the equatorial orientation (Fig. 1c, **3mer-I**). We hypothesized that the non-conventional hydrogen bond could still act as a key stabilizing force to keep in spatial proximity the two branches^20^. Two additional turn units were also designed: (1) **3mer-II** is based on GlcNAc substituted with two Glc residues attached as β-1,4 and β-1,3 branches, lacking the stabilizing non-conventional H-bond, and (2) **3mer-III**, in which the branched GlcNAc residue was substituted by Glc, ideally preserving an optimal spatial arrangement of the two branches to engage in the CH···O hydrogen bond (Fig. 1c).

As model for the stacking strands, we were inspired by the cellulose backbone, a polysaccharide consisting of a β-1,4-Glc repeating sequence (Fig. 1b). Cellulose is a linear polysaccharide adopting a rigid rod conformation in solution stabilized by intramolecular hydrogen bonds between the OH-3 and O-5 of neighbouring Glc residues. Strong intermolecular interactions such as hydrogen bonds and hydrophobic interactions between the C-H-rich faces of Glc drive amphiphilic cellulose chains to self-assemble into highly ordered (insoluble) crystallites^49,52^. Thus, we selected oligomers of cellulose as strand sequences due to their propensity to assemble in a regular manner^53,54^.

Overall, our design includes a rigid turn-like glycan, stabilized by a non-conventional CH···O hydrogen bond, carrying two cellulose oligomer strands. Natural glycan structural elements are thus combined to generate an unnatural glycan hairpin. As target structures, we set a short pentasaccharide (**5mer-I**) and two nonasaccharide analogues (**9mer-I** and **9mer-II**) (Fig. 1d).

**Fig. 2:**
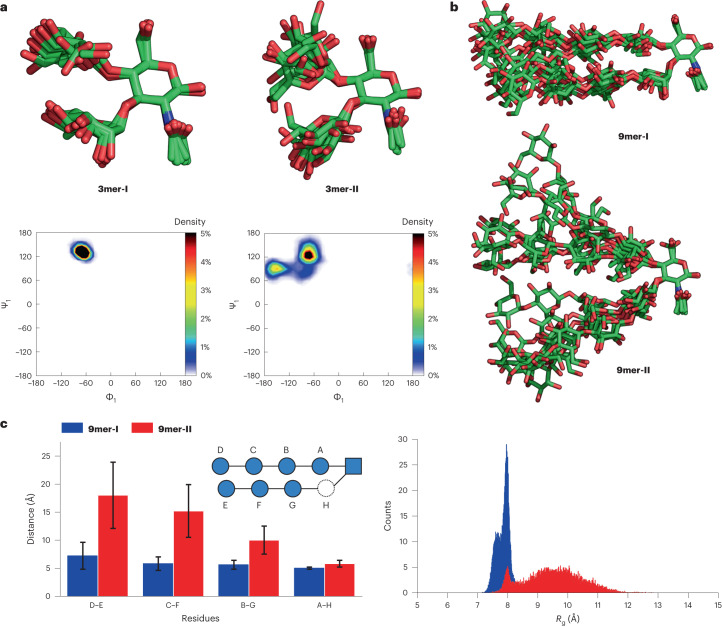
MD simulations of the turn units and glycan hairpins. **a**, Over-imposition of seven representative snapshot (top) and Ramachandran plots for the β-1,4-Glc linkage (bottom) extracted from the MD simulation show the larger degree of flexibility of **3mer-II** compared with **3mer-I**. **b**, Over-imposition of seven representative snapshot shows a closed hairpin conformation for **9mer-I** (top) in contrast to a highly flexible and open conformation for **9mer-II** (bottom). **c**, Average inter-residue distances (left) and *R*_g_ (right) plots showing opposite trends for the two nonasaccharides regarding overall conformation and flexibility. Average inter-residue distances and *R*_g_ were calculated from the MD simulation trajectory (25.001 frames, 500 ns). Error bars represent the standard deviation. Overall, MD suggests that a rigid turn is needed to preserve the closed hairpin conformation.

Atomistic MD simulations were carried out to screen the tendency of the designed glycans to adopt the desired conformation. All the modelled structures were simulated for 500 ns, employing a modified version of the GLYCAM06 (ref. ^55^) carbohydrate force field. The systems were solvated with TIP5P^56^ water model to avoid excessive interactions between the monomers^57^. We began by comparing **3mer-I**, **3mer-II** and **3mer-III** to identify the optimal sequence for the turn unit. The MD trajectory suggested a clear difference in overall conformation between **3mer-I** and **3mer-II**. The latter displayed enhanced flexibility and extended conformation compared to **3mer-I**, as clearly indicated by the large fluctuations in the root-mean-square-deviation plot and the higher value of the radius of gyration (*R*_g_) (Supplementary Fig. 7). In contrast, **3mer-I** adopted a more rigid and compact turn-like conformation with a single conformer dominating nearly all the simulation time (Fig. 2a, top). MD simulations pointed at typical exo-syn-Φ/syn-Ψ conformations for all glycosidic linkages in **3mer-I** (Fig. 2a, bottom, and Supplementary Fig. 8). On the other hand, Ramachandran plots for **3mer-II** suggested a two-state situation (that is, closed versus open conformation; Fig. 2a, top, and Supplementary Fig. 12) with the β-1,4 glycosidic linkage between Glc and GlcNAc fluctuating between exo-syn-Φ/syn-Ψ and non-exo-Φ/syn-Ψ conformations (Fig. 2a, bottom, and Supplementary Fig. 9). Similarly to **3mer-I**, **3mer-III** displayed a rigid closed conformation albeit slightly more flexible, probably due to the lack of the NHAc moiety (Supplementary Figs. 7, 10 and 11). Importantly, **3mer-I** displayed the hydroxyls planned for elongation of the two strands in an ideal orientation (Supplementary Fig. 13).

We then analysed the hairpin analogues, **9mer-I** and **9mer-II**, to monitor how the turn unit impacted the overall conformation of the glycan hairpins. According to MD, only **9mer-I** adopted a closed hairpin conformation with the two cellulose strands in close proximity (Fig. 2b). The turn unit based on **3mer-I** held the two cellulose strands in an optimal orientation resulting in small inter-residue distances across the strands (between 5.0 Å and 7.2 Å; Fig. 2c, left, and Supplementary Fig. 14). In contrast, in **9mer-II** the turn unit based on **3mer-II** did not hold the two strands in proximity and the two cellulose strands tended to stay further apart (up to 18.0 Å; Fig. 2c, left, and Supplementary Fig. 14). This tendency was also reflected in the *R*_g_ and RSMD plots, which pointed at a rigid and compact shape for **9mer-I** versus a flexible, more extended shape for **9mer-II** (Fig. 2c, right, and Supplementary Fig. 15). MD simulations suggested also that larger glycan hairpins based on the **3mer-I** turn unit could in principle be obtained, even though larger fluctuations were observed for the residues located at the non-reducing termini (Supplementary Figs. 16 and 17).

### Synthesis of a glycan hairpin

**Fig. 3:**
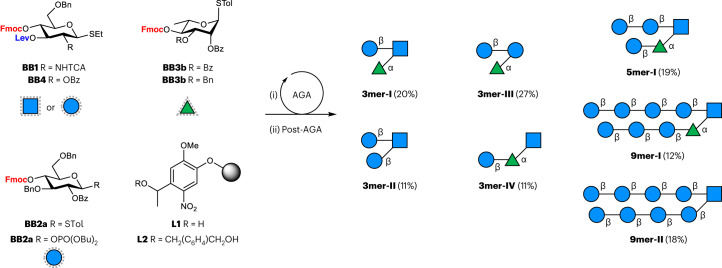
Synthesis of glycan structures. The glycan hairpin model structures were prepared by AGA using protected monosaccharide BBs (Supplementary Section 2). Overall yields are reported in parentheses. Reaction conditions for AGA and Post-AGA are reported in Supplementary Section 3.

The target compounds were synthesized by AGA (Fig. 3), a solid-phase automated synthetic method^58^. Each oligosaccharide was assembled in an overnight run using protected monosaccharide building blocks (BBs). **BB1** and **BB4** were equipped with two orthogonal protecting groups—levulinoyl ester (Lev) and 9-fluorenylmethoxycarbonyl (Fmoc)—allowing for the preparation of the three different turn units. The synthesis proceeded from the branching monosaccharide towards the non-reducing end, elongating one strand at a time, following cycles of glycosylation (modules C1 or C2; see Supplementary Section 3.3) and Fmoc deprotection (module E1). Once the first strand of the hairpin was constructed using **BB2a** or **BB2b** (refs. ^59,60^), the terminal hydroxyl group was capped (module D). Elongation of the second strand proceeded after Lev deprotection (module E2). **BB3a** was first employed for the construction of **5mer-I** and **9mer-I**, but proved to be a poor acceptor during the glycosylation of the l-Rha-OH-4. Low reactivity was also observed when we attempted the elongation of the α-1,3 branch of the hairpin glycan in the absence of the bulky β-1,4 branch, proving that the poor reactivity was not due to steric hindrance (see the AGA of **3mer-IV**, Supplementary Section 3.5). To increase the nucleophilicity of the l-Rha acceptor, we switched to **BB3b**, bearing a C-3 *O*-benzyl group instead of the EWG benzoyl group. The efficiency of the elongation of the second cellulose strand drastically improved, and no major deletion sequence side-products were observed in the synthesis of **5mer-I** and **9mer-I** (Supplementary Figs. 4 and 5). Post-AGA steps included solid-phase methanolysis, photocleavage from the solid support, and hydrogenolysis (Supplementary Section 3.4). The latter proved increasingly challenging for **5mer-I** and **9mer-I**, requiring multiple hydrogenolysis cycles to convert the *N*-trichloroacetyl protecting group to the *N*-acetyl moiety. The turn units **3mer-I**, **3mer-II** and **3mer-III** as well as the longer hairpin analogues **5mer-I**, **9mer-I** and **9mer-II** were obtained after a single final purification step in overall yields of 10–27%.

### NMR structural analysis

The structural analysis has been a major obstacle to the complete description of peptide β-sheet models. Circular dichroism provides simple and rapid information on the overall conformation, but lacks molecular details. X-ray crystallography affords molecular resolution, but fails to represent the range or ensemble of conformations that may be present in solution. NMR spectroscopy can capture the solution conformation(s) with molecular resolution and is so far the most reliable technique to characterize peptide β-sheet models^40^. Glycan structural studies are even more challenging because the lack of chromophores limits the use of circular dichroism, while difficulties in obtaining single crystals hinder the application of X-ray crystallography to glycans beyond a certain size (that is, tetrasaccharides). NMR may provide some structural information, but suffers from severe overlap between the resonances of different residues and the scarcity of inter-residue and long-range NOEs^26,61,62^.

**Fig. 4:**
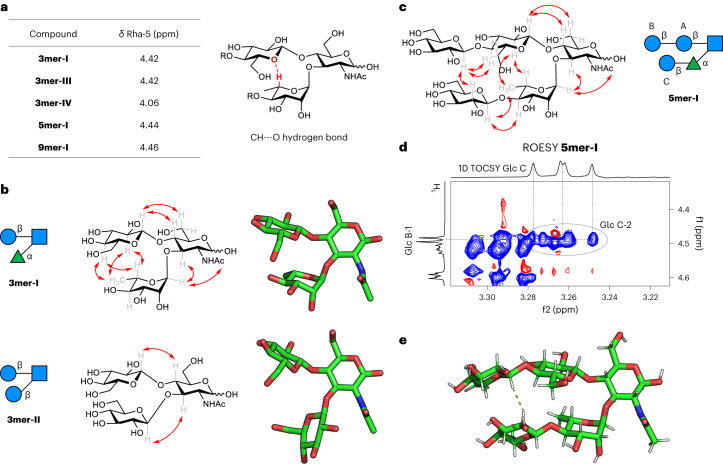
NMR analysis of turn units and 5mer-I. **a**, The chemical shift of Rha-5 reflects the presence of a non-conventional CH···O hydrogen bond, confirming the closed conformation of the turn unit in those structures. **b**, Experimental NOEs extracted from NOESY NMR experiments for **3mer-I** and **3mer-II** (red arrows). **c**, Experimental NOEs extracted from ROESY NMR experiments for **5mer-I** (red arrows). **d**, Excerpt of the 2D ROESY NMR of **5mer-I** showing the key NOE between the two strands of the hairpin (300 K, mixing time 300 ms, D_2_O, 600 MHz). **e**, Snapshot obtained from the MD simulation of **5mer-I** showing a hairpin conformation with the key experimental NOE indicated with a dotted line.

A key feature of our turn is the non-conventional CH···O hydrogen bond forming a ten-membered ring that holds the two branches in an ideal parallel orientation. Such non-conventional hydrogen bonding has been hypothesized for rhamnosylated motifs^20^, but not investigated further^63,64^. We set out to verify this hypothesis using the chemical shift deviation (Δ*δ*) of Rha-5 as an indicator^18–20^. We compared **3mer-I** and **3mer-III** with **3mer-IV**, in which no hydrogen bond can occur, observing a substantial downfield shift of Rha-5 for the first two compounds (Δ*δ* = 0.36 ppm, Fig. 4a; for details, see Supplementary Section 5.2) providing solid ground to our hypothesis.

To confirm the spatial proximity between key residues at both sides of the putative hairpin, nuclear Overhauser effect spectroscopy (NOESY) experiments were employed. The analysis of the NOESY spectra provided additional evidence that **3mer-I** adopts a closed turn-like conformation. In particular, besides the typical NOEs between protons across glycosidic linkages (Glc-1/GlcNAc-4 and Rha-1/GlcNAc-3), we observed key NOEs between the Glc and Rha moieties (Glc-2/Rha-5, Glc-2/Rha-6, Glc-4/Rha-5 and Glc-4/Rha-6) (Supplementary Figs. 29 and 30). Inter-proton distances of 2.9, 3.6, 3.6 and >4 Å, estimated by applying the isolated spin pair approximation to the NOE intensities, satisfactorily matched those calculated in the MD simulations (Supplementary Tables 1 and 2). Similar NOEs between the key proton pairs were also observed for **3mer-III**, suggesting a related conformation (Supplementary Figs. 41 and 42). The NOE pattern observed for **3mer-I** (and **3mer-III**) is fully consistent with previous reports on analogous glycan motifs (including Le^X^) (refs. ^19,20,63^), further corroborating the presence of a closed conformation. In contrast, only the standard NOEs across the glycosidic linkages were observed for **3mer-II**, in agreement with distances calculated in the MD model, indicating that the two branches are further apart compared with **3mer-I** (Fig. 4b and Supplementary Figs. 35 and 36).

The short hairpin model **5mer-I** was then scrutinized. The observed downfield shift of Rha-5 confirmed the presence of the non-conventional hydrogen bond (Δ*δ* = 0.38 ppm; Fig. 4a). The non-conventional hydrogen bond was preserved over a wide temperature range (Supplementary Fig. 24). The ROESY^65^ experiment confirmed the presence of the key inter-residue NOEs between Glc A and Rha, identical to those observed for **3mer-I** (Fig. 4c and Supplementary Figs. 47 and 52). These data prove that the closed conformation of the turn unit remains stable upon elongation of the two strands. To confirm that **5mer-I** globally folds into a hairpin conformation, additional evidence of the proximity between Glc B and Glc C on the two strands was required. The NOEs between Glc B-1 and Glc C-2 were evident in the 2D t-ROESY (Fig. 4d and Supplementary Fig. 48) and selective 1D t-ROESY (Supplementary Figs. 49–51), corresponding to an inter-proton distance of ca. 3.0 Å, in full agreement with the MD simulations. As commonly recognized for peptides, the NOEs between residues distant in sequence are a strong indication that **5mer-I** adopts a hairpin conformation (Fig. 4e).

**Fig. 5:**
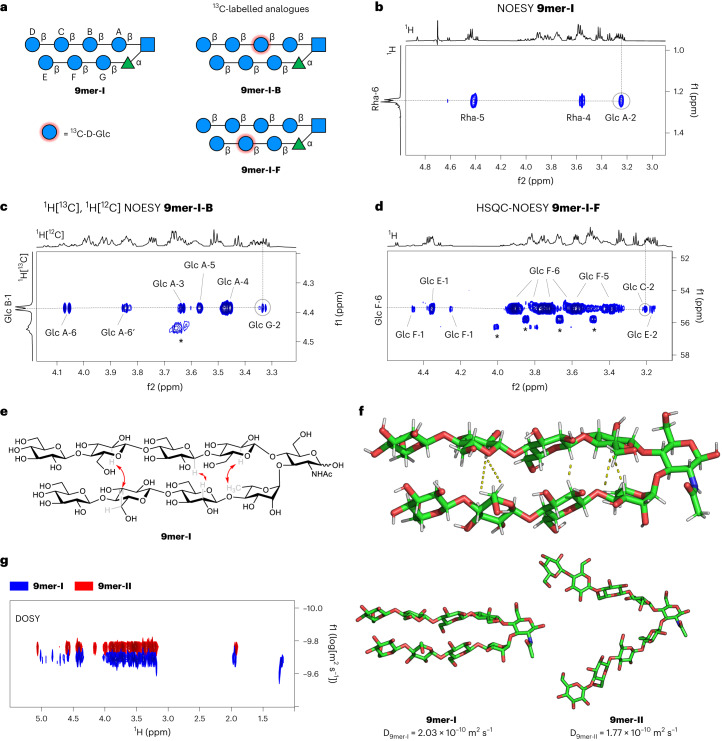
NMR analysis of the glycan hairpin. **a**, AGA enabled the rapid synthesis of two labelled analogues of **9mer-I**. For details about BBs, reaction conditions and AGA, see Supplementary Sections 2.3 and 3.5. **b**, Excerpt of the 2D NOESY NMR of **9mer-I** showing the key NOE (Rha-6/Glc A-2) at the turn unit (298 K, mixing time 300 ms, D_2_O, 800 MHz). **c**, Excerpt of the half-filtered 2D ^1^H[^13^C], ^1^H[^12^C] NOESY NMR of **9mer-I-B** showing the typical inter-residue NOEs across the glycosidic linkages and the key NOE (Glc B-1/Glc G-2) between the two strands of the hairpin (298 K, mixing time 300 ms, D_2_O, 800 MHz). The asterisk indicates signals belonging to an impurity. **d**, Excerpt of the 2D HSQC-NOESY NMR of **9mer-I-F** showing the expected intra-residue NOEs and the key NOE (Glc F-6/Glc C-2) between the two strands of the hairpin (288 K, mixing time 300 ms, D_2_O, 800 MHz). The asterisks indicate signals belonging to an impurity (Supplementary Figs. 67 and 68). **e**, Selected experimental NOEs extracted from NMR conformational experiments for **9mer-I** (red arrows) that confirm its folded conformation. **f**, Snapshot of the MD simulation of **9mer-I** showing a hairpin conformation with selected experimental NOEs indicated with dotted lines. **g**, Superimposed DOSY spectra of **9mer-I** (blue) and **9mer-II** (red) (295 K, D_2_O, 600 MHz). The corresponding diffusion coefficients are indicated, confirming that the effective hydrodynamic volume of **9mer-II** is 50% larger than that of **9mer-I**.

The next challenge was to demonstrate that the hairpin conformation is also kept in longer glycan structures. Thus, long-range inter-residue NOEs were also searched within **9mer-I**, to ultimately prove that this oligomer autonomously folds into a hairpin conformation. However, the severe chemical shift degeneracy hampered any standard structural analysis besides the observation of the hydrogen bond-induced downfield shift of Rha-5 (Δ*δ* = 0.40 ppm; Fig. 4a) and a key Rha-6/Glc A-2 NOE confirming the stability of the closed turn unit conformation over a wide temperature range (Fig. 5b and Supplementary Fig. 24).

To break the chemical shift degeneracy, and thus perform the required NMR analysis, two selectively ^13^C-labelled analogues of **9mer-I** were synthesized, employing ^**13**^**C-BB2b**, using AGA (Supplementary Sections 2.3 and 3.5). **9mer-I-B** and **9mer-I-F** present a ^13^C_6_-labelled Glc at residues B and F of the hairpin structure, respectively (Fig. 5a). The use of site-selective labelled analogues allowed extraction of the key inter-residue NOE information otherwise inaccessible. Half-filtered ^1^H[^13^C], ^1^H[^12^C] NOESY experiments, showing only inter-residue NOEs, demonstrated the presence of the Glc B-1/Glc G-2 medium size NOE, corresponding to an inter-proton distance of 2.9–3.0 Å on **9mer-I-B** (estimated by applying the isolated spin pair approximation to the NOE intensities, Fig. 5c), which was further confirmed by HSQC-NOESY (Supplementary Fig. 58). Next, we recorded HSQC-NOESY on **9mer-I-F** and detected another key medium size NOE (Glc F-6/Glc C-2, Fig. 5d), corresponding to an inter-proton distance of ca. 4.0 Å. These data unambiguously show that, even for the nonasaccharide, the hairpin conformation is kept in solution (Fig. 5e,f).

Further demonstration of the different 3D shape of **9mer-I** versus the **9mer-II** analogue was deduced from diffusion-ordered NMR spectroscopy (DOSY) experiments independently carried out for both molecules at the same concentration (Fig. 5g). A substantial difference in the diffusion coefficients estimated for both molecules (2.03 × 10^−10^ m^2^ s^−1^ for **9mer-I** and 1.77 × 10^−10^ m^2^ s^−1^ for **9mer-II**) was observed, indicating a much more compact structure for **9mer-I**. According to the Stokes–Einstein equation that describes DOSY experiments, the effective hydrodynamic volume of **9mer-II** is 50% larger than that of **9mer-I** (the ratio of hydrodynamic volumes is 1.52; Supplementary Fig. 64).

## Conclusions

We present a glycan that folds into a secondary structure not found in nature. Employing design principles developed for creating peptide hairpin structures, we designed and synthesized a glycan hairpin. We combined natural glycan structural elements to access a turn unit and two stacking strands. The glycan turn unit was inspired by the Le^X^ trisaccharide motif, adopting a closed conformation stabilized by a non-conventional hydrogen bond. The two strands were based on cellulose chains, adopting a rigid rod conformation stabilized by intramolecular hydrogen bonds and hydrophobic interactions. Atomistic MD simulations aided the design, by providing 3D models of the glycans as a blueprint for synthesis and structural analysis. A collection of synthetic hairpin analogues, including ^13^C-labelled oligosaccharides, was rapidly synthesized by AGA. The detection of long-range NOEs unambiguously demonstrated the hairpin conformation adopted by the nonasaccharide **9mer-I**, and its shorter analogues, in an aqueous solution. DOSY experiments further confirmed the propensity to adopt a closed and compact conformation.

Our work illustrates that glycan sequences capable of adopting specific secondary structure motifs can be designed. Glycans with predictable folded shapes will expand the catalogue of foldamer scaffolds. The vast pool of monosaccharides available might result in many structural motifs, beyond the commonly known natural geometries. Previously unknown natural polysaccharides are continuously discovered, and their sequences could provide a source of secondary structure motifs^66,67^. In addition, non-canonical monosaccharides^68^ and/or glycomimetics^69,70^ could be used to bring in proximity different glycan motifs or to induce folding of even shorter glycans^71^. Improvements in glycan synthesis will open up opportunities to design folded glycans^72^, allowing for the synthesis of long polysaccharides, for the construction of distinct linkages and for the production of larger quantities of materials^73^. We imagine that the ability to control the conformation of glycans could lead to predictable functions and properties, with applications in catalysis and nanotechnology.

## Methods

### Synthesis

The oligosaccharides were prepared using a home-built synthesizer designed at the Max Planck Institute of Colloids and Interfaces^58^. All details concerning BB synthesis^75,76^, AGA modules^60,77^ and post-AGA manipulations^78^ can be found in Supplementary Sections 2 and 3.

### MD simulations

All-atom MD simulations were performed using gromacs 5.1.2 (ref. ^79^). The oligosaccharides were modelled using a modified version of GLYCAM06_OSMO,r14_ force field^55,57^, and the system was solvated with TIP5P^56^ water molecules to avoid excessive interactions between the monomers. The topology was converted to gromacs format using the glycam2gmx.pl script and solvated with 2,100 water molecules using gromacs tools. The systems were kept at a constant temperature of 303 K using a Nosé–Hoover thermostat^80,81^ and at constant pressure of 1 bar with the Parrinello–Rahman barostat^82,83^. Non-bonded interactions were cut off at 1.4 nm, and long-range electrostatics were calculated using the particle mesh Ewald method^84^. Bonds involving hydrogens were constrained using the LINCS^85^ to allow a 2 fs timestep algorithm; water molecules were kept rigid with SETTLE^86^. After energy minimization (steepest descent algorithm) and before the production run, the systems were equilibrated at 300 K for 50 ns in a canonical (NVT) ensemble (constant number of particles N, volume V and temperature T) and subsequently at 300 K and 1 atm for 50 ns in an isothermal–isobaric (NPT) ensemble (constant number of particles N, pressure P and temperature T). All the modelled structures were simulated for 500 ns. Further details on MD simulations are reported in Supplementary Section 4.

### NMR analysis

^1^H, ^13^C, HSQC, 1D and 2D TOCSY, 1D and 2D ROESY, 2D NOESY NMR spectra were recorded on Varian 400-MR (400 MHz), Varian 600-NMR (600 MHz), Bruker Biospin AVANCE700 (700 MHz) Bruker AVANCE III 800 (800 MHz) spectrometers. Samples were prepared by dissolving lyophilized samples in D_2_O (concentration ~1–6 mM). Proton resonances of the oligosaccharides were assigned using a combination of ^1^H, 2D COSY, HSQC, 1D and 2D TOCSY. Selective 1D TOCSY (HOHAHA, pulse program: seldigpzs) spectra were recorded using different mixing times to assign all the resonances (d9 = 40, 80, 120, 160 and 200 ms). 2D TOCSY (pulse program: mlevphpp) spectra were recorded using different mixing times (d9 = 80 or 120 ms). Selective 1D t-ROESY (pulse program: selrogp.2) spectra were recorded using different mixing times (p15 = 100, 200, or 300 ms). 2D t-ROESY (pulse program: roesyph.2) and 2D NOESY (pulse program: noesygpphpp) spectra were recorded using different mixing times (p15 = 100, 200 or 300 ms for ROESY and d8 = 600, 800 or 1,000 ms for NOESY). The full NMR analysis of the glycans reported in the manuscript can be found in Supplementary Section 5.

### Reporting summary

Further information on research design is available in the Nature Portfolio Reporting Summary linked to this article.

## Online content

Any methods, additional references, Nature Portfolio reporting summaries, source data, extended data, supplementary information, acknowledgements, peer review information; details of author contributions and competing interests; and statements of data and code availability are available at 10.1038/s41557-023-01255-5.

### Supplementary information

Supplementary Information Supplementary Figs. 1–68, Tables 1–15 and Schemes 1–4.

Reporting Summary

## Data Availability

The authors declare that all data supporting the findings of this study are available within the article and in the supplementary information files. Raw data for NMR analysis and MD simulations can be downloaded from 10.17617/3.EG7I36, Edmond. Data are also available from the corresponding author upon request.
