# Supplementary material for: Synthesis of a glycan hairpin
Source: Nat Chem. 2023 Jul 3;15(10):1461–9. doi: 10.1038/s41557-023-01255-5 (PMC10533408; doi:10.1038/s41557-023-01255-5)
Supplement: Supplementary file 1 — Supplementary Figs. 1–68, Tables 1–15 and Schemes 1–4. [file 41557_2023_1255_MOESM1_ESM.pdf]

nature chemistry

Article

https://doi.org/10.1038/s41557-023-01255-5

Synthesis of a glycan hairpin

In the format provided by the authors and unedited

Table of contents

1 General materials and methods ... 3

2 Building blocks synthesis ... 4

2.1 Synthesis of BB3a ... 6

2.1.1 S-2 ... 6

2.1.2 BB3a ... 9

2.2 Synthesis of BB3b ... 12

2.2.1 S-3 ... 12

2.2.2 S-4 ... 15

2.2.3 BB3b ... 18

2.3 Synthesis of ^{13}C-BB2b ... 21

2.3.1 S-6 ... 21

2.3.2 S-7 ... 24

2.3.3 ^{13}C-BB2b ... 27

2.4 Synthesis of BB4 ... 31

2.4.1 S-9 ... 31

2.4.2 S-10 ... 34

2.4.3 S-11 ... 37

2.4.4 BB4 ... 40

3 Automated glycan assembly ... 43

3.1 General materials and methods ... 43

3.2 Preparation of stock solutions ... 43

3.3 Modules for automated synthesis ... 43

3.3.1 Module A: Resin preparation ... 43

3.3.2 Module B: Acidic wash with TMSOTf solution (20 min) ... 43

3.3.3 Module C1: Thioglycoside glycosylation (35 min-55 min) ... 44

3.3.4 Module C2: Glycosyl phosphate glycosylation (45 min) ... 44

3.3.5 Module D: Capping (30 min) ... 45

3.3.6 Module E1: Fmoc deprotection (9 min) ... 45

3.3.7 Module E2: Lev deprotection (90 min) ... 46

3.4 Post-AGA manipulations ... 46

3.4.1 Module F: On-resin methanolysis ... 46

3.4.2 Module G1: Cleavage from solid support ... 46

3.4.3 Module G2: Micro-cleavage from solid support ... 46

3.4.4 Module H1: Hydrogenolysis ... 46

3.4.5 Module H2: Hydrogenolysis at ambient pressure ... 47

3.4.6 Module I: Purification ... 47

3.5 Oligosaccharide synthesis 48
3.5.1 3mer-I 49
3.5.2 3mer-II 53
3.5.3 3mer-III 57
3.5.4 3mer-IV 61
3.5.5 5mer-I 65
3.5.6 9mer-I 69
3.5.7 9mer-II 74
3.5.8 9mer-I-B 79
3.5.9 9mer-I-F 84
3.6 Comparison of BB3a and BB3b for AGA of 5mer-I and 9mer-I 89
3.7 Comparison of ^{1}H NMR of ^{13}C-labelled compounds 90
4 Molecular dynamics simulations 91
4.1 General materials and methods 91
4.2 RMSD and RoG analysis of 3mer-I, 3mer-II, and 3mer-III 92
4.3 Dihedral comparison of 3mer-I, 3mer-II, and 3mer-III 93
4.4 Comparison of 3mer-II major and minor conformations 95
4.5 Comparison of 3mer-I with 3mer-II 96
4.6 RMSD and RoG analysis of 9mer-I and 9mer-II 97
4.7 Comparison of hairpin with different length 98
4.8 Inter-proton distances for 3mer-I 100
4.9 Inter-proton distances for 3mer-II 102
4.10 Inter-proton distances for 3mer-III 104
4.11 Inter-proton distances for 5mer-I 106
4.12 Inter-proton distances for 9mer-I 108
5 NMR studies 110
5.1 General materials and methods 110
5.2 Determination of the non-conventional hydrogen bond 111
5.3 NMR characterization of 3mer-I 115
5.4 NMR characterization of 3mer-II 119
5.5 NMR characterization of 3mer-III 123
5.6 NMR characterization of 5mer-I 127
5.7 NMR characterization of 9mer-I 133
5.8 NMR characterization of 9mer-I-B 135
5.9 NMR characterization of 9mer-I-F 138
5.10 Diffusion experiments (DOSY) 141
5.11 Degradation of 9mer-I 144
6 References 146

1 General materials and methods

All chemicals used were reagent grade and used as supplied unless otherwise noted. The automated syntheses were performed on a home-built synthesizer developed at the Max Planck Institute of Colloids and Interfaces. Analytical thin-layer chromatography (TLC) was performed on Merck silica gel 60 F254 plates (0.25 mm). Compounds were visualized by UV irradiation or dipping the plate in a staining solution (sugar stain: 10% H_2SO_4 in EtOH; CAM: 48 g/L ammonium molybdate, 60 g/L ceric ammonium molybdate in 6% H_2SO_4 aqueous solution). Flash column chromatography was carried out by using forced flow of the indicated solvent on Fluka Kieselgel 60 M (0.04 – 0.063 mm). Analysis and purification by normal and reverse phase HPLC was performed by using an Agilent 1200 series. Products were lyophilized using a Christ Alpha 2-4 LD plus freeze dryer. ^1H, ^{13}C and HSQC NMR spectra were recorded on a Varian 400-MR (400 MHz), Varian 600-NMR (600 MHz), Bruker Biospin AVANCE700 (700 MHz) Bruker AVANCE III 800 (800 MHz) spectrometer. Spectra were recorded in $CDCl_3$ by using the solvent residual peak chemical shift as the internal standard ($CDCl_3$: 7.26 ppm ^1H, 77.0 ppm ^{13}C) or in D_2O using the solvent as the internal standard in ^1H NMR (D_2O: 4.79 ppm ^1H). ^1H NMR spectra for all compounds were recorded without ^{13}C decoupling. Weak intensity ^{13}C resonances were derived from the respective HSQC crosspeaks. ^1H NMR integrals of the resonances corresponding to residues at the reducing end are reported as non-integer numbers and the sum of the integrals of α and β anomers is set to 1. High resolution mass spectra were obtained using a 6210 ESI-TOF mass spectrometer (Agilent) and a MALDI-TOF autoflexTM (Bruker). MALDI and ESI mass spectra were run on IonSpec Ultima instruments. IR spectra were recorded on a Perkin-Elmer 1600 FTIR spectrometer. Optical rotations were measured by using a Perkin-Elmer 241 and Unipol L1000 polarimeter.

2 Building blocks synthesis

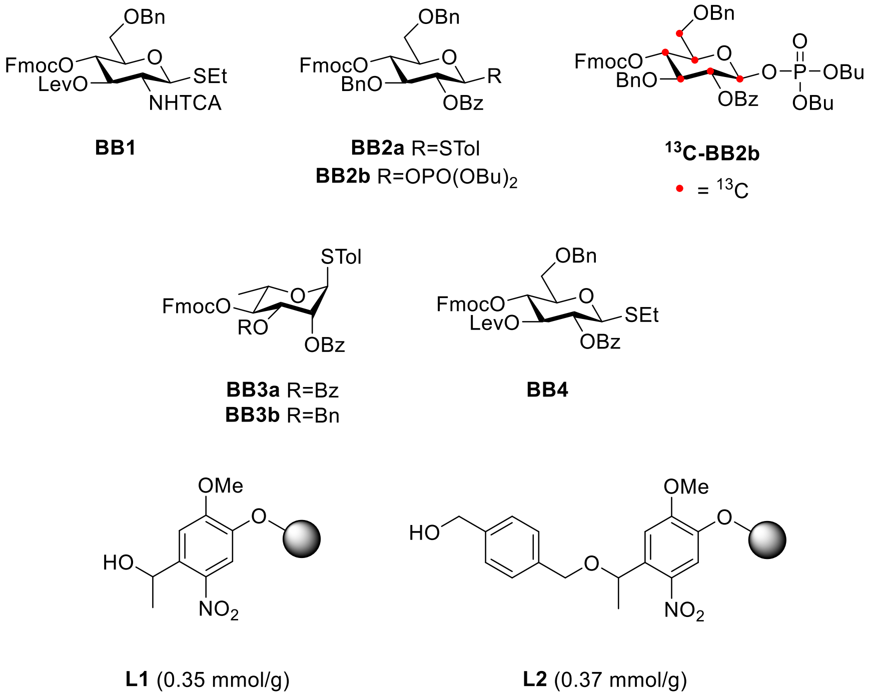

Figure 1 BBs and solid supports used in this work. Loading of **L1** and **L2** is reported in parenthesis.

BB1, **BB2a** were purchased from GlycoUniverse (Germany). **BB2b** was synthesized according to previously reported procedures.[1] The syntheses of **^{13}C-BB2b, BB3a**, **BB3b**, and **BB4** are described herein (**Scheme 2**, **3**, **4**, and **5**). Merrifield resin equipped with photocleavable linkers **L1** (loading 0.35 mmol/g) or **L2** (loading 0.37 mmol/g) was prepared according to previously reported procedures.[2]

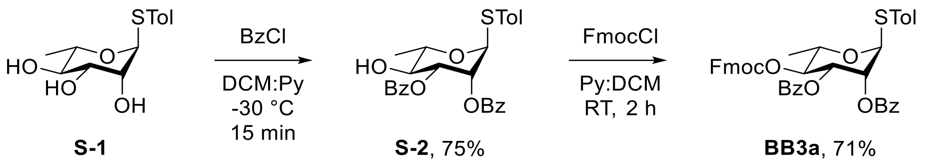

Scheme 1 Synthetic route for **BB3a**.

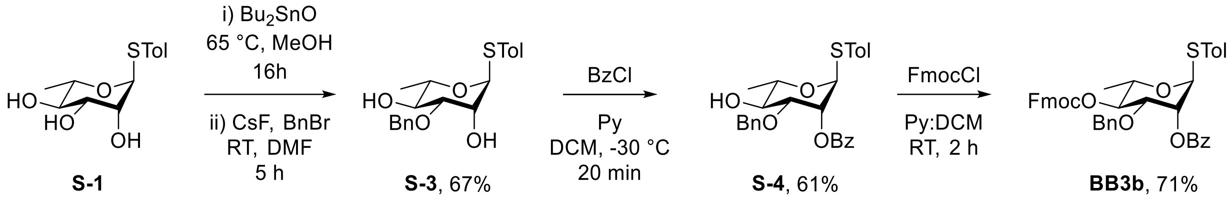

Scheme 2 Synthetic route to **BB3b**.

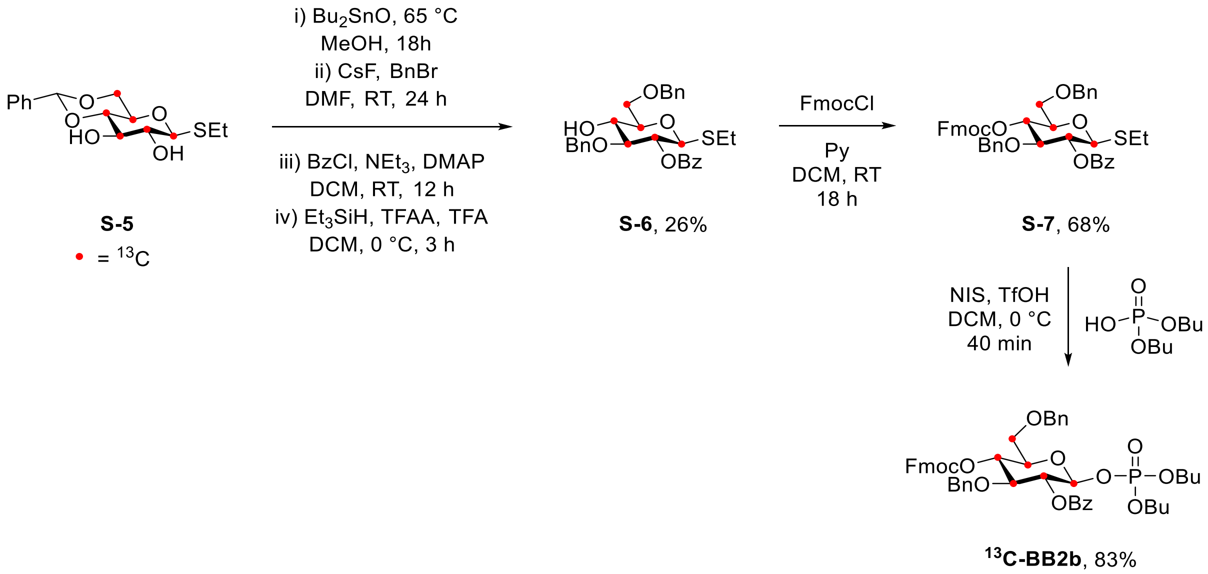

Scheme 3 Synthetic route for ^{13}**C-BB2b**.

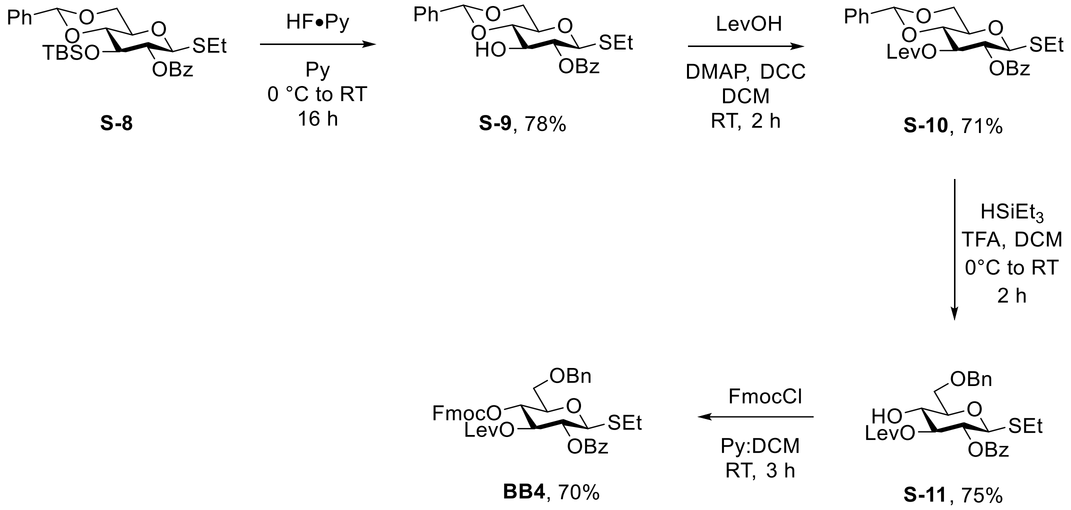

Scheme 4 Synthetic route for **BB4**.

2.1 Synthesis of BB3a

2.1.1 S-2

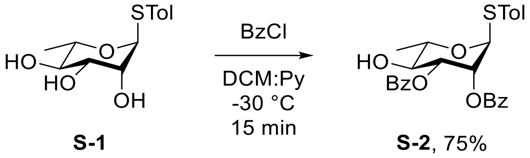

S-1 was synthesized according to previously reported procedures.[3]

S-1 (2.27 g, 8.4 mmol) was dissolved in a 1:1 mixture of DCM:Py anhydrous (35 mL) and cooled to -35 °C (ACN:dry ice bath) under Ar atmosphere. A solution of BzCl (2.2 mL in 6.5 mL of DCM, 19.0 mmol) was added dropwise over 5 min while stirring. The reaction was kept between -35 and -30 °C and the reaction progress was monitored every 3 min by TLC. Upon complete consumption of **S-1** (ca. 15 min), the reaction was quenched with a saturated aqueous solution of $NaHCO_3$. The reaction mixture was diluted with DCM, washed three times with saturated aqueous solution of $NaHCO_3$ and once with brine. The organic layer was dried with Na_2SO_4 and concentrated under reduce pressure. The crude product was purified by silica gel flash column chromatography (Hexane : Acetone, 4:1→3:1→2:1) to yield **S-2** as a sticky colorless solid (3.03 g, 75%).

^{1}H NMR (400 MHz, $CDCl_3$) δ 8.05 (dd, *J* = 8.3, 1.2 Hz, 2H), 7.93 (dd, *J* = 8.3, 1.2 Hz, 2H), 7.63 – 7.57 (m, 1H), 7.56 – 7.50 (m, 1H), 7.47 (t, *J* = 7.7 Hz, 2H), 7.42 (d, *J* = 8.1 Hz, 2H), 7.36 (t, *J* = 7.8 Hz, 2H), 7.14 (d, *J* = 7.9 Hz, 2H), 5.84 (dd, *J* = 3.3, 1.6 Hz, 1H), 5.55 – 5.49 (m, 2H), 4.40 (dq, *J* = 9.4, 6.2 Hz, 1H), 4.00 (t, *J* = 9.6 Hz, 1H), 2.33 (s, 3H), 1.46 (d, *J* = 6.2 Hz, 3H). ^{13}C NMR (101 MHz, $CDCl_3$) δ 166.96, 165.56, 138.32, 133.63, 132.76, 130.11, 130.01, 129.96, 128.69, 128.58, 86.41, 73.59, 72.54, 72.36, 70.13, 21.30, 17.76. $[\alpha]_D^{20}$ -41.37 (c = 1.07 g/100mL, $CHCl_3$). IR (film) ν = 3495, 1726, 1280, 1095, 1071 cm^{-1}. R_f = 0.43 (Hexane : Acetone 2:1). ESI-HRMS m/z 501.1401 $[M+Na]^+$ ($C_{27}H_{26}O_6SNa$ requires 501.1342).

^{1}H NMR of S-2 (400 MHz, $CDCl_3$)

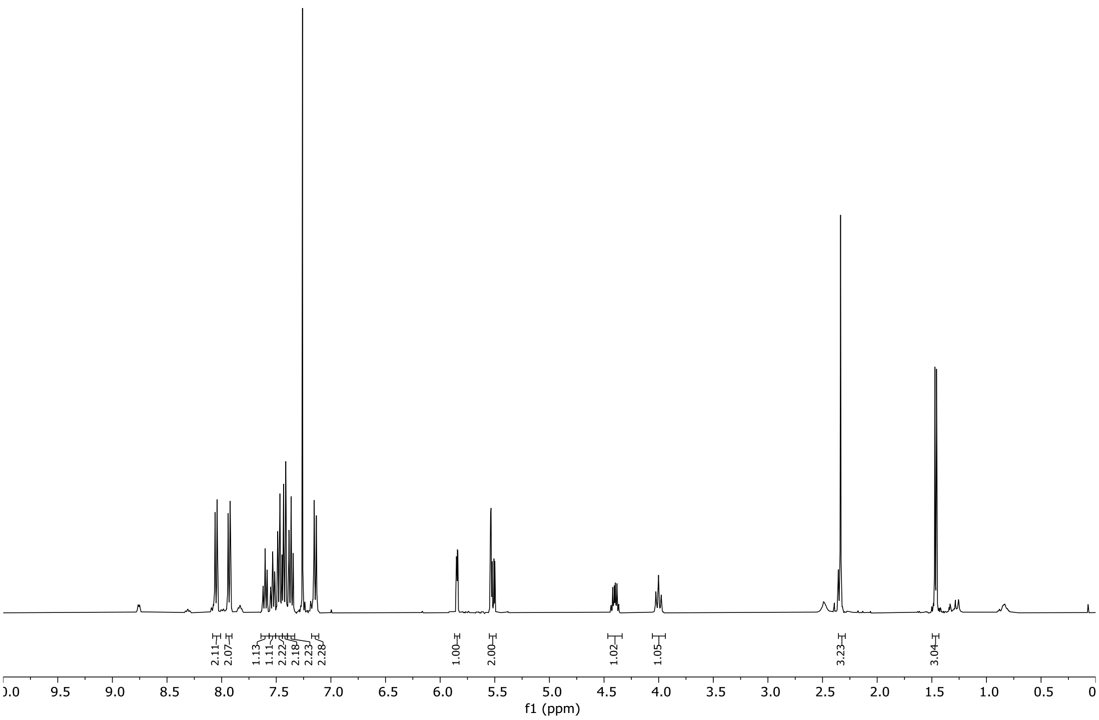

^{13}C NMR of S-2 (101 MHz, $CDCl_3$)

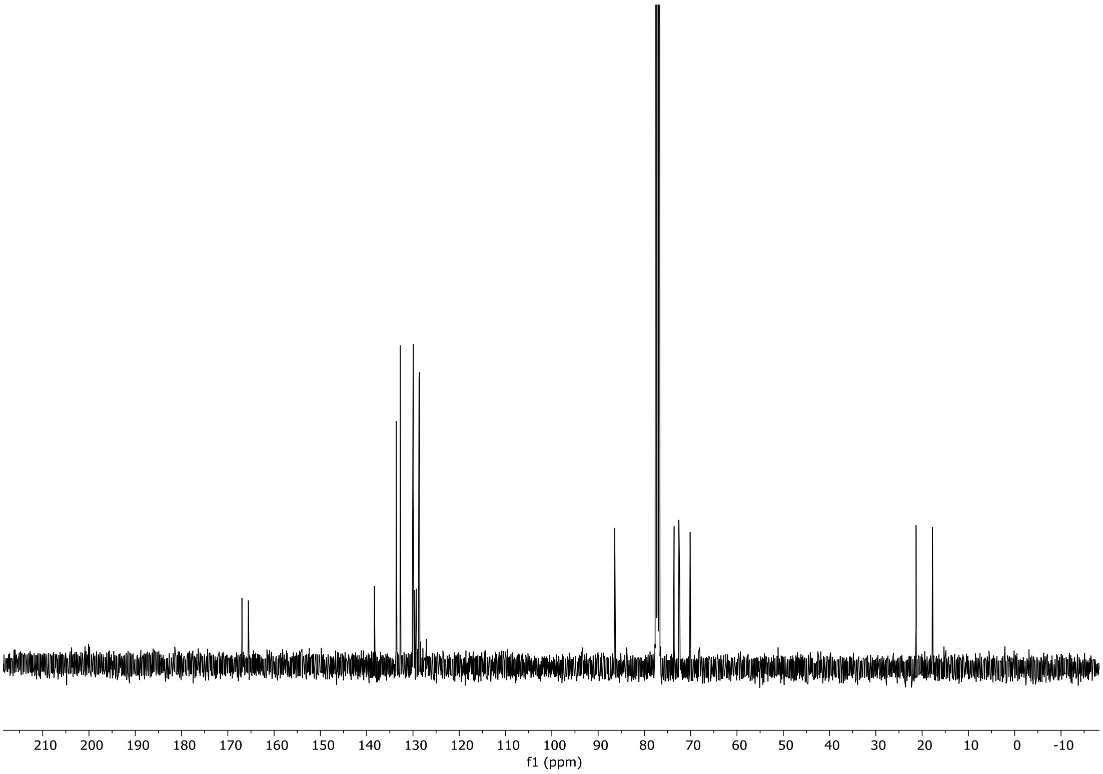

COSY NMR of S-2 ($CDCl_3$)

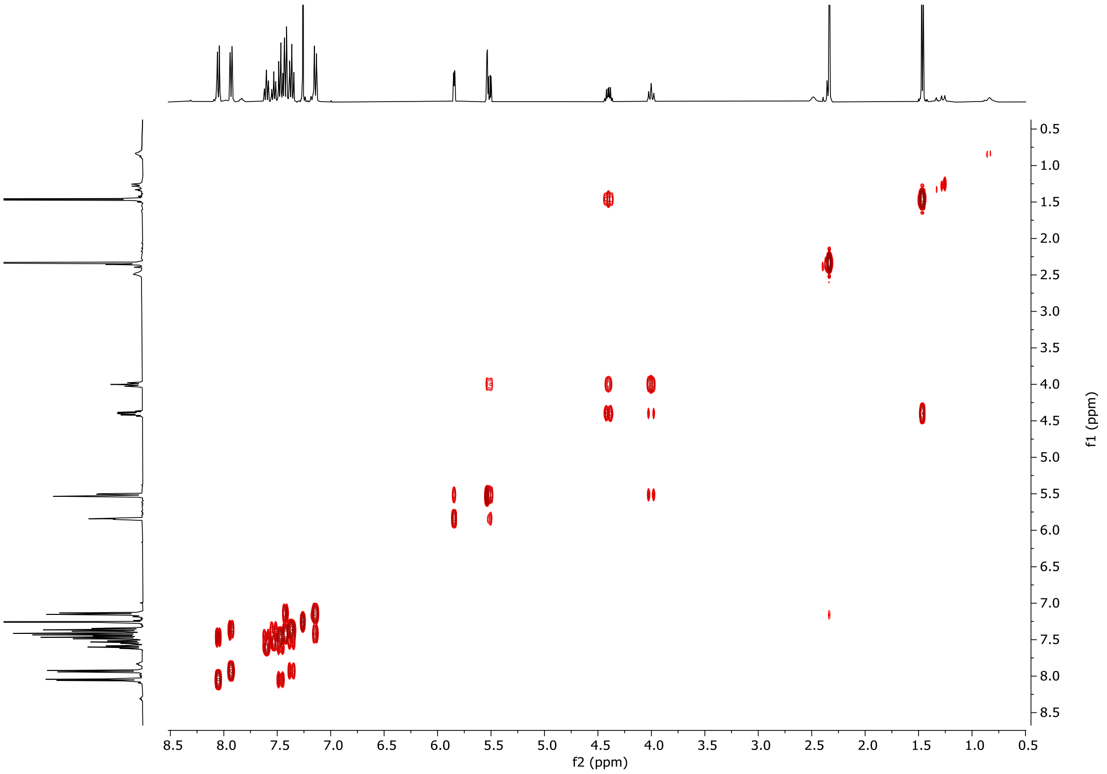

HSQC NMR of S-2 ($CDCl_3$)

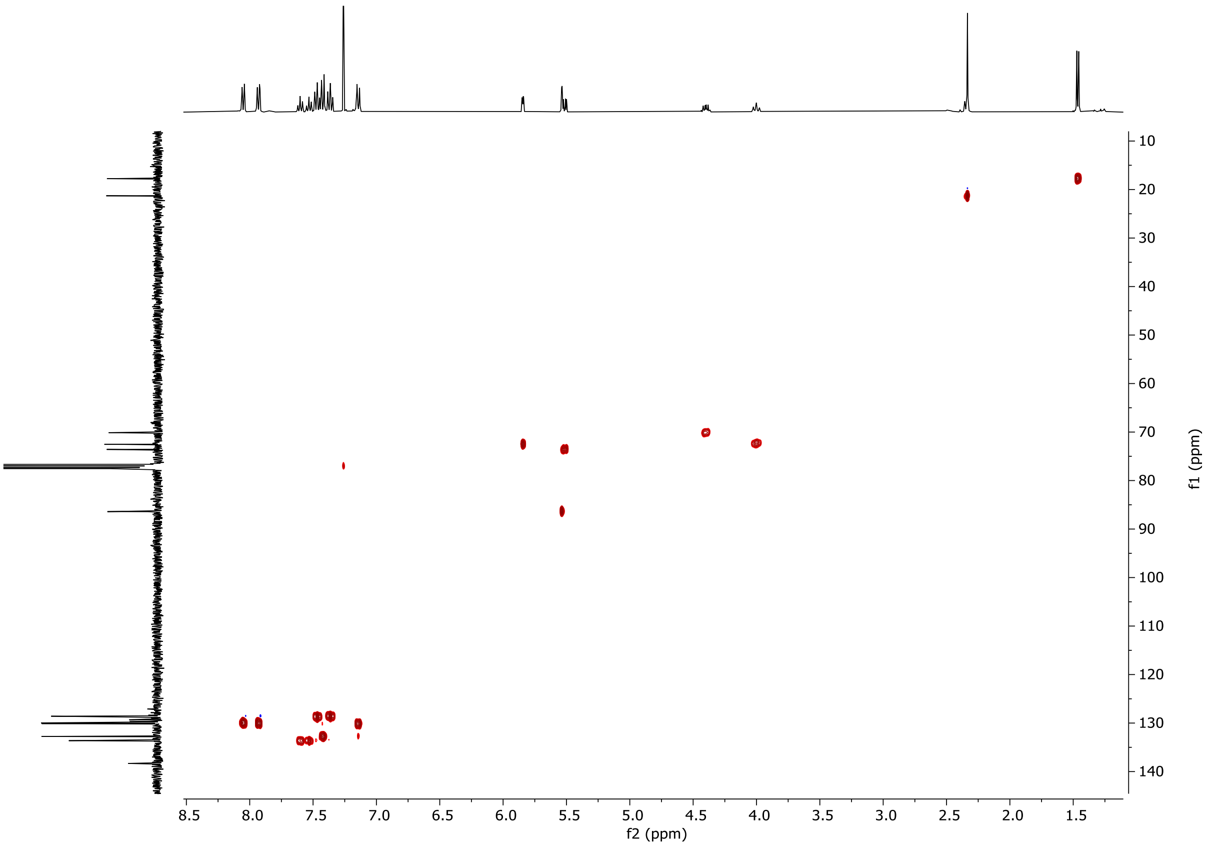

2.1.2 BB3a

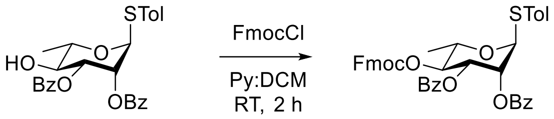

S-2 **BB3a**, 71%

S-2 (3.03 g, 6.3 mmol) was dissolved in DCM:Py anhydrous (3:1, 30 mL) and cooled to 0 °C under Ar atmosphere. FmocCl (3.2 g, 12.4 mmol) was dissolved in DCM (5 mL) and added dropwise to the stirred reaction mixture. The solution was stirred for 30 min at 0 °C and then allowed to RT and stirred for additional 2 h, after which time the reaction was quenched with an aqueous solution of citric acid (1 M). The reaction mixture was diluted with DCM, washed twice with an aqueous solution of citric acid (1 M) and once with brine. The organic layer was dried over Na_2SO_4 and concentrated under reduced pressure. The crude product was purified by silica gel flash column chromatography (Hexane : Acetone, 4:1→3:1) and recrystallized from DCM : Hexane to yield **BB3a** as a sticky colorless solid (3.18 g, 71%).

^{1}H NMR (400 MHz, $CDCl_3$) δ 8.06 – 8.01 (m, 2H), 7.92 – 7.87 (m, 2H), 7.72 (dd, *J* = 7.5, 5.0 Hz, 2H), 7.58 (t, *J* = 7.4 Hz, 1H), 7.50 – 7.40 (m, 7H), 7.35 (dt, *J* = 14.3, 7.5 Hz, 2H), 7.30 – 7.13 (m, 6H), 5.90 (dd, *J* = 3.3, 1.5 Hz, 1H), 5.72 (dd, *J* = 10.1, 3.3 Hz, 1H), 5.57 (d, *J* = 1.1 Hz, 1H), 5.30 (t, *J* = 9.9 Hz, 1H), 4.68 – 4.58 (m, 1H), 4.41 – 4.30 (m, 2H), 4.10 (t, *J* = 7.3 Hz, 1H), 2.35 (s, 3H), 1.38 (d, *J* = 6.2 Hz, 3H). ^{13}C NMR (101 MHz, $CDCl_3$) δ 165.54, 165.49, 154.97, 143.37, 142.99, 141.38, 141.30, 138.47, 133.66, 133.47, 132.78, 130.19, 130.01, 129.96, 129.51, 129.45, 129.19, 128.73, 128.50, 128.01, 127.30, 125.20, 125.06, 120.15, 86.25, 75.91, 72.42, 70.52, 70.36, 67.67, 46.71, 21.33, 17.56. $[\alpha]_D^{20}$ 13.48 (c = 0.78 g/100mL, $CHCl_3$). IR (film) ν = 1754, 1730, 1282, 1248, 1103, 711 cm^{-1}. R_f = 0.54 (Hexane : Acetone 2:1). ESI-HRMS m/z 723.2101 $[M+Na]^+$ ($C_{42}H_{36}O_8SNa$ requires 723.2023).

^{1}H NMR of BB3a (400 MHz, $CDCl_3$)

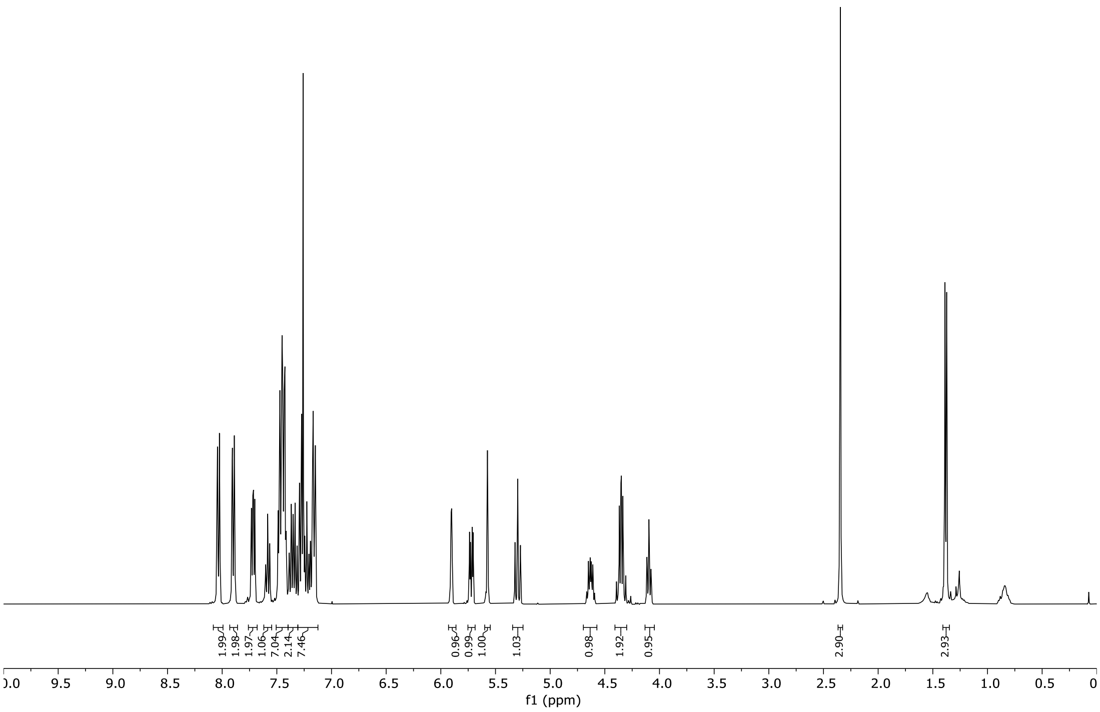

^{13}C NMR of BB3a (101 MHz, $CDCl_3$)

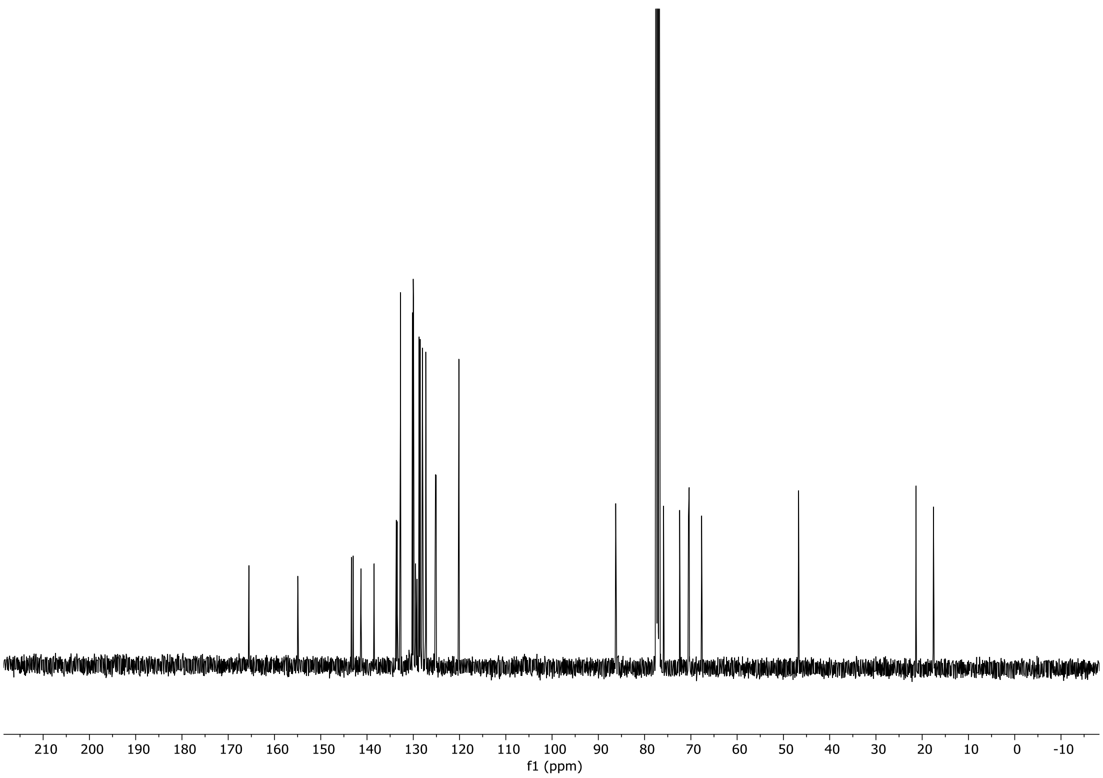

COSY NMR of BB3a ($CDCl_3$)

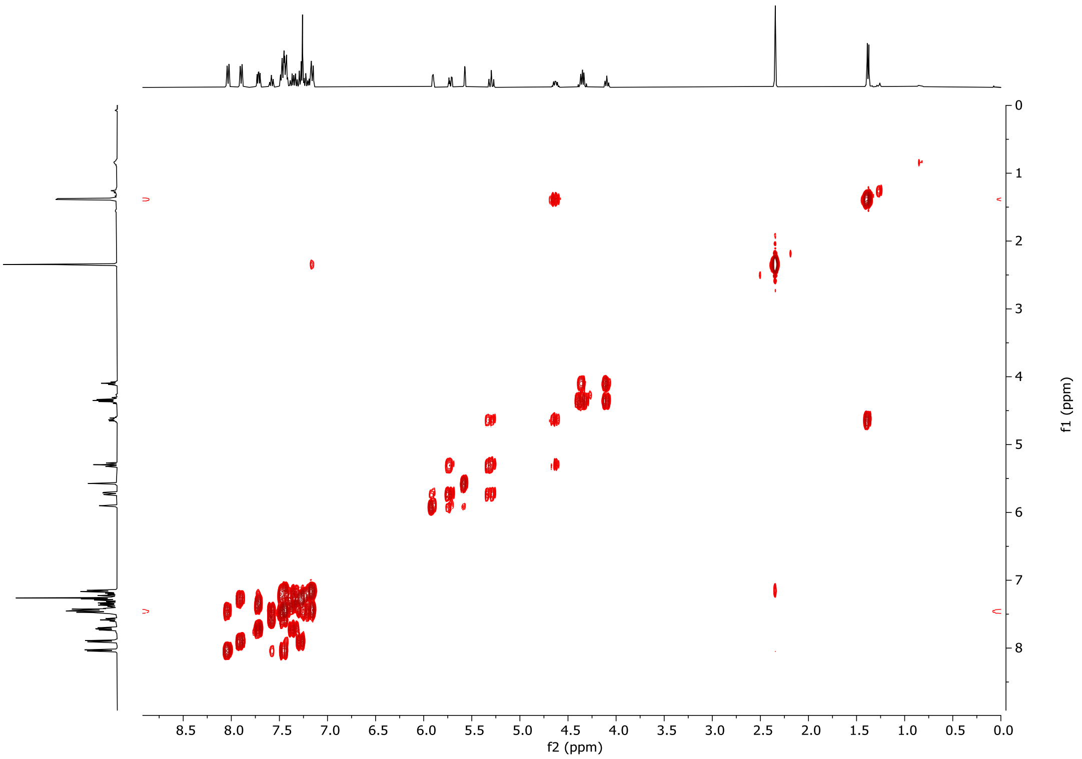

HSQC NMR of BB3a ($CDCl_3$)

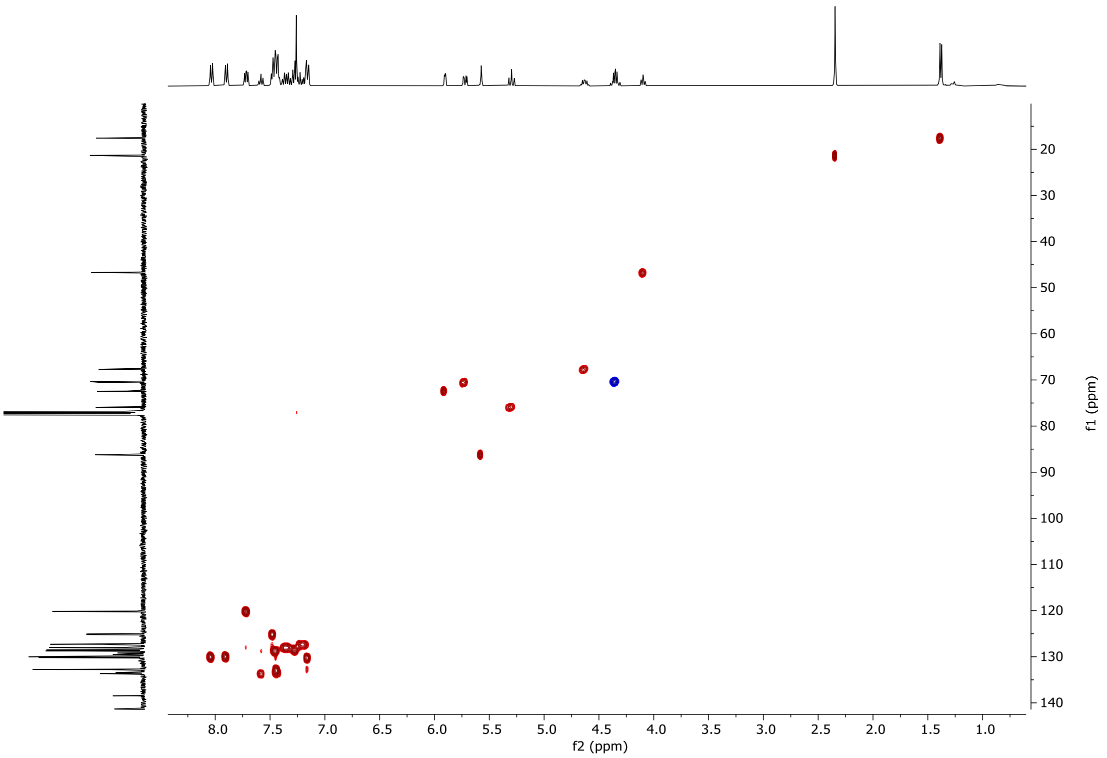

2.2 Synthesis of BB3b

2.2.1 S-3

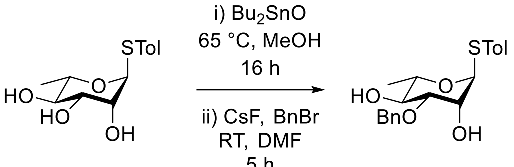

S-1 **S-3**, 67%

S-1 was synthesized according to previously reported procedures.[3]

S-1 (2.98 g, 11.0 mmol) was dissolved in MeOH (100 mL), di-*n*-butyltin oxide (3.28 g, 13.2 mmol) was added and the reaction mixture (white suspension) heated at reflux at 65 °C while vigorously stirring for 16 h. The reaction mixture (clear solution) was then cooled, concentrated under reduced pressure and the crude product was used in the next step without further purification. The crude product was dissolved in DMF (50 mL), benzyl bromide (1.6 mL, 13.5 mmol) and cesium(I) fluoride (2.2 g, 14.5 mmol) were added and the clear solution stirred at RT for 5 h under Ar atmosphere. The reaction mixture (cloudy) was diluted with EtOAc and the organic layer was passed through a short plug of silica gel and dried under reduced pressure. The crude product was then diluted with EtOAc and the organic layer washed once with an aqueous solution of KF (1 M), dried with Na_2SO_4 and concentrated under reduced pressure. The crude product was purified by silica gel flash column chromatography (Hexane : Acetone, 4:1→3:1→2:1→1:1) to yield **S-3** as a sticky colorless solid (2.67 g, 67%).

^{1}H NMR (400 MHz, $CDCl_3$) δ 7.42 – 7.30 (m, 7H), 7.12 (d, *J* = 8.0 Hz, 2H), 5.49 – 5.44 (m, 1H), 4.73 (d, *J* = 11.5 Hz, 1H), 4.61 (d, *J* = 11.5 Hz, 1H), 4.27 – 4.23 (m, 1H), 4.16 (quint, *J* = 6.2 Hz, 1H), 3.70 – 3.59 (m, 2H), 2.33 (s, 3H), 1.31 (d, *J* = 6.2 Hz, 3H). ^{13}C NMR (101 MHz, $CDCl_3$) δ 137.81, 137.47, 132.15, 130.28, 129.98, 128.92, 128.50, 128.21, 87.78, 79.95, 71.95, 71.92, 69.47, 69.12, 21.23, 17.66. $[\alpha]_D^{20}$ -158.13 (c = 1.00 g/100mL, $CHCl_3$). IR (film) ν = 3437, 2923, 1494, 1100, 1061 cm^{-1}. R_f = 0.46 (Hexane : Acetone 2:1). ESI-HRMS m/z 383.1288 [M+Na]$^+$ ($C_{20}H_{24}O_4SNa$ requires 383.1287).

^{1}H NMR of S-3 (400 MHz, $CDCl_3$)

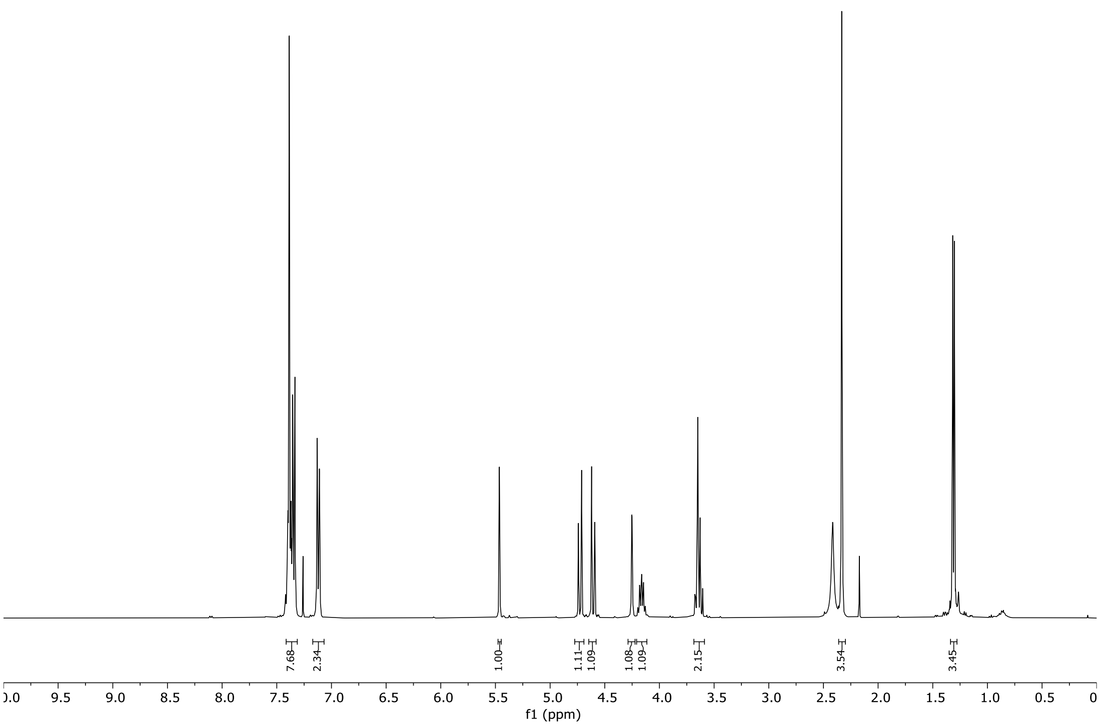

^{13}C NMR of S-3 (101 MHz, $CDCl_3$)

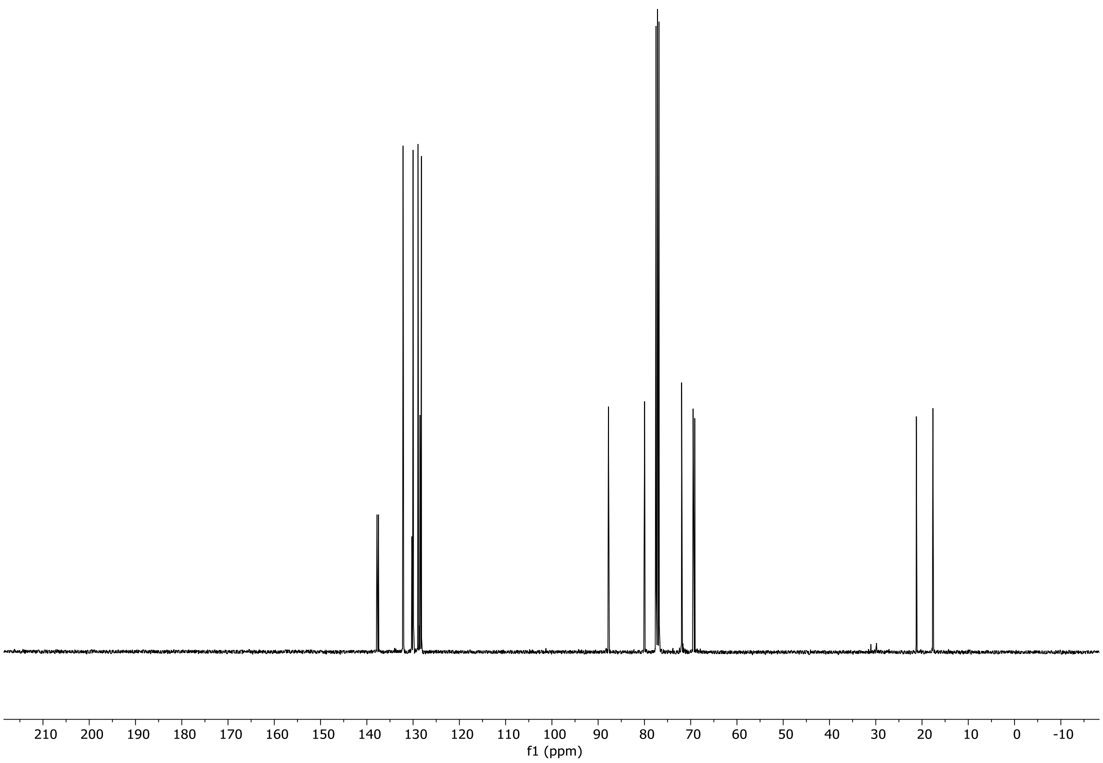

COSY NMR of S-3 ($CDCl_3$)

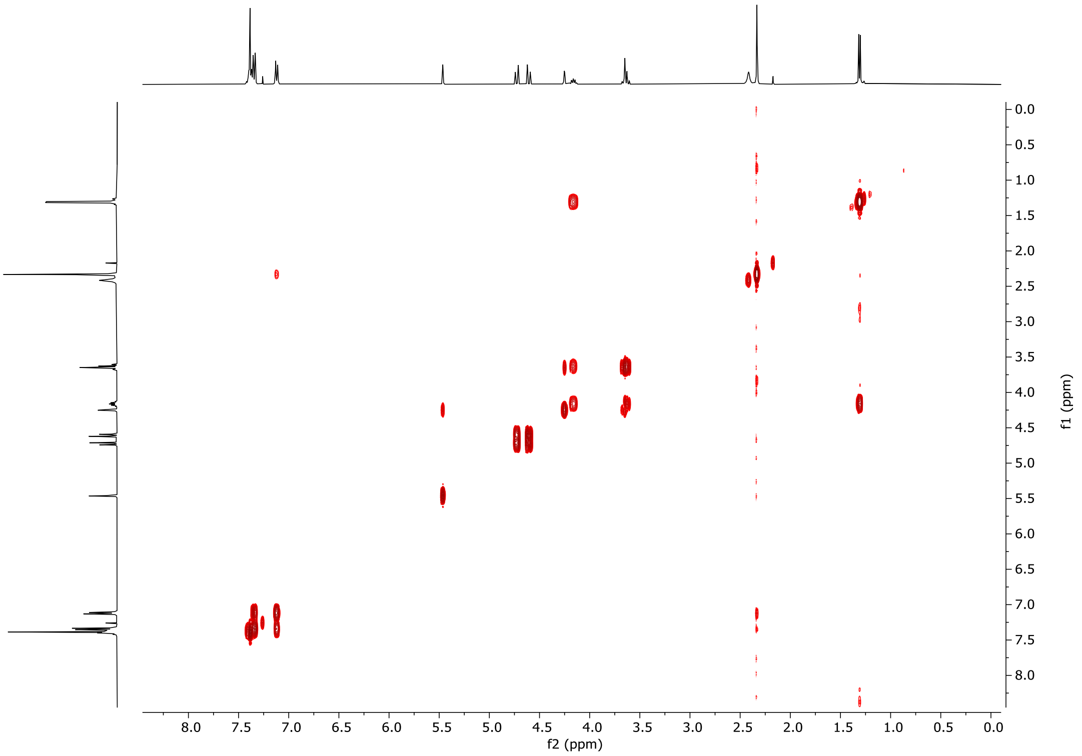

HSQC NMR of S-3 ($CDCl_3$)

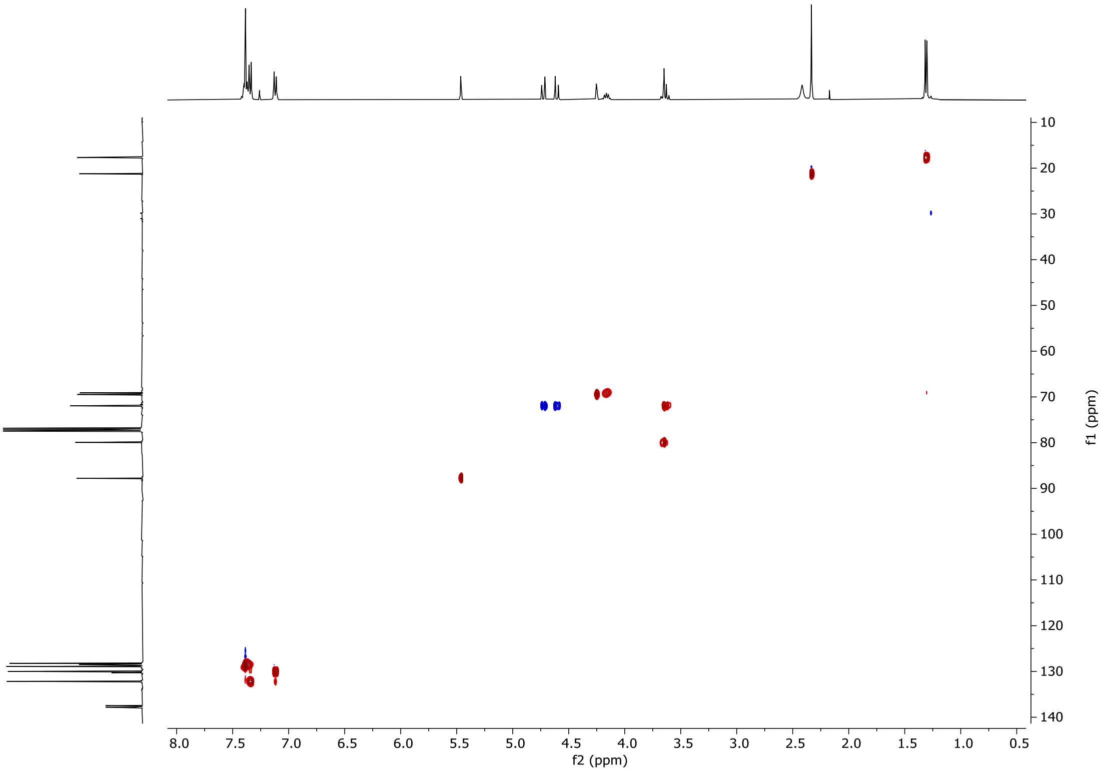

2.2.2 S-4

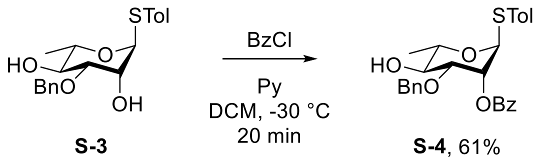

S-3 (2.07 g, 5.7 mmol) was dissolved in a 1:1 mixture of DCM:Py anhydrous (30 mL) and cooled to -35 °C (ACN:dry ice bath) under Ar atmosphere. A solution of BzCl (790 µL in 4 mL of DCM, 6.8 mmol) was added dropwise over 5 min while stirring. The reaction was kept between -35 and -30 °C and the reaction progress was monitored every 3 min by TLC. Upon complete consumption of **S-3** (ca. 20 min), the reaction was quenched with a saturated aqueous solution of $NaHCO_3$. The reaction mixture was diluted with DCM, washed three times with saturated aqueous solution of $NaHCO_3$ and once with brine. The organic layer was dried over Na_2SO_4 and concentrated under reduce pressure. The crude product was purified by silica gel flash column chromatography (Hexane : Acetone, 4:1→3:1→2:1) and recrystallized from DCM : Hexane to yield **S-4** as a sticky colorless solid (1.61 g, 61%).

^{1}H NMR (400 MHz, $CDCl_3$) δ 8.09 – 8.02 (m, 2H), 7.58 (tt, *J* = 7.0, 1.3 Hz, 1H), 7.49 – 7.42 (m, 2H), 7.42 – 7.36 (m, 2H), 7.35 – 7.27 (m, 5H), 7.13 (d, *J* = 7.9 Hz, 2H), 5.85 (dd, *J* = 2.8, 1.7 Hz, 1H), 5.51 (d, *J* = 1.3 Hz, 1H), 4.81 (d, *J* = 11.2 Hz, 1H), 4.50 (d, *J* = 11.2 Hz, 1H), 4.27 (dq, *J* = 8.6, 6.2 Hz, 1H), 3.85 – 3.73 (m, 2H), 2.33 (s, 3H), 1.40 (d, *J* = 6.2 Hz, 3H). ^{13}C NMR (101 MHz, $CDCl_3$) δ 165.77, 138.17, 137.40, 133.46, 132.54, 130.10, 130.05, 129.99, 128.73, 128.57, 128.39, 128.26, 86.87, 78.12, 72.30, 71.54, 70.27, 69.46, 21.26, 17.90. $[\alpha]_D^{20}$ -36.20 (c = 1.00 g/100mL, $CHCl_3$). IR (film) ν = 3496, 1721, 1268, 1109, 1070, 711 cm^{-1}. R_f = 0.37 (Hexane : Acetone = 3:1). ESI-HRMS m/z 487.1548 [M+Na]$^+$ ($C_{27}H_{28}O_5SNa$ requires 487.1549).

^1H NMR of S-4 (400 MHz, $CDCl_3$)

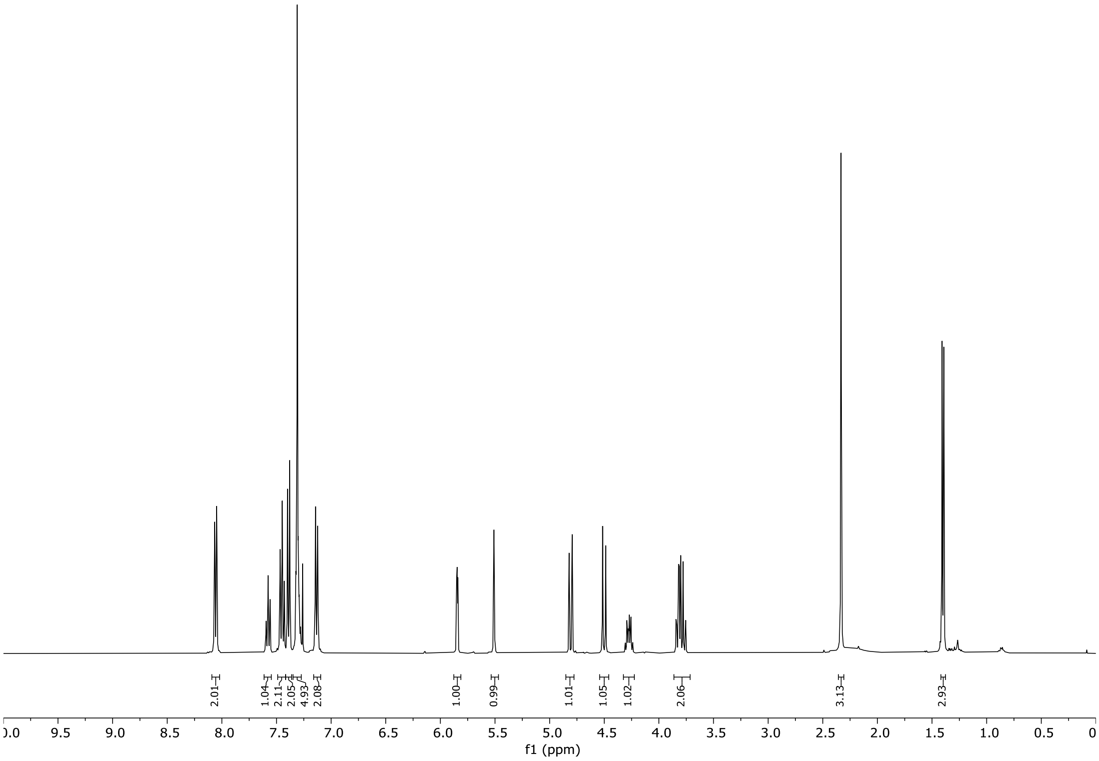

^{13}C NMR of S-4 (101 MHz, $CDCl_3$)

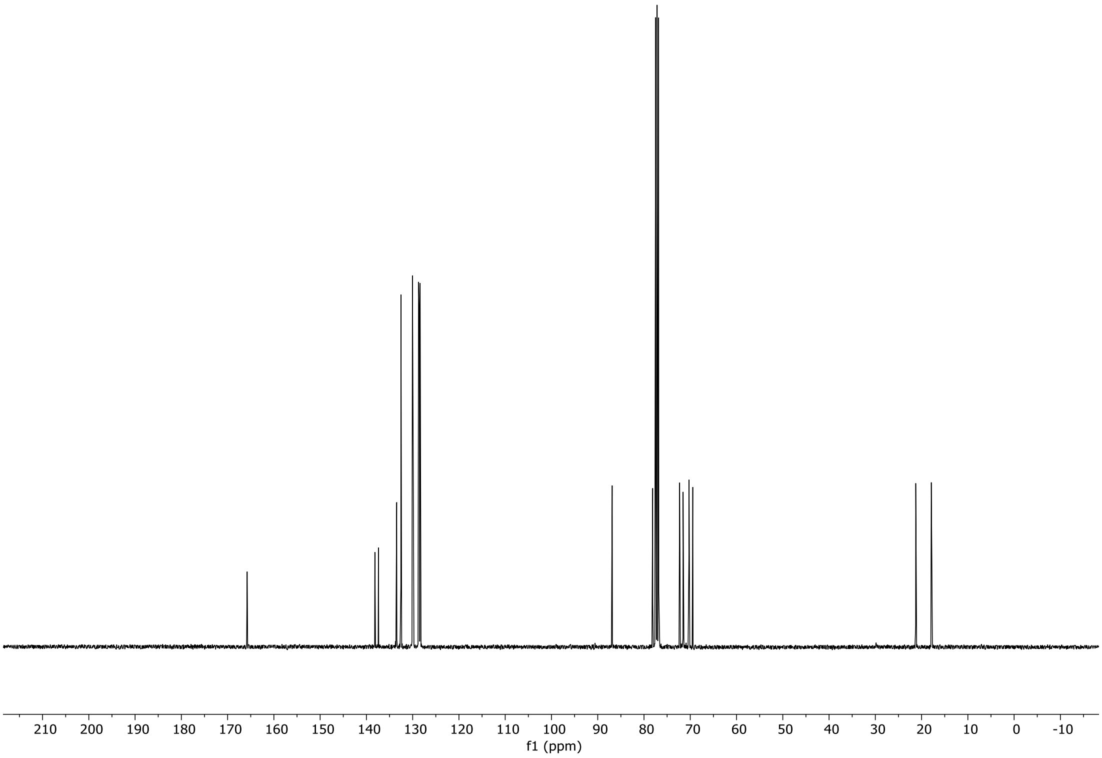

COSY NMR of S-4 ($CDCl_3$)

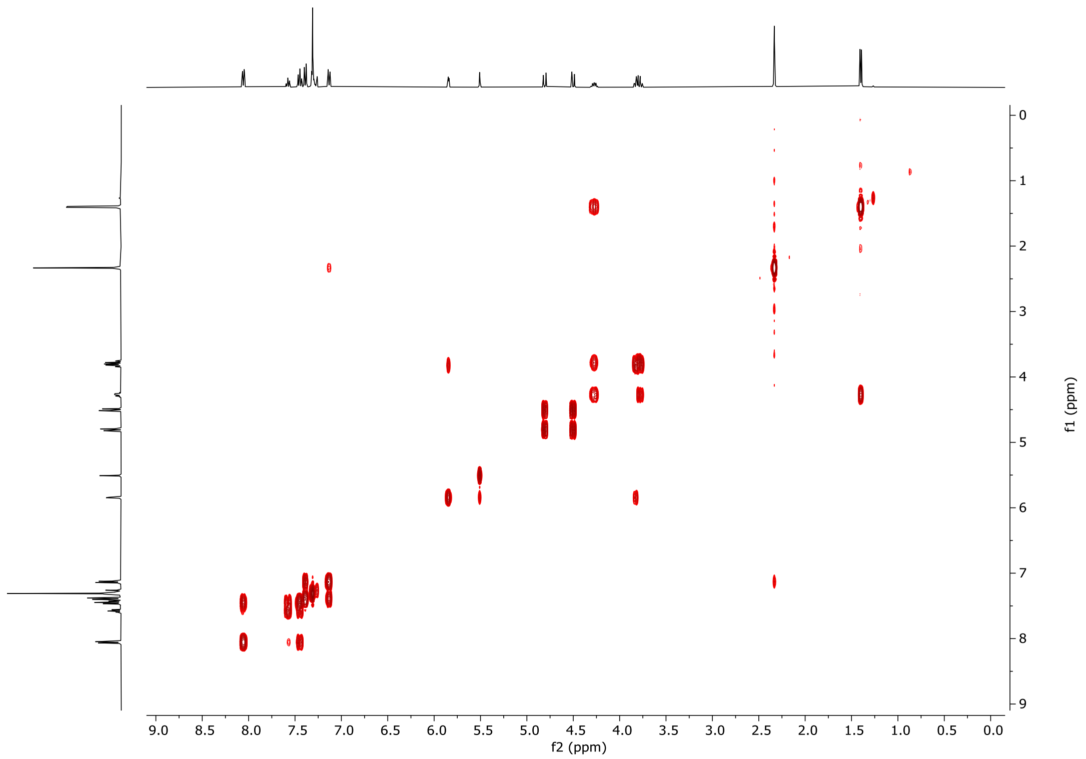

HSQC NMR of S-4 ($CDCl_3$)

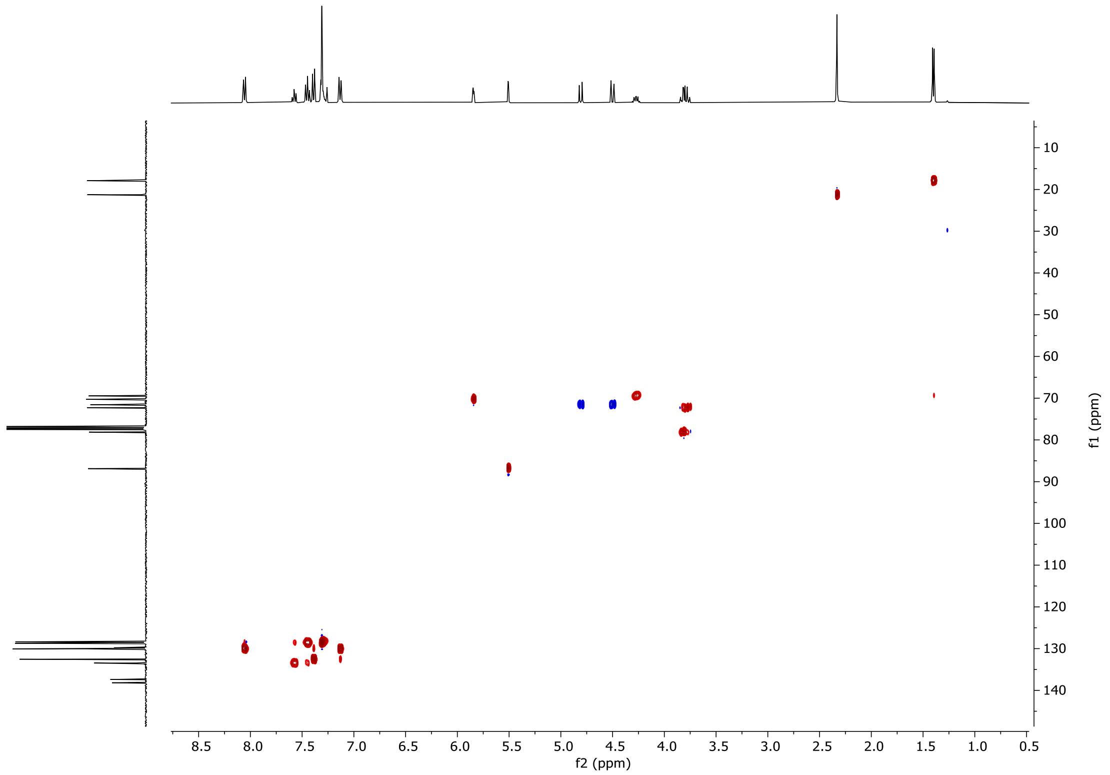

2.2.3 BB3b

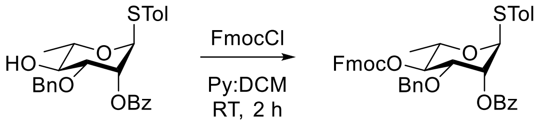

S-4 **BB3b**, 71%

S-4 (1.61 g, 3.5 mmol) was dissolved in DCM:Py anhydrous (3:1, 20 mL) and cooled to 0 °C under Ar atmosphere. FmocCl (1.79 g, 6.9 mmol) was dissolved in DCM (6 mL) and added dropwise to the stirred reaction mixture. The solution was stirred for 15 min at 0 °C and then allowed to RT and stirred for additional 2 h, after which time the reaction was quenched with an aqueous solution of citric acid (1 M). The reaction mixture was diluted with DCM, washed once with an aqueous solution of citric acid (1 M), and once with brine. The organic layer was dried over Na_2SO_4 and concentrated under reduced pressure. The crude product was purified using automated silica gel flash column chromatography (Hexane : Acetone, linear gradient of Acetone from 5% to 30%) to yield **BB3b** as a sticky colorless solid (1.70 g, 71%).

^{1}H NMR (400 MHz, $CDCl_3$) δ 8.11 – 8.03 (m, 2H), 7.79 (d, *J* = 7.5 Hz, 2H), 7.63 (dd, *J* = 7.4, 2.2 Hz, 2H), 7.60 – 7.54 (m, 1H), 7.50 – 7.38 (m, 4H), 7.38 – 7.33 (m, 2H), 7.31 (dd, *J* = 7.5, 0.9 Hz, 2H), 7.29 – 7.24 (m, 2H), 7.24 – 7.19 (m, 3H), 7.13 (d, *J* = 8.0 Hz, 2H), 5.80 (dd, *J* = 3.1, 1.7 Hz, 1H), 5.50 (d, *J* = 1.4 Hz, 1H), 5.06 (t, *J* = 9.8 Hz, 1H), 4.72 (d, *J* = 12.1 Hz, 1H), 4.57 (d, *J* = 12.1 Hz, 1H), 4.47 (d, *J* = 7.2 Hz, 2H), 4.42 (dd, *J* = 9.8, 6.2 Hz, 1H), 4.26 (t, *J* = 7.1 Hz, 1H), 4.01 (dd, *J* = 9.7, 3.2 Hz, 1H), 2.33 (s, 3H), 1.32 (d, *J* = 6.2 Hz, 3H). ^{13}C NMR (101 MHz, $CDCl_3$) δ 165.81, 155.04, 143.54, 143.31, 141.47, 141.44, 138.31, 137.45, 133.51, 132.50, 130.11, 130.08, 129.75, 129.65, 128.62, 128.51, 128.06, 127.95, 127.34, 125.24, 125.22, 120.24, 86.57, 75.06, 71.60, 70.91, 70.16, 46.98, 21.28, 17.58. $[\alpha]_D^{20}$ -34.10 (c = 1.00 g/100mL, $CHCl_3$). IR (film) ν = 2928, 1754, 1723, 1452, 1249, 1104, 758, 743, 711 cm^{-1}. R_f = 0.40 (Hexane : Acetone 3:1). ESI-HRMS m/z 709.2239 [M+Na]$^+$ ($C_{42}H_{38}O_7SNa$ requires 709.2230).

^{1}H NMR of BB3b (400 MHz, $CDCl_3$)

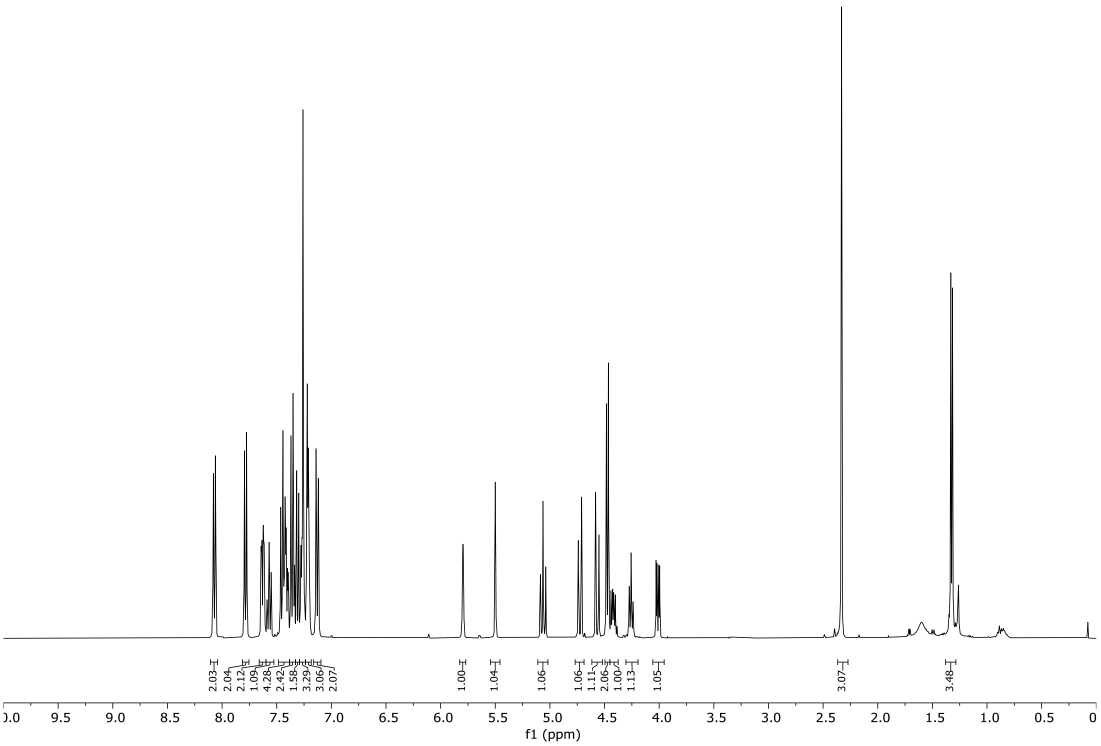

^{13}C NMR of BB3b (101 MHz, $CDCl_3$)

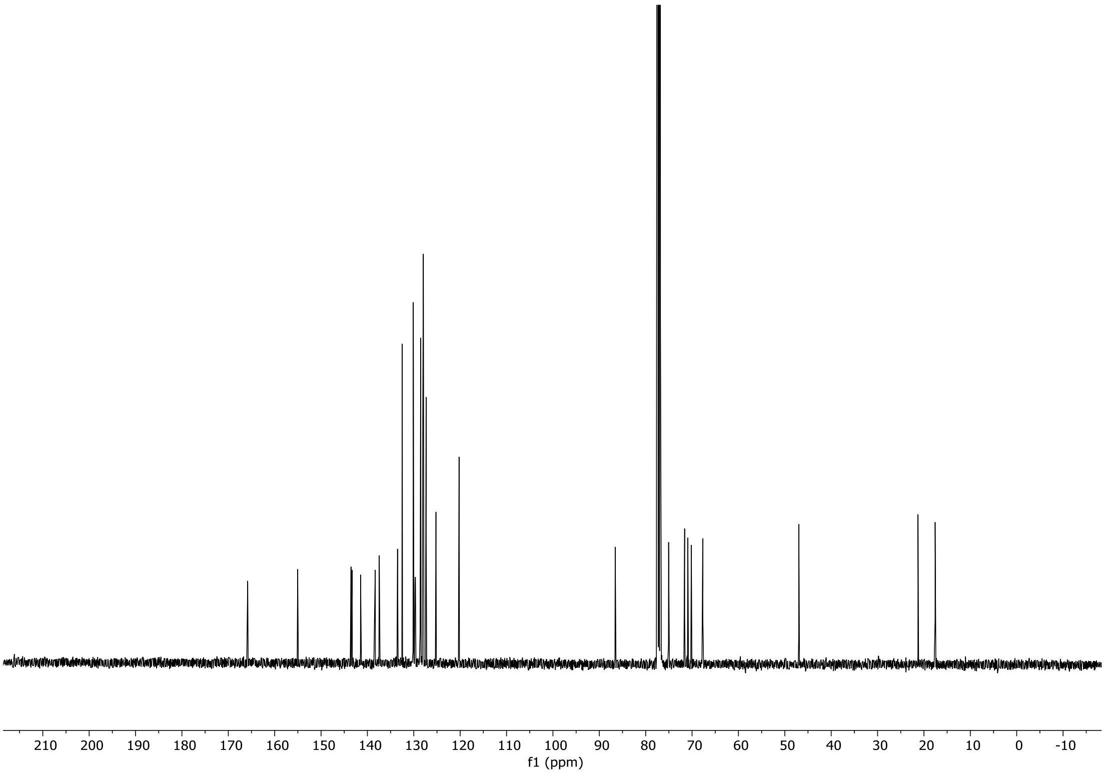

COSY NMR of BB3b ($CDCl_3$)

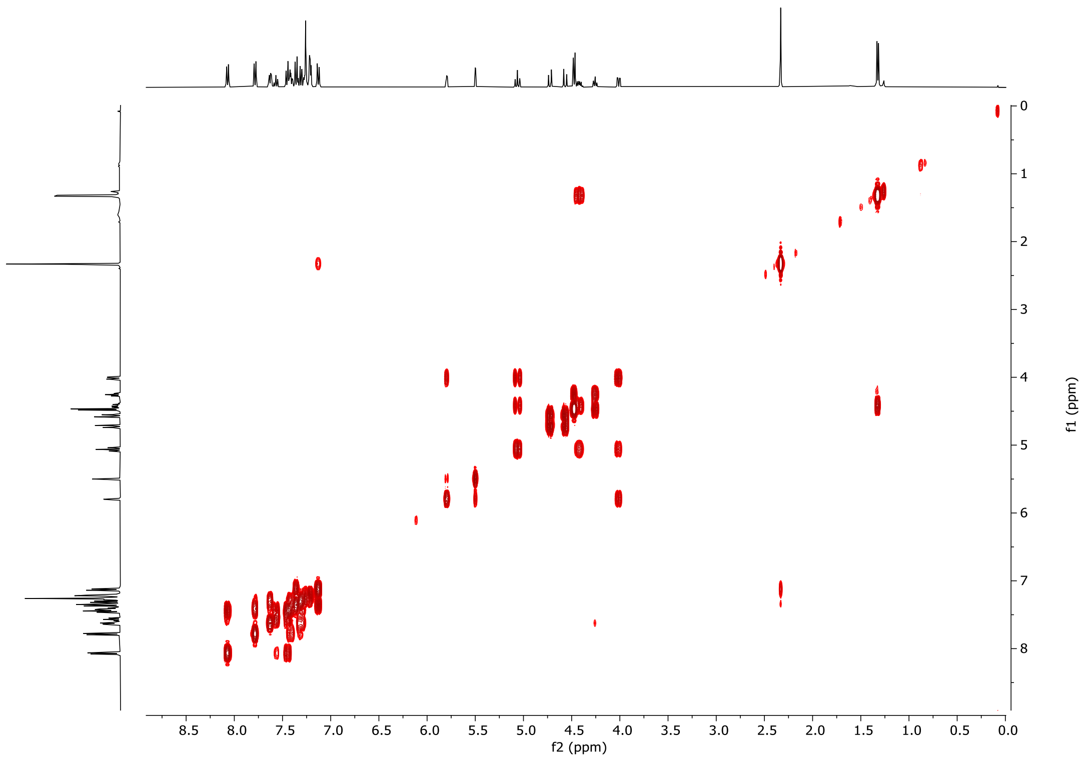

HSQC NMR of BB3b ($CDCl_3$)

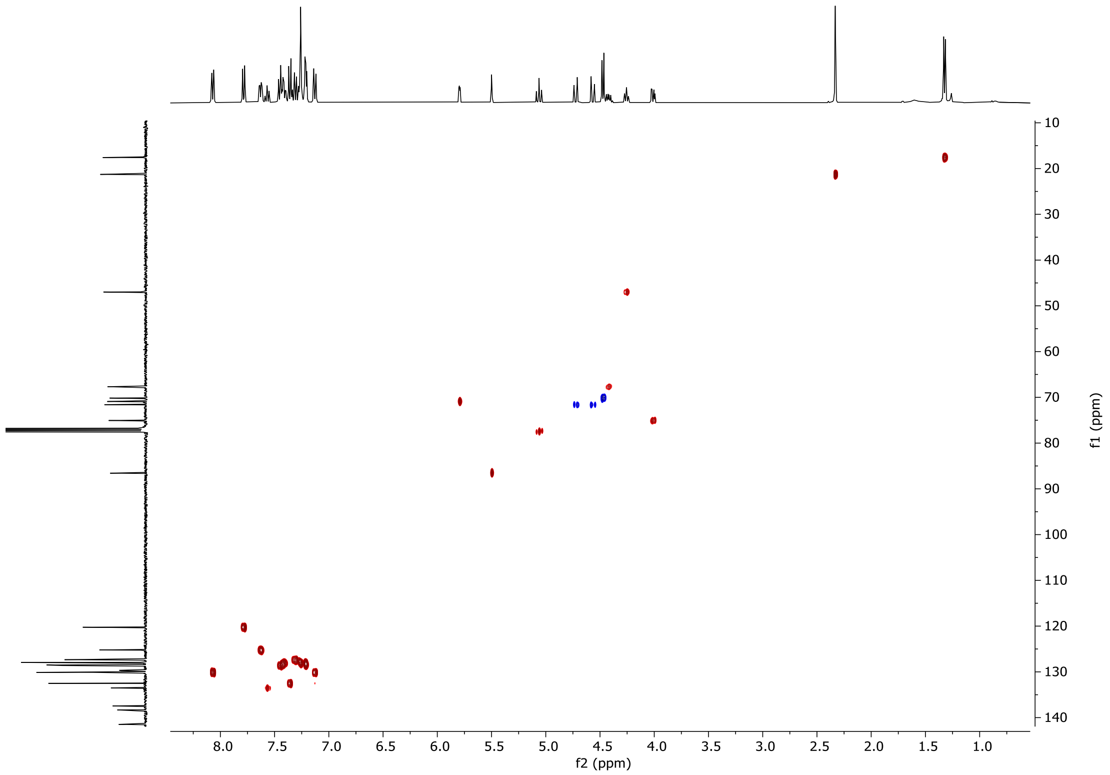

2.3 Synthesis of ^{13}C-BB2b

2.3.1 S-6

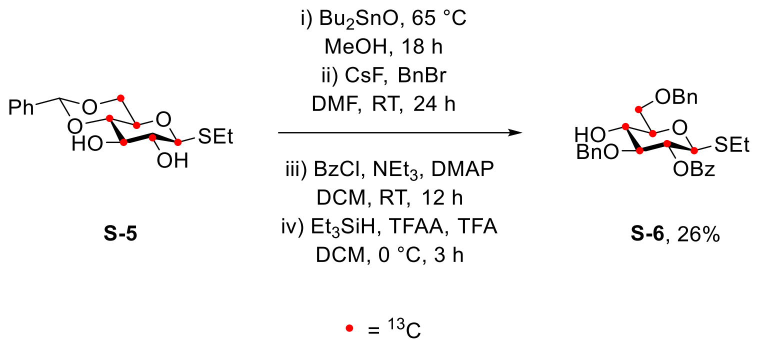

S-5 **S-6**, 26%

S-5 was synthesized according to previously reported procedure.[4]

S-5 (4.9 g, 18 mmol) was dissolved in MeOH (100 mL), di-*n*-butyltin oxide (5.5 g, 22 mmol) was added and the reaction mixture heated to 65 °C for 18 h. The reaction mixture was then cooled, concentrated *in vacuo* and the crude product was used in the next step without further purification. The crude product was dissolved in DMF (100 mL), benzyl bromide (3.76 g, 22 mmol) and cesium(I) fluoride (3.34 g, 22 mmol) were added and the mixture stirred at RT for 24 h under Ar atmosphere. The reaction mixture was concentrated *in vacuo* and the residue dissolved in DCM (120 mL). The organic layer was washed with aqueous potassium fluoride (100 mL, 1 M), dried over $MgSO_4$, filtered and concentrated *in vacuo*. The crude product was recrystallized from DCM : Hexane to give a white solid. The while solid was then dissolved in DCM (100 mL), benzoyl chloride (4.6 g, 33 mmol), DMAP (439 mg, 0.2 eq), and TEA (15 mL, 108 mmol) were added sequentially at 0 °C under Ar atmosphere. The reaction mixture was stirred at RT for 12 h, diluted with DCM and washed once with a saturated aqueous solution of $NaHCO_3$ and once with brine. The organic layer was dried over $MgSO_4$, filtered, and concentrated under reduced pressure. The crude product was recrystallized from DCM : Hexane to give a white solid. The white solid was dissolved in anhydrous DCM (50 mL) and triethylsilane (14.3 mL, 90 mmol) was added and the solution was cooled to 0 °C under Ar atmosphere. Trifluoroacetic acid (TFA) (6.9 mL, 90 mmol) and trifluoroacetic anhydride (TFAA) (1.2 mL, 9.0 mmol) were added sequentially. The solution was stirred at 0 °C for 3 h, after which time the reaction was diluted with DCM (50 mL) and washed once with a saturated aqueous solution of $NaHCO_3$ and once with brine. The organic layer was dried over $MgSO_4$, filtered, and concentrated under reduced pressure. The crude product was purified by silica gel flash column chromatography (Hexane : EtOAc, 3:1) to give **S-6** as a white solid (2.5 g, 26% over 4 steps).

^{1}H NMR (400 MHz, $CDCl_3$) δ 8.01 – 7.94 (m, 2H), 7.56 – 7.47 (m, 1H), 7.43 – 7.34 (m, 2H), 7.31 – 7.17 (m, 5H), 7.11 (s, 5H), 5.21 (dtt, *J* = 152.7, 9.5, 4.8 Hz, 1H), 4.49 (dm, *J* = 157.2 Hz, 1H), 3.72 (dm, *J* = 143.5 Hz, 3H), 3.63 (dm, *J* = 143.8 Hz, 1H), 3.51 (dm, *J* = 141.0 Hz, 1H), 2.73 (quintd, *J* = 7.4, 3.8 Hz, 2H), 1.25 (t, *J* = 7.5 Hz, 3H). ^{13}C NMR (101 MHz, $CDCl_3$) δ 165.33, 137.92, 137.90, 137.69, 137.66, 133.27, 129.90, 129.80, 128.52, 128.47, 128.44, 128.04, 127.91, 127.85, 127.82, 84.24 – 82.82 (m), 78.79 – 77.61 (m), 77.49 – 76.62 (m), 74.72, 73.80, 72.89 – 71.39 (m), 70.50 (dt, *J* = 44.7, 4.2 Hz), 24.00, 14.92; $[\alpha]_D^{20}$ -8.36 (c 1.00 g/100mL, $CHCl_3$). IR (film) ν = 1727, 1269, 1052 cm^{-1}. R_f = 0.39 (Hexane : EtOAc = 3:1). ESI-HRMS m/z 537.1986 $[M+Na]^+$ ($C_{23}{}^{13}C_6H_{32}O_6SNa$ requires 537.2013).

^{1}H NMR of S-6 (400 MHz, $CDCl_3$)

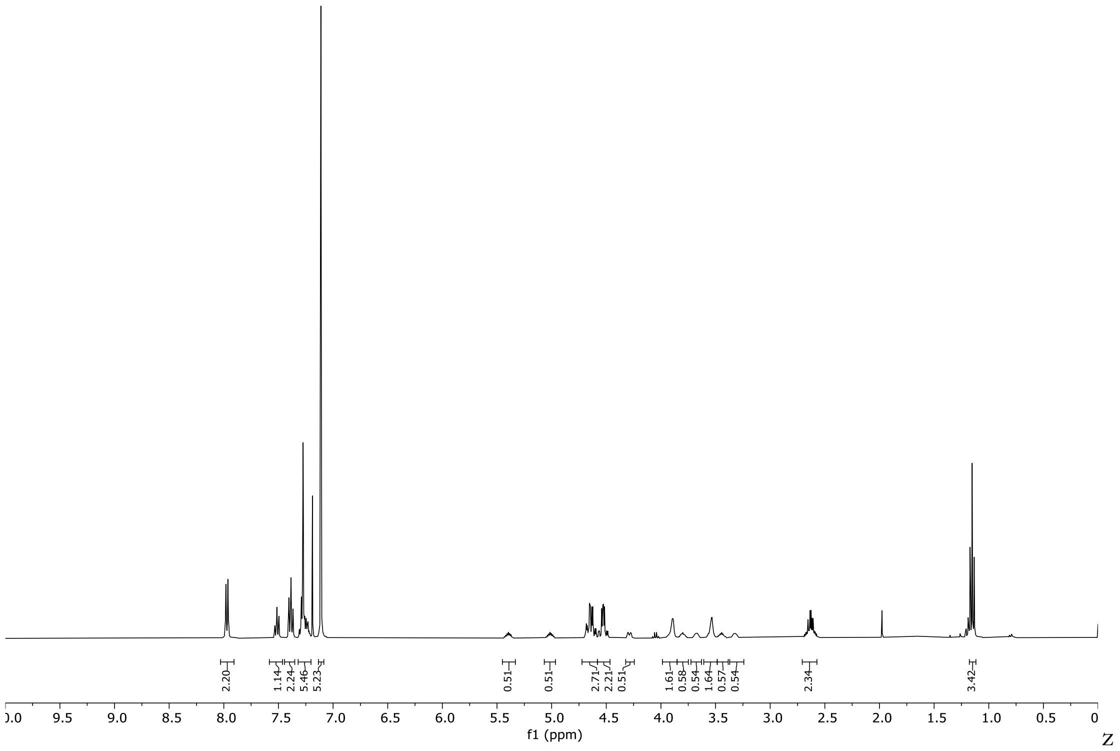

^{13}C NMR of S-6 (101 MHz, $CDCl_3$)

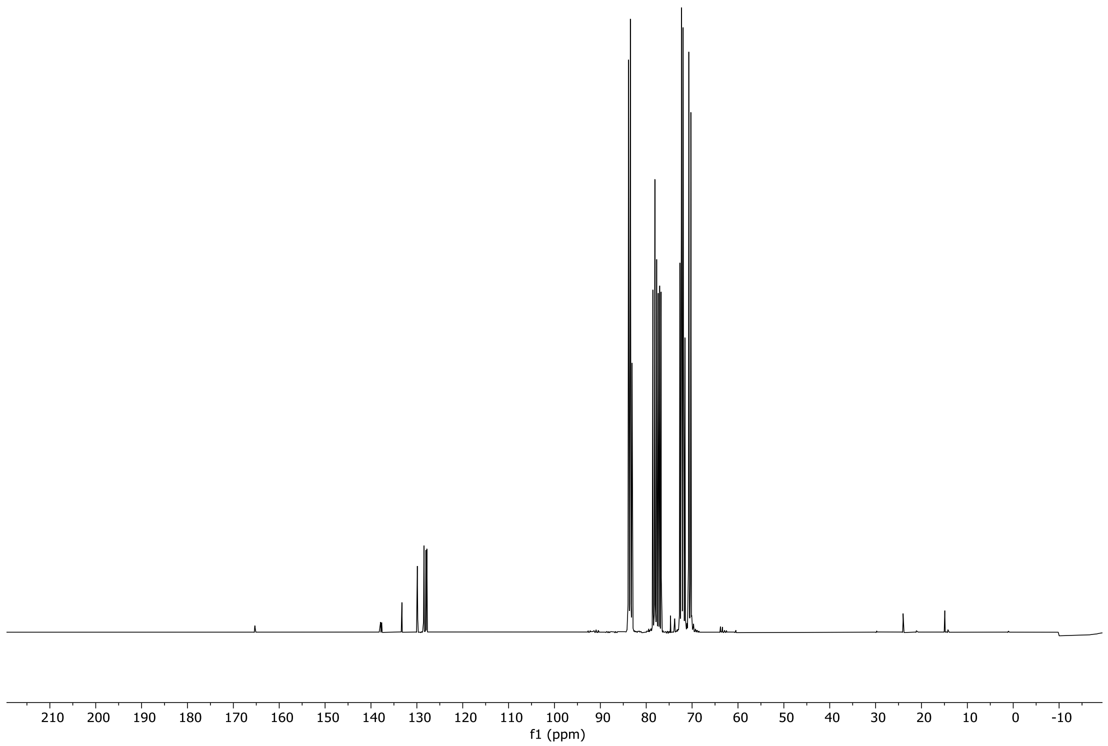

HSQC NMR of S-6 ($CDCl_3$)

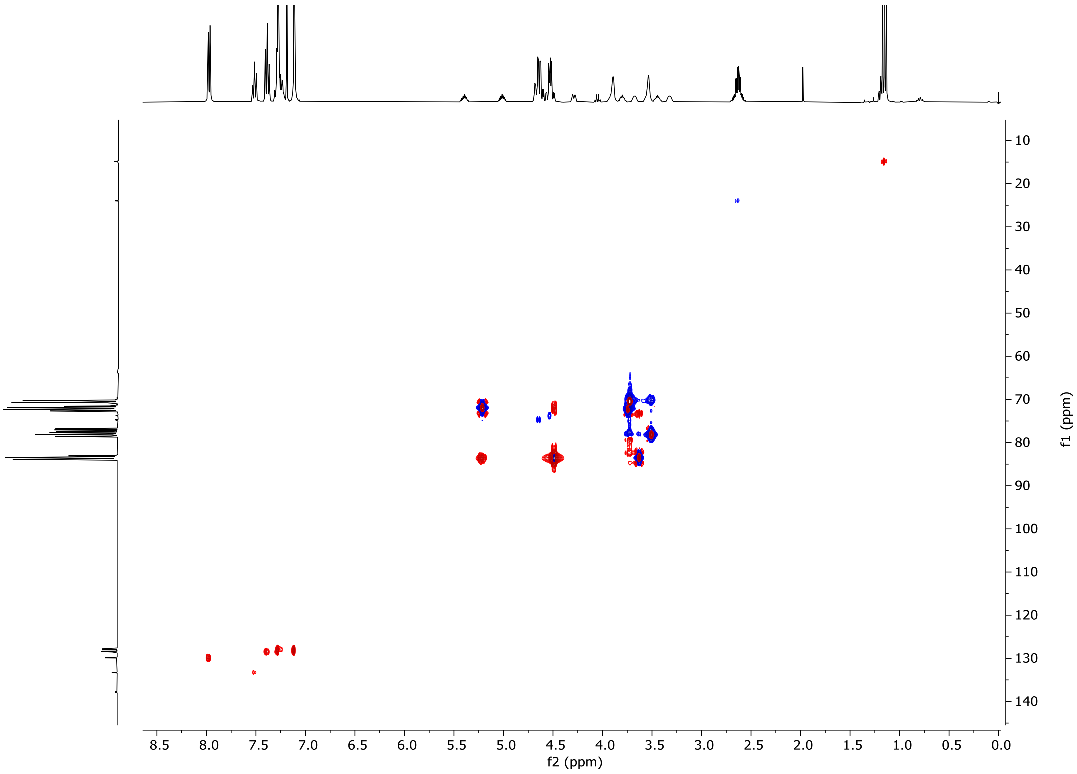

2.3.2 S-7

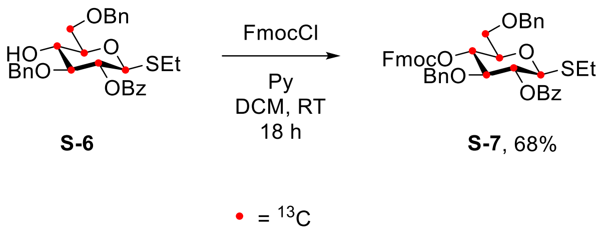

S-6 (2.5 g, 4.9 mmol) was dissolved in DCM (50 mL) under Ar atmosphere. Pyridine (1.2 mL, 15 mmol) and FmocCl (2.5 g, 9.8 mmol) were added to the stirred reaction mixture. The yellow solution was stirred for 3 h and then quenched with an aqueous solution of HCl (1 M). The organic layer was washed once with an aqueous solution of HCl (1 M), once with a saturated aqueous solution of $NaHCO_3$, and once with brine. The crude product was purified with silica gel flash column chromatography (Hexane : EtOAc, 5:1) to give compound **S-7** as white solid (2.9 g, 78%).

^{1}H NMR (400 MHz, $CDCl_3$) δ 7.98 – 7.91 (m, 2H), 7.68 (ddt, *J* = 7.5, 4.3, 0.9 Hz, 2H), 7.51 (d, *J* = 7.4 Hz, 2H), 7.46 (dt, *J* = 7.4, 1.0 Hz, 1H), 7.38 (t, *J* = 7.8 Hz, 2H), 7.34 – 7.29 (m, 2H), 7.27 – 7.14 (m, 7H), 7.03 – 6.91 (m, 5H), 5.26 (dm, *J* = 153.0 Hz, 1H), 4.92 (dm, *J* = 153.2 Hz, 1H), 4.49 (dm, *J* = 151.9 Hz, 1H), 4.25 (d, *J* = 6.8 Hz, 2H), 4.05 (t, *J* = 7.1 Hz, 1H), 3.83 (dm, *J* = 142.7 Hz, 1H), 3.67 (dm, *J* = 144.7 Hz, 1H), 3.60 (dm, *J* = 142.6 Hz, 2H), 2.65 (td, *J* = 7.6, 3.9 Hz, 2H), 1.16 (t, *J* = 7.4 Hz, 3H). ^{13}C NMR (101 MHz, cdcl$_3$) δ 165.04, 143.28, 143.10, 141.30, 141.27, 137.36, 133.27, 129.89, 129.64, 128.42, 128.33, 128.15, 127.91, 127.83, 127.65, 127.63, 127.17, 125.10, 125.01, 120.08, 83.67 (dd, *J* = 42.1, 4.0 Hz), 81.07 (t, *J* = 40.3 Hz), 78.08 – 76.48 (m), 75.50 (td, *J* = 40.8, 4.5 Hz), 74.38, 73.60, 71.84 (td, *J* = 41.5, 40.7, 4.6 Hz), 70.02, 69.67 (dt, *J* = 44.7, 3.9 Hz), 46.68, 24.06, 14.89. $[\alpha]_D^{20}$ +35.87 (c 1.00 g/100mL, $CHCl_3$); IR (film) ν = 1754, 1452, 1253 cm^{-1}. R_f = 0.25 (Hexane : Acetone = 3:1). ESI-HRMS m/z 759.2678 $[M+Na]^+$ ($C_{38}{}^{13}C_6H_{42}O_8SNa$ requires 759.2694).

^{1}H NMR of S-7 (400 MHz, $CDCl_3$)

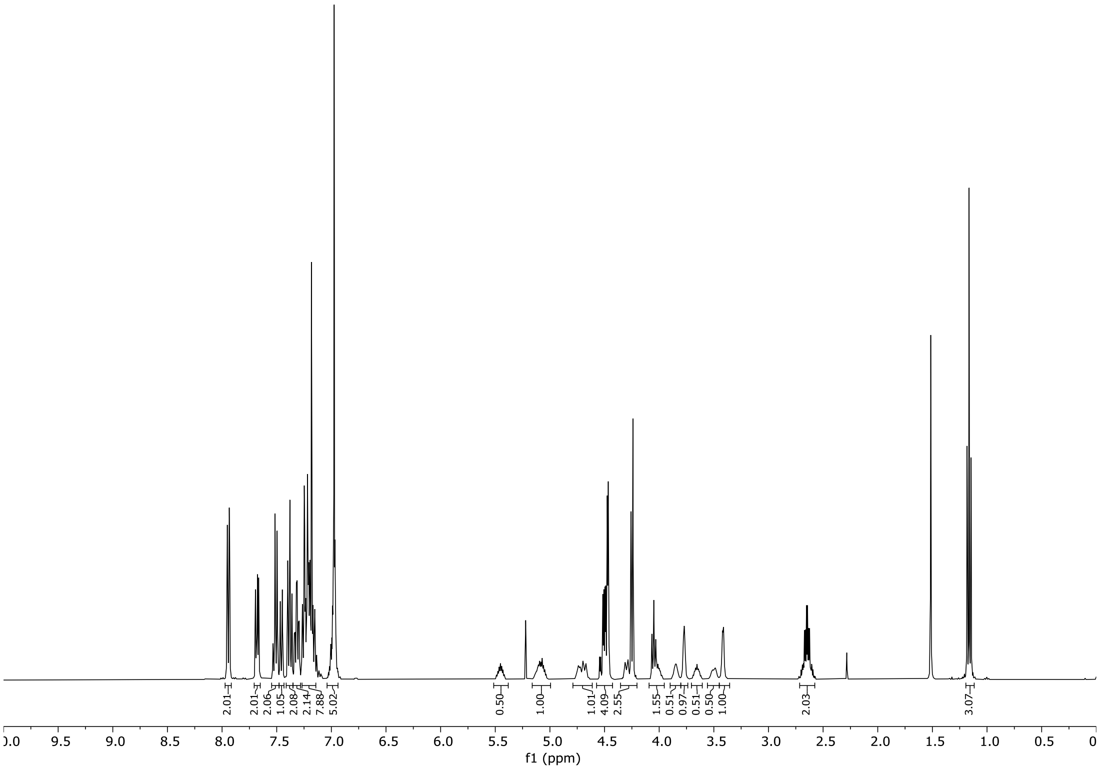

^{13}C NMR of S-7 (101 MHz, $CDCl_3$)

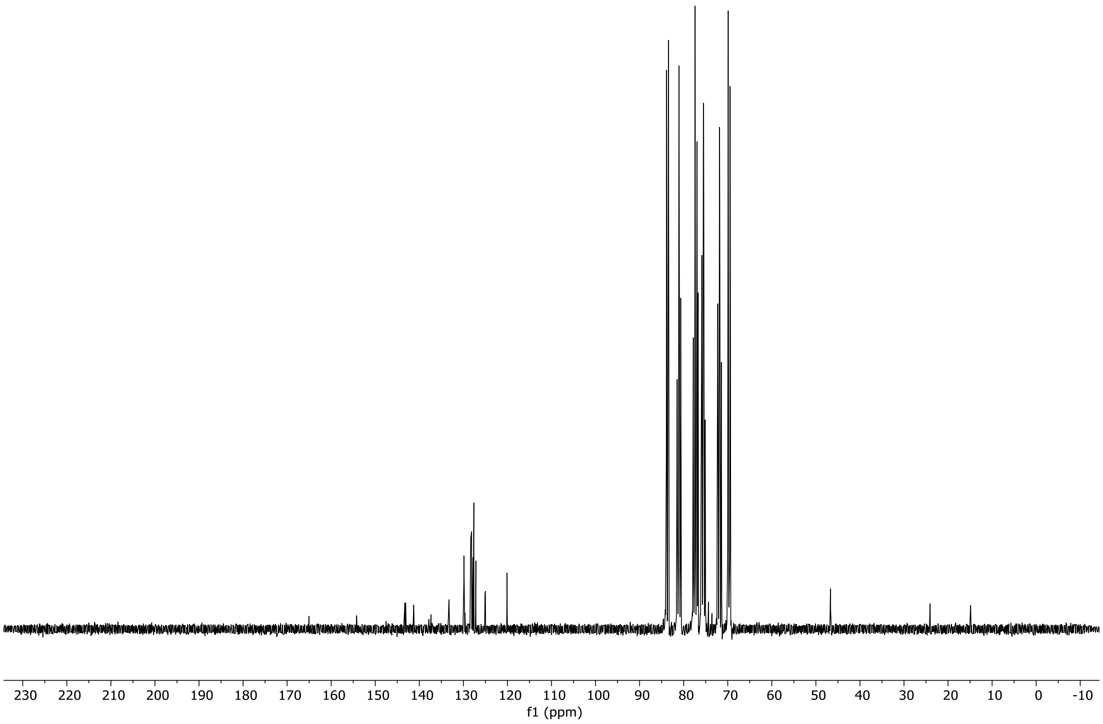

HSQC NMR of S-7 ($CDCl_3$)

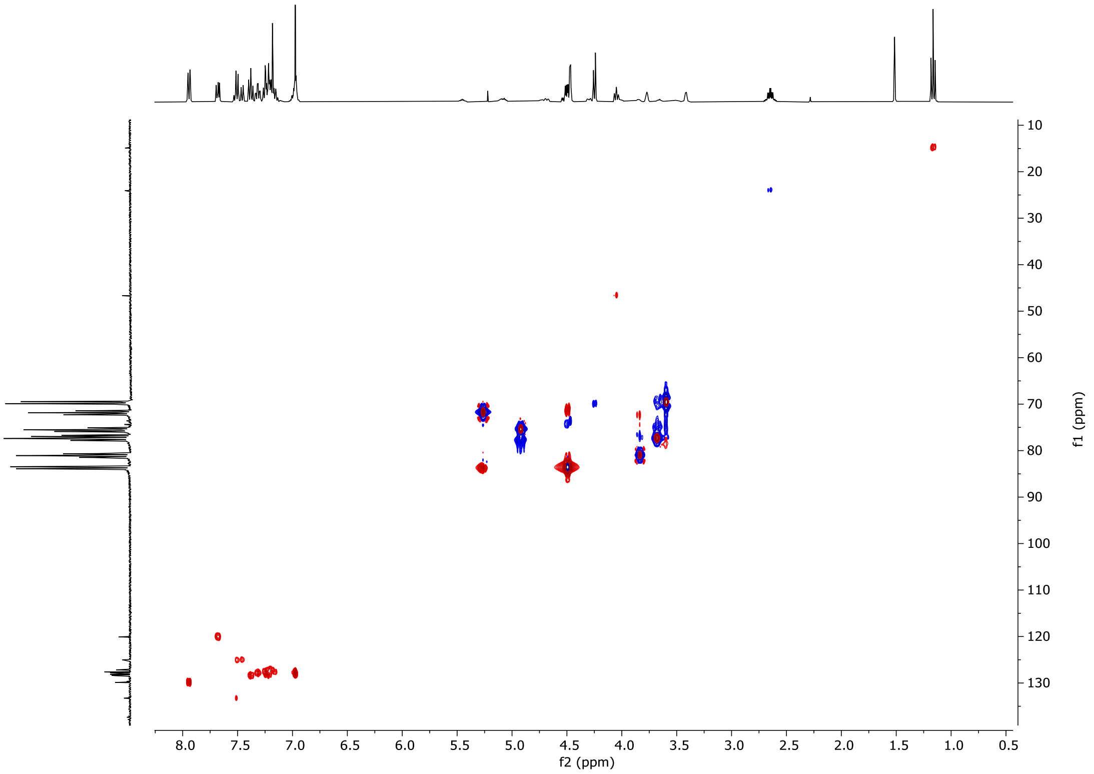

2.3.3 ^{13}C-BB2b

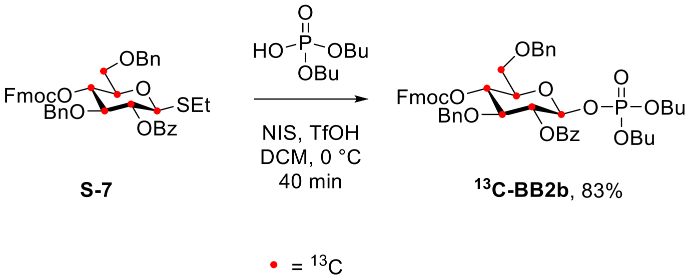

S-7 (338 mg, 0.46 mmol) was co-evaporated three times with toluene, dissolved in anhydrous DCM (1.6 mL) and stirred in the presence of molecular sieves (4 Å, powder, flame dried) for 1 h under Ar atmoshpere. Di-*n*-butyl phosphate (375 µL) was dissolved in anhydrous DCM (2 mL) and stirred in the presence of flame dried 4 Å molecular sieves for 1 h (Solution A). Solution A (1.1 mL, 1.0 mmol of di-*n*-butyl phosphate): was added to the reaction mixture and cooled to 0 °C. NIS (124 mg, 0.55 mmol) was added in one portion followed by a dropwise addition of TfOH (3 µL, 0.03 mmol) and the reaction was stirred for 40 min at 0 °C, after which time it was quenched by addition of Py (1 drop). The reaction was diluted with DCM, filtered over celite, washed once with a saturated aqueous solution of $Na_2S_2O_3$ and once with brine. The organic layer was dried over Na_2SO_4 and concentrated under reduced pressure. The crude product was purified by silica gel flash column chromatography (Hexane : Acetone, 5:1→4:1→3:1→2:1) to yield **^{13}C-BB2b** as a sticky colorless solid (338 mg, 83%).

^{1}H NMR (400 MHz, $CDCl_3$) δ 8.01 (d, *J* = 7.3 Hz, 2H), 7.81 – 7.71 (m, 2H), 7.64 – 7.50 (m, 3H), 7.49 – 7.35 (m, 4H), 7.34 – 7.18 (m, 8H), 7.16 – 6.98 (m, 4H), 5.66 – 5.53 (m, 1H), 5.34 – 5.23 (m, 0.5H), 5.23 – 5.11 (m, 1H), 4.99 – 4.82 (m, 0.5H), 4.64 – 4.45 (m, 4H), 4.42 – 4.27 (m, 2H), 4.19 – 3.93 (m, 4H), 3.91 – 3.80 (m, 1H), 3.79 – 3.58 (m, 3H), 3.55 – 3.43 (m, 1H), 1.56 (dt, *J* = 14.6, 6.7 Hz, 2H), 1.39 – 1.21 (m, 4H), 1.06 – 0.93 (m, 2H), 0.86 (t, *J* = 7.4 Hz, 3H), 0.67 (t, *J* = 7.4 Hz, 3H). ^{13}C NMR (101 MHz, $CDCl_3$) δ 143.40, 143.20, 141.42, 133.58, 130.06, 129.40, 128.60, 128.47, 128.34, 128.11, 128.07, 127.87, 127.81, 127.75, 127.33, 125.22, 125.14, 120.23, 96.91, 96.86, 96.43, 96.39, 92.36, 91.90, 90.77, 90.31, 88.40, 87.97, 79.93, 79.64, 79.24, 78.84, 77.48, 77.16, 76.84, 75.56, 75.52, 75.15, 75.11, 74.75, 74.71, 74.14, 73.73, 73.31, 72.96, 72.92, 72.52, 71.89, 71.74, 71.46, 71.28, 71.05, 70.90, 70.23, 69.38, 68.94, 68.55, 46.82, 32.16, 32.08, 31.91, 31.84, 18.78, 18.68, 18.35, 13.69, 13.50. ^{31}P NMR (162 MHz, $CDCl_3$) δ -2.75. $[\alpha]_D^{20}$ 56.57 (c = 1.00 g/100mL, $CHCl_3$). IR (film) ν = 2962, 1756, 1734, 1453, 1250, 1070, 1028, 740 cm^{-1}. R_f = 0.46 (Hexane : Acetone 2:1). ESI-HRMS m/z 907.3526 $[M+Na]^+$ ($C_{44}{}^{13}C_6H_{55}O_{12}PNa$ requires 907.3524).

^{1}H NMR of ^{13}C-BB2b (400 MHz, $CDCl_3$)

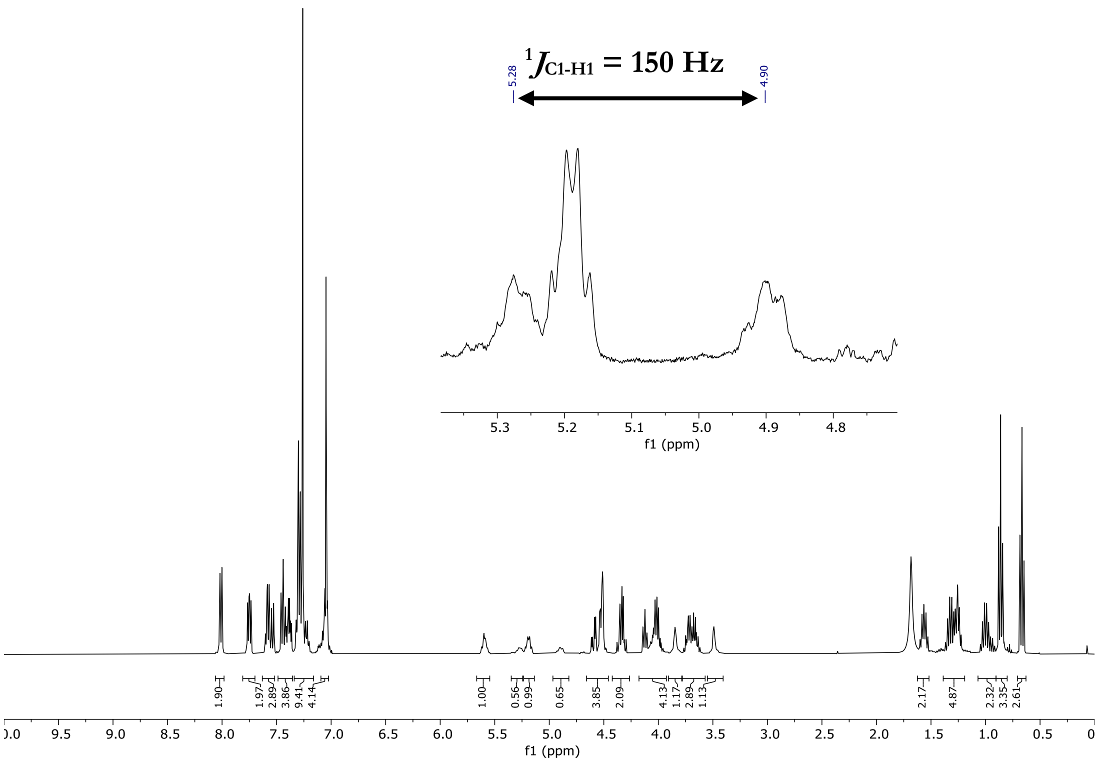

^{13}C NMR of ^{13}C-BB2b (101 MHz, $CDCl_3$)

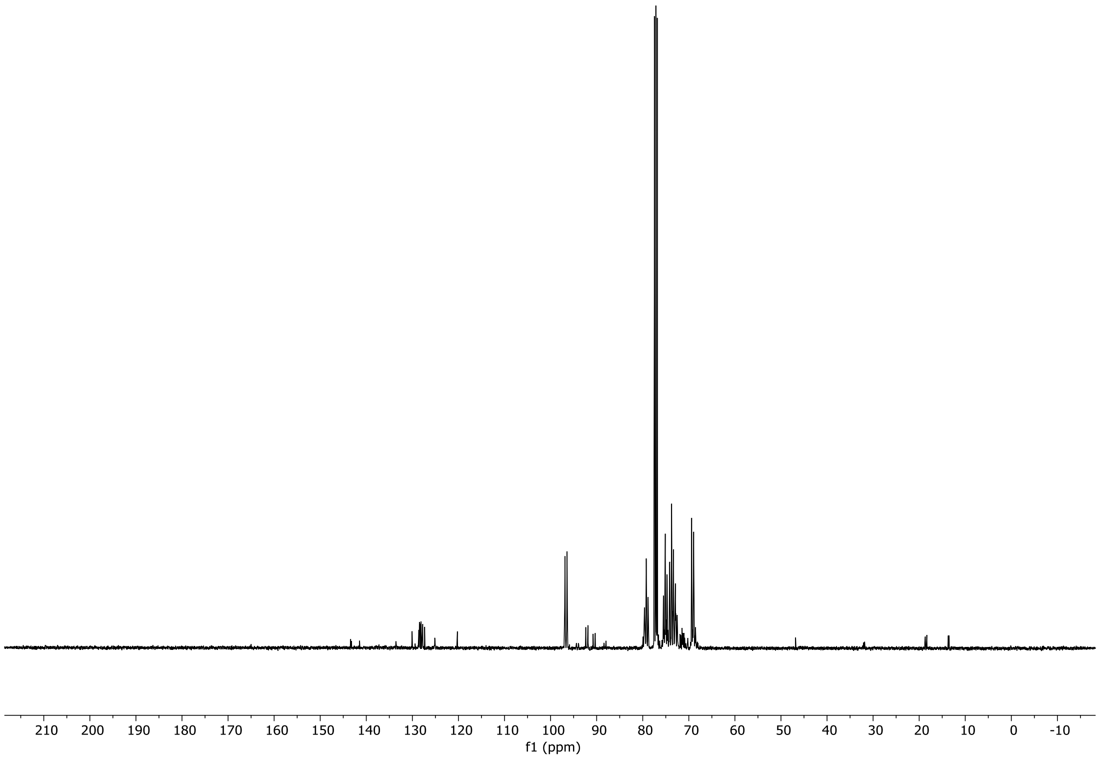

^{31}P NMR of ^{13}C-BB2b (162 MHz, CDCl$_3$)

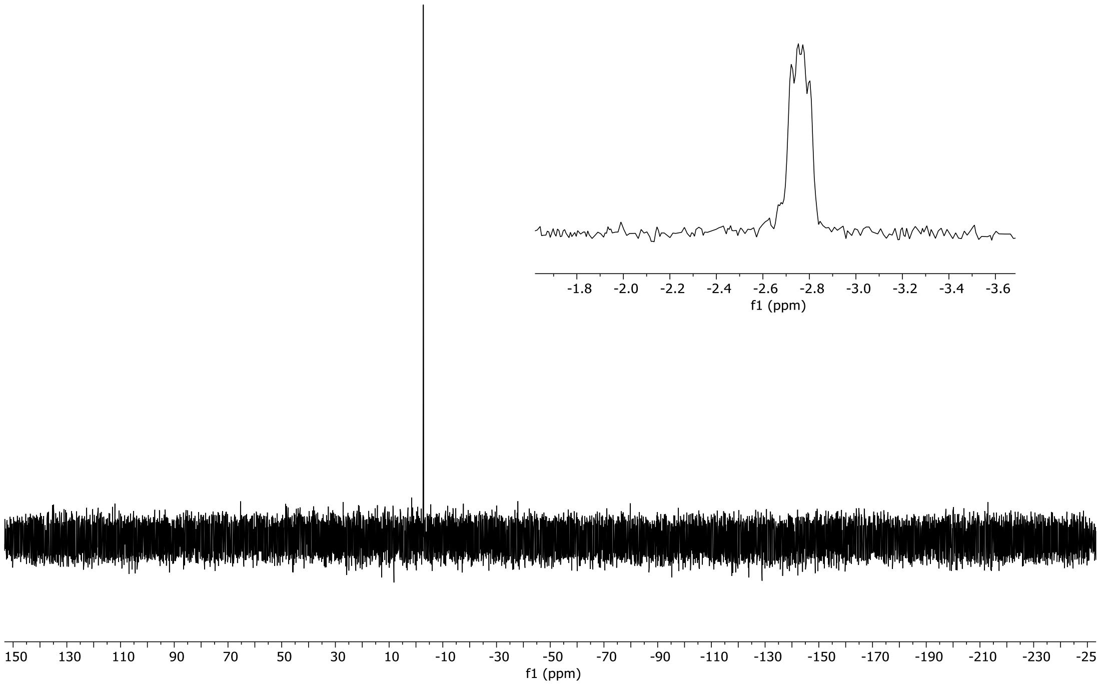

HSQC NMR of ^{13}C-BB2b (CDCl$_3$)

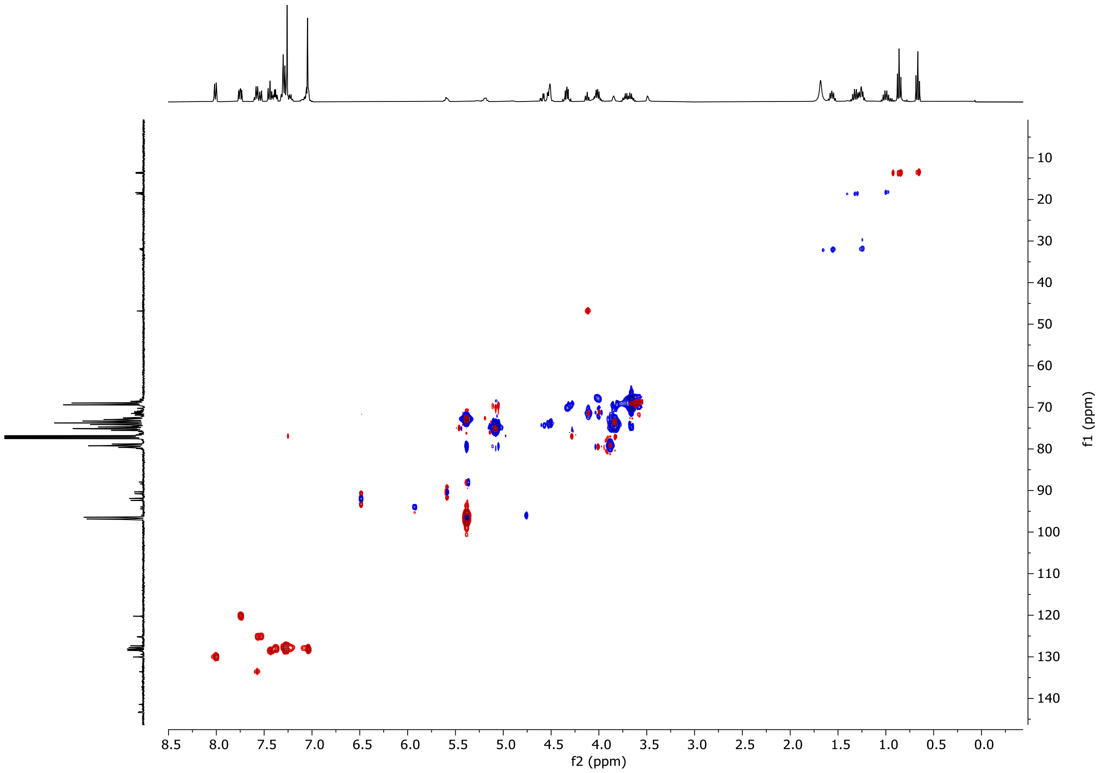

Comparison of ¹H NMR of BB2b (top) and ¹³C-BB2b (bottom) ($CDCl_3$)

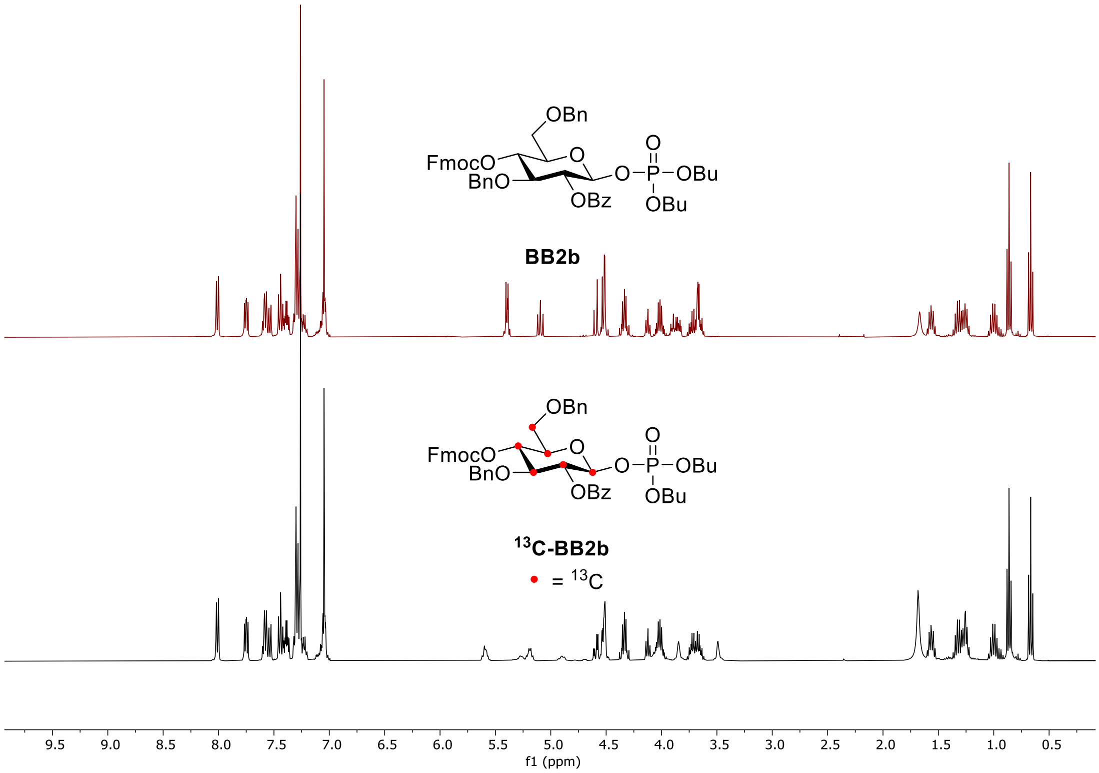

2.4 Synthesis of BB4

2.4.1 S-9

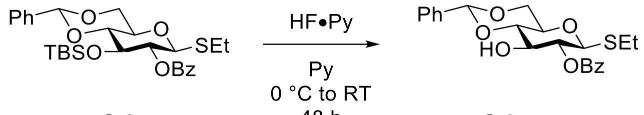

S-8 → **S-9**, 78%

S-8 was synthesized according to previously reported procedure.[5]

S-8 (514 mg, 0.97 mmol) was dissolved in pyridine (Py, 7 mL) and cooled to 0 °C. HF•Py (1.25 mL, 70%$_w$ HF) was added dropwise to the stirred reaction mixture. After 1 h, the reaction was allowed to RT and stirred for 48 h, after which time the reaction was cooled to 0 °C and quenched with a saturated aqueous solution of $NaHCO_3$. The crude product was diluted with DCM and washed twice with saturated aqueous solution of $NaHCO_3$. The organic layer was dried over Na_2SO_4 and concentrated under reduced pressure. The crude product was purified by silica gel flash column chromatography (Hexane : EtOAc, 4:1→3:1→2:1) to yield **S-9** as a colorless foam (320 mg, 78%).

^{1}H NMR (400 MHz, $CDCl_3$) δ 8.09 (d, *J* = 7.8 Hz, 2H), 7.59 (t, *J* = 7.4 Hz, 1H), 7.53 – 7.43 (m, 4H), 7.38 (d, *J* = 4.9 Hz, 3H), 5.58 (s, 1H), 5.25 (t, *J* = 9.4 Hz, 1H), 4.70 (d, *J* = 10.0 Hz, 1H), 4.41 (dd, *J* = 10.5, 4.8 Hz, 1H), 4.10 (q, *J* = 8.3 Hz, 1H), 3.82 (t, *J* = 10.2 Hz, 1H), 3.69 (t, *J* = 9.3 Hz, 1H), 3.58 (td, *J* = 9.6, 4.9 Hz, 1H), 2.74 (quint, *J* = 7.1 Hz, 2H), 2.67 (s, 1H), 1.25 (t, *J* = 7.4 Hz, 3H). ^{13}C NMR (101 MHz, $CDCl_3$) δ 165.96, 136.96, 133.54, 130.15, 129.66, 129.52, 128.59, 128.53, 126.42, 102.11, 84.18, 80.97, 73.74, 73.14, 70.67, 68.72, 24.28, 15.00. $[\alpha]_D^{20}$ -24.01 (c = 0.80 g/100mL, $CHCl_3$). IR (film) ν = 3475, 2927, 1727, 1270, 1095, 711 cm^{-1}. R_f = 0.24 (Hexane : EtOAc = 4:1). ESI-HRMS m/z 439.1208 $[M+Na]^+$ ($C_{22}H_{24}O_6SNa$ requires 439.1186).

^{1}H NMR of S-9 (400 MHz, $CDCl_3$)

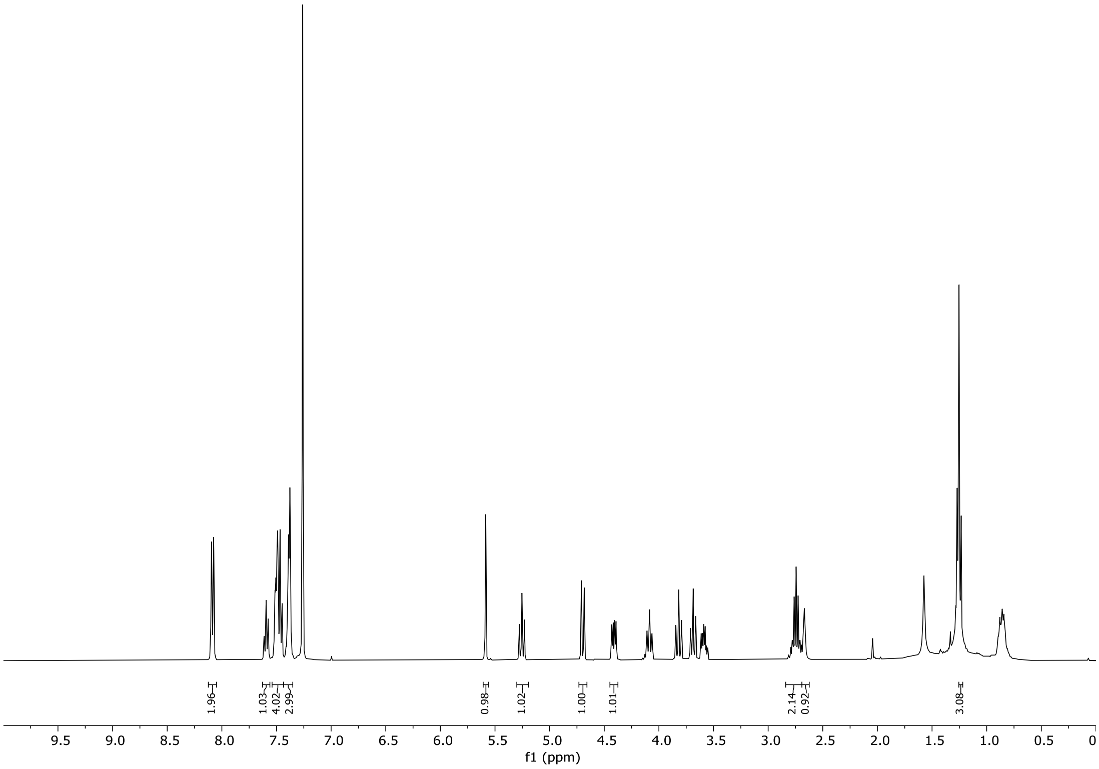

^{13}C NMR of S-9 (101 MHz, $CDCl_3$)

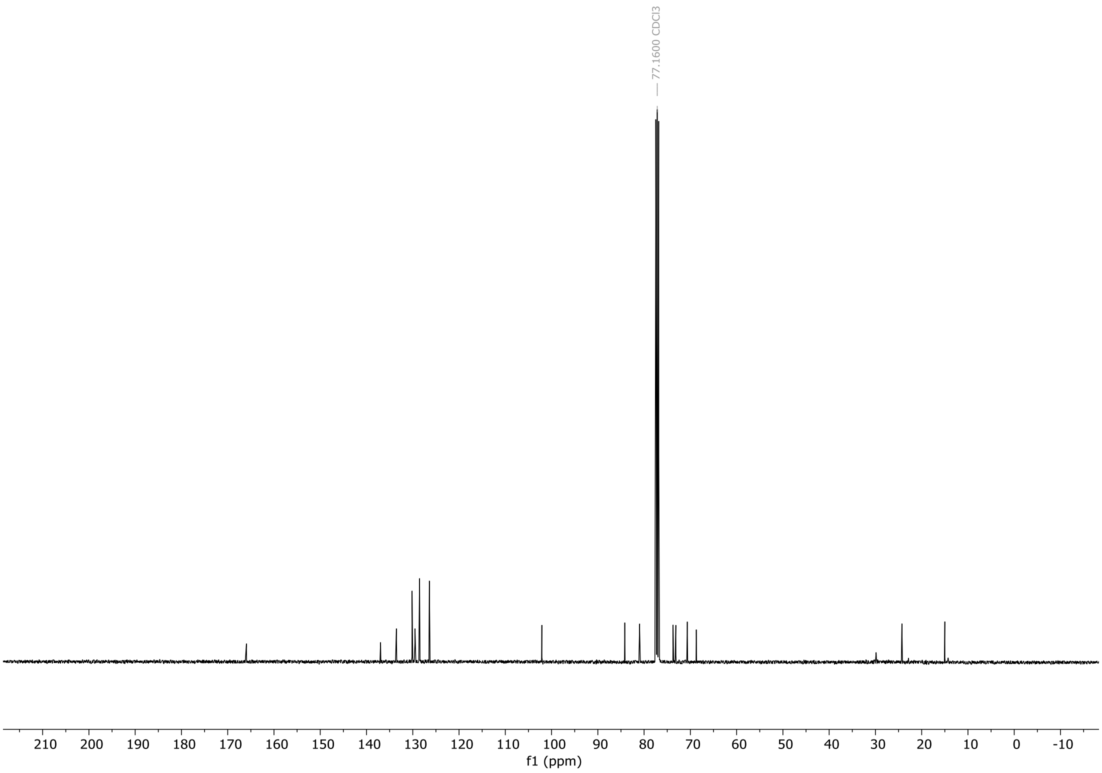

COSY NMR of S-9 ($CDCl_3$)

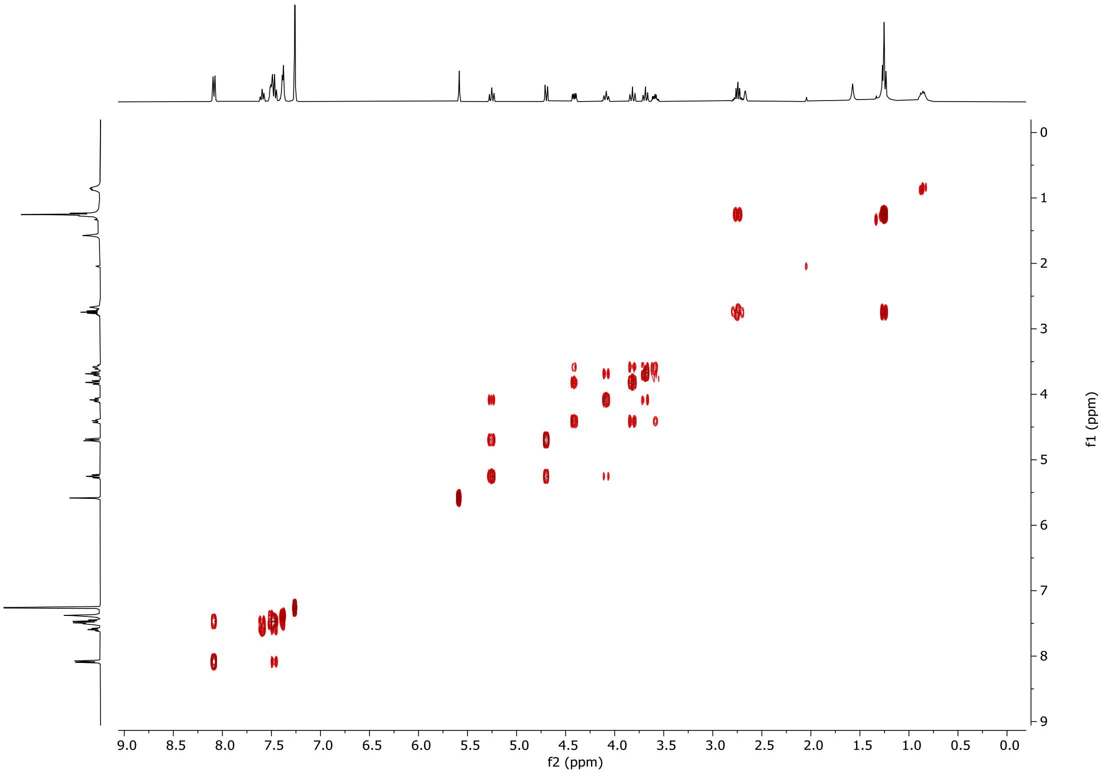

HSQC NMR of S-9 ($CDCl_3$)

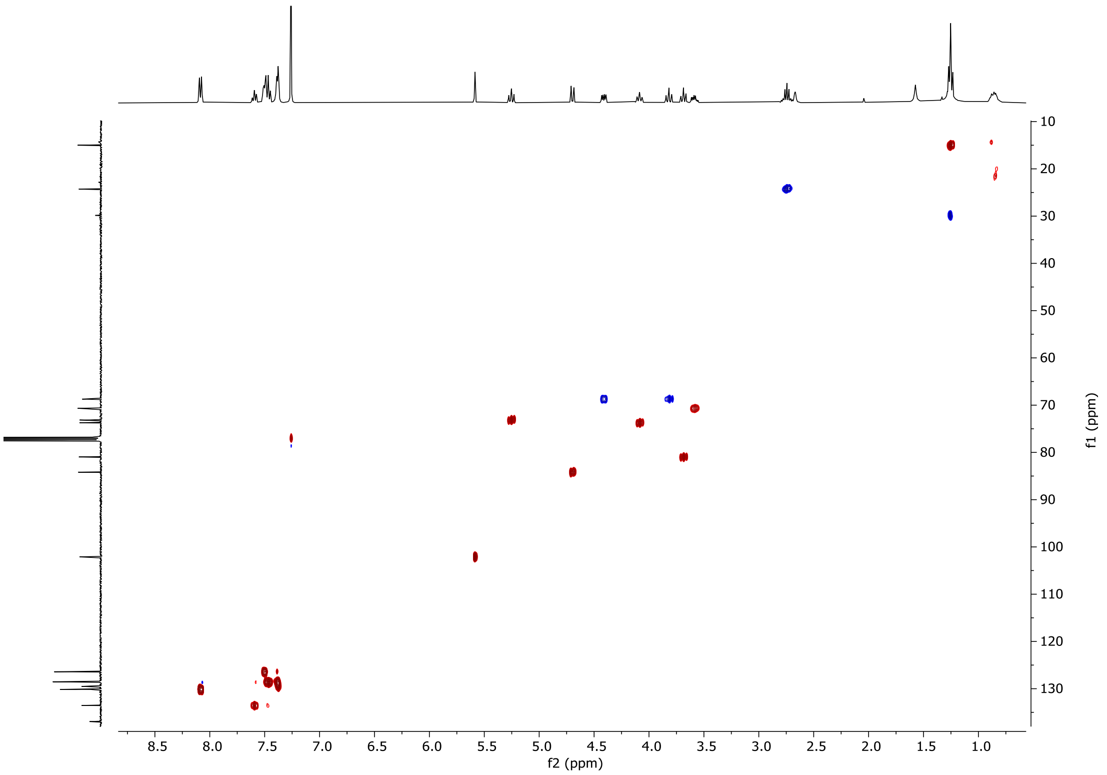

2.4.2 S-10

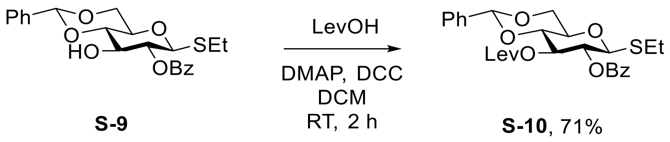

S-9 (311 mg, 0.75 mmol) was dissolved in anhydrous DCM (4 mL) under Ar atmosphere. Levulinic acid (LevOH, 100 μL, 1.5 mmol), *N,N*'-dicyclohexylcarbodiimide (DCC, 280 mg, 1.35 mmol), and 4-dimethylaminopyridine (DMAP, 20 mg, 0.15 mmol) were added to the stirred reaction mixture at RT. After 2 h, the reaction was passed through a short plug of silica gel using DCM as eluent. The crude reaction mixture was concentrated under vacuum and recrystallized from DCM : Hexane to yield **S-10** as a colorless crystalline solid (275 mg, 71%).

^{1}H NMR (400 MHz, $CDCl_3$) δ 8.02 (d, *J* = 7.9 Hz, 2H), 7.58 (t, *J* = 7.3 Hz, 1H), 7.49 – 7.42 (m, 4H), 7.41 – 7.33 (m, 3H), 5.57 – 5.48 (m, 2H), 5.33 (t, *J* = 9.6 Hz, 1H), 4.73 (d, *J* = 10.0 Hz, 1H), 4.41 (dd, *J* = 10.5, 4.8 Hz, 1H), 3.88 – 3.73 (m, 2H), 3.65 (td, *J* = 9.6, 4.9 Hz, 1H), 2.74 (qt, *J* = 7.3, 4.0 Hz, 2H), 2.60 – 2.39 (m, 4H), 1.97 (s, 3H), 1.24 (t, *J* = 7.4 Hz, 3H). ^{13}C NMR (101 MHz, $CDCl_3$) δ 205.81, 171.93, 165.42, 136.88, 133.51, 130.10, 129.39, 129.22, 128.57, 128.36, 126.27, 101.56, 84.51, 78.57, 72.97, 71.10, 71.04, 68.64, 38.05, 29.62, 28.13, 24.51, 14.94. $[\alpha]_D^{20}$ -23.81 (c = 0.70 g/100mL, $CHCl_3$). IR (film) ν = 2930, 1724, 1269, 1097, 712 cm^{-1}. R_f = 0.42 (Hexane : EtOAc = 2:1). ESI-HRMS m/z 537.1572 [M+Na]$^+$ ($C_{27}H_{30}O_8SNa$ requires 537.1553).

^{1}H NMR of S-10 (400 MHz, $CDCl_3$)

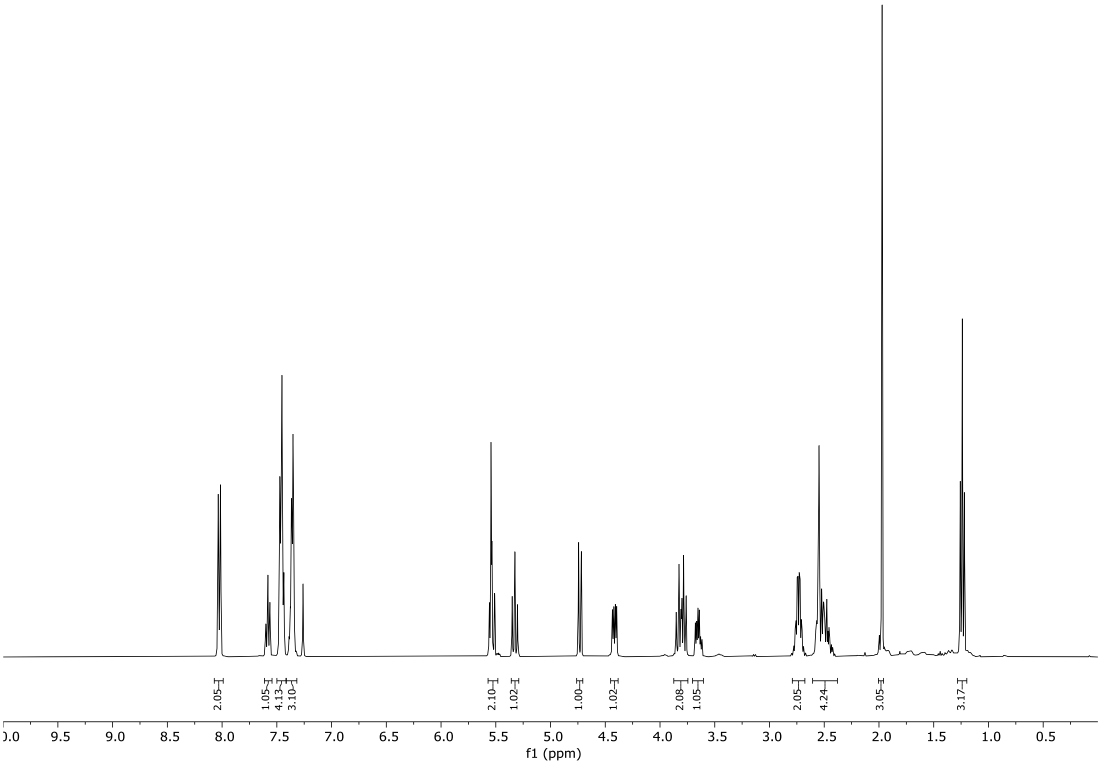

^{13}C NMR of S-10 (101 MHz, $CDCl_3$)

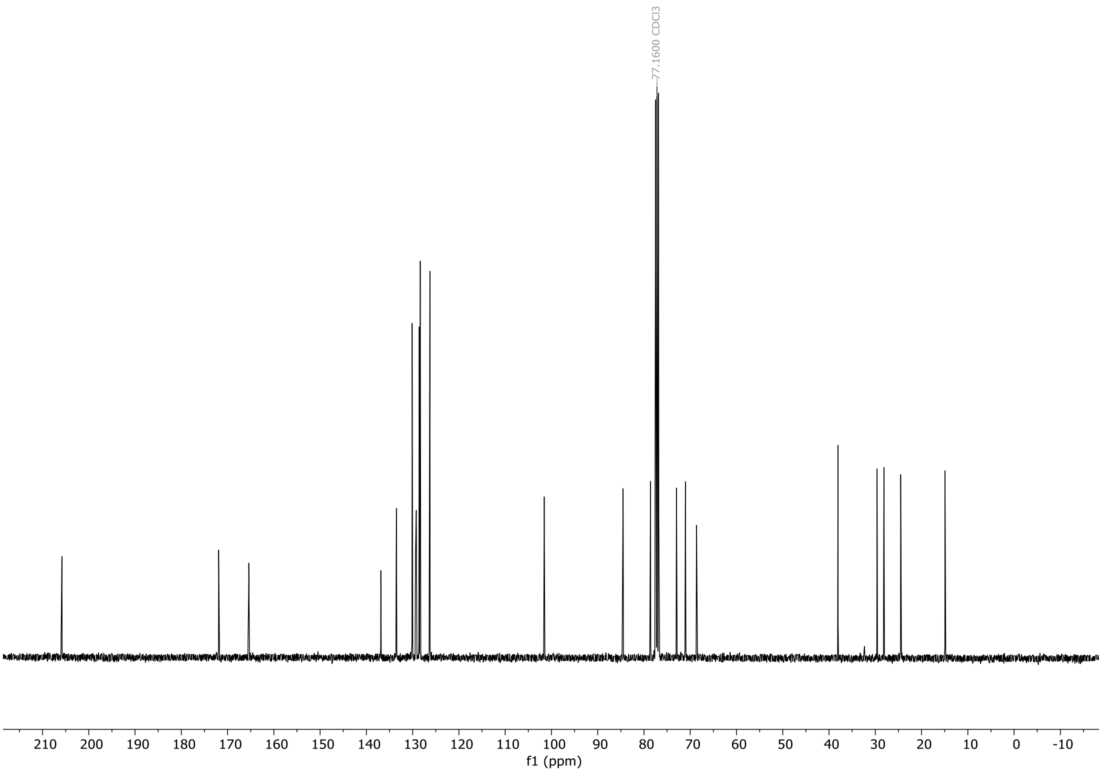

COSY NMR of S-10 ($CDCl_3$)

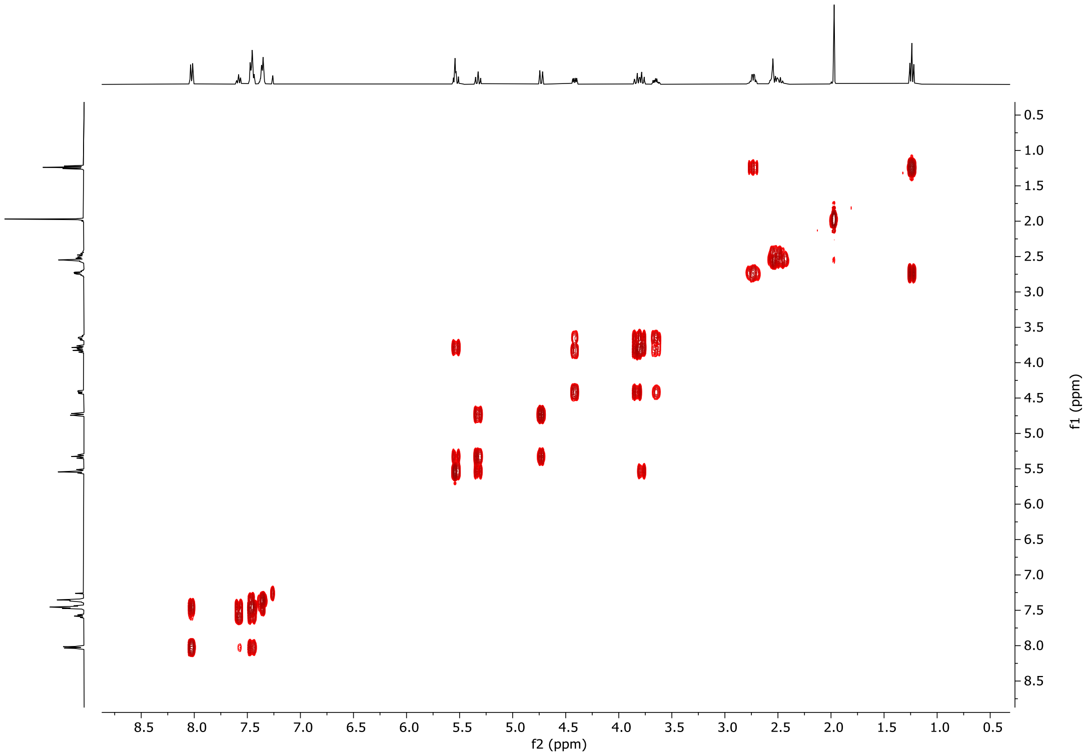

HSQC NMR of S-10 ($CDCl_3$)

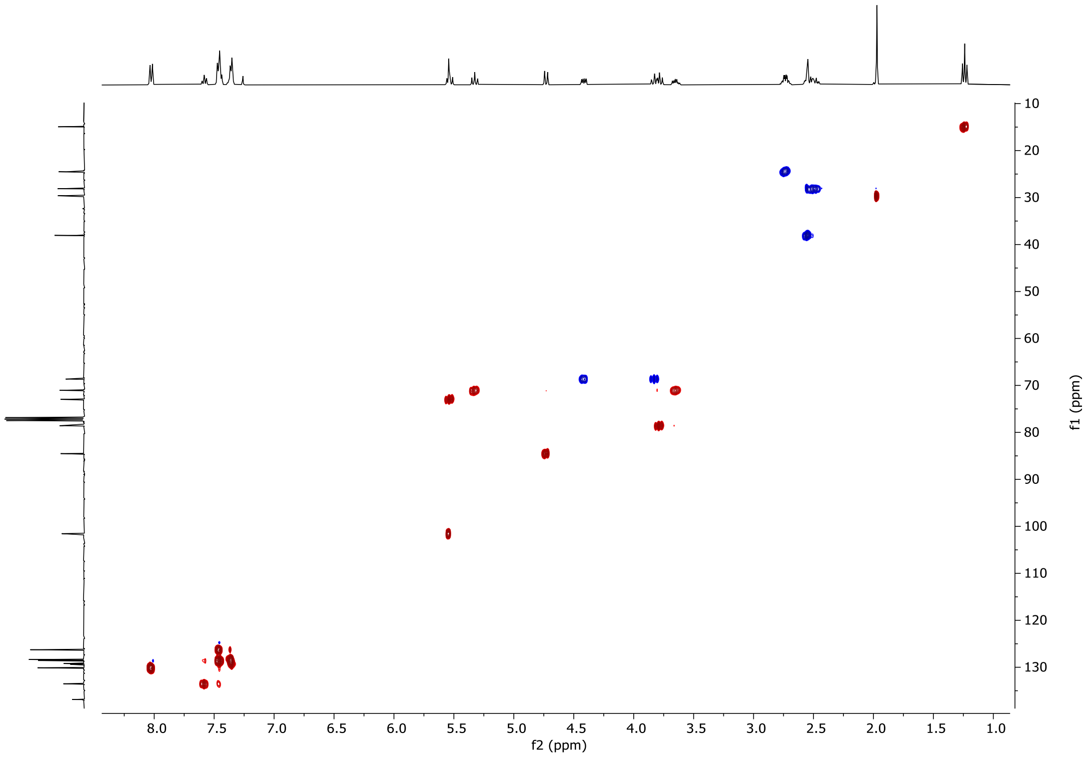

2.4.3 S-11

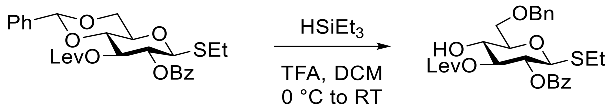

S-10 ... **S-11**, 75%

S-10 (275 mg, 0.53 mmol) was dissolved in anhydrous DCM (10 mL) and stirred in the presence of molecular sieves (4 Å, pellet, flame dried) under Ar atmosphere. The stirred reaction mixture was cooled to 0 °C and $HSiEt_3$ (500 µL, 3.2 mmol) and TFA (235 µL, 3.2 mmol) were added. After 20 min, the reaction was allowed to RT and stirred for 3 h, after which time it was quenched by addition of a saturated aqueous solution of $NaHCO_3$. The crude product was diluted with DCM and washed once with a saturated aqueous solution of $NaHCO_3$, and once with brine. The organic layer was dried over Na_2SO_4 and concentrated under reduced pressure. The crude product was purified by silica gel flash column chromatography (Hexane : EtOAc, 2:1→3:2→1:1→2:3) to yield **S-11** as a colorless viscous oil (205 mg, 75%).

^1H NMR (400 MHz, $CDCl_3$) δ 8.01 (d, *J* = 7.9 Hz, 2H), 7.57 (t, *J* = 7.3 Hz, 1H), 7.44 (t, *J* = 7.6 Hz, 2H), 7.37 – 7.27 (m, 5H), 5.30 (t, *J* = 9.2 Hz, 1H), 5.23 (t, *J* = 9.5 Hz, 1H), 4.67 – 4.59 (m, 3H), 3.91 – 3.75 (m, 3H), 3.68 – 3.60 (m, 1H), 3.42 (s, 1H), 2.81 – 2.58 (m, 4H), 2.56 – 2.44 (m, 1H), 2.43 – 2.32 (m, 1H), 2.06 (s, 3H), 1.24 (t, *J* = 7.4 Hz, 3H). ^{13}C NMR (101 MHz, $CDCl_3$) δ 207.50, 172.85, 165.47, 137.98, 133.42, 130.03, 129.50, 128.54, 127.87, 127.81, 83.65, 78.87, 77.16, 73.78, 70.37, 70.02, 38.43, 29.71, 28.31, 24.37, 15.01. $[\alpha]_D^{20}$ -1.19 (c = 1.00 g/100mL, $CHCl_3$). IR (film) ν = 3483, 2929,m 1725, 1271, 1070, 713 cm^{-1}. R_f = 0.16 (Hexane : EtOAc = 4:1). ESI-HRMS m/z 539.1705 $[M+Na]^+$ ($C_{27}H_{32}O_8SNa$ requires 539.1710).

^{1}H NMR of S-11 (400 MHz, $CDCl_3$)

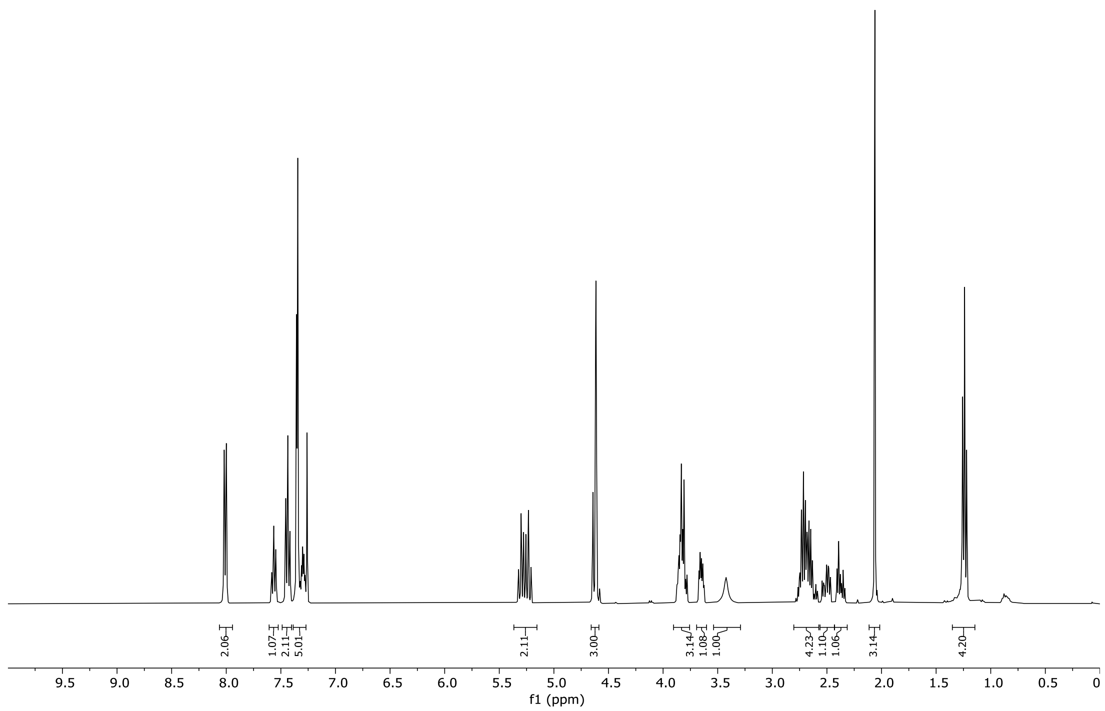

^{13}C NMR of S-11 (101 MHz, $CDCl_3$)

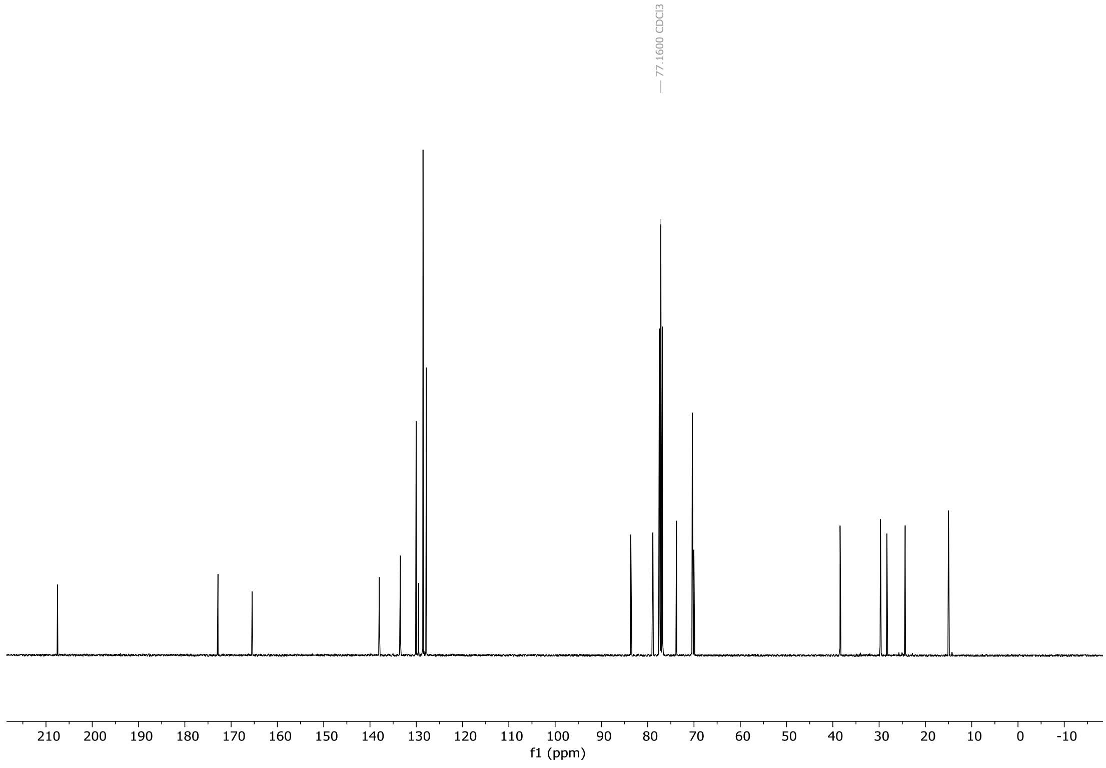

COSY NMR of S-11 ($CDCl_3$)

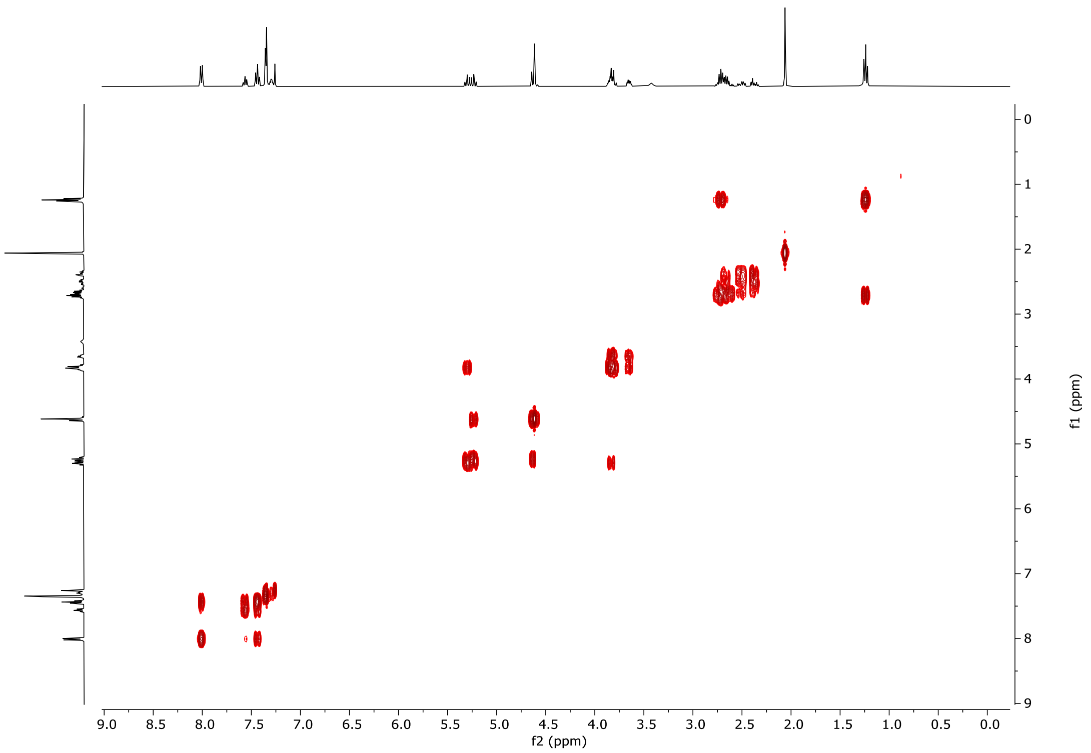

HSQC NMR of S-11 ($CDCl_3$)

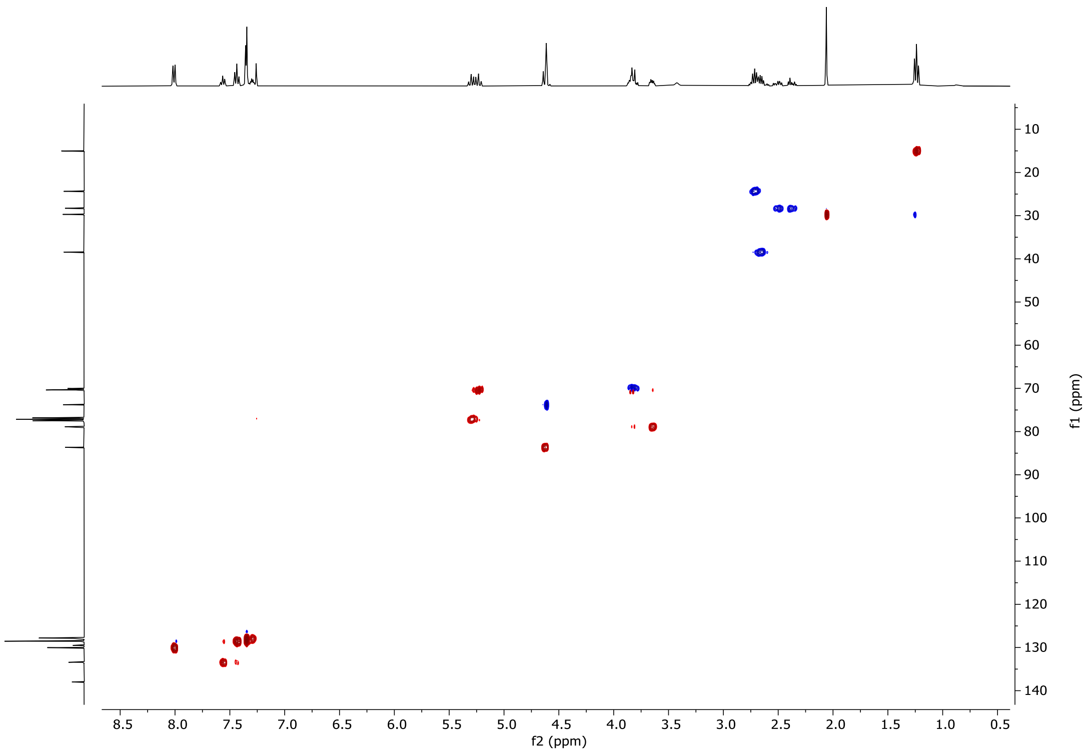

2.4.4 BB4

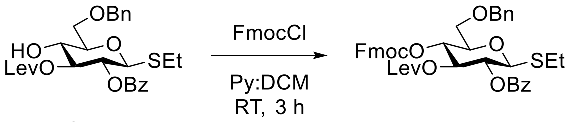

S-11 (205 mg, 0.4 mmol) was dissolved in anhydrous DCM (6 mL) and cooled to 0 °C under Ar atmosphere. FmocCl (205 mg, 0.8 mmol) was dissolved in DCM (0.7 mL) and added dropwise to the stirred reaction mixture. The solution was stirred for 15 min at 0 °C and then allowed to RT and stirred for additional 5 h, after which time the reaction was quenched by addition of a saturated aqueous solution of $NaHCO_3$. The reaction mixture was diluted with DCM, washed once with an aqueous solution of citric acid (0.5 M), and once with brine. The organic layer was dried with Na_2SO_4 and concentrated under reduced pressure. The crude product was purified using silica gel flash column chromatography (Hexane : EtOAc, 3:1→2:1→1:1) to yield **BB4** as a colorless foam (208 mg, 70%).

^{1}H NMR (600 MHz, $CDCl_3$) δ 8.01 (d, *J* = 7.3 Hz, 2H), 7.76 (dd, *J* = 7.5, 3.5 Hz, 2H), 7.61 – 7.55 (m, 3H), 7.45 (t, *J* = 7.8 Hz, 2H), 7.40 (q, *J* = 7.1 Hz, 2H), 7.34 – 7.27 (m, 6H), 7.22 (t, *J* = 7.2 Hz, 1H), 5.54 (t, *J* = 9.5 Hz, 1H), 5.31 (t, *J* = 9.7 Hz, 1H), 5.05 (t, *J* = 9.7 Hz, 1H), 4.66 (d, *J* = 10.0 Hz, 1H), 4.58 (d, *J* = 12.0 Hz, 1H), 4.53 (d, *J* = 12.0 Hz, 1H), 4.45 (dd, *J* = 10.1, 7.3 Hz, 1H), 4.28 (dd, *J* = 10.1, 7.8 Hz, 1H), 4.23 (t, *J* = 7.4 Hz, 1H), 3.88 – 3.82 (m, 1H), 3.72 – 3.64 (m, 2H), 2.81 – 2.68 (m, 2H), 2.56 – 2.37 (m, 3H), 2.36 – 2.29 (m, 1H), 1.89 (s, 3H), 1.26 (t, *J* = 7.4 Hz, 3H). ^{13}C NMR (151 MHz, $CDCl_3$) δ 205.68, 171.95, 165.30, 154.24, 143.54, 143.41, 141.41, 141.38, 137.90, 133.53, 130.11, 129.38, 128.49, 128.03, 127.82, 127.81, 127.34, 127.32, 125.43, 125.40, 120.18, 120.15, 83.75, 77.44 (d, *J* = 10.6 Hz), 73.96, 73.76, 73.26, 70.58, 69.17, 46.70, 37.87, 29.49, 28.09, 24.40, 15.02. $[\alpha]_D^{20}$ +18.81 (c = 1.00 g/100mL, $CHCl_3$). IR (film) ν = 2929, 1756, 1725, 1253 cm^{-1}. R_f = 0.35 (Hexane : EtOAc 2:1). ESI-HRMS m/z 761.2391 $[M+Na]^+$ ($C_{42}H_{42}O_{10}SNa$ requires 761.2391).

^{1}H NMR of BB4 (600 MHz, $CDCl_3$)

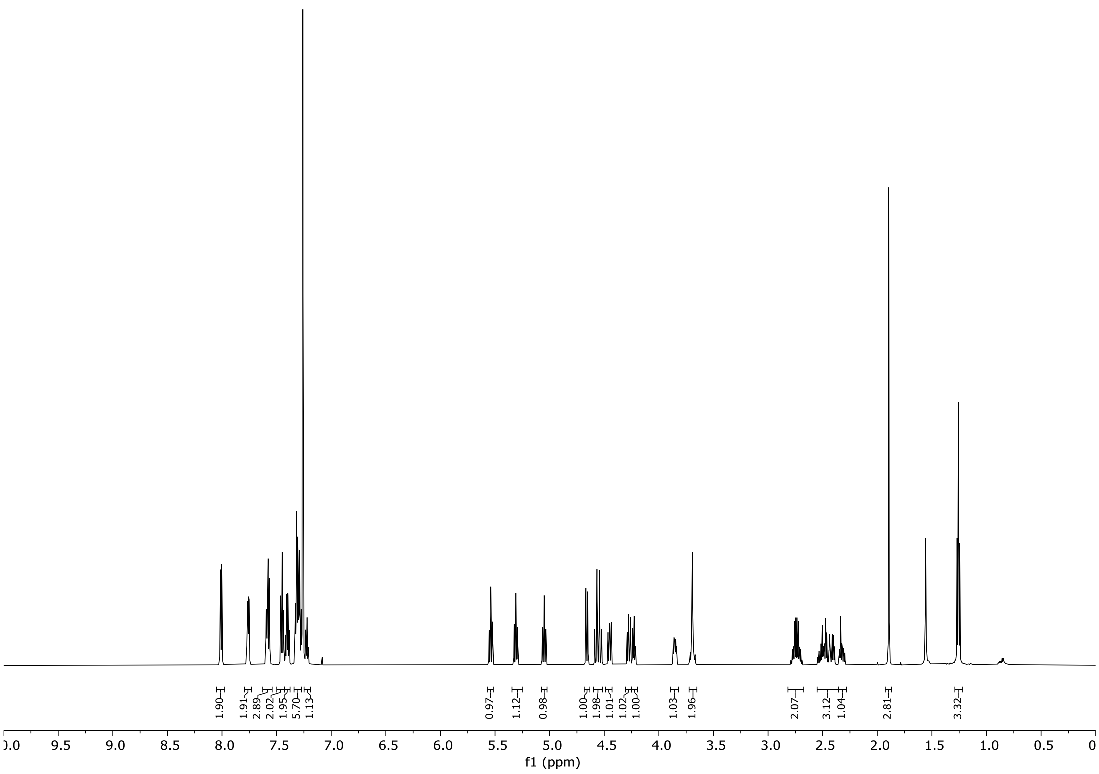

^{13}C NMR of BB4 (151 MHz, $CDCl_3$)

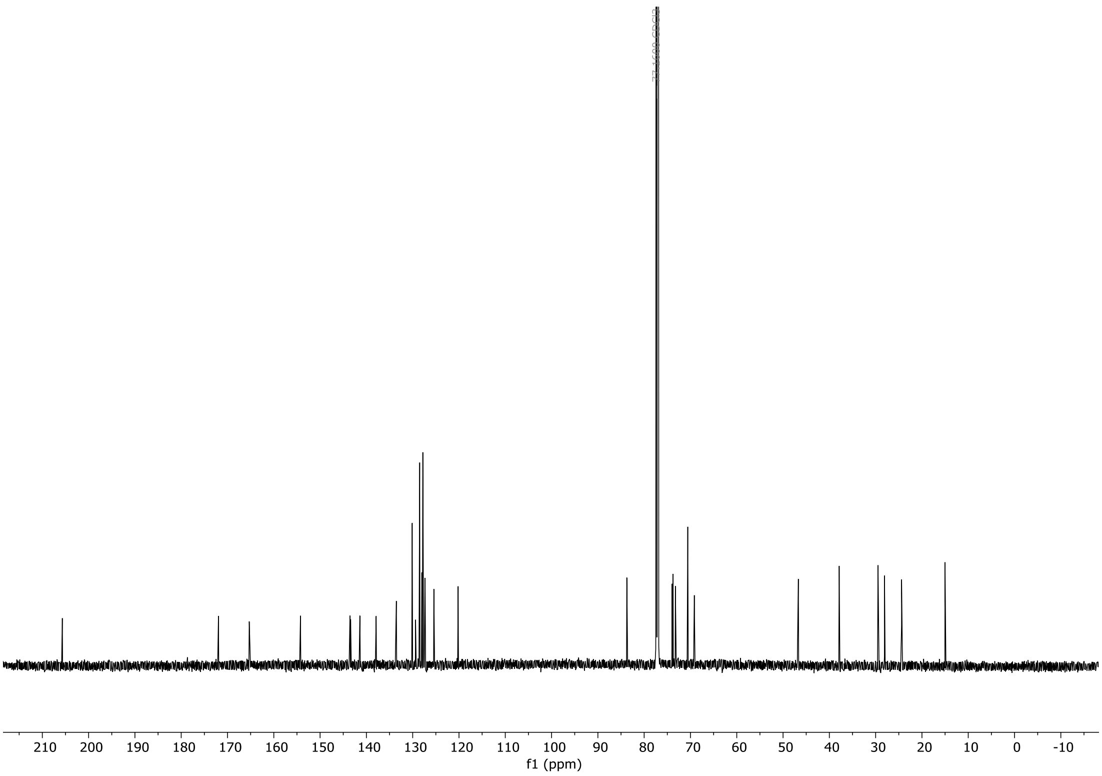

COSY NMR of BB4 ($CDCl_3$)

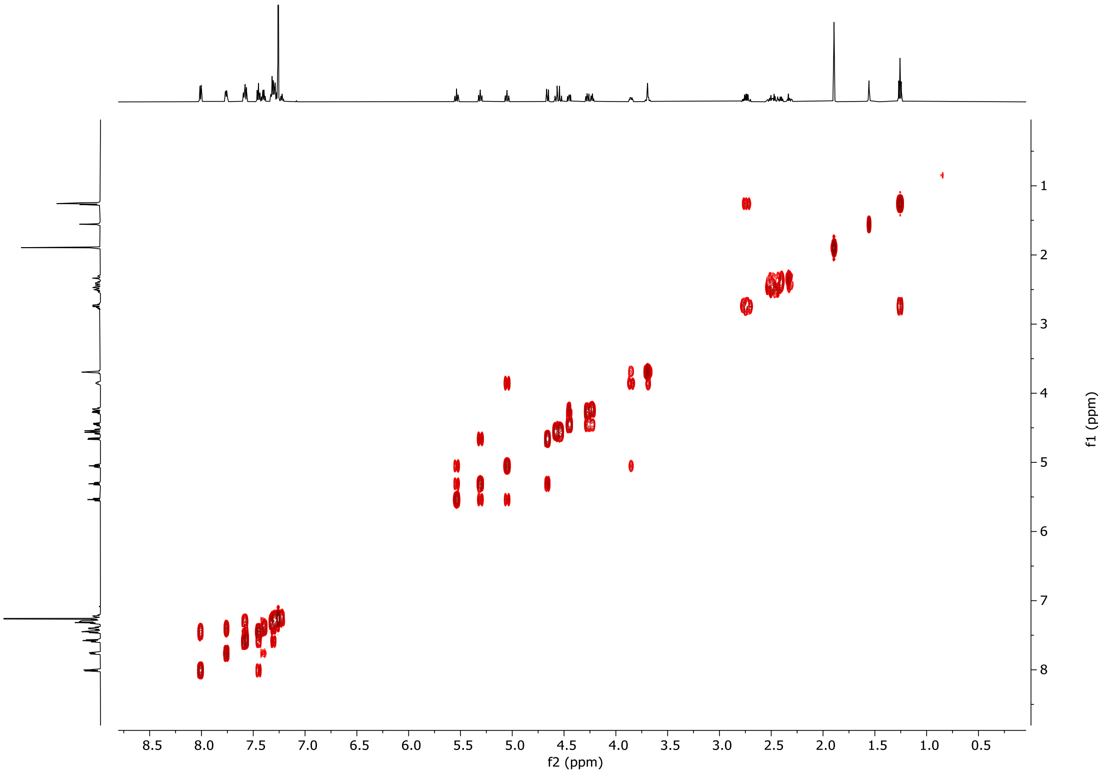

HSQC NMR of BB4 ($CDCl_3$)

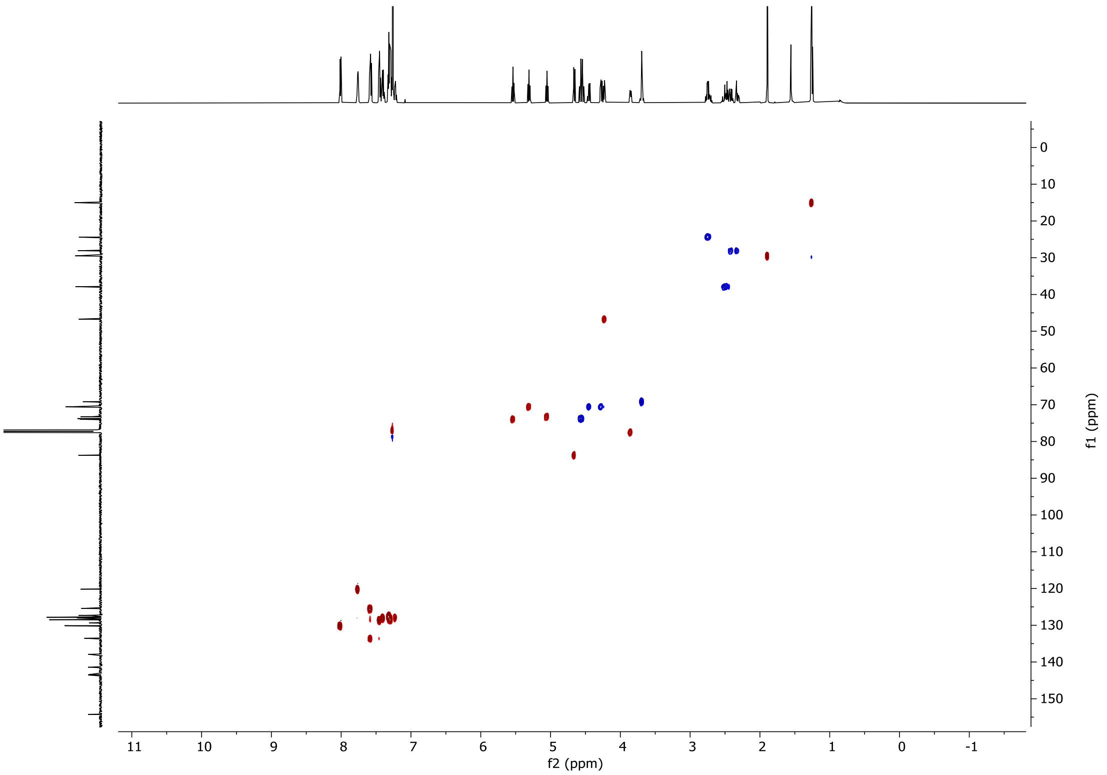

3 Automated glycan assembly

3.1 General materials and methods

The automated syntheses were performed on a home-built synthesizer developed at the Max Planck Institute of Colloids and Interfaces. All solvents used were HPLC-grade. The solvents used for the building blocks, activator, TMSOTf and capping solutions were taken from an anhydrous solvent system (J.C. Meyer). The building blocks were co-evaporated three times with toluene and dried for 1 h on high vacuum before use. Oven-heated, argon-flushed flasks were used to prepare all moisture-sensitive solutions. Activator, capping, deprotection, acidic wash and building block solutions were freshly prepared and kept under argon during the automation run. All yields of products obtained by AGA were calculated on the basis of resin loading. Resin loading was determined following previously established procedures.[6]

3.2 Preparation of stock solutions

- **Building block solution**: Between 0.06 and 0.10 mmol of building block (depending on the BB, see Module C1 and C2) was dissolved in DCM (1 mL).
- **NIS/TfOH activator solution**: 1.35 g (6.0 mmol) of recrystallized NIS was dissolved in 40 mL of a 2:1 v/v mixture of anhydrous DCM and anhydrous dioxane. Then triflic acid (55 µL, 0.6 mmol) was added. The solution is kept at 0 °C (ice bath) for the duration of the automation run.
- **Fmoc deprotection solution**: A solution of 20%$_{v/v}$ piperidine in DMF was prepared.
- **Lev deprotection solution**: Hydrazine acetate (550 mg, 5.97 mmol) was dissolved in pyridine/AcOH/H_2O (40mL, v/v, 32:8:2) and sonicated for 10 min.
- **TMSOTf solution**: TMSOTf (0.45 mL, 2.49 mmol) was added to DCM (40 mL).
- **Capping solution**: A solution of 10%$_{v/v}$ acetic anhydride and 2%$_{v/v}$ methanesulfunic acid in DCM was prepared.

3.3 Modules for automated synthesis

3.3.1 Module A: Resin preparation

All automated syntheses were performed on 0.0125 mmol scale. Resin (**L1** or **L2**) is placed in the reaction vessel and swollen in DCM for 20 min at room temperature prior to the synthesis. During this time, all reagent lines needed for the synthesis are washed and primed. After the swelling, the resin is washed with DMF, THF, and DCM (three times each with 2 mL for 25 s).

3.3.2 Module B: Acidic wash with TMSOTf solution (20 min)

The resin is swollen in 2 mL DCM and the temperature of the reaction vessel adjusted to -20 °C. Upon reaching the low temperature, TMSOTf solution (1 mL) is added dropwise to the reaction vessel. After bubbling for 3 min, the acidic solution is drained and the resin washed with 2 mL DCM for 25 s.

Action	Cycles	Solution	Amount	T (°C)	Incubation time
**Cooling**	-	-	-	-20	(15 min)*
**Deliver**	1	DCM	2 mL	-20	-
**Deliver**	1	TMSOTf solution	1 mL	-20	3 min
**Wash**	1	DCM	2 mL	-20	25 sec

*Time required to reach the desired temperature.

3.3.3 Module C1: Thioglycoside glycosylation (35 min-55 min)

The building block solution (0.10 mmol of BB in 1 mL of DCM per glycosylation) was delivered to the reaction vessel. After the set temperature was reached, the reaction was started by dropwise addition of the NIS/TfOH activator solution (1.0 mL, excess). The glycosylation conditions (T_1, T_2, t_1, and t_2) are building block dependent and are reported in a table below. After completion of the reaction, the solution was drained and the resin was washed with DCM, DCM:dioxane (1:2, 3 mL for 20 s) and DCM (two times, each with 2 mL for 25 s). The temperature of the reaction vessel was increased to 25 °C for the next module. In case of a double cycle (C1*, *Double cycle), module C1 was repeated twice.

Action	Cycles	Solution	Amount	T (°C)	Incubation time
**Cooling**	-	-	-	T_1	-
**Deliver**	1	BB solution	1 mL	T_1	-
**Deliver**	1	NIS/TfOH activator solution	1 mL	T_1	-
**Reaction time (BB dependent)**	1	-	-	T_1 to T_2	t_1 t_2
**Wash**	1	DCM	2 mL	T_2	5 sec
**Wash**	1	DCM : Dioxane (1:2)	2 mL	T_2	20 sec
**Heating**	-	-	-	25	-
**Wash**	2	DCM	2 mL	> 0	25 sec

BB	Equiv.	t_1 (min)	T_1 (°C)	t_2 (min)	T_2 (°C)
**BB1**	6.5	5	-20	40	0
**BB2a**	6.5	5	-20	20	0
**BB3a**	6.5	5	-20	20	0
**BB3b**	6.5	5	-20	20	0
**BB4**	6.5	5	-20	20	0

3.3.4 Module C2: Glycosyl phosphate glycosylation (45 min)

The building block solution (0.06 mmol of BB in 1 mL of DCM per glycosylation) is delivered to the reaction vessel. After the set temperature is reached, the reaction is started by dropwise addition of the TMSOTf solution (1.0 mL, stoichiometric). After completion of the reaction, the solution is drained and the resin washed with DCM (six times, each with 2 mL for 25 s). The temperature of the reaction vessel is increased to 25 °C for the next module. In case of a double cycle (C2*, *Double cycle), module C2 was repeated twice.

Action	Cycles	Solution	Amount	T (°C)	Incubation time
**Cooling**	-	-	-	-30	-
**Deliver**	1	BB solution	1 mL	-30	-
**Deliver**	1	TMSOTf solution	1 mL	-30	-
**Reaction time (BB dependent)**	1	-	-	-30 to -10	5 min 40 min
**Wash**	1	DCM	2 mL	-10	5 sec
**Heating**	-	-	-	25	-
**Wash**	6	DCM	2 mL	> 0	25 sec

BB	Equiv.	t1 (min)	T1 (°C)	t2 (min)	T2 (°C)
**BB2b**	5	5	-30	40	-10
**^{13}C-BB2b**	5	5	-30	40	-10

3.3.5 Module D: Capping (30 min)

The resin is washed with DMF (two times with 2 mL for 25 s) and the temperature of the reaction vessel adjusted to 25 °C. A pyridine solution (2 mL, 10%$_{v/v}$ in DMF) is delivered into the reaction vessel. After 1 min, the reaction solution is drained and the resin washed with DCM (three times with 3 mL for 25 s). Capping solution (4 mL) is delivered into the reaction vessel. After 20 min, the reaction solution is drained and the resin washed with DCM (three times with 3 mL for 25 s).

Action	Cycles	Solution	Amount	T (°C)	Incubation time
**Heating**	-	-	-	25	(5 min)*
**Wash**	2	DMF	2 mL	25	25 sec
**Deliver**	1	10% Pyridine in DMF	2 mL	25	1 min
**Wash**	3	DCM	2 mL	25	25 sec
**Deliver**	1	Capping Solution	4 mL	25	20 min
**Wash**	3	DCM	2 mL	25	25 sec

*Time required to reach the desired temperature.

3.3.6 Module E1: Fmoc deprotection (9 min)

The resin is washed with DMF (three times with 2 mL for 25 s) and the temperature of the reaction vessel adjusted to 25 °C. Fmoc deprotection solution (2mL) is delivered to the reaction vessel and kept under Ar bubbling. After 5 min, the reaction solution is drained and the resin washed with DMF (three times with 3 mL for 25 s) and DCM (five times each with 2 mL for 25 s). The temperature of the reaction vessel is decreased to -20 °C for the next module.

Action	Cycles	Solution	Amount	T (°C)	Incubation time
**Wash**	3	DMF	2 mL	25	25 sec
**Deliver**	1	Fmoc depr. solution	2 mL	25	5 min
**Wash**	1	DMF	2 mL		
**Cooling**	-	-	-	-20	-
**Wash**	3	DMF	2 mL	< 25	25 sec
**Wash**	5	DCM	2 mL	< 25	25 sec

3.3.7 Module E2: Lev deprotection (90 min)

The resin is washed with DCM (three times with 2 mL for 25 s). DCM (1.3 mL) is delivered to the reaction vessel and the temperature of the reaction vessel is adjusted to 25 °C. Lev deprotection solution (2mL) is delivered to the reaction vessel, kept under pulsed Ar bubbling for 30 min. This procedure is repeated twice. The reaction solution is drained and the resin washed with DMF (three times with 3 mL for 25 s) and DCM (five times each with 2 mL for 25 s).

Action	Cycles	Solution	Amount	T (°C)	Incubation time
**Wash**	3	DMF	2 mL	25	25 sec
**Deliver**	2	Lev depr. solution	2 mL	25	30 min
**Wash**	1	DMF	2 mL	-	-
**Cooling**	-	-	-	-20	-
**Wash**	3	DMF	2 mL	< 25	25 sec
**Wash**	5	DCM	2 mL	< 25	25 sec

3.4 Post-AGA manipulations

3.4.1 Module F: On-resin methanolysis

The resin is suspended in THF (4 mL). MeONa in MeOH (0.5 M, 0.4 mL) is added and the suspension is gently shaken at room temperature. After micro-cleavage (see *Module G2*) indicates the complete removal of benzoyl groups, the resin is repeatedly washed with MeOH (3 x 2 mL) and DCM (3 x 2 mL).

3.4.2 Module G1: Cleavage from solid support

The oligosaccharides are cleaved from the solid support using a continuous-flow photoreactor as described previously.[7]

3.4.3 Module G2: Micro-cleavage from solid support

Trace amount of resin (around 20 beads) is dispersed in DCM (0.1 mL) and irradiated with a UV lamp (6 W, 356 nm) for 10 minutes. ACN (10 µL) is then added to the resin and the resulting solution analyzed by MALDI.

3.4.4 Module H1: Hydrogenolysis

The crude compound obtained from *Module G1* is dissolved in 2 mL of EtOAc:*t*BuOH:H_2O (2:1:1). 100% by weight Pd/C (10%$_w$) or $Pd(OH)_2$/C (10-20%$_w$, moistened with water) is added and the reaction stirred in a pressurized reactor under H_2 pressure (4 bar). The reaction progress is monitored to avoid undesired

side products formation (*i.e.* degradation of reducing end).[8] Upon completion, the reaction is filtered (PTFE 0.45 µm 25 mm syringe filter, Fisher scientific) and washed with EtOAc, H_2O, and ACN (4 mL each). The filtrates are concentrated *in vacuo.*

3.4.5 Module H2: Hydrogenolysis at ambient pressure

The crude compound obtained from *Module G1* is dissolved in 2 mL of EtOAc:*t*BuOH:H_2O (2:1:1). 100% by weight Pd/C (10%$_w$) is added to the stirred flask, the reaction purged for 5 min with a N_2 balloon, and equipped with a H_2 balloon. The reaction progress is monitored to avoid undesired side products formation (*i.e.* degradation of reducing end).[8] Upon completion, the reaction is filtered (PTFE 0.45 µm 25 mm syringe filter, Fisher scientific) and washed with EtOAc, H_2O, and ACN (4 mL each). The filtrates are concentrated *in vacuo.*

3.4.6 Module I: Purification

The final compounds are analyzed using analytical reversed phase HPLC (Agilent 1200 Series, Methods A1, B1, and C1). The purification of the crudes is conducted using reversed phase HPLC (Agilent 1200 Series, Method A2, B2, and C2).

- **Method A1**: (Hypercarb column, ThermoFisher scientific, 150 x 4.6 mm, 3 µm) flow rate of 0.7 mL/min with H_2O (0.1% formic acid) and ACN as eluents [isocratic (5 min), linear gradient to 30% ACN (30 min), linear gradient to 100% ACN (5 min), isocratic 100% ACN (5 min)].

- **Method A2 (Prep)**: (Hypercarb column, ThermoFisher scientific, 150 x 10 mm, 5 µm), flow rate of 3 mL /min with H_2O (0.1% formic acid) and ACN as eluents [isocratic (5 min), linear gradient to 30% ACN (30 min), linear gradient to 100% ACN (5 min), isocratic 100% ACN (5 min)].

- **Method B1**: (Hypercarb column, ThermoFisher scientific, 150 x 4.6 mm, 3 µm) flow rate of 0.7 mL/min with H_2O (0.1% formic acid) and ACN as eluents [isocratic (5 min), linear gradient to 60% ACN (30 min), linear gradient to 100% ACN (5 min), isocratic 100% ACN (5 min)].

- **Method B2 (Prep)**: (Hypercarb column, ThermoFisher scientific, 150 x 10 mm, 5 µm), flow rate of 3 mL/min with H_2O (0.1% formic acid) and ACN as eluents [isocratic (5 min), linear gradient to 60% ACN (30 min), linear gradient to 100% ACN (5 min), isocratic 100% ACN (5 min)].

- **Method C1**: (Synergi Hydro RP18 column, Phenomenex, 250 x 4.6 mm), flow rate of 1.0 mL/min with H_2O (0.1% formic acid) and ACN as eluents [isocratic (5 min), linear gradient to 30% ACN (30 min), linear gradient to 100% ACN (5 min), isocratic 100% ACN (5 min)].

- **Method C2 (Prep)**: (Synergi Hydro RP18 column, Phenomenex, 250 x 10 mm) flow rate of 4.0 mL/min with H_2O (0.1% formic acid) and ACN as eluents [isocratic (5 min), linear gradient to 30% ACN (30 min), linear gradient to 100% ACN (5 min), isocratic 100% ACN (5 min)].

Following final purification, all deprotected products are lyophilized on a Christ Alpha 2-4 LD plus freeze dryer prior to characterization.

3.5 Oligosaccharide synthesis

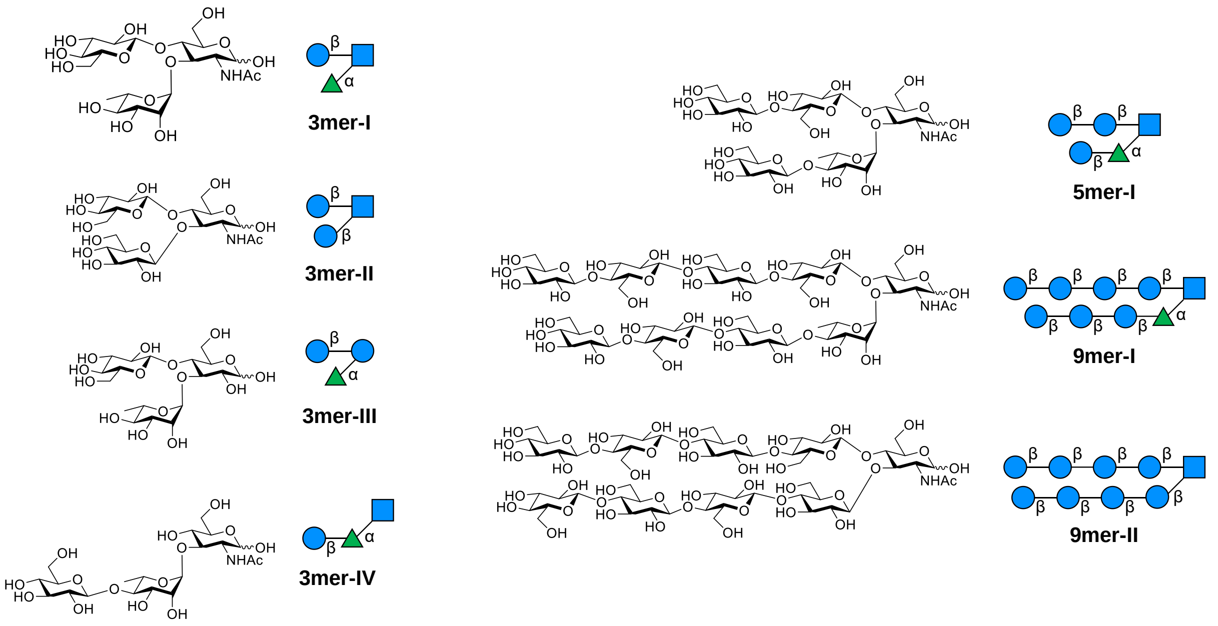

Figure 2 Oligosaccharides synthesized by AGA in this work.

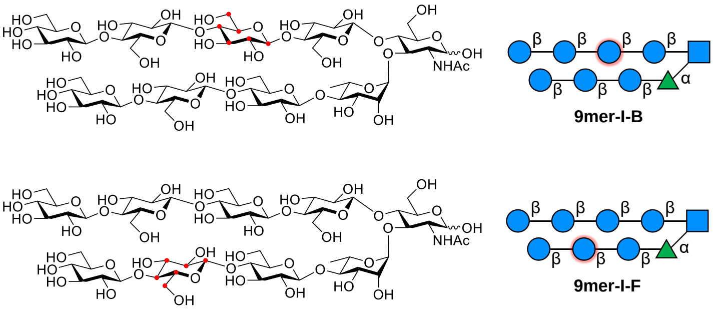

Figure 3 ^{13}C-labelled oligosaccharides synthesized by AGA in this work.

3.5.1 3mer-I

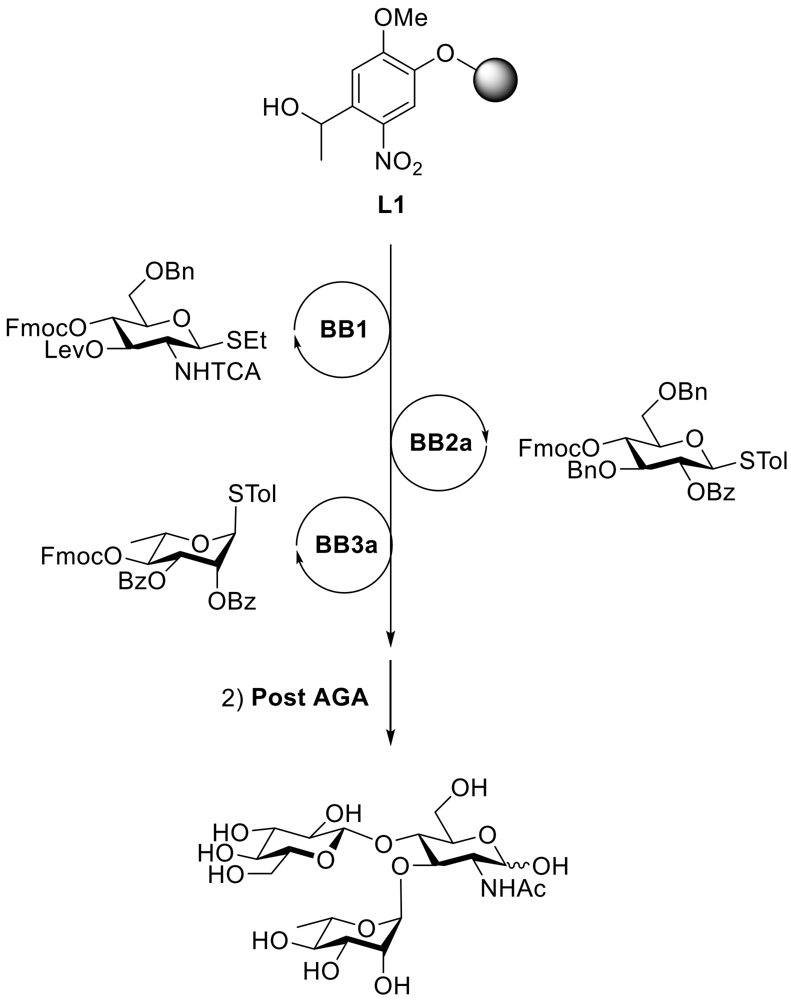

3mer-I

Step	BB	Modules	Notes
AGA	-	**A**	**L1** swelling
	**BB1**	**B**, **C1**, **D**, **E1**	**C1**: (**BB1**, -20 °C for 5 min, 0 °C for 40 min)
	**BB2a**	**B**, **C1***, **D**, **E1**	**C1***: (**BB2a**, -20 °C for 5 min, 0 °C for 20 min) *Double cycle
	-	**D**, **E2**	-
	**BB3a**	**B**, **C1**, **D**, **E1**	**C1**: (**BB3a**, -20 °C for 5 min, 0 °C for 20 min)
Post-AGA	-	**F, G1, H2, I**	**F**: (2 h)<br>**H2**: (2 h)<br>**I**: (Method A2: 17.1 min)

Automated synthesis, global deprotection, and purification afforded **3mer-I** as a white solid (1.3 mg, 20% overall yield).

^{1}H NMR (600 MHz, D_2O) δ 5.10 (d, *J* = 3.6 Hz, 0.6H, H-1α GlcNAc), 4.94 – 4.88 (m, 1H, H-1 Rha), 4.73 (d, *J* = 8.2 Hz, 0.4H, H-1β GlcNAc), 4.51 – 4.46 (m, 1H, H-1 Glc), 4.42 – 4.34 (m, 1H, H-5 Rha), 4.09 (dd, *J* = 10.1, 3.6 Hz, 0.6H), 4.00 – 3.67 (m, 9H), 3.59 – 3.52 (m, 0.4H), 3.47 (td, *J* = 9.2, 1.9 Hz, 1H), 3.43 – 3.36 (m, 2H), 3.32 (t, *J* = 9.4 Hz, 1H), 3.21 (ddd, *J* = 9.3, 7.9, 4.4 Hz, 1H), 2.03 (s, 3H, CH_3 Ac GlcNAc), 1.21 (d, *J* = 6.2 Hz, 3H, CH_3-6 Rha). ^{13}C NMR (151 MHz, D_2O) δ 174.11, 101.41 (C-1 Glc), 100.57 (C-1 Rha), 94.24 (C-1β GlcNAc), 90.85 (C-1α GlcNAc), 76.05, 75.47, 75.31, 75.20, 73.52, 73.25, 71.70, 71.13, 70.48, 69.95, 69.91, 69.64, 68.51 (C-5 Rha), 61.00, 60.98, 59.46, 56.82, 53.87, 21.85, 16.57. ESI-HRMS m/z 552.1973 $[M+Na]^+$ ($C_{20}H_{35}NO_{15}Na$ requires 552.1899).

RP-HPLC of crude 3mer-I (ELSD trace, Method A1)

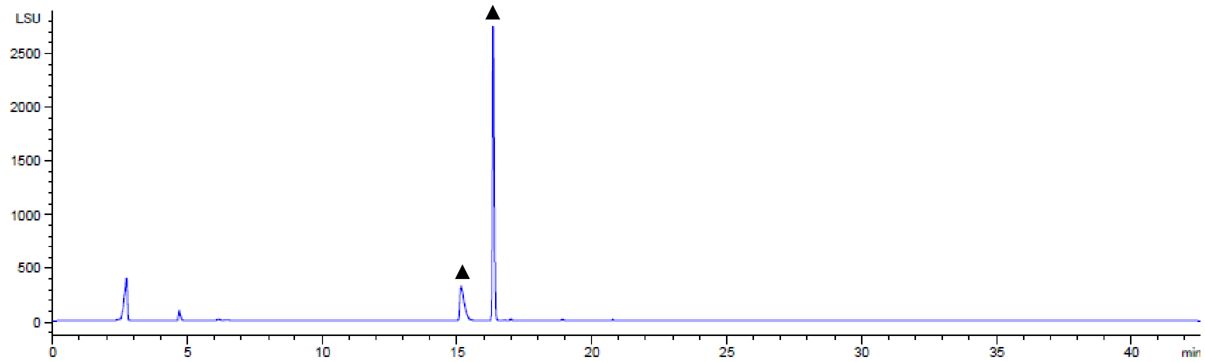

▲ **3mer-I** (α and β anomers, m/z 552 $[M+Na]^+$).

RP-HPLC of 3mer-I (ELSD trace, Method A1, t_R = 15.2, 16.3 min)

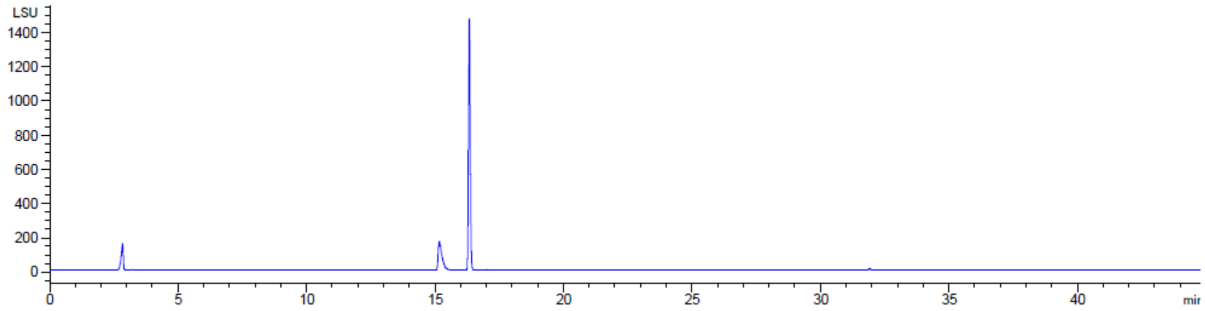

^{1}H NMR of 3mer-I (600 MHz, D_2O)

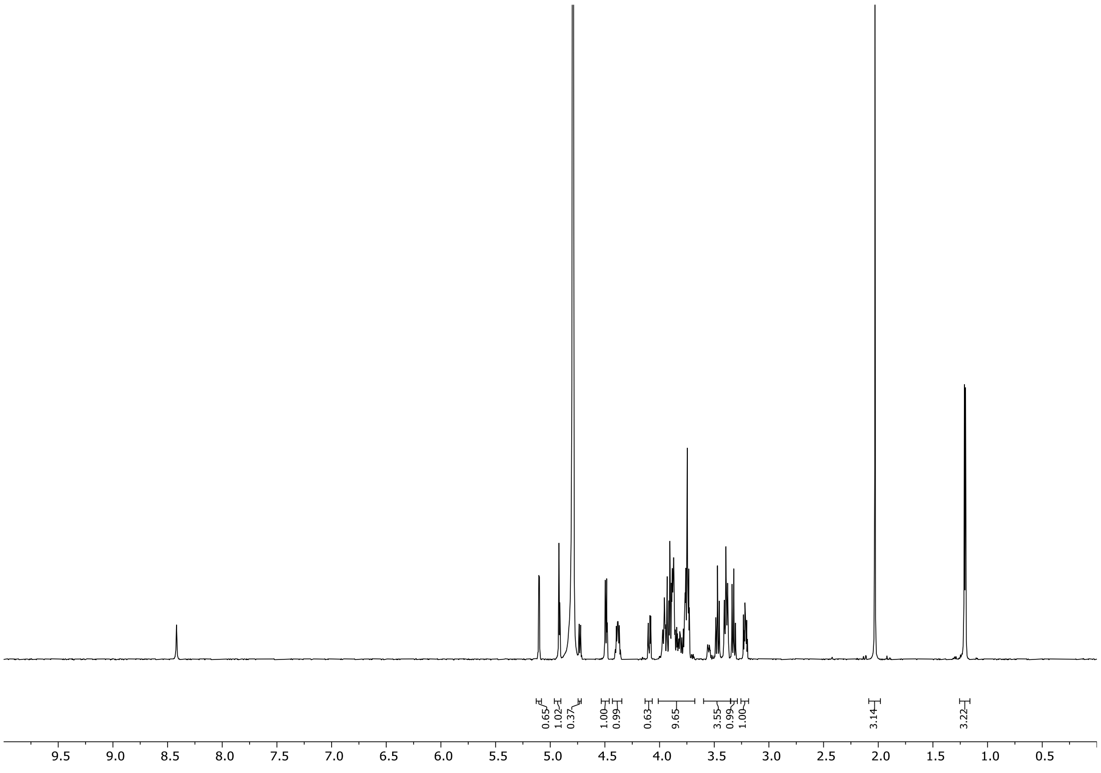

^{13}C NMR of 3mer-I (151 MHz, D_2O)

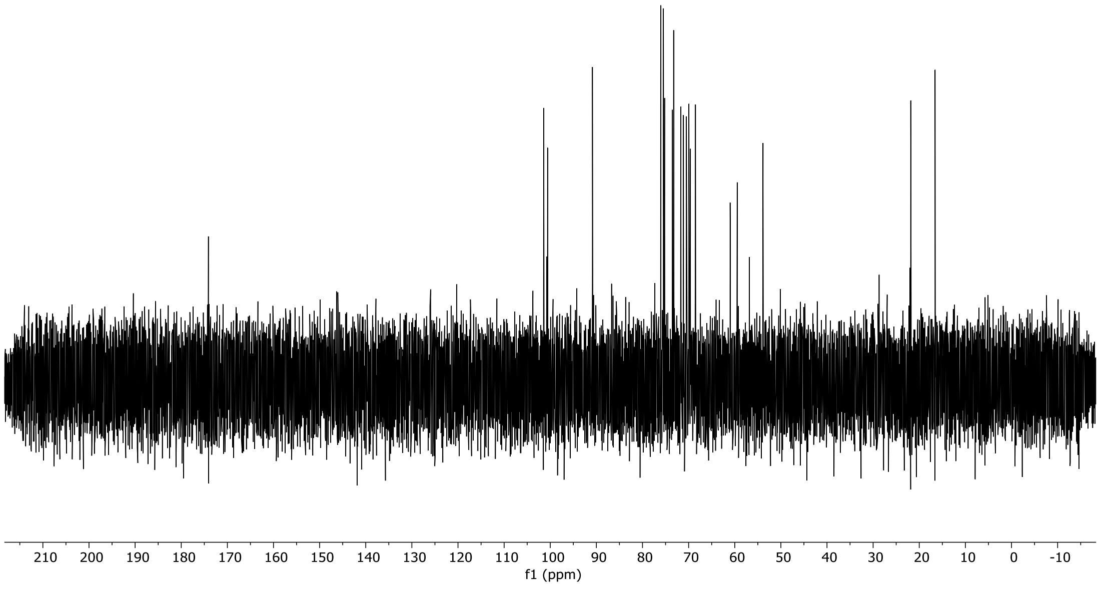

COSY NMR of 3mer-I (D_2O)

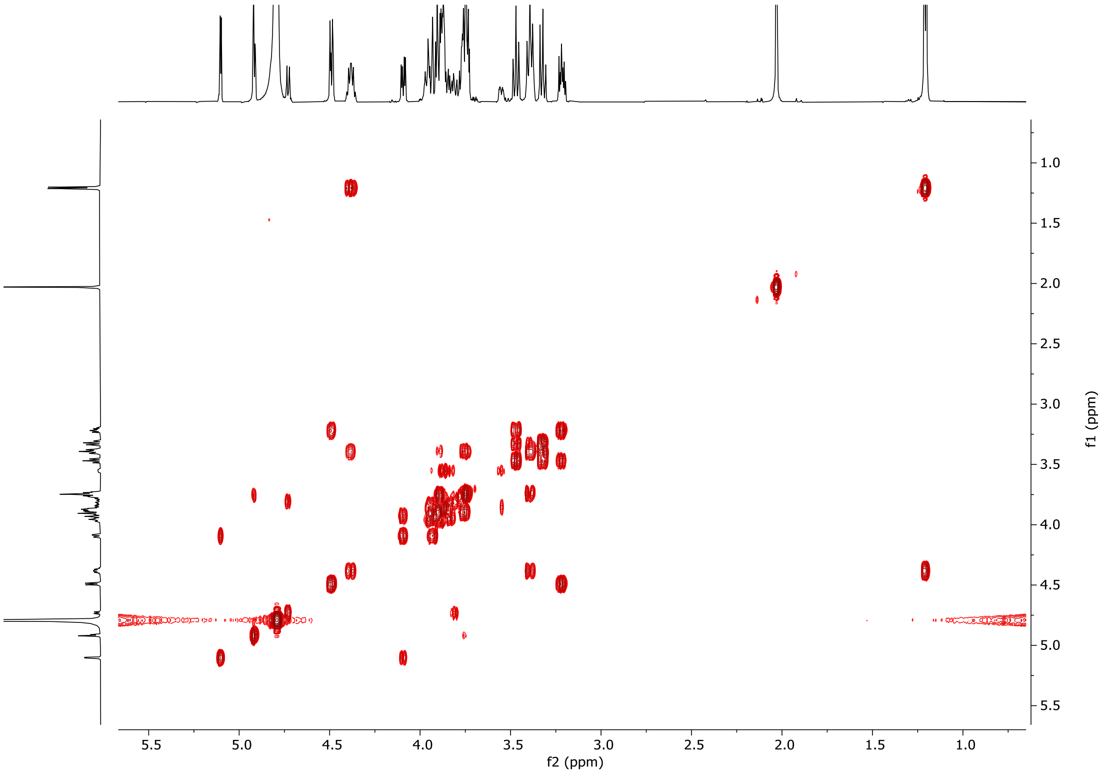

HSQC NMR of 3mer-I (D_2O)

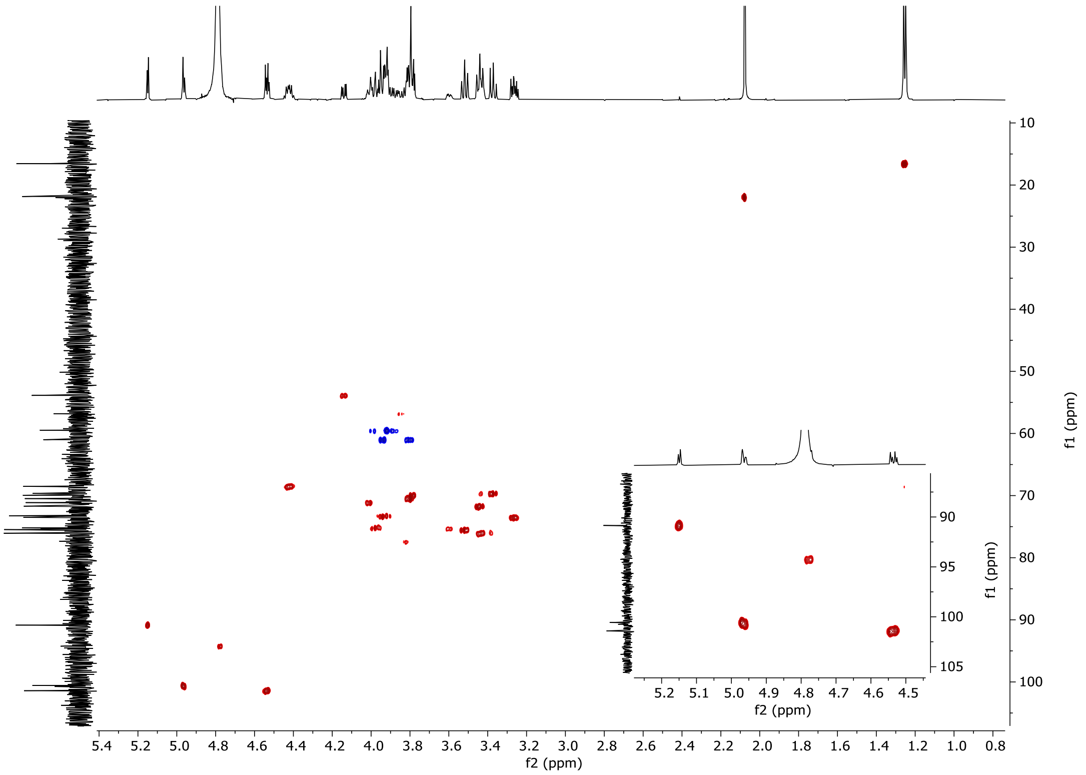

3.5.2 3mer-II

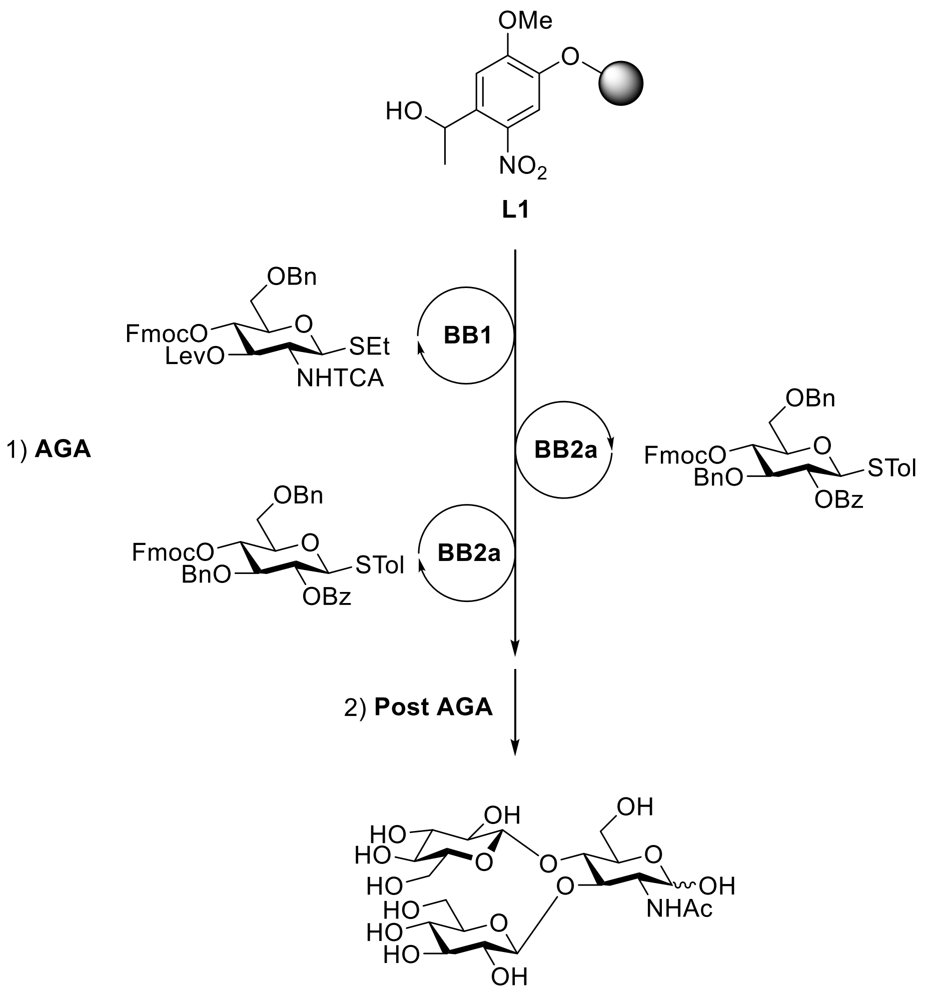

3mer-II

Step	BB	Modules	Notes
AGA	-	**A**	**L1** swelling
	**BB1**	**B**, **C1***, **D**, **E1**	**C1**: (**BB1**, -20 °C for 5 min, 0 °C for 40 min) *Double cycle
	**BB2a**	**B**, **C1***, **D**, **E1**	**C1**: (**BB2a**, -20 °C for 5 min, 0 °C for 20 min) *Double cycle
	-	**D**, **E2**	-
	**BB2a**	**B**, **C1**, **D**, **E1**	**C1**: (**BB2a**, -20 °C for 5 min, 0 °C for 20 min)
Post-AGA	-	**F**, **G1**, **H2**, **I**	**F**: (2 h)<br>**H2**: (8 h)<br>**I**: (Method A2: 19.1 min)

Automated synthesis, global deprotection, and purification afforded **3mer-II** as a white solid (0.7 mg, 11% overall yield).

^{1}H NMR (600 MHz, D_2O) ^{1}H NMR (600 MHz, D_2O) δ 5.09 (d, *J* = 3.5 Hz, 0.6H, H-1α GlcNAc), 4.62 (d, *J* = 8.4 Hz, 0.4H, H-1β GlcNAc), 4.61 – 4.53 (m, 2H, H-1 Glcx2), 4.21 – 4.15 (m, 0.6H), 4.05 – 4.00 (m, 0.4H), 3.98 (dd, *J* = 10.4, 3.5 Hz, 0.6H), 3.93 – 3.75 (m, 6H), 3.67 – 3.60 (m, 2H), 3.51 (s, 0.4H), 3.44 – 3.22 (m, 8H), 1.95 (s, 1.8H, CH_3 Ac GlcNAc α), 1.95 (s, 1.2H, CH_3 Ac GlcNAc β). ^{13}C NMR (176 MHz, D_2O) δ 101.14 (C-1 Glc), 100.96 (C-1 Glc), 94.80 (C-1β GlcNAc), 90.63 (C-1α GlcNAc), 76.22, 76.09, 75.68, 75.43, 75.40, 75.35, 73.11, 72.96, 72.89, 72.57, 71.36, 69.37, 60.66, 59.97, 53.71, 22.06. ESI-HRMS m/z 568.1872 $[M+Na]^+$ ($C_{20}H_{35}NO_{16}Na$ requires 568.1848).

RP-HPLC of crude 3mer-II (ELSD trace, Method A1)

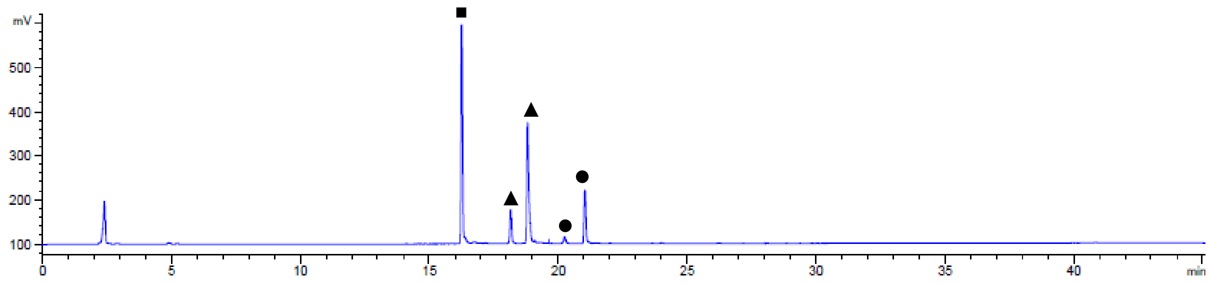

▲ **3mer-II** (α and β anomers, m/z 568 $[M+Na]^+$). ■ Sideproduct with GlcNAc reducing end hydrogenated (m/z 570 $[M+Na]^+$) formed during hydrogenolysis (Module I2). ● 2mer deletion sideproduct (m/z 406 $[M+Na]^+$).

RP-HPLC of 3mer-II (ELSD trace, Method A1, t_R = 18.2, 18.9 min)

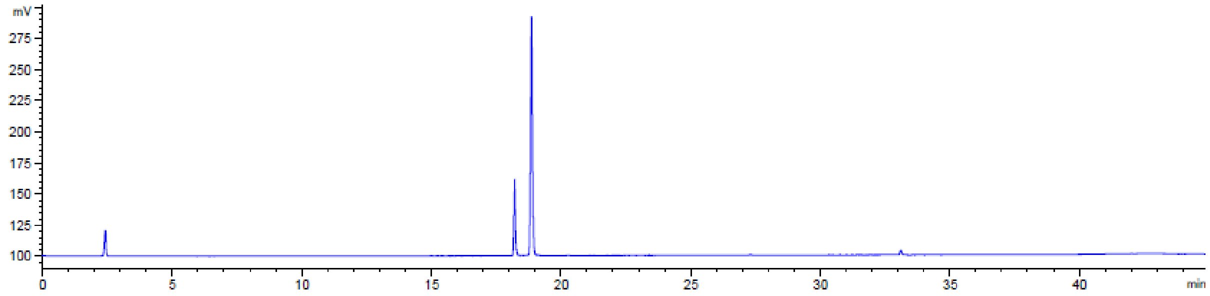

^1H NMR of 3mer-II (600 MHz, D_2O)

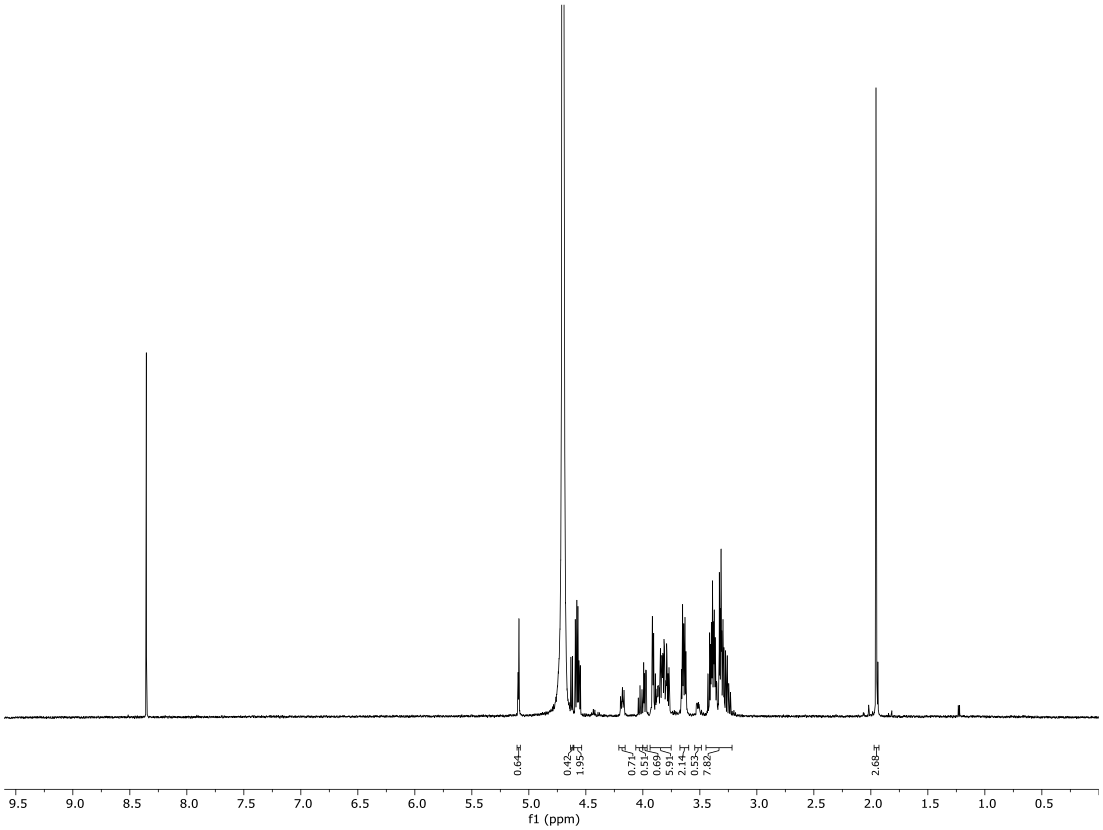

^{13}C NMR of 3mer-II (151 MHz, D_2O)

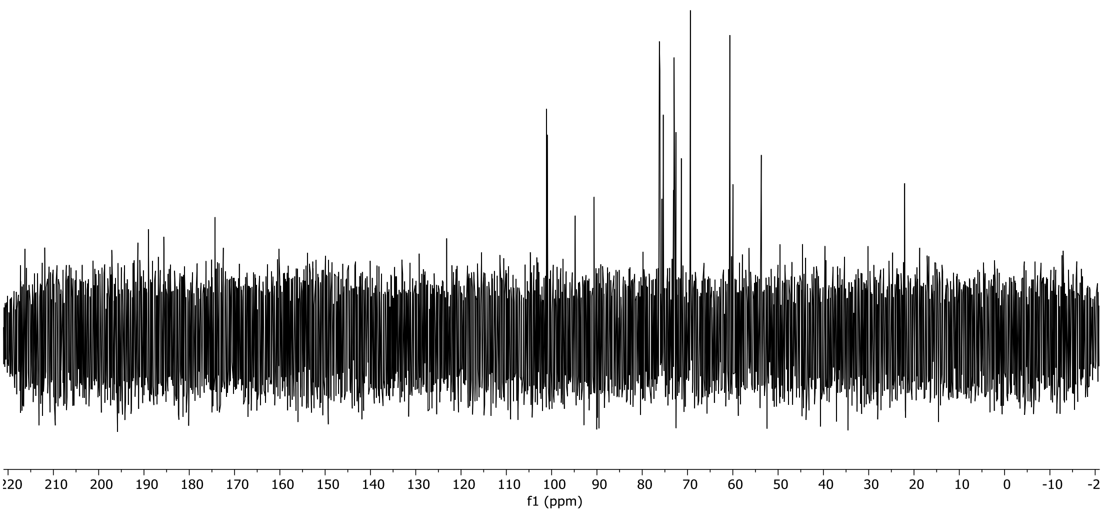

COSY NMR of 3mer-II (D_2O)

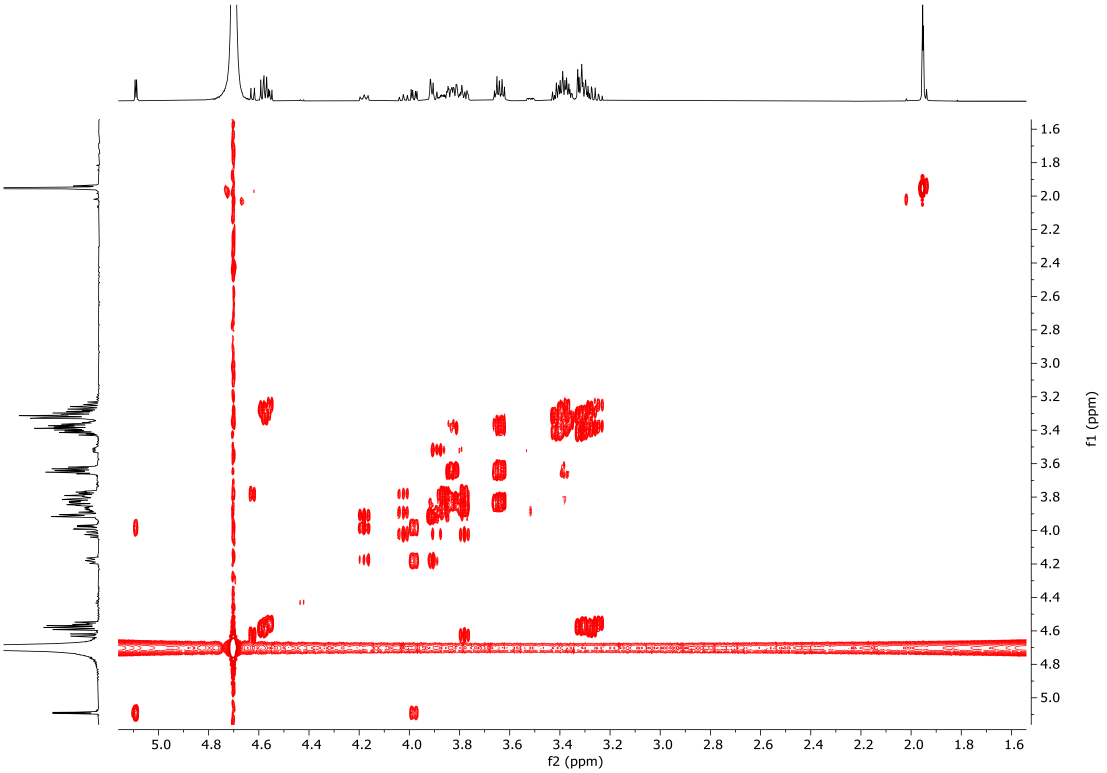

HSQC NMR of 3mer-II (D_2O)

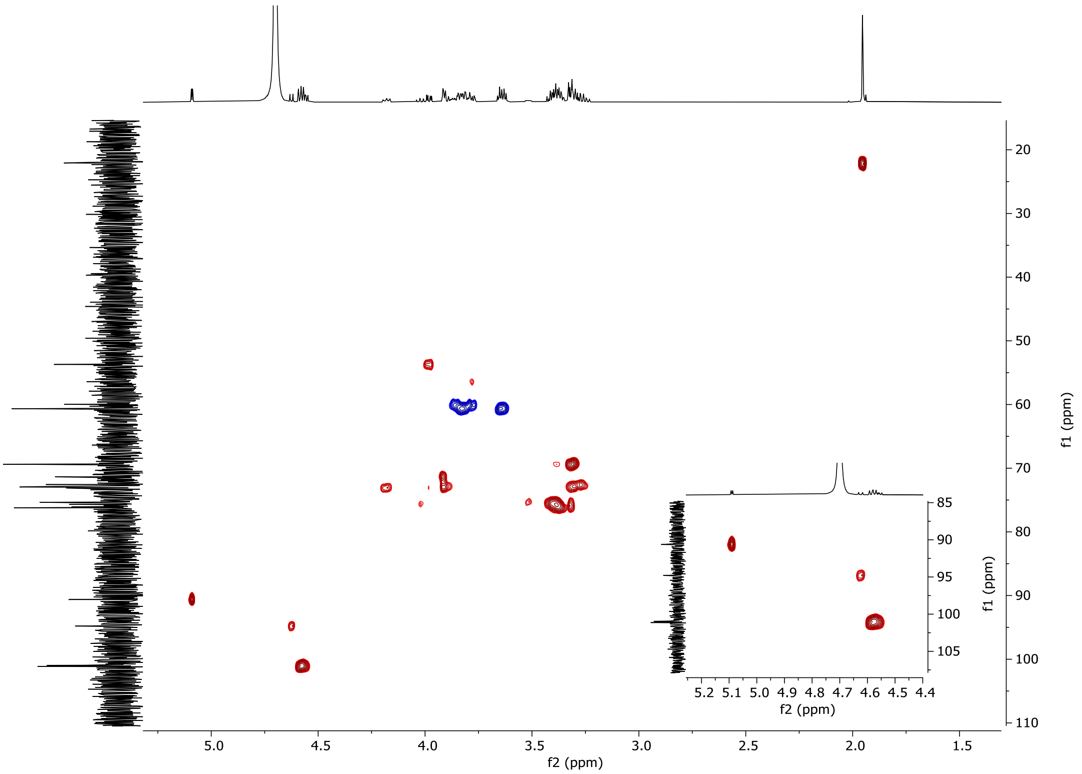

3.5.3 3mer-III

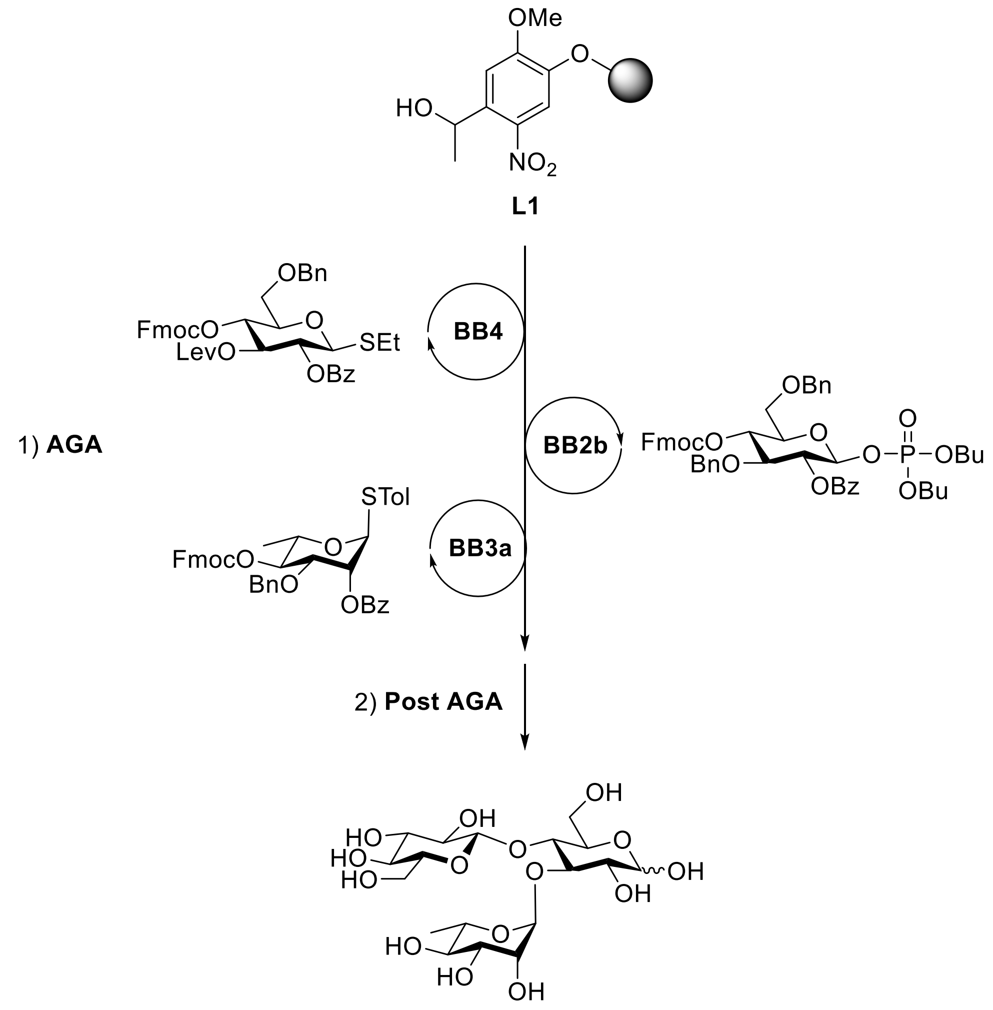

3mer-III

Step	BB	Modules	Notes
AGA	-	**A**	**L1** swelling
	**BB4**	**B**, **C1**, **D**, **E1**	**C1**: (**BB4**, -20 °C for 5 min, 0 °C for 20 min)
	**BB2b**	**B**, **C1***, **D**, **E1**,	**C1***: (**BB2b**, -30 °C for 5 min, -10 °C for 40 min) *Double cycle
	-	**D**, **E2**	-
	**BB3a**	**B**, **C1**, **D**, **E1**	**C1**: (**BB3a**, -20 °C for 5 min, 0 °C for 20 min)
Post-AGA	-	**F**, **G1**, **H2**, **I**	**F**: (4 h)<br>**H2**: (3 h)<br>**I**: (Method A2: 18.1 min)

Automated synthesis, global deprotection, and purification afforded **3mer-III** as a white solid (1.7 mg, 27% overall yield).

^{1}H NMR (400 MHz, D_2O) δ 5.27 (d, *J* = 1.6 Hz, 0.6H, H-1 Rha), 5.22 (d, *J* = 1.5 Hz, 0.4H, H-1 Rha), 5.20 (d, *J* = 3.8 Hz, 0.4H, H-1α Glc), 4.67 (d, *J* = 8.0 Hz, 0.6H, H-1β Glc), 4.50 (d, *J* = 7.9 Hz, 1H, H-1 Glc), 4.42 (dt, *J* = 13.5, 6.5 Hz, 1H, H-5 Rha), 4.07 – 4.03 (m, 1H), 3.96 (t, *J* = 11.1 Hz, 2.4H), 3.91 – 3.74 (m, 5H), 3.69 (dd, *J* = 9.9, 3.8 Hz, 0.4H), 3.59 (s, 0.6H), 3.55 – 3.33 (m, 4.6H), 3.25 (t, *J* = 8.7 Hz, 1H), 1.26 (d, *J* = 5.6 Hz, 3H, CH_3-6 Rha). ^{13}C NMR (101 MHz, D_2O) δ 101.49 (C-1 Glc), 100.75 (C-1 Rha), 100.62 (C-1 Rha), 95.75 (C-1α Glc), 92.01 (C-1β Glc), 78.30, 76.13, 75.94, 75.48, 75.30, 75.26, 73.57, 72.89, 72.85, 72.43, 71.89, 70.90, 70.10, 69.95, 69.66, 68.39 (C-5 Rha), 61.07, 59.56, 16.45 (CH_3-6 Rha). ESI-HRMS m/z 511.1666 $[M+Na]^+$ ($C_{18}H_{32}O_{15}Na$ requires 511.1633).

RP-HPLC of crude 3mer-III (ELSD trace, Method A1)

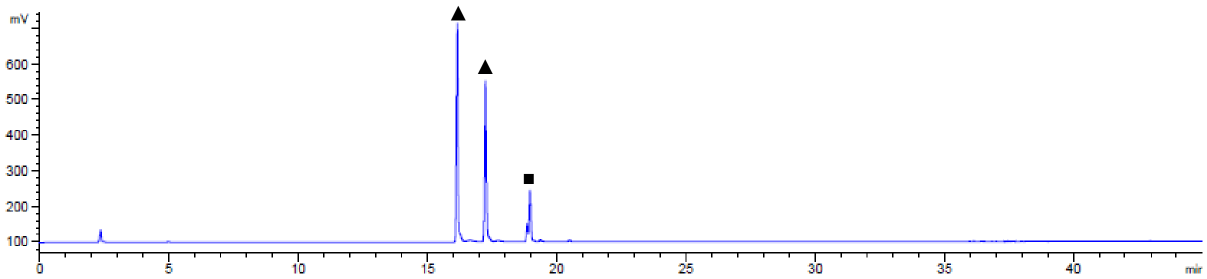

▲ **3mer-III** (α and β anomers, m/z 568 $[M+Na^+]$). ■ 2mer deletion sideproduct (-Rha, m/z 365 $[M+Na]^+$).

RP-HPLC of 3mer-III (ELSD trace, Method A1, t_R = 16.2, 17.3 min)

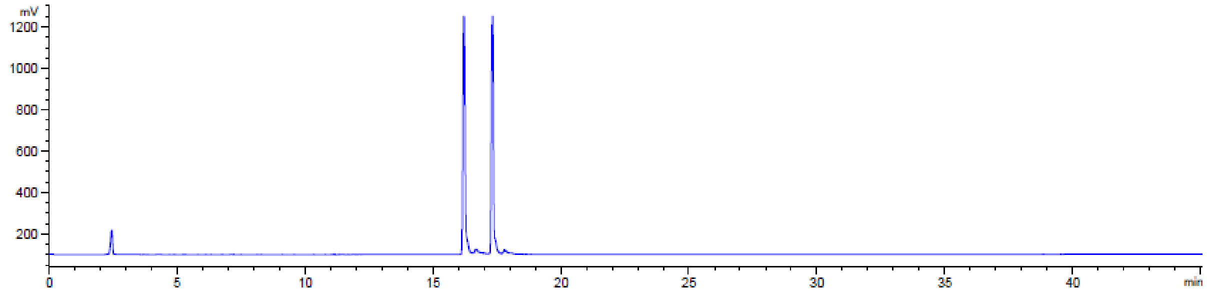

^{1}H NMR of 3mer-III (400 MHz, D_2O)

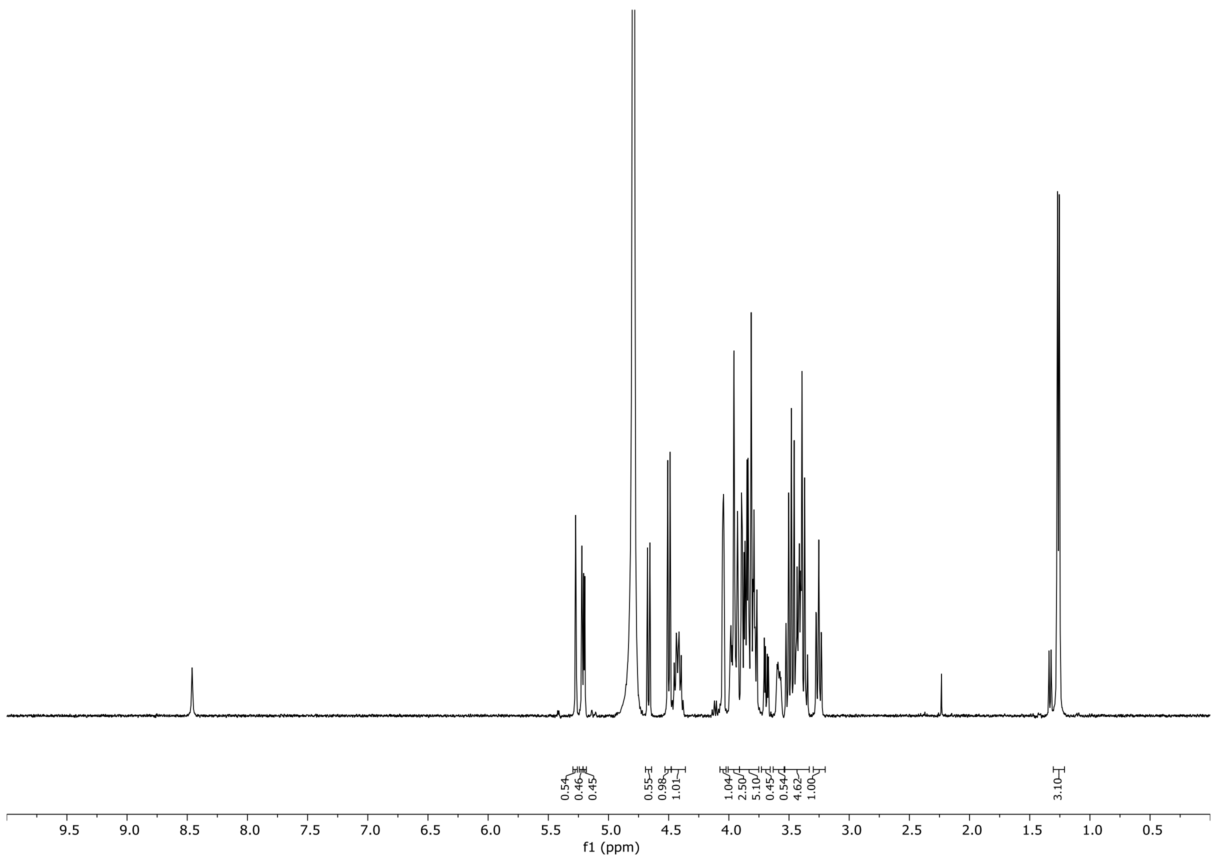

^{13}C NMR of 3mer-III (151 MHz, D_2O)

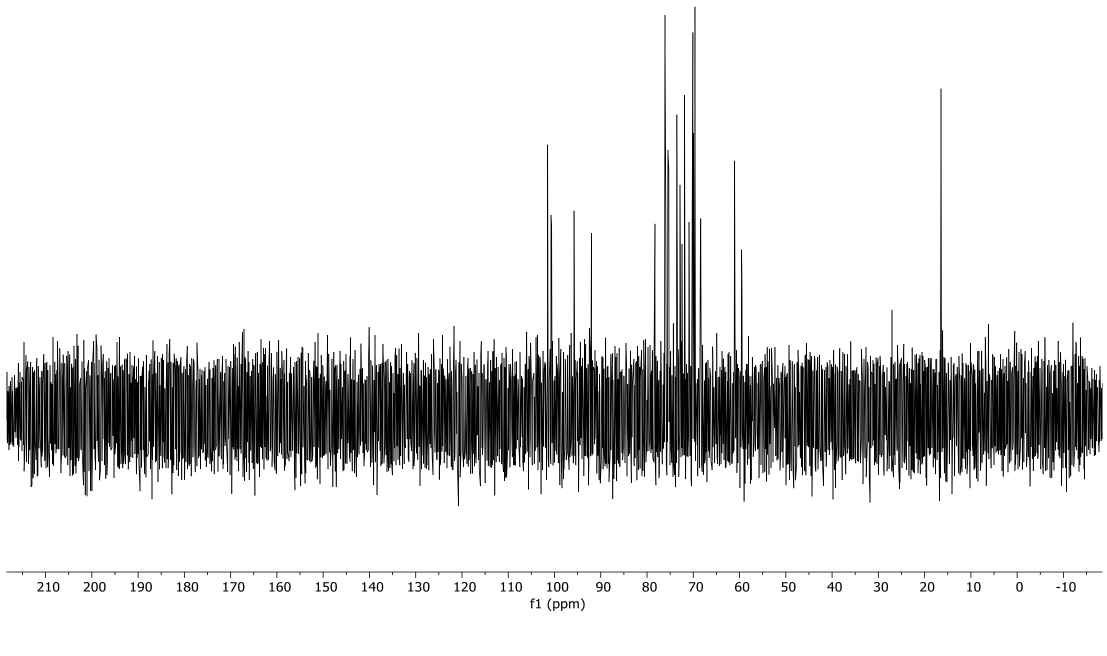

COSY NMR of 3mer-III (D_2O)

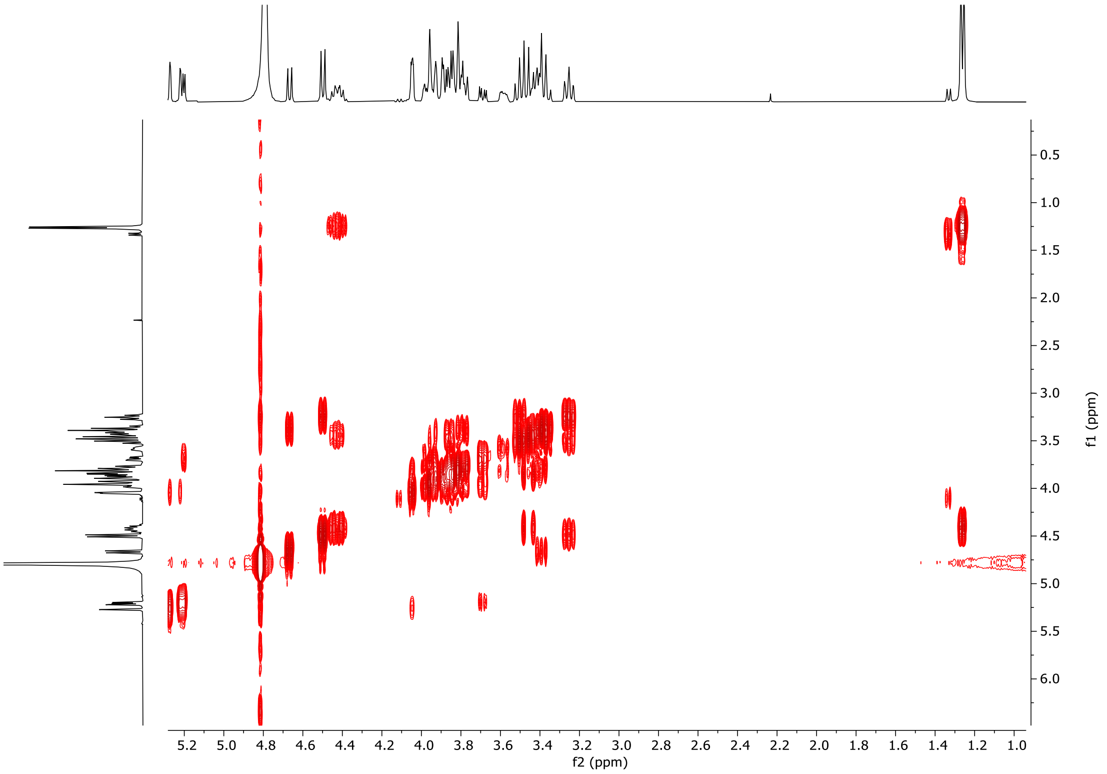

HSQC NMR of 3mer-III (D_2O)

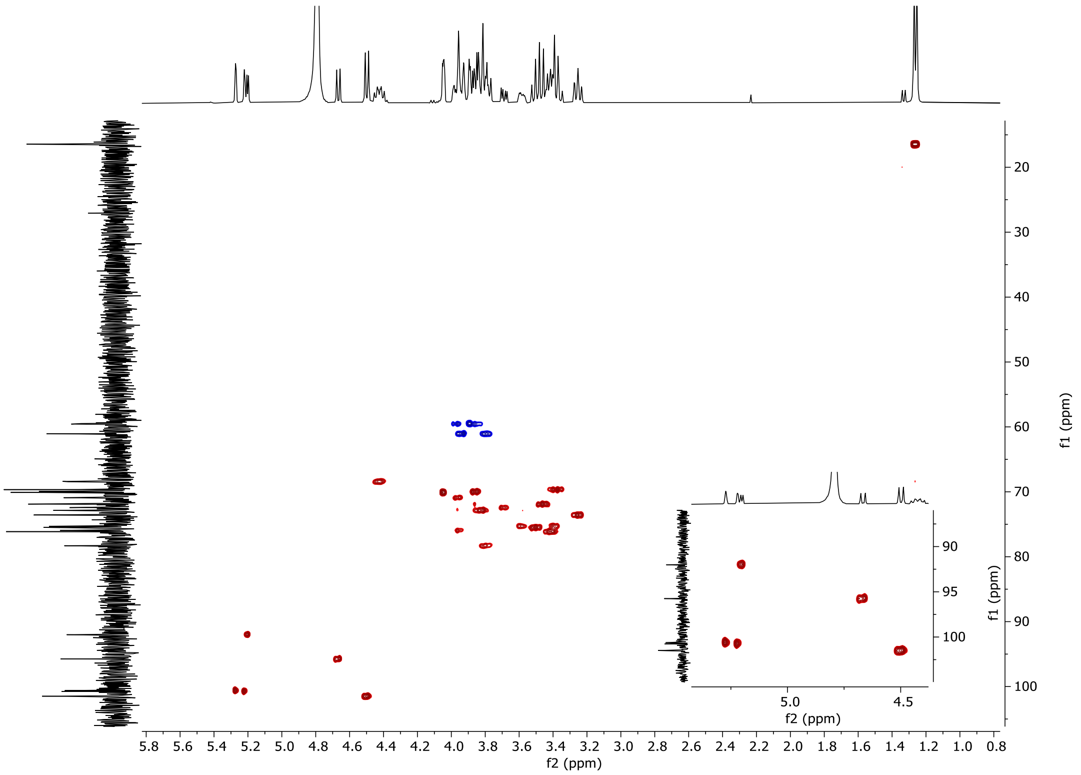

3.5.4 3mer-IV

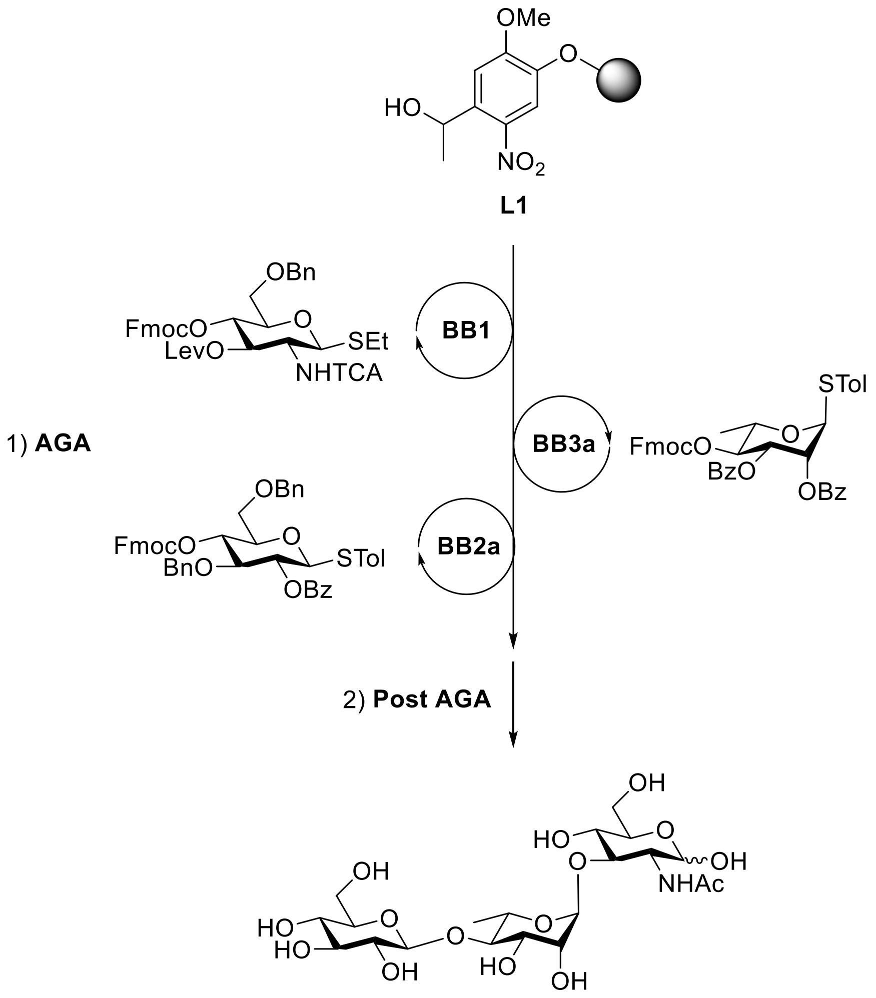

3mer-IV

Step	BB	Modules	Notes
AGA	-	**A**	**L1** swelling
	**BB1**	**B**, **C1***, **D**, **E1**	**C1***: (**BB1**, -20 °C for 5 min, 0 °C for 40 min)<br>*Double cycle
	-	**D**, **E2**	-
	**BB3a**	**B**, **C1**, **D**, **E1**	**C1**: (**BB3a**, -20 °C for 5 min, 0 °C for 20 min)
	**BB2a**	**B**, **C1**, **D**, **E1**	**C1**: (**BB2a**, -20 °C for 5 min, 0 °C for 20 min)
Post-AGA	-	**F**, **G1**, **H2**, **I**	**F**: (24 h)<br>**H2**: (4 h)<br>**I**: (Method A2: 20.5 min)

Automated synthesis, global deprotection, and purification afforded **3mer-IV** as a white solid (0.7 mg, 11% overall yield).

^{1}H NMR (600 MHz, D_2O) δ 5.14 (d, *J* = 3.5 Hz, 0.6H, H-1α GlcNAc), 4.87 – 4.82 (m, 1H, H-1 Rha), 4.75 (d, *J* = 8.7 Hz, 0.4H, H-1β GlcNAc), 4.71 (d, *J* = 8.0 Hz, 1H, H-1 Glc), 4.06 (dt, *J* = 9.8, 6.4 Hz, 1H, H-5 Rha), 4.02 (dd, *J* = 10.5, 3.5 Hz, 0.6H), 3.95 (dd, *J* = 9.5, 3.3 Hz, 1H), 3.93 – 3.70 (m, 6.4H), 3.67 (t, *J* = 9.6 Hz, 1H), 3.61 – 3.42 (m, 4H), 3.41 – 3.36 (m, 1H), 3.33 – 3.27 (m, 1H), 2.06 (s, 3H, CH_3 Ac GlcNAc), 1.31 – 1.27 (m, 3H, CH_3-6 Rha). ^{13}C NMR (151 MHz, D_2O) δ 103.21 (C-1 Glc), 101.08 (C-1 Rha), 94.16 (C-1β GlcNAc), 90.94 (C-1α GlcNAc), 81.76, 80.99, 79.31, 75.90, 75.69, 73.86, 71.74, 70.73, 70.30, 69.47, 68.37, 67.35 (C-5 Rha), 60.53, 56.28, 53.55, 21.88 (CH_3 Ac GlcNAc), 16.63 (C-6 Rha). ESI-HRMS m/z 552.1901 $[M+Na]^+$ ($C_{20}H_{35}NO_{15}Na$ requires 552.1899).

RP-HPLC of crude 3mer-IV (ELSD trace, Method A1)

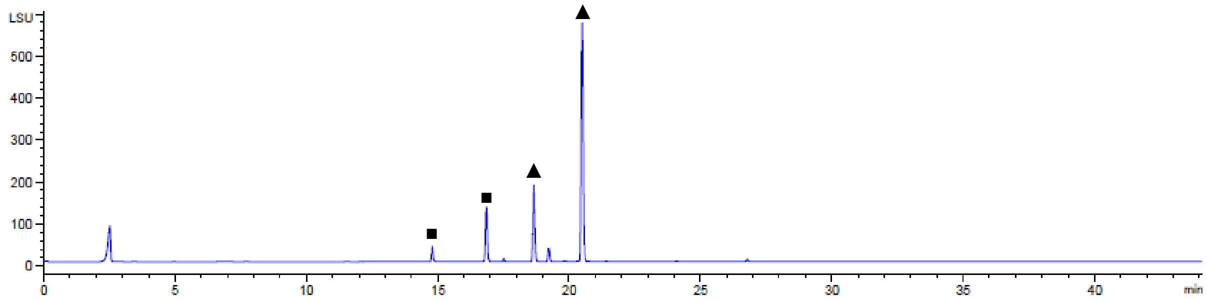

▲**3mer-IV** (α and β anomers, m/z 552 $[M+Na]^+$). ■ 2mer deletion sideproduct (-Glc, m/z 390 $[M+Na]^+$).

RP-HPLC of 3mer-IV (ELSD trace, Method A1, t_R = 19.9, 20.5 min)

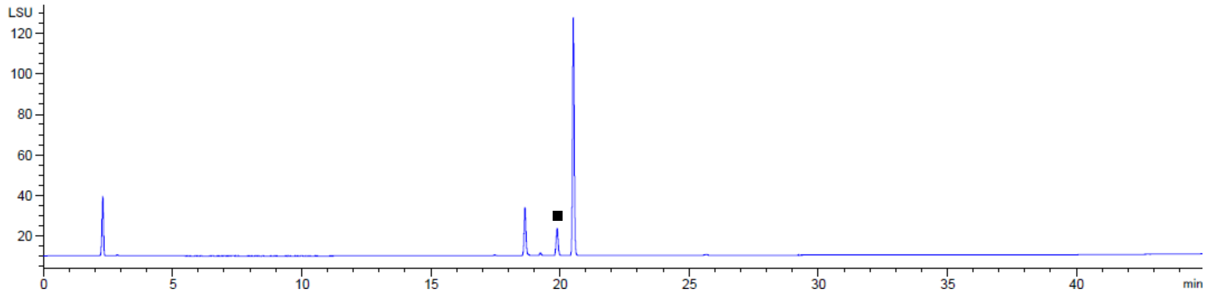

■ Unidentified impurity.

^{1}H NMR of 3mer-IV (600 MHz, D_2O)

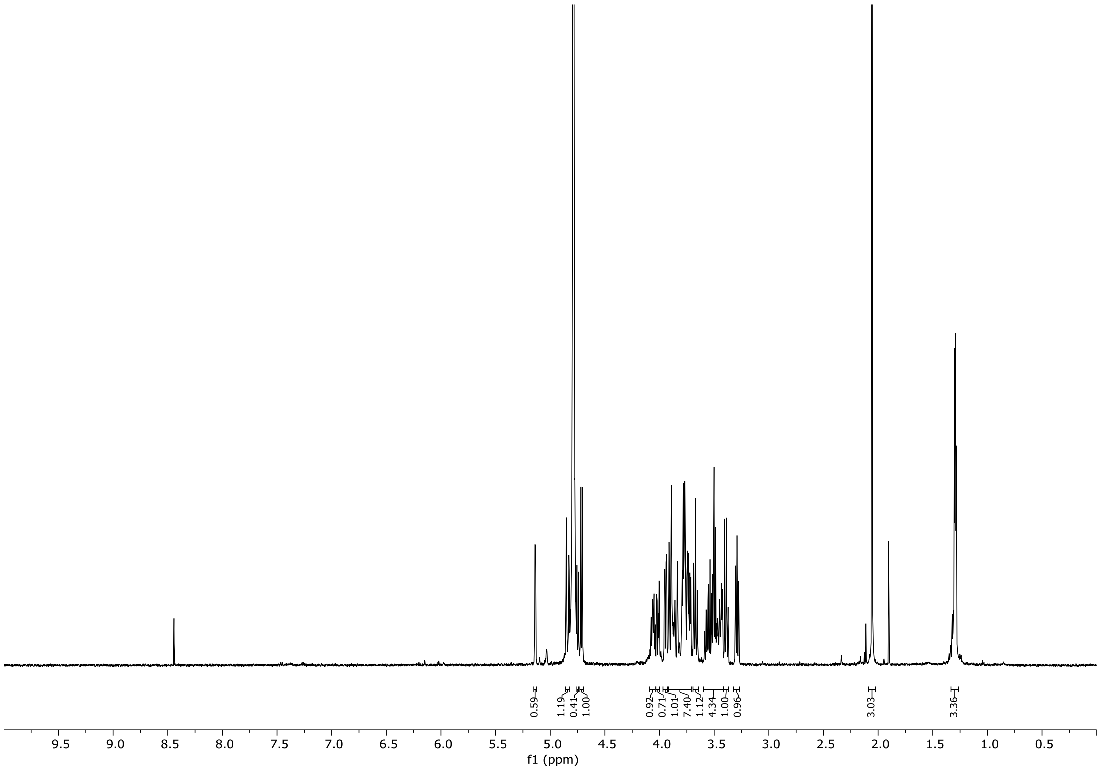

^{13}C NMR of 3mer-IV (151 MHz, D_2O)

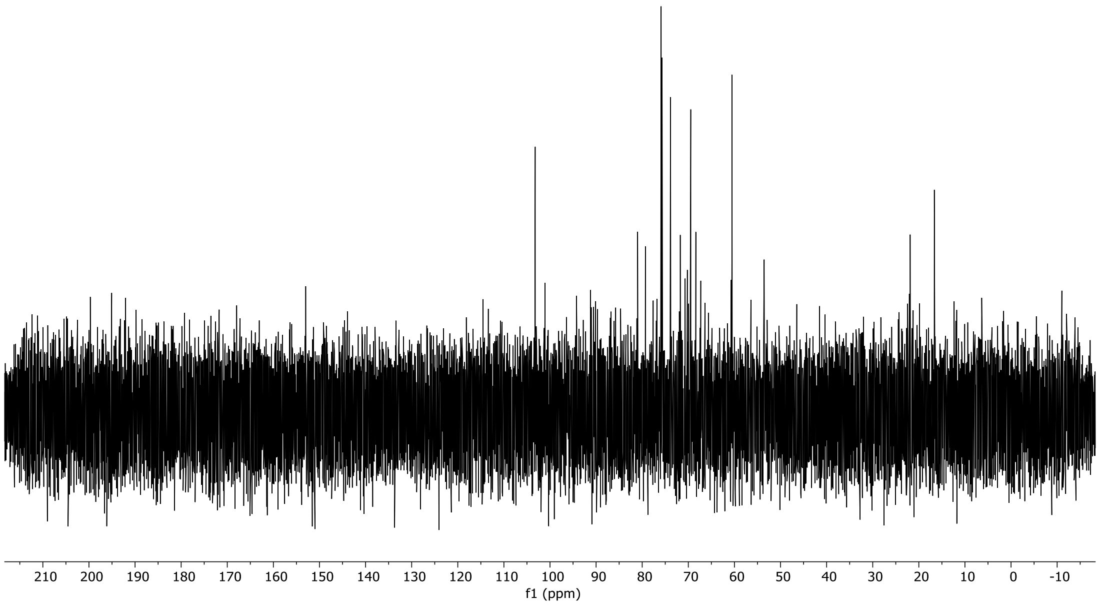

HSQC NMR of 3mer-IV (D_2O)

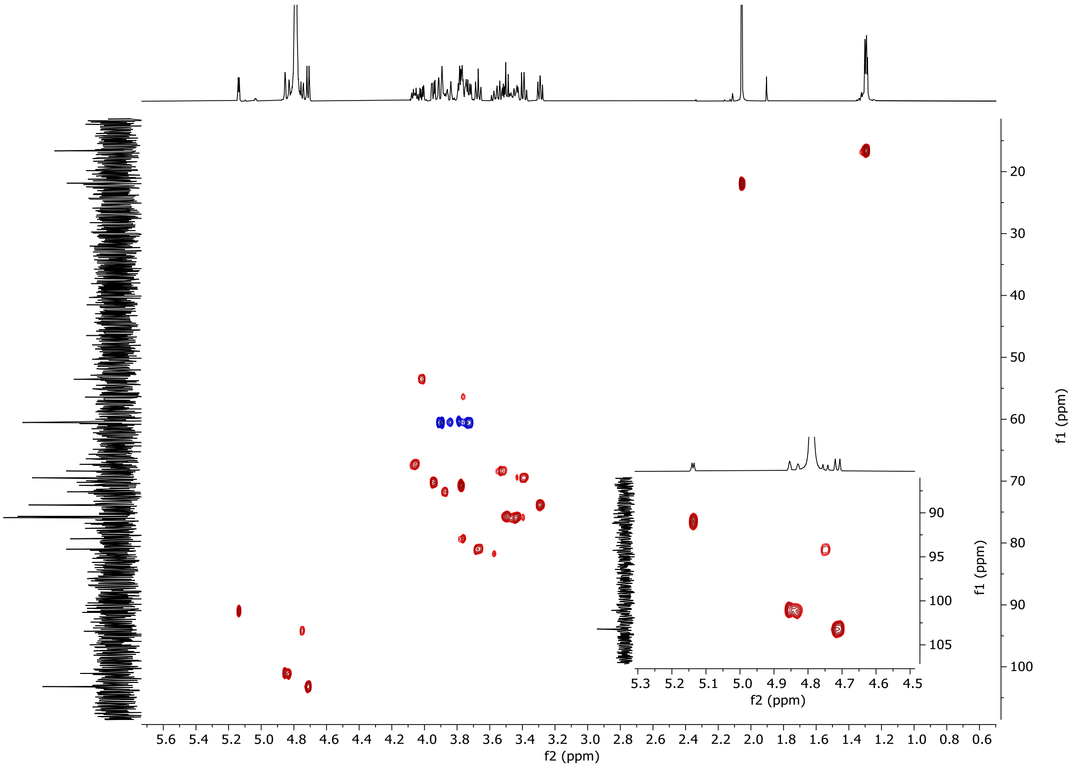

3.5.5 5mer-I

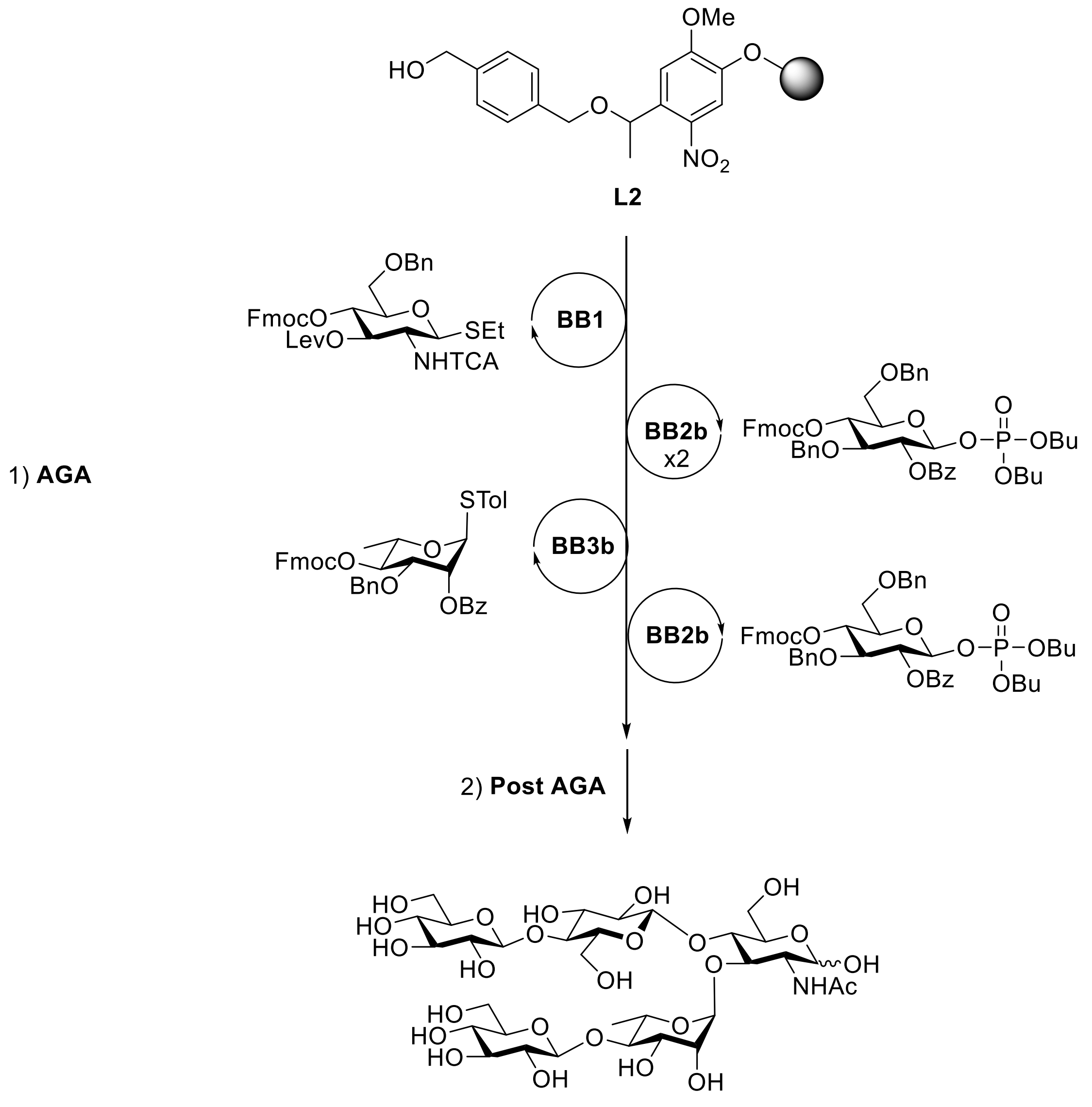

5mer-I

Step	BB	Modules	Notes
AGA	-	**A**	**L2** swelling
	**BB1**	**B**, **C1***, **D**, **E1**	**C1***: (**BB1**, -20 °C for 5 min, 0 °C for 40 min) *Double cycle
	**BB2b**	**B**, **C2***, **D**, **E1**	**C2***: (**BB2b**, -30 °C for 5 min, -10 °C for 40 min) *Double cycle
	**BB2b**	**B**, **C2**, **D**, **E1**	**C2**: (**BB2b**, -30 °C for 5 min, -10 °C for 40 min)
	-	**D**, **E2**	-
	**BB3b**	**B**, **C1**, **D**, **E1**	**C1**: (**BB3b**, -20 °C for 5 min, 0 °C for 20 min)
	**BB2b**	**B**, **C2**, **D**, **E1**	**C2**: (**BB2b**, -30 °C for 5 min, -10 °C for 40 min)
Post-AGA	-	**F, G1, H2, I**	**F**: (48 h)<br>**H2**: (3 h)<br>**I**: (Method A2: 23.1 min)

Automated synthesis, global deprotection, and purification afforded **5mer-I** as a white solid (2.1 mg, 19% overall yield).

^{1}H NMR (600 MHz, D_2O) δ 5.10 (d, *J* = 3.5 Hz, 0.6H, H-1α GlcNAc), 4.93 – 4.90 (m, 1H, H-1 Rha), 4.73 (d, *J* = 2.9 Hz, 0.4H, H-1β GlcNAc), 4.65 (d, *J* = 7.9 Hz, 1H, H-1 Glc), 4.54 – 4.51 (m, 1H, H-1 Glc), 4.48 – 4.41 (m, 1H, H-5 Rha), 4.41 (d, *J* = 7.9 Hz, 1H, H-1 Glc), 4.10 (dd, *J* = 10.2, 3.6 Hz, 0.6H), 4.06 – 4.02 (m, 1H), 4.02 – 3.94 (m, 2.4H), 3.94 – 3.85 (m, 4.4H), 3.84 – 3.76 (m, 3H), 3.74 – 3.67 (m, 2H), 3.65 – 3.59 (m, 2H), 3.58 – 3.53 (m, 1.6H), 3.52 – 3.47 (m, 3H), 3.46 – 3.42 (m, 1H), 3.42 – 3.37 (m, 2H), 3.37 – 3.33 (m, 1H), 3.32 – 3.23 (m, 3H), 2.04 – 2.02 (m, 3H, CH_3 Ac GlcNAc), 1.31 – 1.27 (m, 3H, CH_3-6 Rha). ^{13}C NMR (151 MHz, D_2O) δ 103.66 (C-1 Glc), 102.86 (C-1 Glc), 101.04 (C-1 Glc), 100.15 (C-1 Rha), 94.26 (C-1β GlcNAc), 90.97 (C-1α GlcNAc), 81.29, 80.09, 75.99, 75.89, 75.31, 75.13, 74.53, 74.29, 74.27, 74.22, 73.15, 73.10, 71.09, 70.37, 70.31, 70.18, 70.13, 69.60, 69.26, 67.05 (C-5 Rha), 60.93, 60.63, 60.49, 56.84, 53.90, 21.86 (CH_3 Ac GlcNAc), 16.74 (C-6 Rha). ESI-HRMS m/z 876.2943 $[M+Na]^+$ ($C_{32}H_{55}NO_{25}Na$ requires 876.2955).

RP-HPLC of crude 5mer-I (ELSD trace, Method A1)

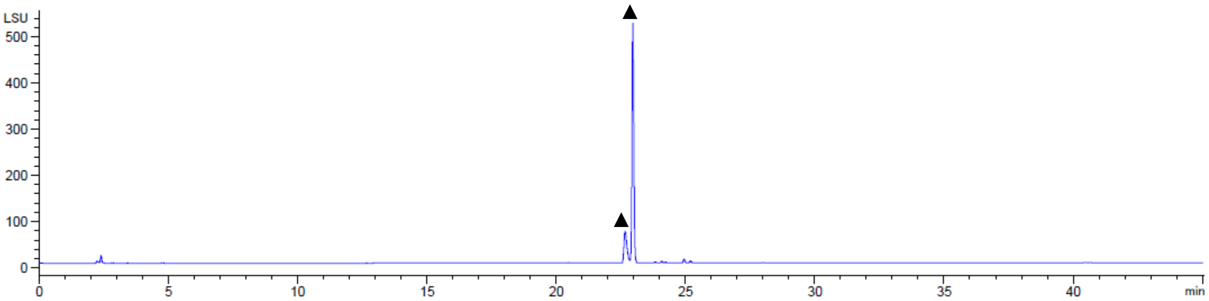

▲**5mer-I** (α and β anomers, m/z 876 $[M+Na]^+$).

RP-HPLC of 5mer-I (ELSD trace, Method A1, t_R = 22.5, 22.8 min)

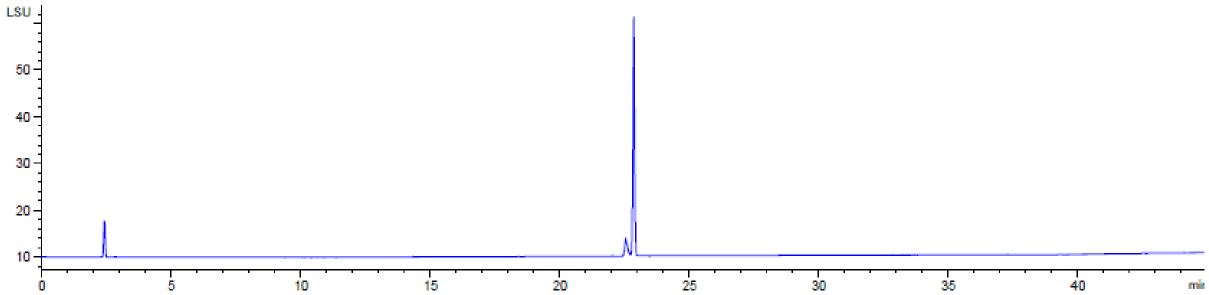

^{1}H NMR of 5mer-I (600 MHz, D_2O)

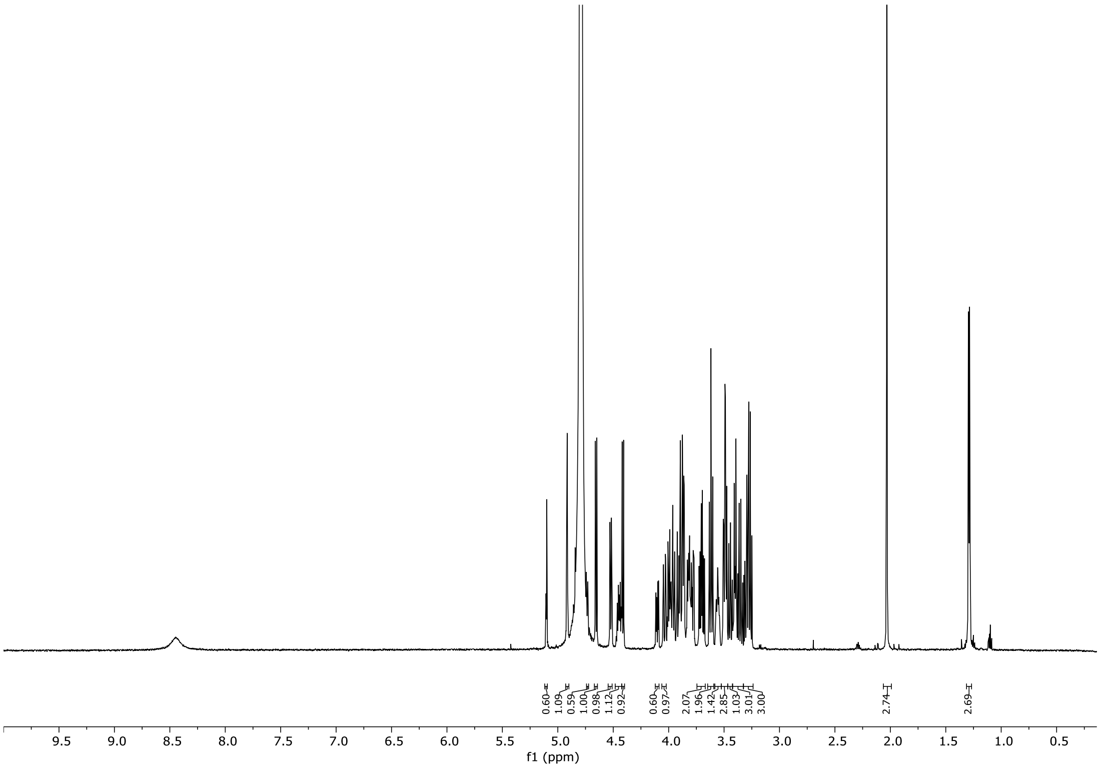

^{13}C NMR of 5mer-I (151 MHz, D_2O)

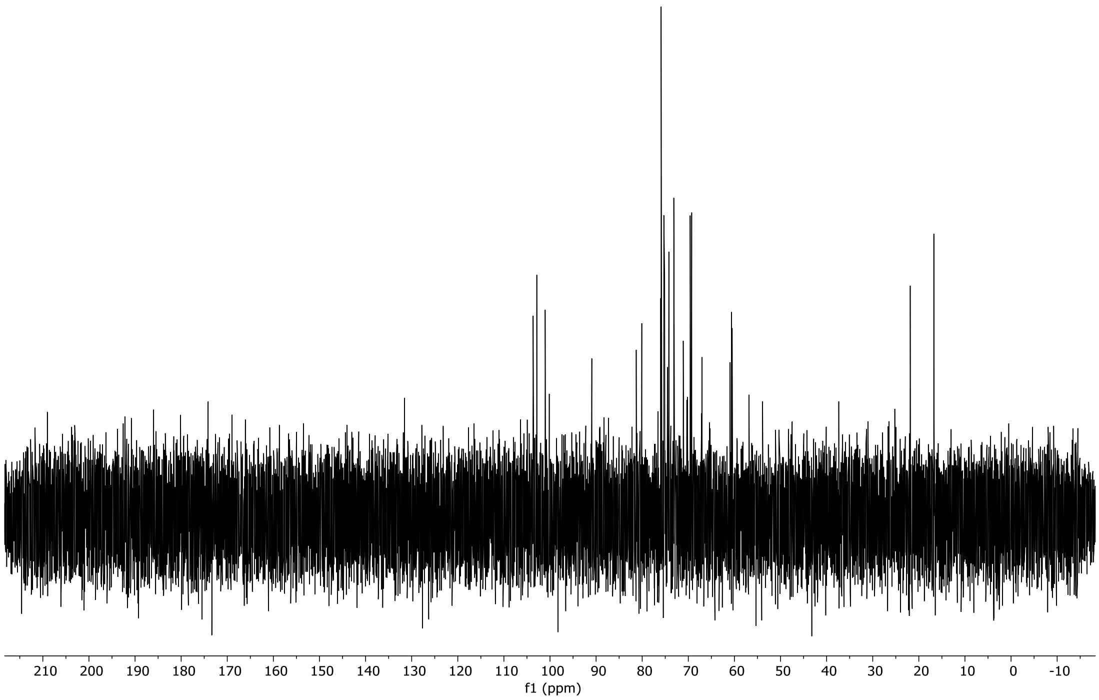

COSY NMR of 5mer-I (D_2O)

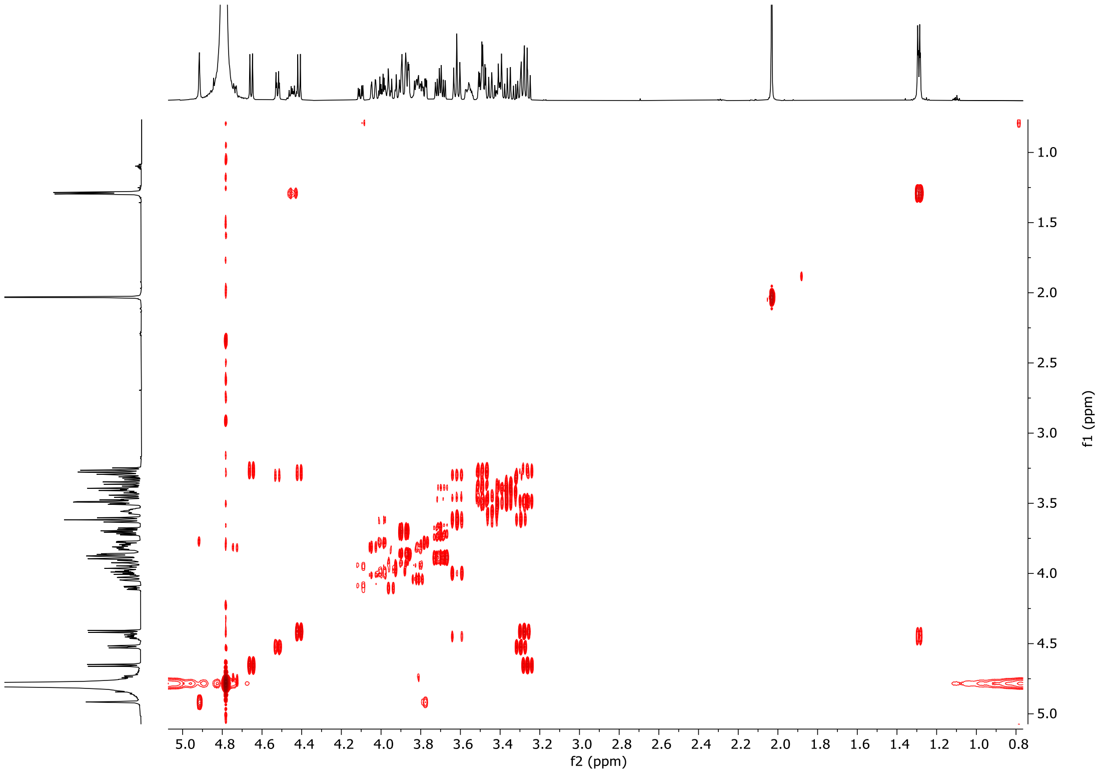

HSQC NMR of 5mer-I (D_2O)

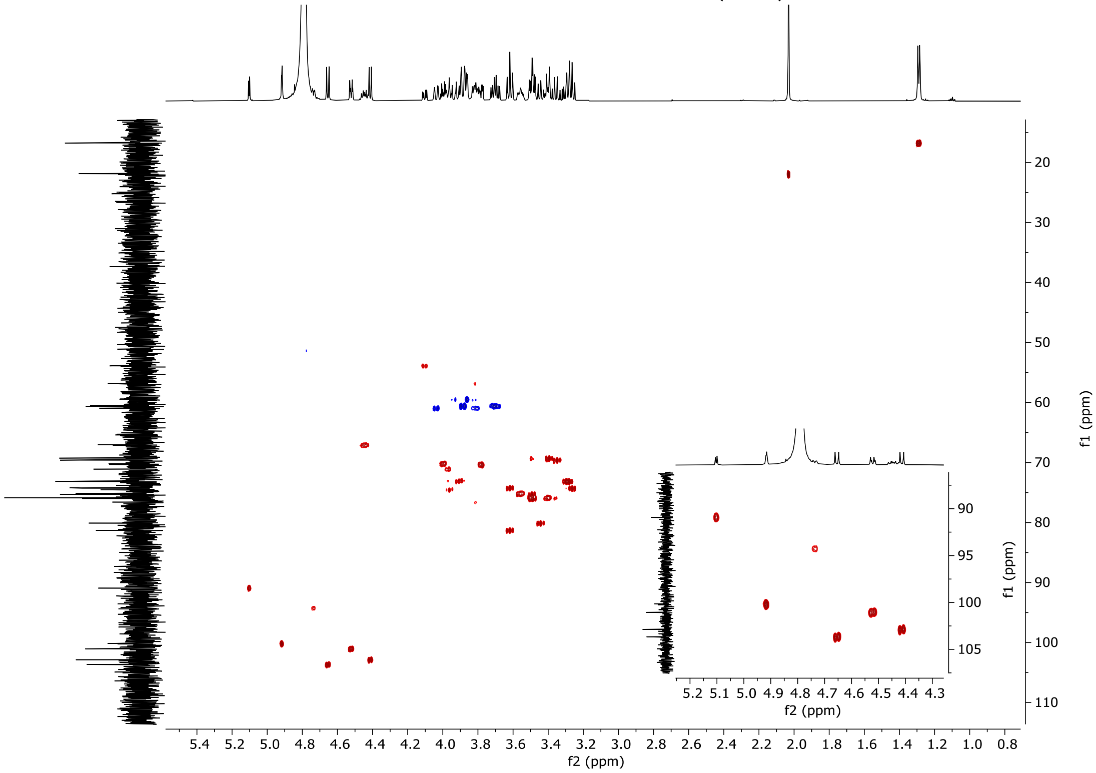

3.5.6 9mer-I

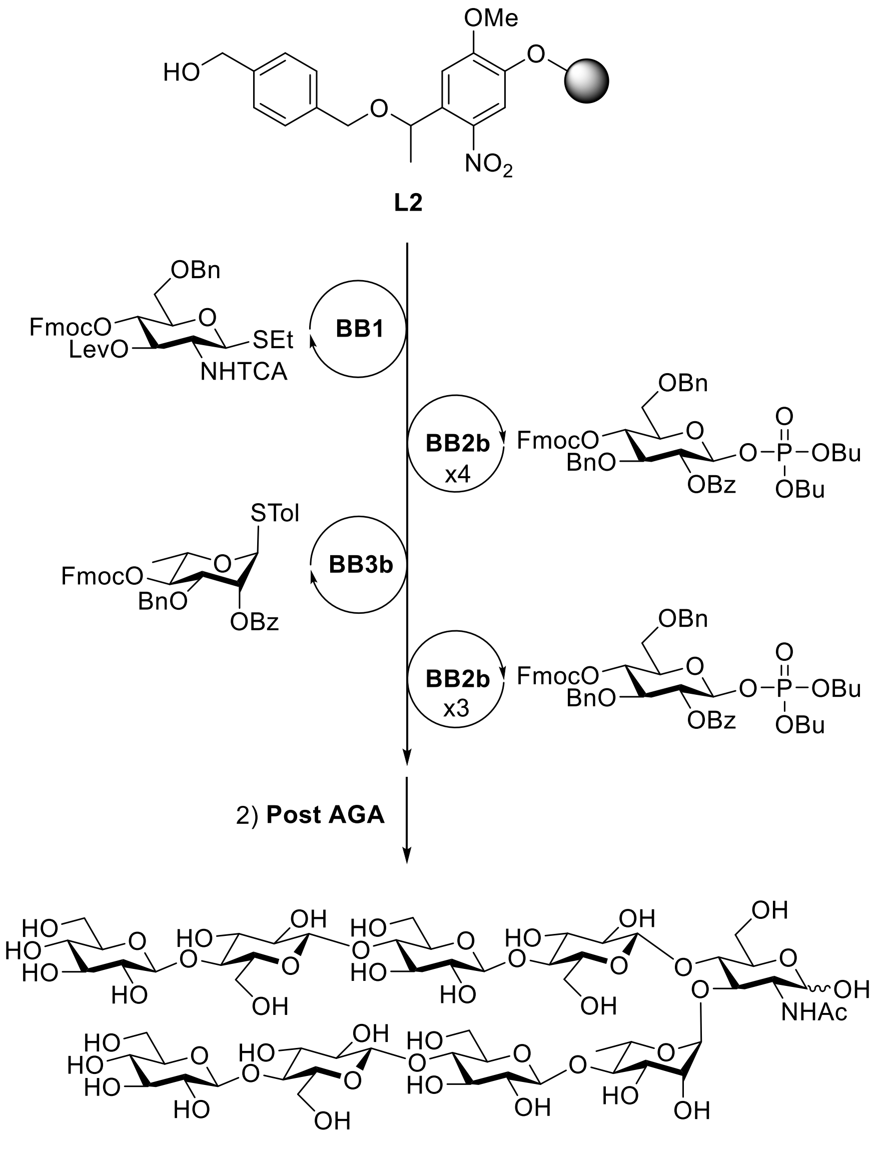

OMe
HO
O
NO2
L2
OBn
FmocO
LevO
O
SEt
NHTCA
BB1
BB2b
x4
FmocO
BnO
OBz
O-P-OBu
OBu
1) AGA
STol
FmocO
BnO
OBz
BB3b
BB2b
x3
2) Post AGA
HO
OH
NHAc
9mer-I

Step	BB	Modules	Notes
AGA	-	**A**	**L2** swelling
	**BB1**	**B**, **C1***, **D**, **E1**	**C1***: (**BB1**, -20 °C for 5 min, 0 °C for 40 min) *Double cycle
	**BB2b**	**B**, **C2***, **D**, **E1**	**C2***: (**BB2b**, -30 °C for 5 min, -10 °C for 40 min) *Double cycle
	(**BB2b**)x3	(**B**, **C2**, **D**, **E1**)x3	**C2**: (**BB2b**, -30 °C for 5 min, -10 °C for 40 min)
	-	**D**, **E2**	-
	**BB3b**	**B**, **C1**, **D**, **E1**	**C1**: (**BB3b**, -20 °C for 5 min, 0 °C for 20 min)
	(**BB2b**)x3	(**B**, **C2**, **D**, **E1**)x3	**C2**: (**BB2b**, -30 °C for 5 min, -10 °C for 40 min)
Post-AGA	-	**F**, **G1**, **H2**, **I**	**F**: (24 h)<br>**H2**: (10 h)<br>**I**: (Method B2: 26.9 min)

Automated synthesis, global deprotection, and purification afforded **9mer-I** as a white solid (2.3 mg, 12% overall yield).

^{1}H NMR (600 MHz, D_2O) δ 5.10 (d, *J* = 3.6 Hz, 0.6H, H-1α GlcNAc), 4.91 (s, 1H, H-1 Rha), 4.73 (d, *J* = 8.0 Hz, 0.4H, H-1β GlcNAc), 4.67 (d, *J* = 7.7 Hz, 1H, H-1 Glc), 4.54 – 4.45 (m, 6H, 5xH-1 Glc, H-5 Rha), 4.44 (d, *J* = 7.9 Hz, 1H, H-1 Glc), 4.12 – 4.09 (m, 0.6H), 4.05 (d, *J* = 11.2 Hz, 1H), 4.03 – 3.92 (m, 7.4H), 3.92 – 3.85 (m, 4H), 3.85 – 3.76 (m, 7H), 3.74 – 3.52 (m, 17H), 3.47 (q, *J* = 8.6 Hz, 5H), 3.39 (t, *J* = 9.1 Hz, 2H), 3.36 – 3.26 (m, 7H), 2.03 (d, *J* = 1.2 Hz, 3H, CH_3 Ac GlcNAc), 1.33 – 1.26 (m, 3H, CH_3-6 Rha). ^{13}C NMR (151 MHz, D_2O) δ 103.58 (C-1 Glc), 102.75 (C-1 Glc), 102.57 (C-1 Glc), 102.51 (C-1 Glc), 102.37 (C-1 Glc), 100.95(C-1 Glc), 100.01 (C-1 Rha), 94.17 (C-1β GlcNAc), 90.88 (C-1α GlcNAc), 81.57, 80.31, 78.76, 78.16, 75.89, 75.45, 75.37, 75.09, 74.65, 74.44, 74.25, 74.04, 73.95, 73.06, 72.98, 72.79, 69.35, 66.96 (C-5 Rha), 60.46, 59.71, 56.82, 53.93, 21.82 (CH_3 Ac GlcNAc), 16.71 (C-6 Rha). ESI-HRMS m/z 1524.5063 $[M+Na]^+$ ($C_{56}H_{95}NO_{45}Na$ requires 1524.5068).

RP-HPLC of crude 9mer-I (ELSD trace, Method B1)

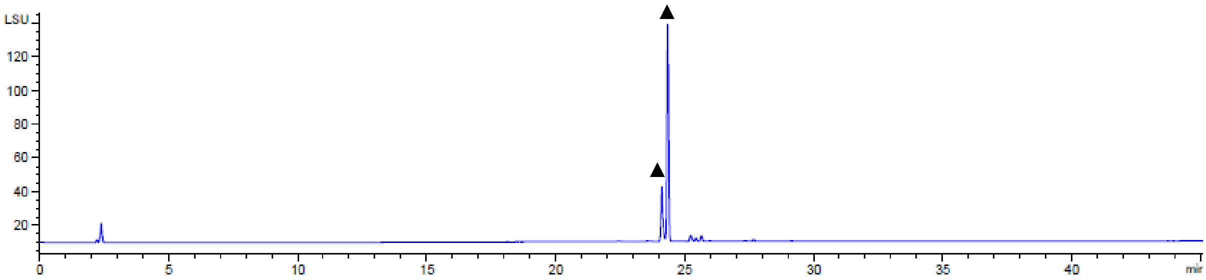

▲ **9mer-I** (α and β anomers, m/z 1524 [M+Na]$^+$).

RP-HPLC of 9mer-I (ELSD trace, Method B1, t_R = 24.0, 24.3 min)

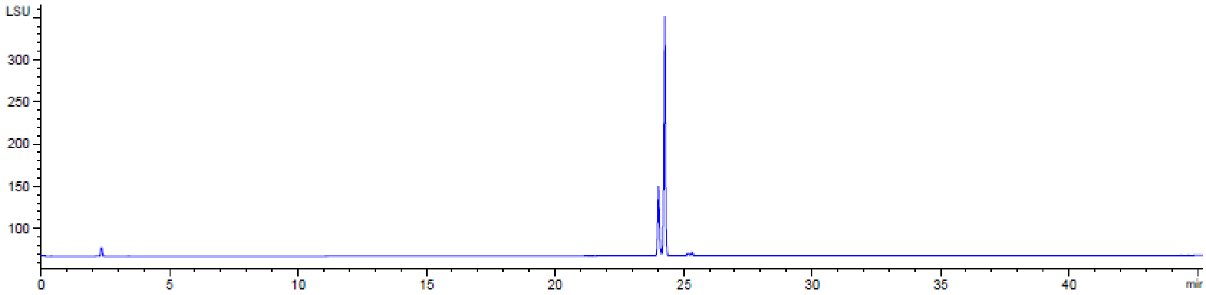

^{1}H NMR of 9mer-I (600 MHz, D_2O)

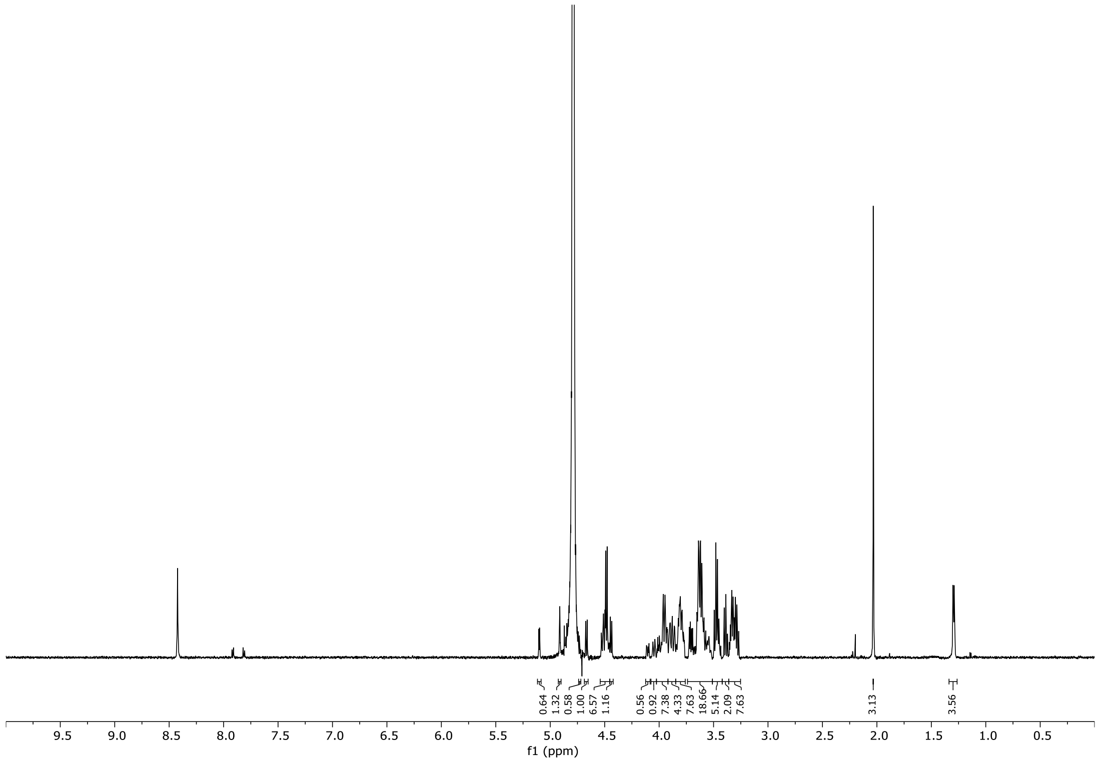

^{13}C NMR of 9mer-I (151 MHz, D_2O)

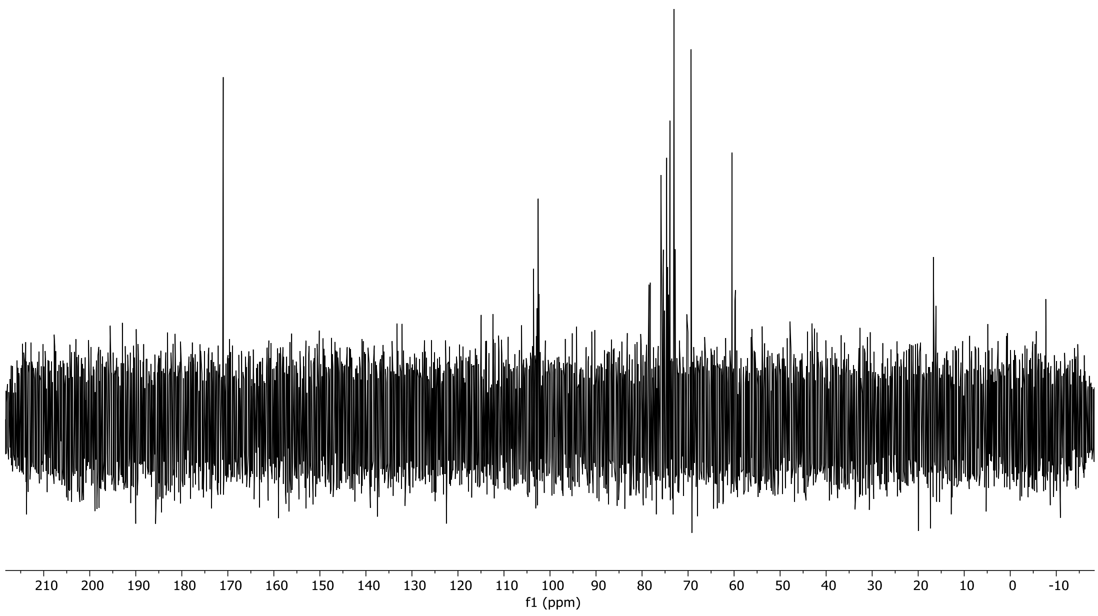

COSY NMR of 9mer-I (D_2O)

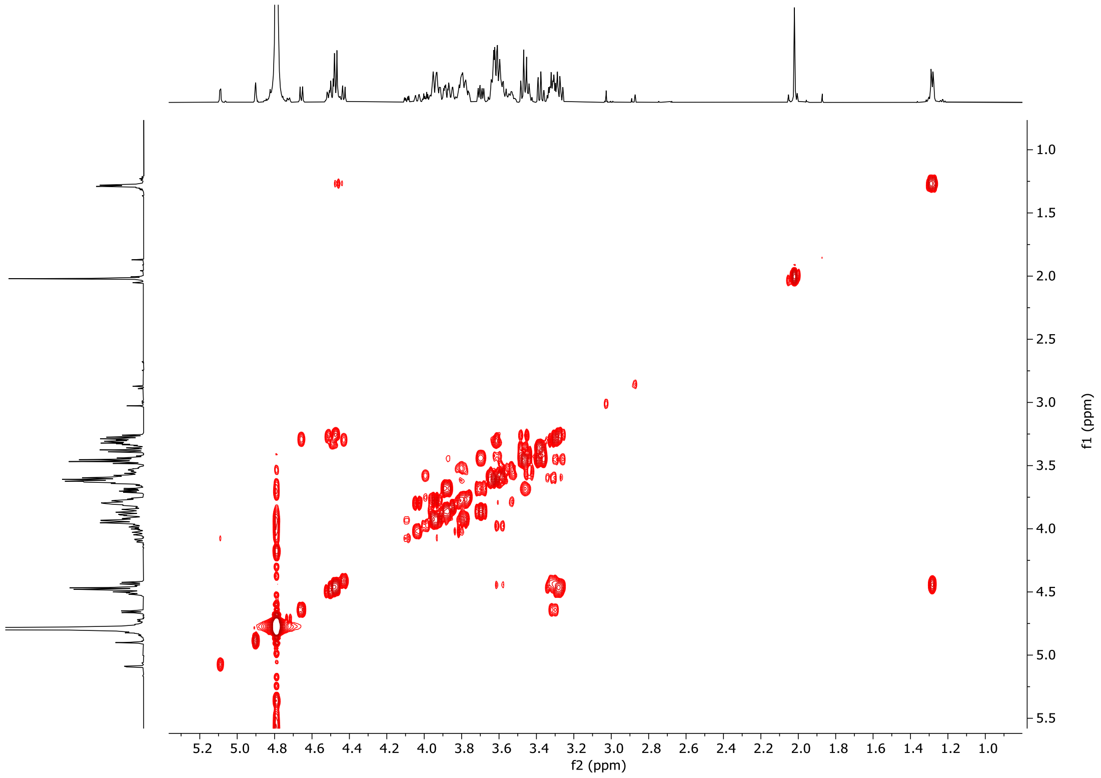

HSQC NMR of 9mer-I (D_2O)

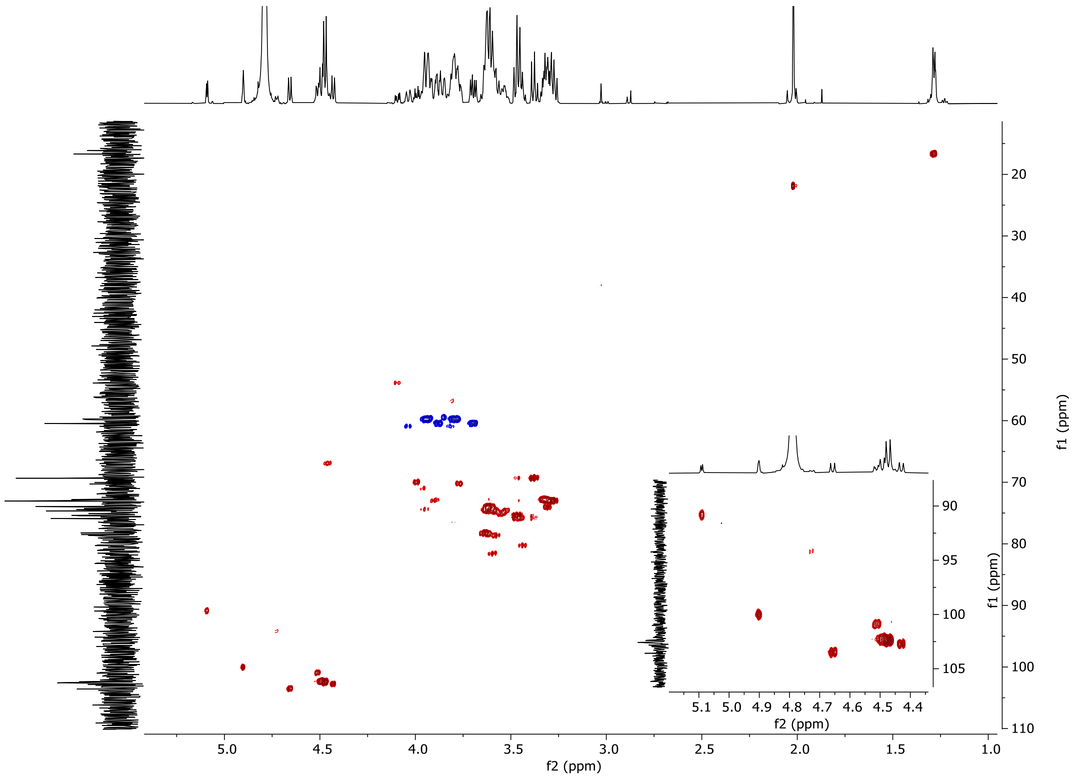

3.5.7 9mer-II

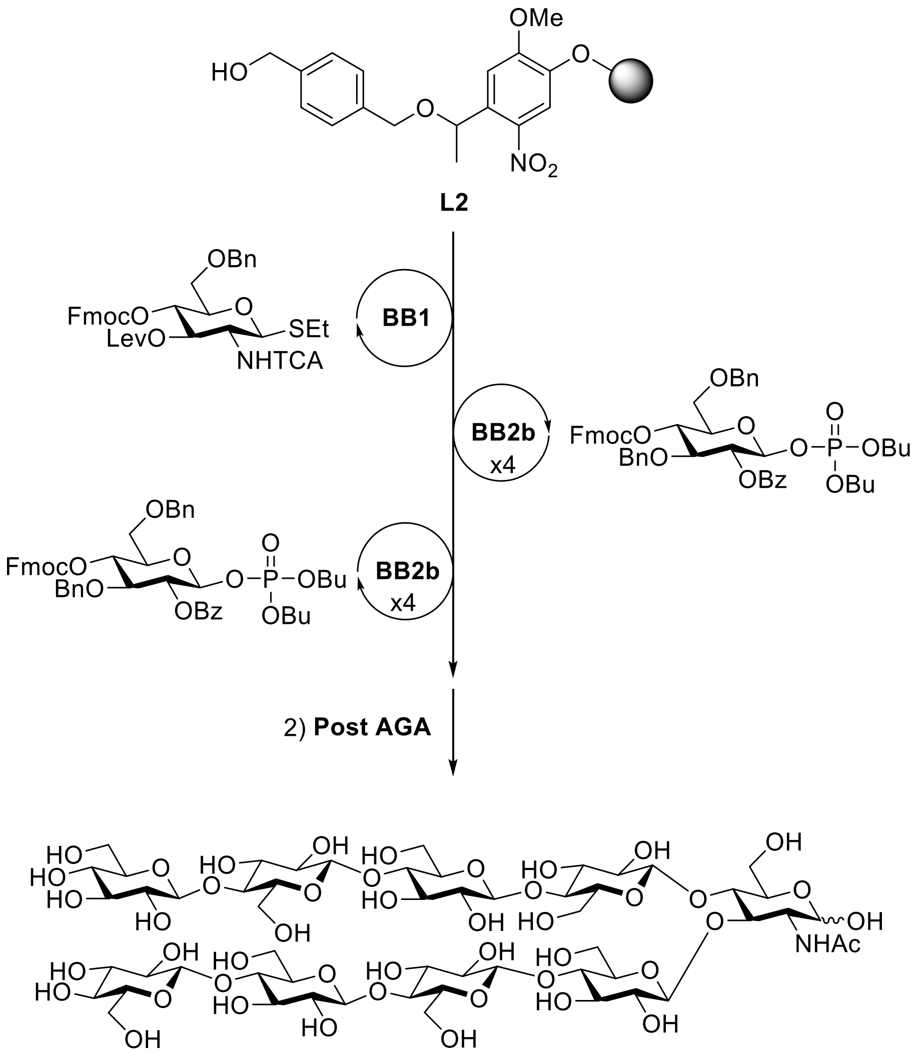

OMe
HO
O
NO2
L2
OBn
FmocO
LevO
O
SEt
NHTCA
BB1
1) AGA
BB2b
x4
OBn
FmocO
BnO
O
O
P
OBu
OBz
OBu
BB2b
x4
2) Post AGA
HO
OH
NHAc
9mer-II

Step	BB	Modules	Notes
AGA	-	**A**	**L2** swelling
	**BB1**	**B**, **C1***, **D**, **E1**	**C1***: (**BB1**, -20 °C for 5 min, 0 °C for 40 min) *Double cycle
	**BB2b**	**B**, **C2***, **D**, **E1**	**C2***: (**BB2b**, -20 °C for 5 min, 0 °C for 20 min) *Double cycle
	(**BB2b**)x3	(**B**, **C2**, **D**, **E1**)x3	**C2**: (**BB2b**, -30 °C for 5 min, -10 °C for 40 min)
	-	**D**, **E2**	-
	(**BB2b**)x4	(**B**, **C2**, **D**, **E1**)x4	**C2**: (**BB2b**, -20 °C for 5 min, 0 °C for 20 min)
Post-AGA	-	**F**, **G1**, **H2**, **I**	**F**: (18 h)<br>**H2**: (8 h)<br>**I**: (Method C2: 15.7, 16.1 min)

Automated synthesis, global deprotection, and purification afforded **9mer-II** as a white solid (3.7 mg, 18% overall yield).

^{1}H NMR (600 MHz, D_2O) δ 5.13 (d, *J* = 3.4 Hz, 0.6H, H-1α GlcNAc), 4.69 – 4.61 (m, 2.4H, H-1β GlcNAc, 2xH-1 Glc), 4.52 – 4.44 (m, 6H, 6xH-1 Glc), 4.26 – 4.18 (m, 0.6H), 4.10 – 4.02 (m, 1H), 4.01 – 3.72 (m, 17.4H), 3.69 (dd, *J* = 12.4, 5.8 Hz, 2H), 3.66 – 3.50 (m, 16H), 3.50 – 3.23 (m, 14H), 2.00 (d, *J* = 1.4 Hz, 3H, CH_3 Ac GlcNAc). ^{13}C NMR (101 MHz, D_2O) δ 174.19, 170.96, 102.46 (C-1 Glc), 102.28(C-1 Glc), 102.24(C-1 Glc), 100.93 (C-1 Glc), 100.66 (C-1 Glc), 94.58 (C-1β GlcNAc), 90.59 (C-1α GlcNAc), 78.20, 78.08, 75.87, 75.34, 74.71, 73.90, 73.02, 72.81, 69.32, 60.44, 59.74, 53.63, 21.93 (CH_3 Ac GlcNAc). ESI-HRMS m/z 1540.5099 $[M+Na]^+$ ($C_{56}H_{95}NO_{46}Na$ requires 1540.5017).

RP-HPLC of crude 9mer-II (ELSD trace, Method C1)

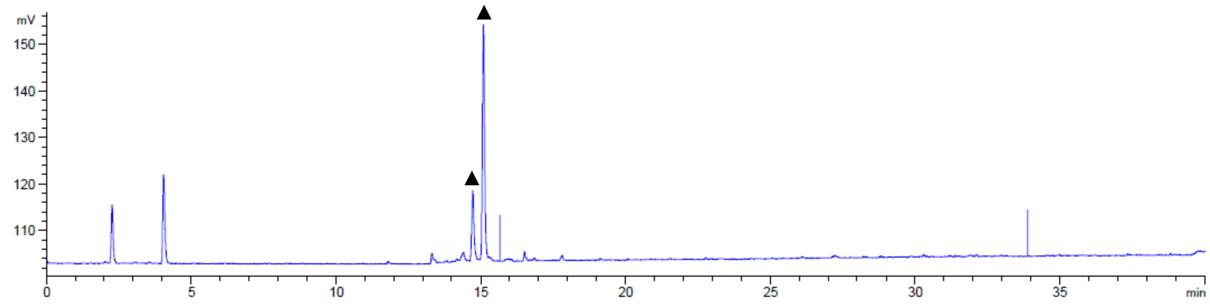

▲ **9mer-II** (α and β anomers, m/z 1540 [M+Na]$^+$).

RP-HPLC of 9mer-II (ELSD trace, Method C1, t_R = 14.9, 15.1 min)

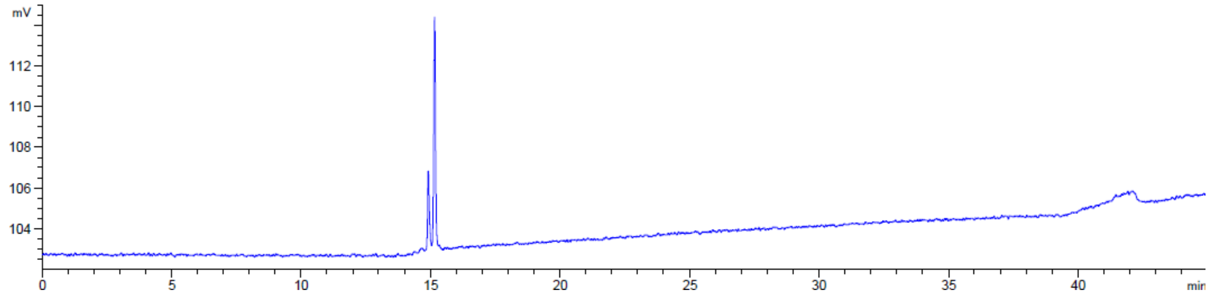

^1H NMR of 9mer-II (600 MHz, D_2O)

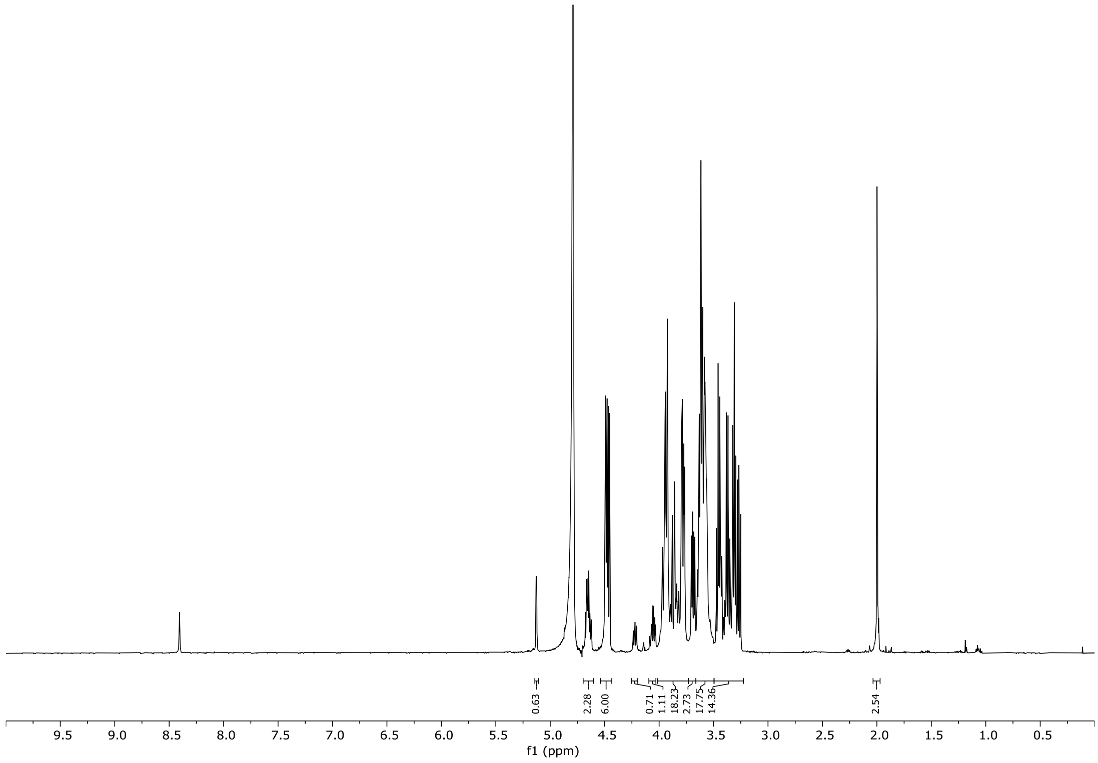

^{13}C NMR of 9mer-II (151 MHz, D_2O)

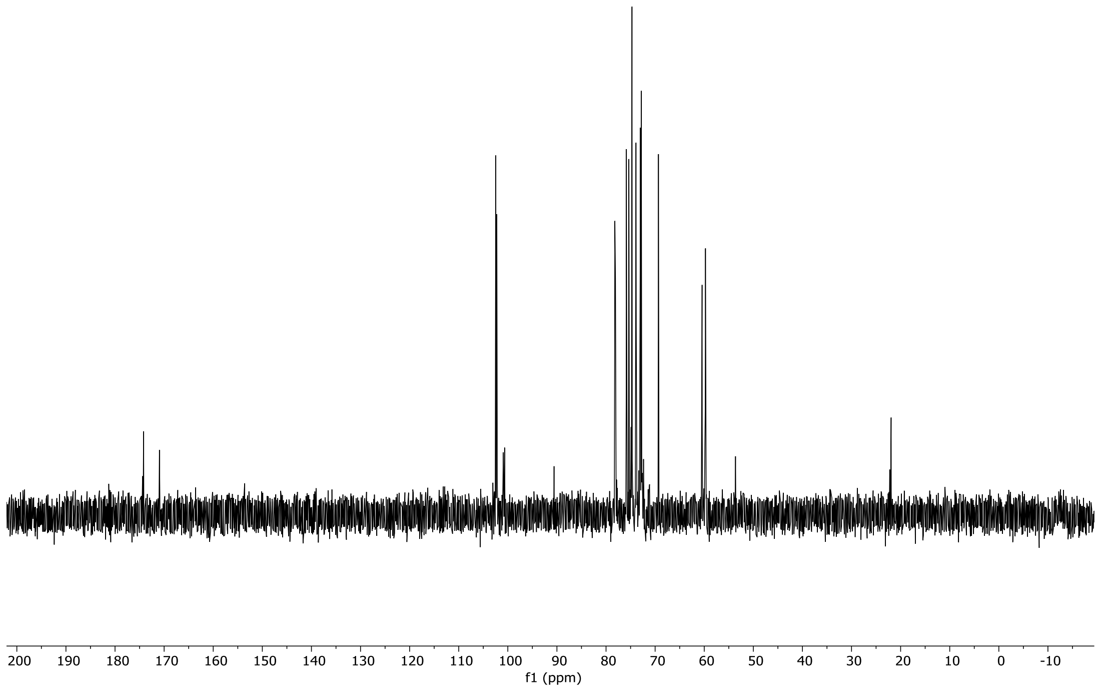

COSY NMR of 9mer-II (D_2O)

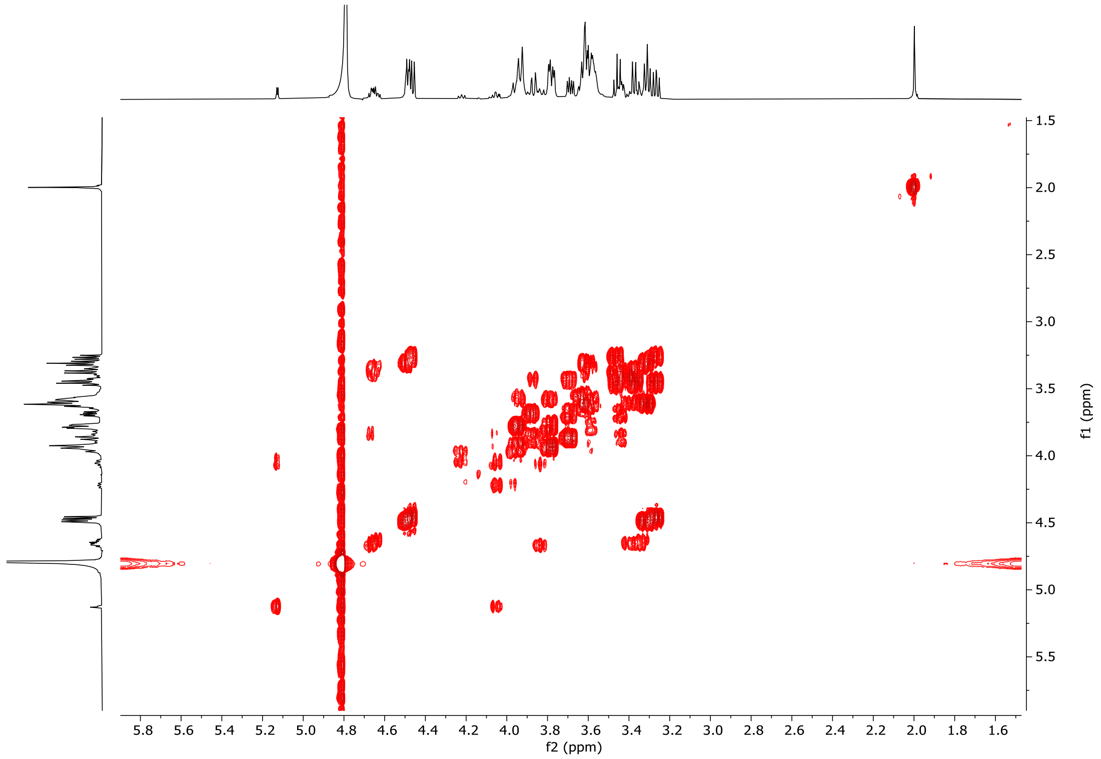

HSQC NMR of 9mer-II (D_2O)

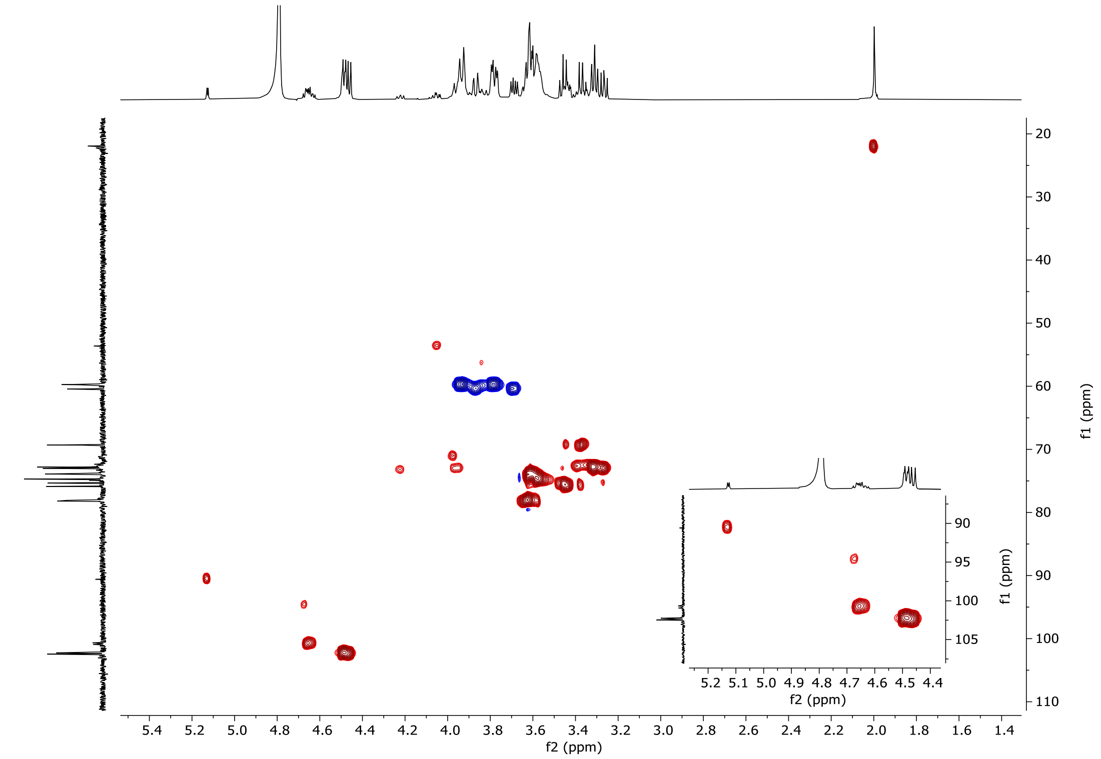

3.5.8 9mer-I-B

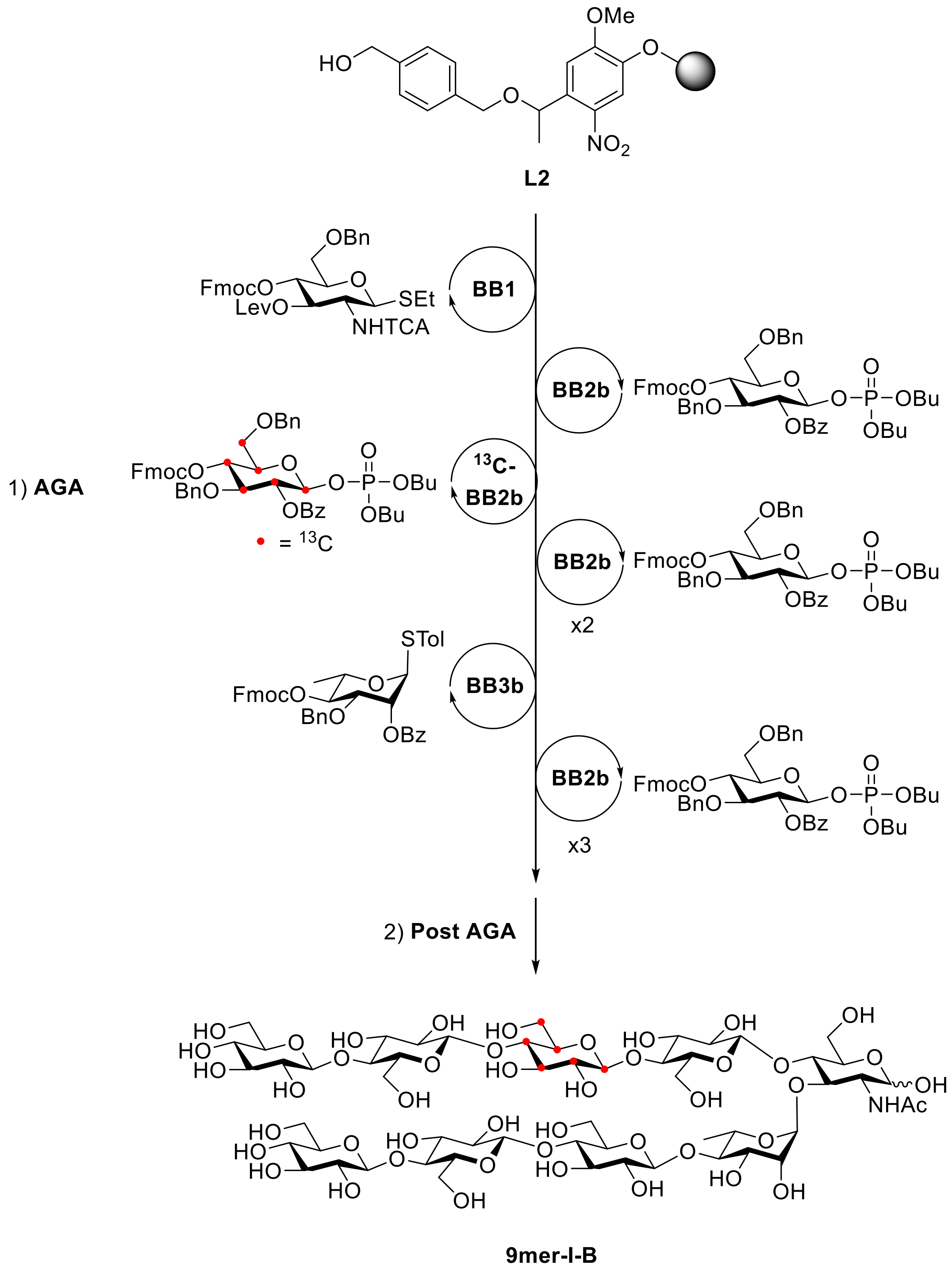

OMe
HO
O
NO2
L2
OBn
FmocO
LevO
O
SEt
NHTCA
BB1
BB2b
FmocO
BnO
O
OBz
O-P-OBu
OBu
1) AGA
13C-
BB2b
• = 13C
x2
STol
BB3b
x3
2) Post AGA
OH
NHAc
9mer-I-B

Step	BB	Modules	Notes
AGA	-	**A**	**L2** swelling
	**BB1**	**B**, **C1***, **D**, **E1**	**C1***: (**BB1**, -20 °C for 5 min, 0 °C for 40 min) *Double cycle
	**BB2b**	**B**, **C2***, **D**, **E1**	**C2***: (**BB2b**, -30 °C for 5 min, -10 °C for 40 min) *Double cycle
	**^{13}C-BB2b**	**B**, **C2**, **D**, **E1**	**C2**: (**^{13}C-BB2b**, -30 °C for 5 min, -10 °C for 40 min)
	(**BB2b**)x2	(**B**, **C2**, **D**, **E1**)x2	**C2**: (**BB2b**, -30 °C for 5 min, -10 °C for 40 min)
	-	**D**, **E2**	-
	**BB3b**	**B**, **C1**, **D**, **E1**	**C1**: (**BB3b**, -20 °C for 5 min, 0 °C for 20 min)
	(**BB2b**)x3	(**B**, **C2**, **D**, **E1**)x3	**C2**: (**BB3b**, -30 °C for 5 min, -10 °C for 40 min)
Post-AGA	-	**F, G1, H2, I**	**F**: (24 h)<br>**H2**: (10 h)<br>**I**: (Method B2: 26.9 min)

Automated synthesis, global deprotection, and purification afforded **9mer-I-B** as a white solid (2.0 mg, 10% overall yield).

^{1}H NMR (700 MHz, D_2O) δ 5.08 (d, *J* = 3.5 Hz, 0.6H, H-1α GlcNAc), 4.89 (s, 1H, H-1 Rha), 4.72 (d, *J* = 7.5 Hz, 0.4H, H-1β GlcNAc), 4.65 (d, *J* = 7.9 Hz, 1H, H-1 Glc), 4.53 (d, *J* = 8.0 Hz, 0.5H, H-1 ^{13}C-Glc), 4.52 – 4.43 (m, 6H, 5x H-1 Glc, H-5 Rha), 4.30 (d, *J* = 7.2 Hz, 0.5H, H-1 ^{13}C-Glc), 4.12 – 4.07 (m, 0.6H), 4.07 – 4.00 (m, 2.4H), 4.00 – 3.40 (m, 36.5H), 3.37 (t, *J* = 9.5 Hz, 3H), 3.34 – 3.24 (m, 8H), 3.20 (s, 0.5H), 2.01 (s, 3H, CH_3 Ac GlcNAc), 1.28 (d, *J* = 6.9 Hz, 3H, CH_3-6 Rha). ^{13}C NMR (176 MHz, D_2O) δ 103.03, 102.76, 102.47, 102.21, 78.57, 78.48, 78.34, 78.25, 78.12, 78.03, 75.98, 75.03, 74.96, 74.80, 74.72, 74.48, 74.28, 74.06, 73.83, 73.28, 73.15, 73.01, 72.93, 72.79, 72.68, 69.45, 60.05, 59.98, 59.81, 59.72. ESI-HRMS m/z 1530.532 $[M+Na]^+$ ($C_{50}{}^{13}C_6H_{95}NO_{45}Na$ requires 1530.5269).

RP-HPLC of crude 9mer-I-B (ELSD trace, Method B1)

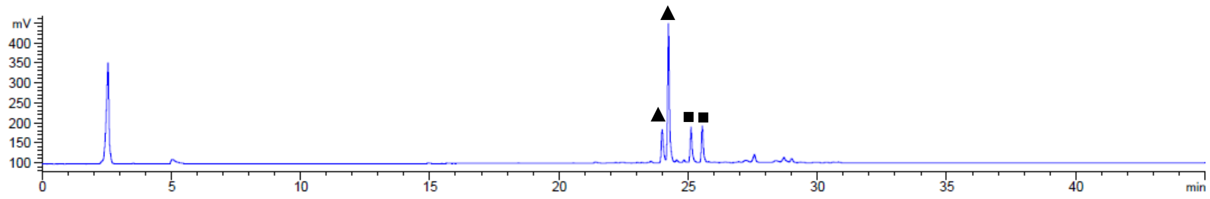

▲**9mer-I-B** (α and β anomers, m/z 1530 $[M+Na]^+$). ■ Incomplete methanolysis side-products.

RP-HPLC of 9mer-I-B (ELSD trace, Method B1, t_R = 24.0, 24.3 min)

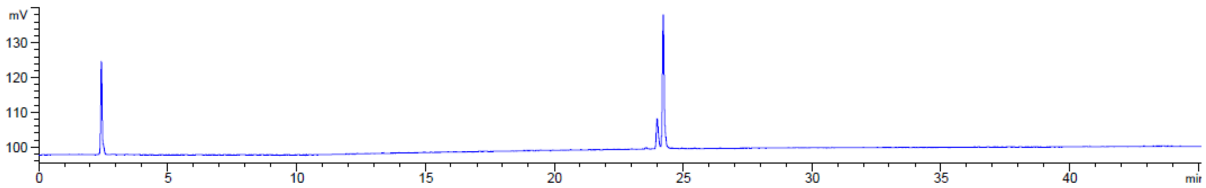

^1H NMR of 9mer-I-B (700 MHz, D_2O)

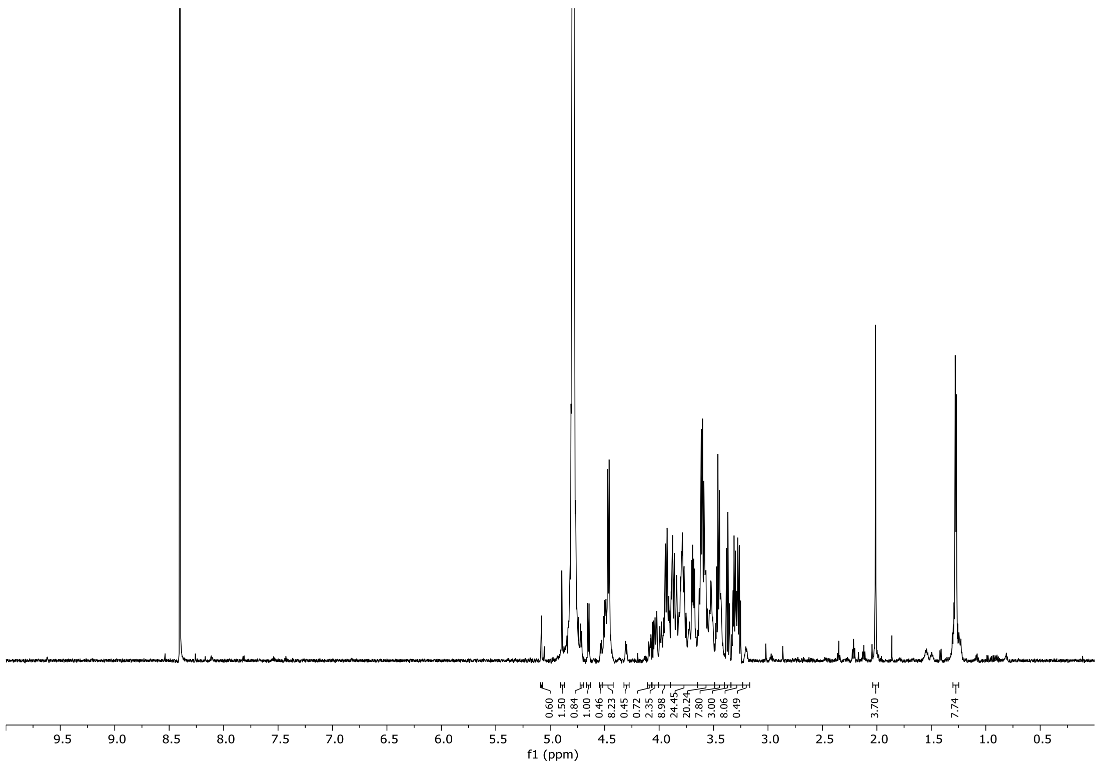

^{13}C NMR of 9mer-I-B (176 MHz, D_2O)

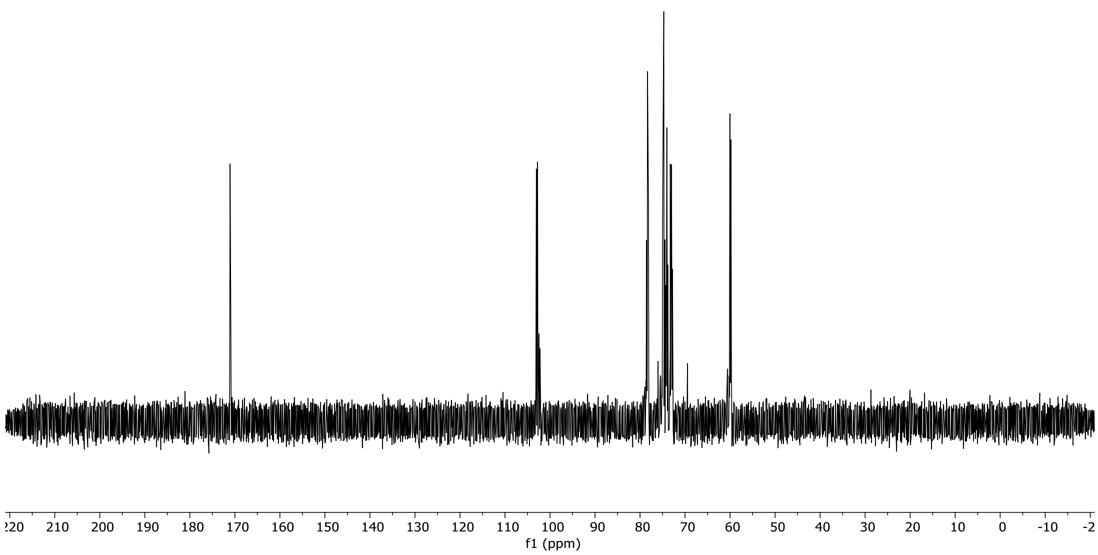

HSQC NMR of 9mer-I-B (D_2O)

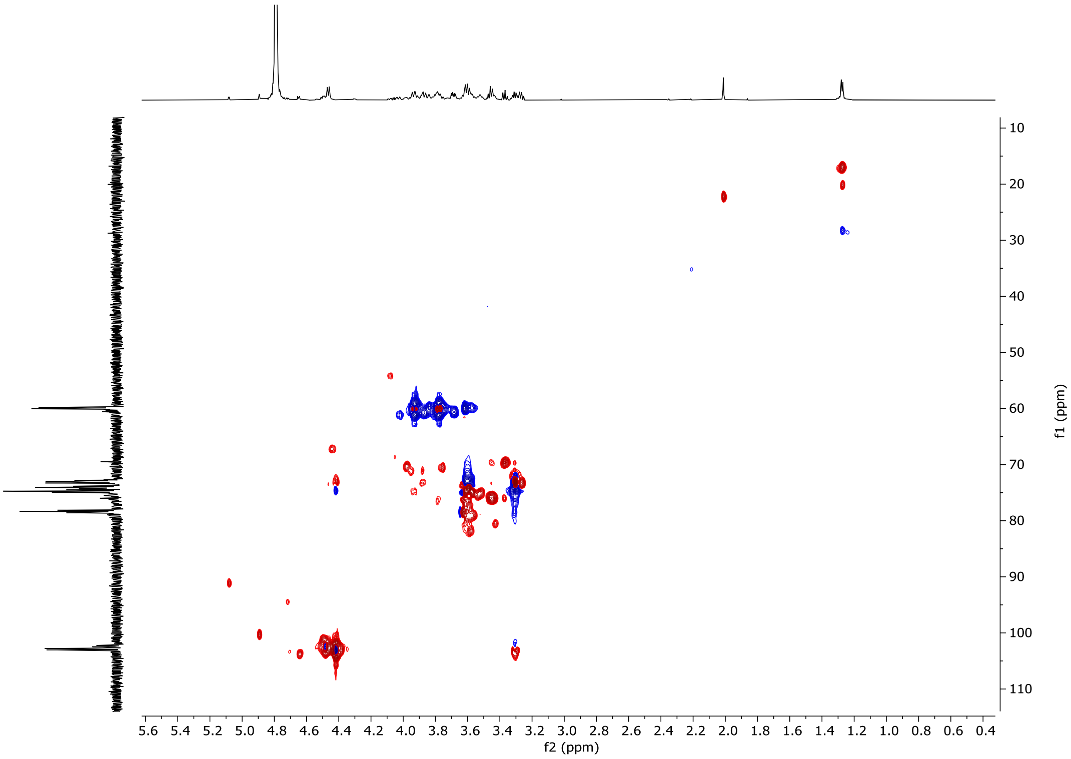

3.5.9 9mer-I-F

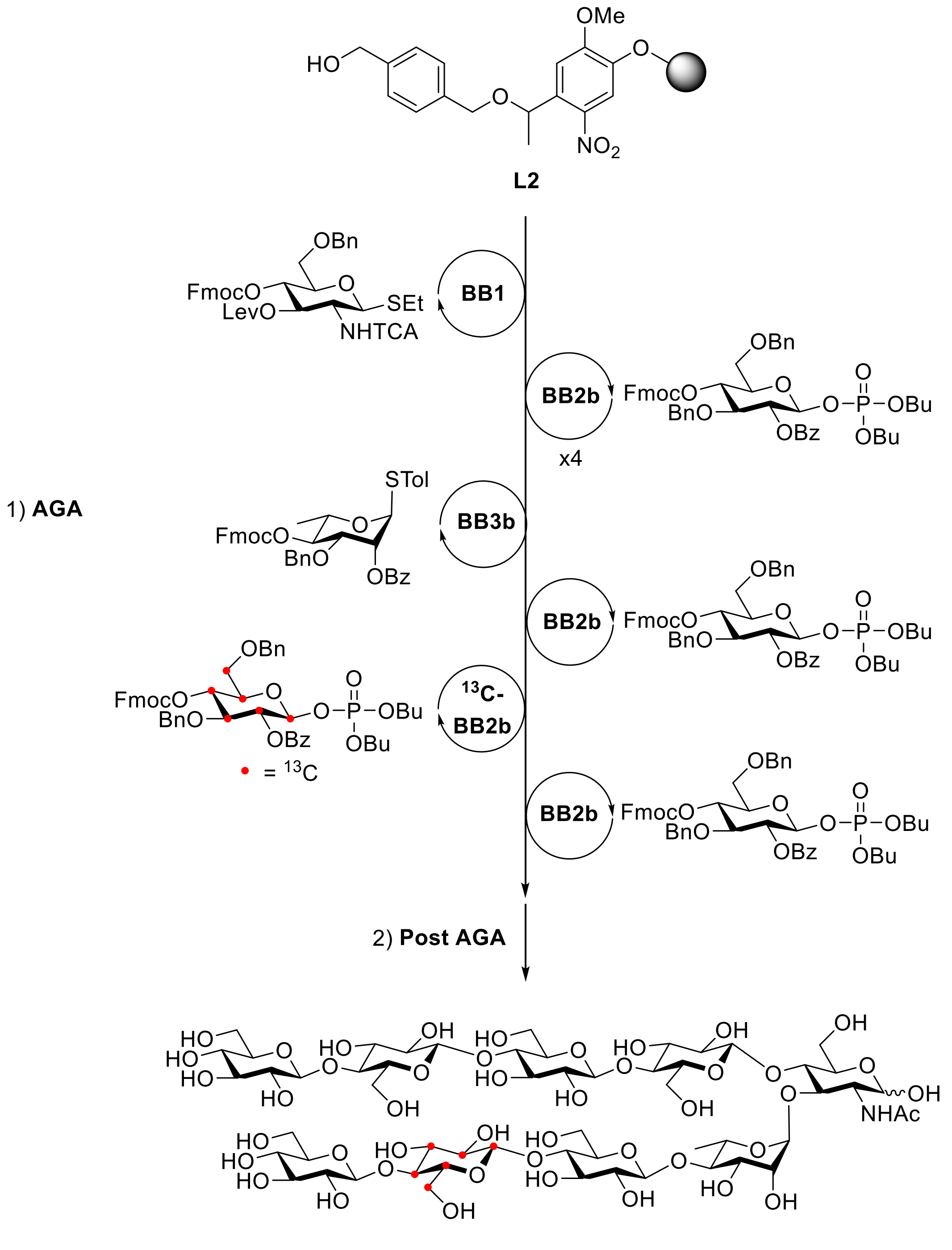

9mer-I-F

Step	BB	Modules	Notes
AGA	-	**A**	**L2** swelling
	**BB1**	**B**, **C1***, **D**, **E1**	**C1***: (**BB1**, -20 °C for 5 min, 0 °C for 40 min) *Double cycle
	**BB2b**	**B**, **C2***, **D**, **E1**	**C2***: (**BB2b**, -30 °C for 5 min, -10 °C for 40 min) *Double cycle
	(**BB2b**)x3	(**B**, **C2**, **D**, **E1**)x3	**C2**: (**BB2b**, -30 °C for 5 min, -10 °C for 40 min)
	-	**D**, **E2**	-
	**BB3b**	**B**, **C1**, **D**, **E1**	**C1**: (**BB3b**, -20 °C for 5 min, 0 °C for 20 min)
	**BB2b**	**B**, **C1**, **D**, **E1**	**C2**: (**BB2b**, -30 °C for 5 min, -10 °C for 40 min)
	**^{13}C-BB2b**	**B**, **C1**, **D**, **E1**	**C2**: (**^{13}C-BB2b**, -30 °C for 5 min, -10 °C for 40 min)
	**BB2b**	**B**, **C1**, **D**, **E1**	**C2**: (**BB2b**, -30 °C for 5 min, -10 °C for 40 min)
Post-AGA	-	**F, G1, H2, I**	**F**: (24 h)<br>**H2**: (10 h)<br>**I**: (Method B2: 26.9 min)

Automated synthesis, global deprotection, and purification afforded **9mer-I-F** as a white solid (1.9 mg, 10% overall yield).

^{1}H NMR (600 MHz, D_2O) δ 5.08 (d, *J* = 3.5 Hz, 0.6H, H-1α GlcNAc), 4.89 (s, 1H, H-1 Rha), 4.71 (d, *J* = 9.2 Hz, 0.4H, H-1β GlcNAc), 4.65 (d, *J* = 8.0 Hz, 1H, H-1 Glc), 4.59 (d, *J* = 7.3 Hz, 0.5H, H-1 ^{13}C-Glc), 4.53 – 4.43 (m, 5H, 4x H-1 Glc, H-5 Rha), 4.42 (d, *J* = 7.7 Hz, 1H, H-1 Glc), 4.32 (d, *J* = 7.6 Hz, 0.5H, H-1 ^{13}C-Glc), 4.11 – 4.01 (m, 1.5H), 4.00 – 3.73 (m, 18H), 3.72 – 3.66 (m, 3H), 3.65 – 3.49 (m, 13H), 3.48 – 3.40 (m, 6H), 3.36 (t, *J* = 9.2 Hz, 2H), 3.33 – 3.24 (m, 7H), 2.01 (s, 3H, CH_3 Ac GlcNAc), 1.27 (d, *J* = 6.3 Hz, 3H, CH_3-6 Rha). ^{13}C NMR (151 MHz, D_2O) δ 102.51, 102.20, 78.64, 78.38, 78.23, 78.12, 74.90, 74.69, 74.61, 74.33, 74.20, 73.93, 73.68, 73.04, 72.74, 72.46, 59.98, 59.71. ESI-HRMS m/z 1530.531 $[M+Na]^+$ ($C_{50}{}^{13}C_6H_{95}NO_{45}Na$ requires 1530.52).

RP-HPLC of crude 9mer-I-F (ELSD trace, Method B1)

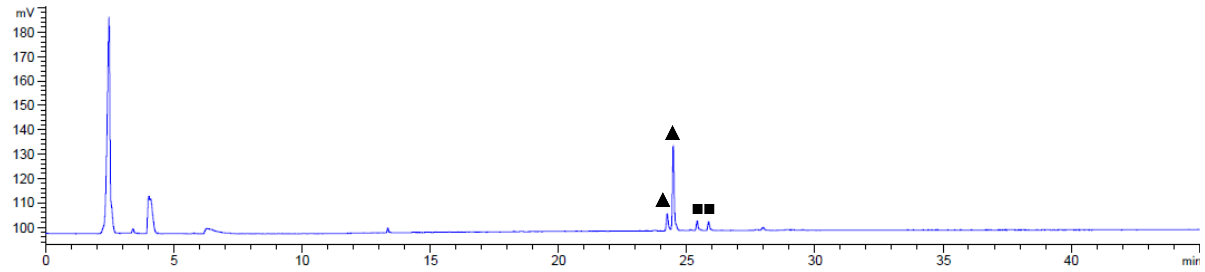

▲**9mer-I-F** (α and β anomers, m/z 1530 [M+Na]$^+$). ■ Incomplete methanolysis side-products.

RP-HPLC of 9mer-I-F (ELSD trace, Method B1, t_R = 24.3, 24.5 min)

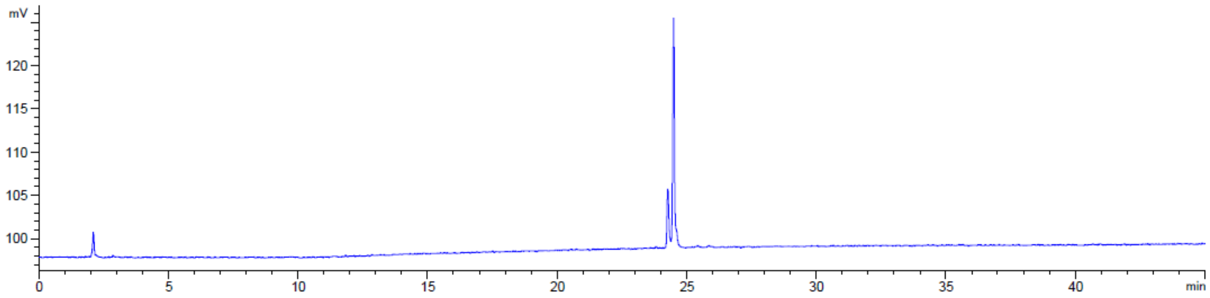

^{1}H NMR of 9mer-I-F (600 MHz, D_2O)

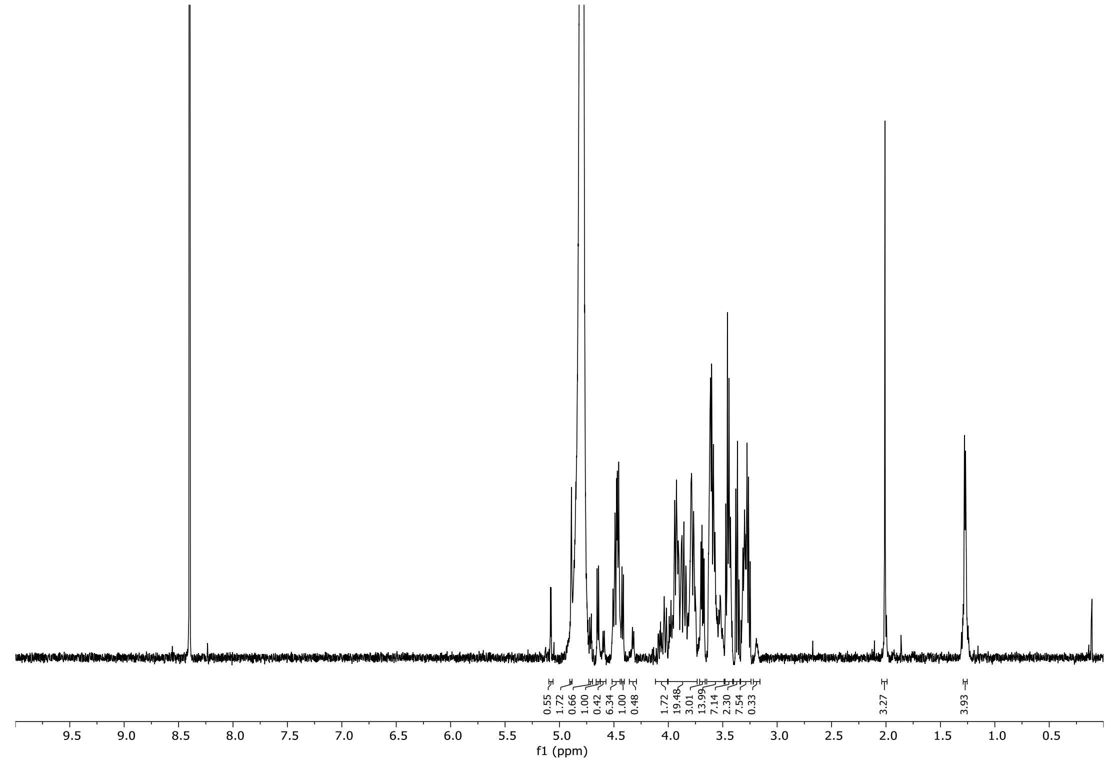

^{13}C NMR of 9mer-I-F (151 MHz, D_2O)

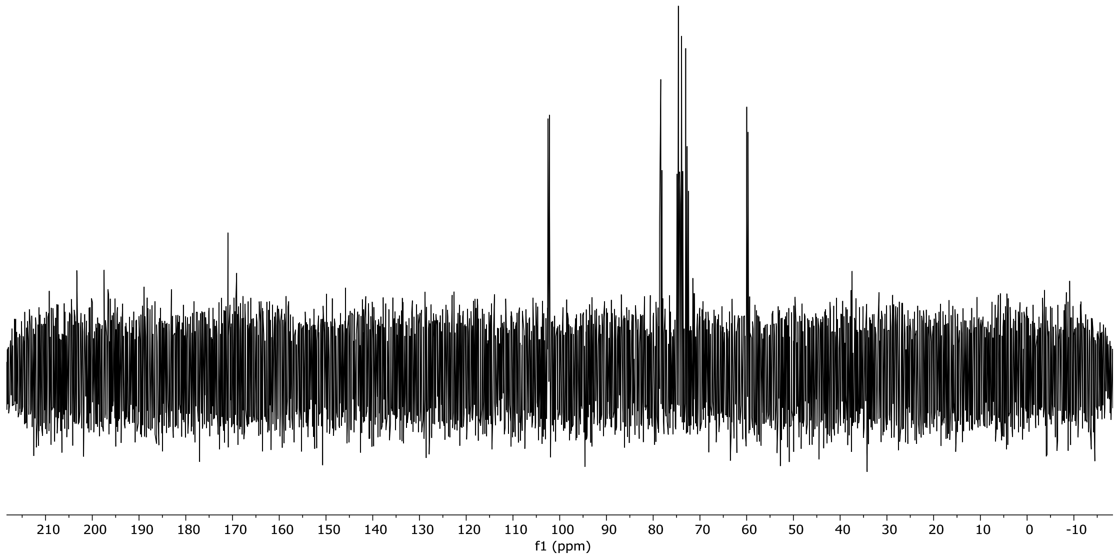

HSQC NMR of 9mer-I-F (D_2O)

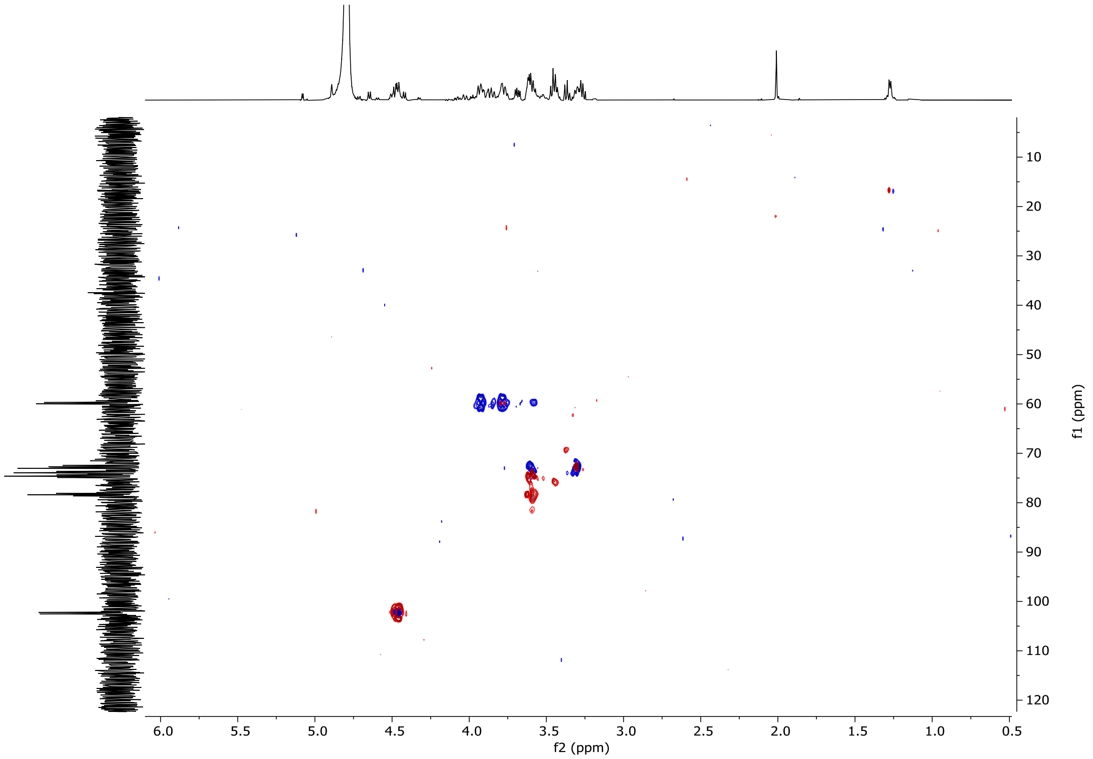

3.6 Comparison of BB3a and BB3b for AGA of 5mer-I and 9mer-I

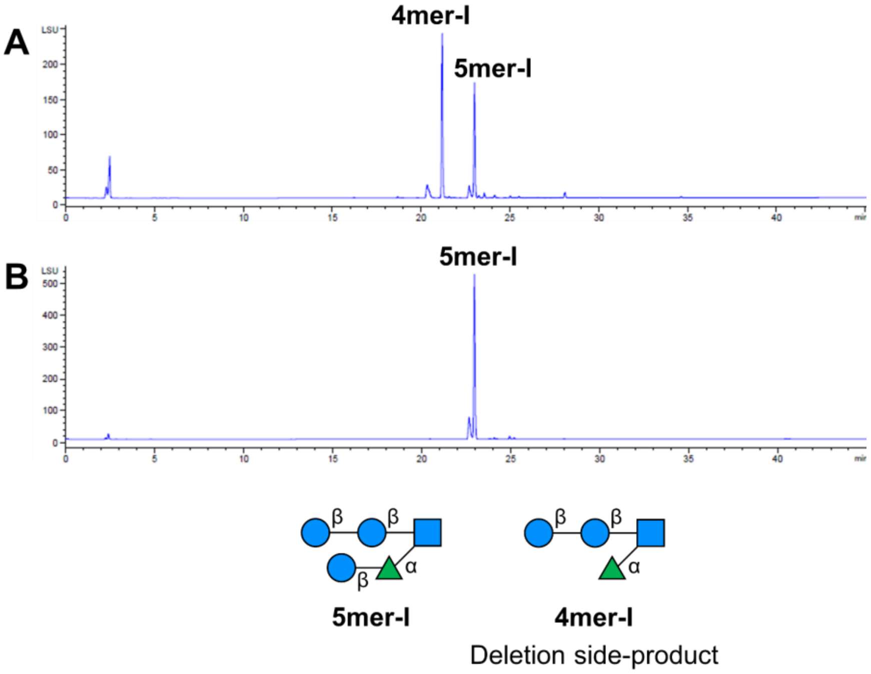

Figure 4 Comparison of crude RP HPLC traces (ELSD trace, Method A1) of **5mer-I** synthesized using **BB3a** (A) or **BB3b** (B).

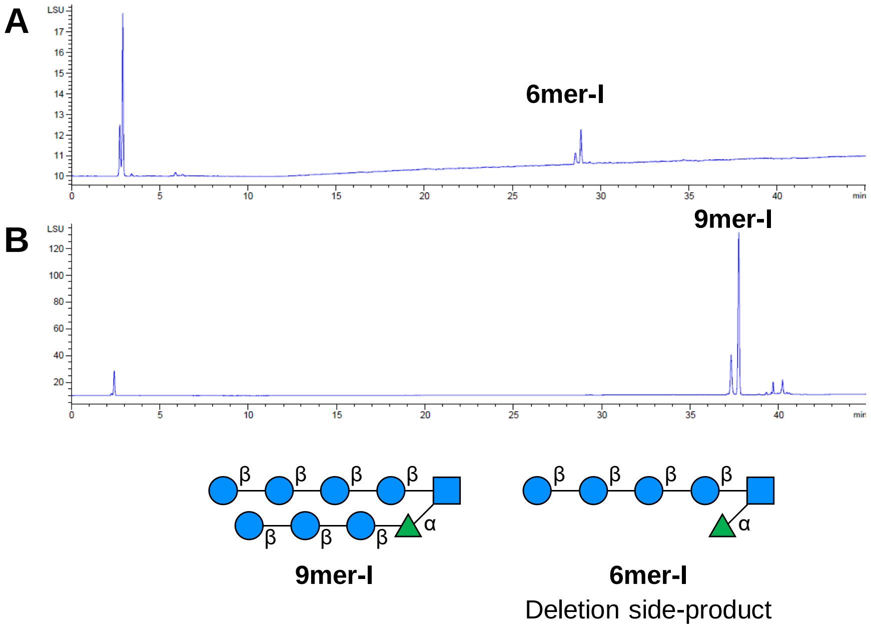

Figure 5 Comparison of crude RP HPLC traces (ELSD trace, Method A1) of **9mer-I** synthesized using **BB3a** (A) or **BB3b** (B).

3.7 Comparison of ^{1}H NMR of ^{13}C-labelled compounds

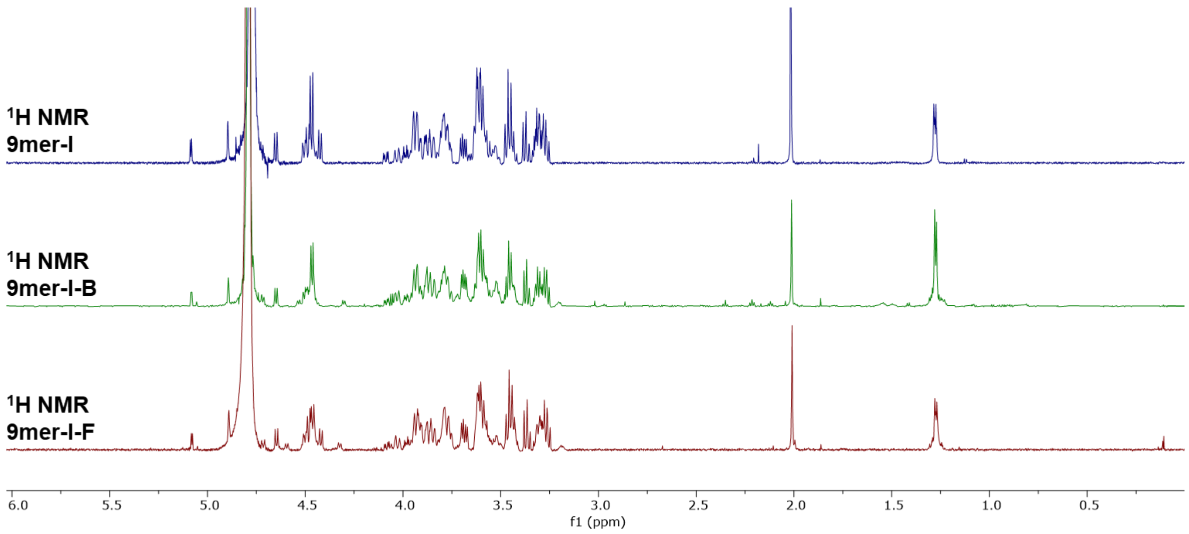

Figure 6 Comparison of ^{1}H NMR of ^{13}C-labelled compounds **9mer-I**, **9mer-I-B**, and **9mer-I-F**.

4 Molecular dynamics simulations

4.1 General materials and methods

All-atom molecular dynamics (MD) simulations were performed using gromacs 5.1.2.[9] The oligosaccharides were modeled using a modified version of GLYCAM06$_{OSMO,r14}$ force field,[10,11] and the system was solvated with TIP5P[12] water molecules to avoid excessive interactions between the monomers. The topology was converted to gromacs format using the glycam2gmx.pl script and solvated with 2100 water molecules using gromacs tools. The systems were kept at a constant temperature of 303 K using a Nosé-Hoover thermostat[13,14] and at constant pressure of 1 bar with the Parrinello-Rahman barostat.[15,16] Non-bonded interactions were cut-off at 1.4 nm, long range electrostatics were calculated using the particle mesh Ewald method.[17] Bonds involving hydrogens were constrained using the LINCS[18] to allow a 2 fs time step algorithm; water molecules were kept rigid with SETTLE.[19]

After energy minimization (steepest descent algorithm) and before the production run, the systems were equilibrated at 300 K for 50 ns in a canonical (NVT) ensemble (constant number of particles, volume and temperature) and subsequently at 300 K and 1 atm for 50 ns in an isothermal-isobaric (NPT) ensemble. All the modelled structures were simulated for 500 ns.

4.2 RMSD and RoG analysis of 3mer-I, 3mer-II, and 3mer-III

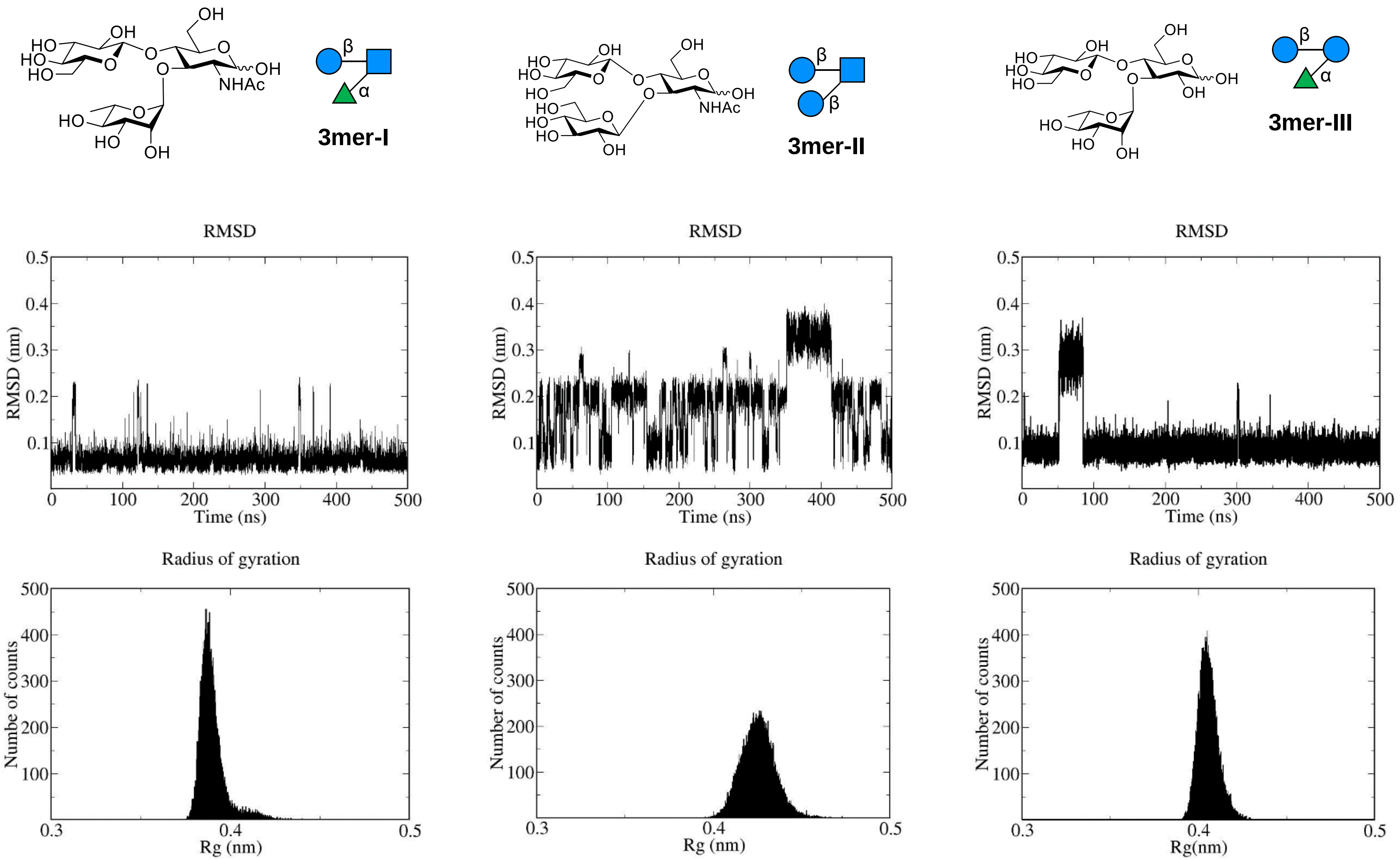

Figure 7 RMSD and RoG plots for **3mer-I**, **3mer-II**, and **3mer-III**.

4.3 Dihedral comparison of 3mer-I, 3mer-II, and 3mer-III

Glycosidic torsional angles were defined as follows: $\Phi = O_5\text{-}C_1\text{-}O\text{-}C_n$ and $\Psi = C_1\text{-}O\text{-}C_n\text{-}C_{(n-1)}$.

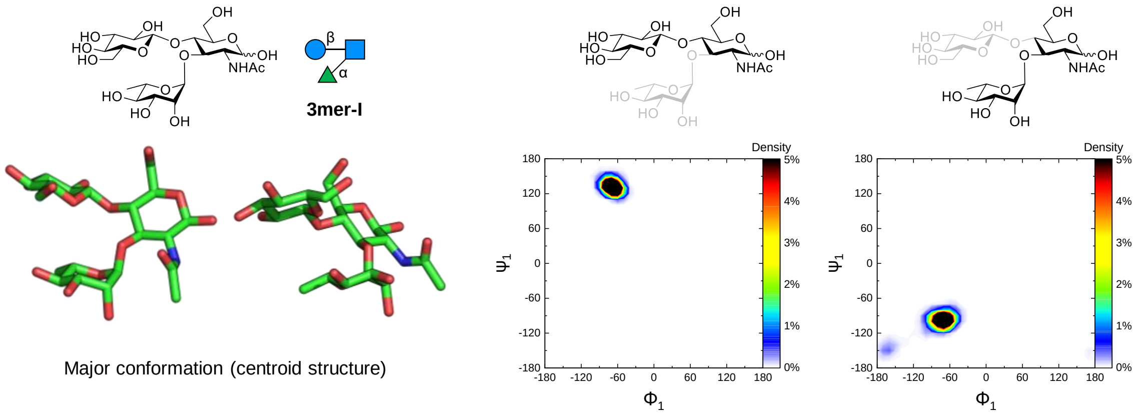

Figure 8 Ramachandran plots for the two glycosidic linkages of **3mer-I**.

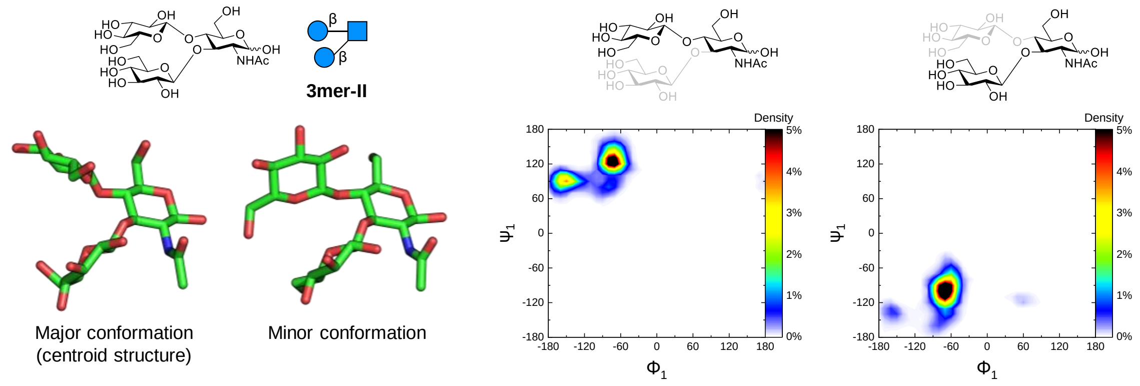

Figure 9 Ramachandran plots for the two glycosidic linkages of **3mer-II**.

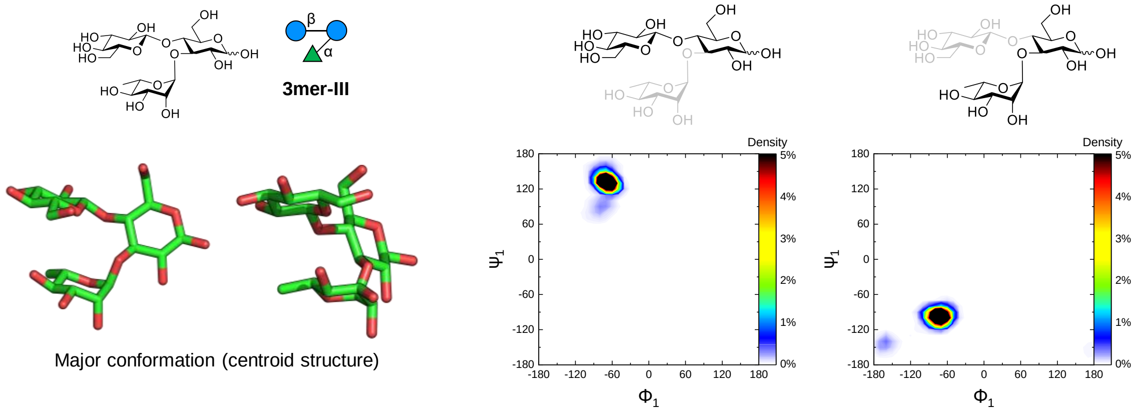

Figure 10 Ramachandran plots for the two glycosidic linkages of **3mer-III**.

3mer-III

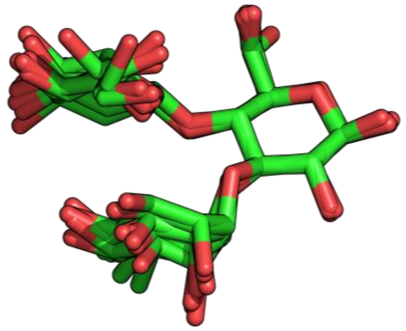

Figure 11 Overimposition of seven snapshots extracted from the MD simulation of **3mer-III**.

4.4 Comparison of 3mer-II major and minor conformations

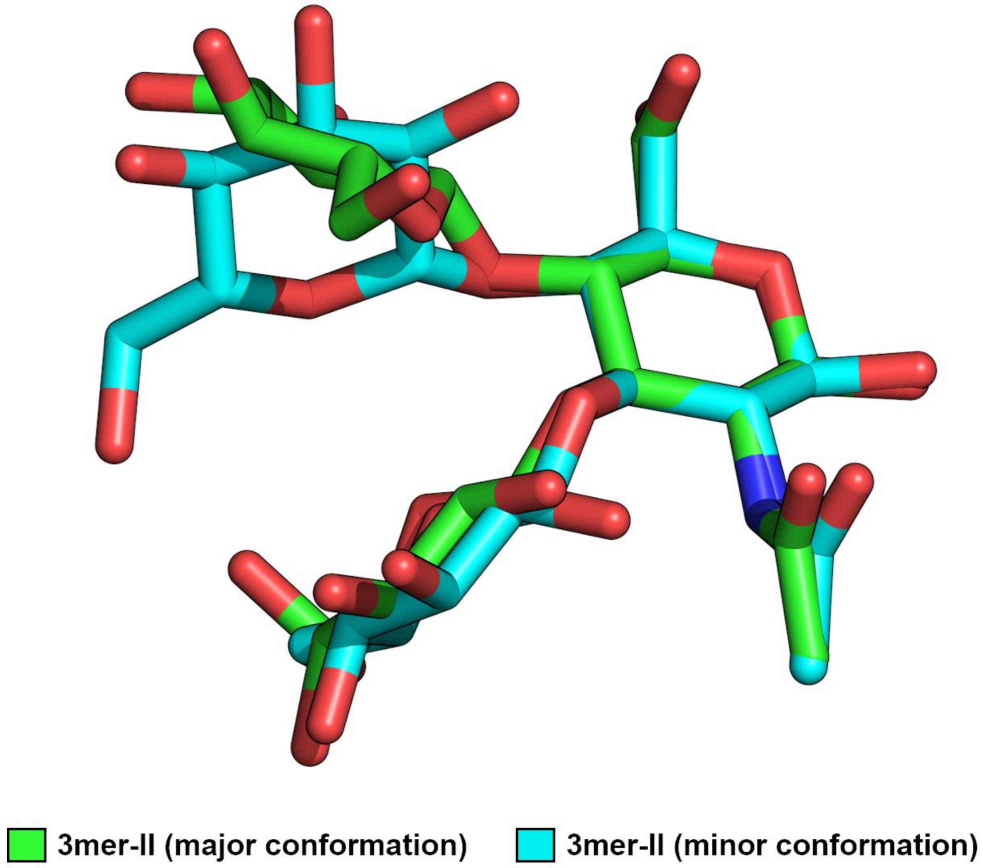

Figure 12 Two representative snapshots of the major and minor conformations of **3mer-II** overimposed.

4.5 Comparison of 3mer-I with 3mer-II

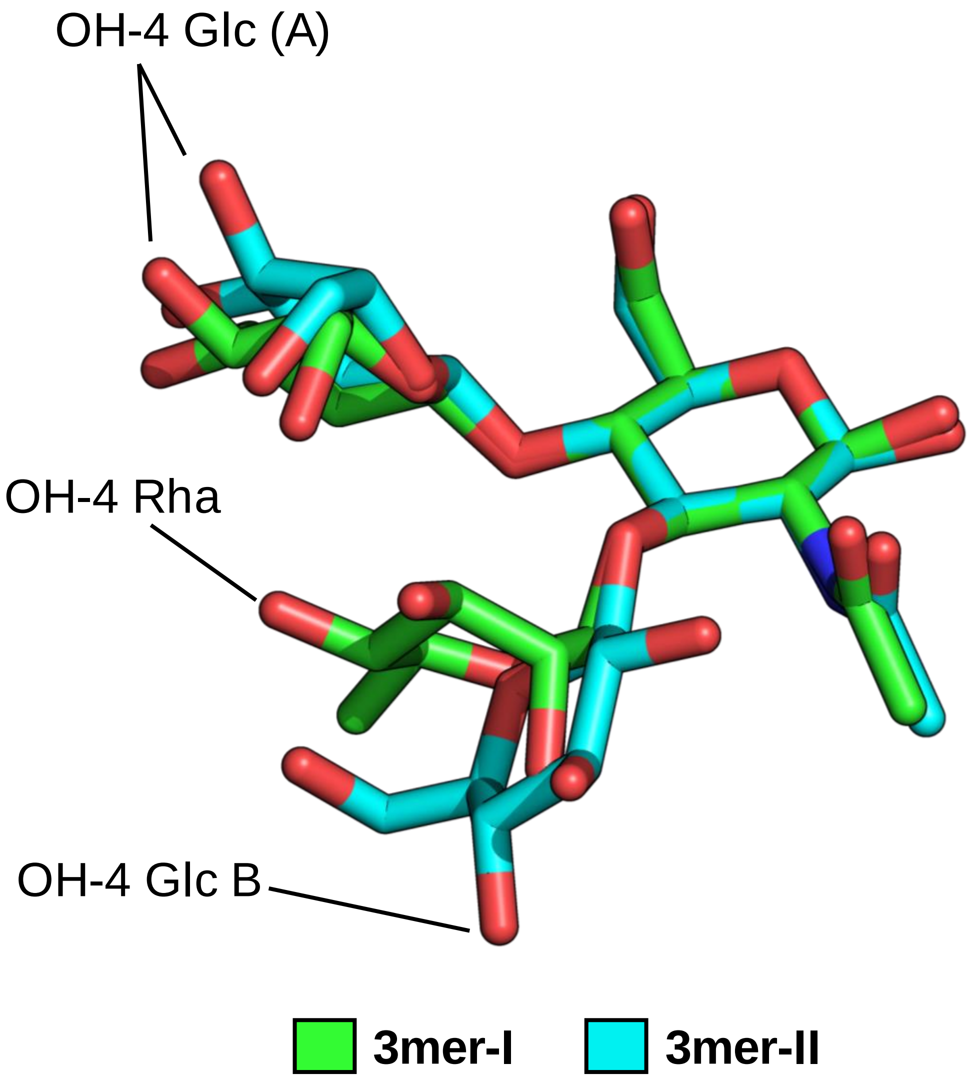

Figure 13 Two snapshots of **3mer-I** and **3mer-II** overimposed to show the different orientation of OH-4 of Rha *vs* OH-4 of Glc.

4.6 RMSD and RoG analysis of 9mer-I and 9mer-II

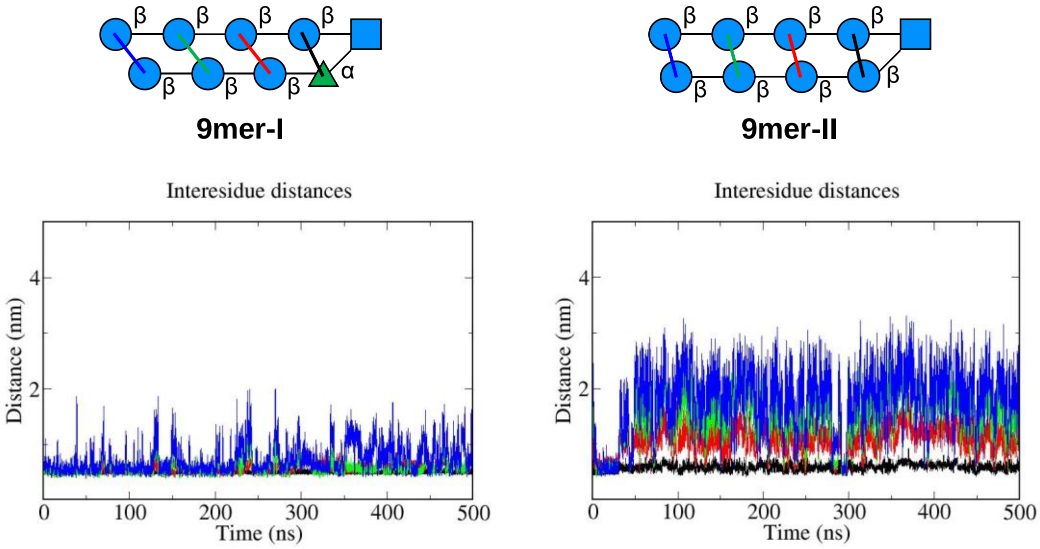

Figure 14 Inter residue plots extracted from MD simulations for **9mer-I** and **9mer-II**.

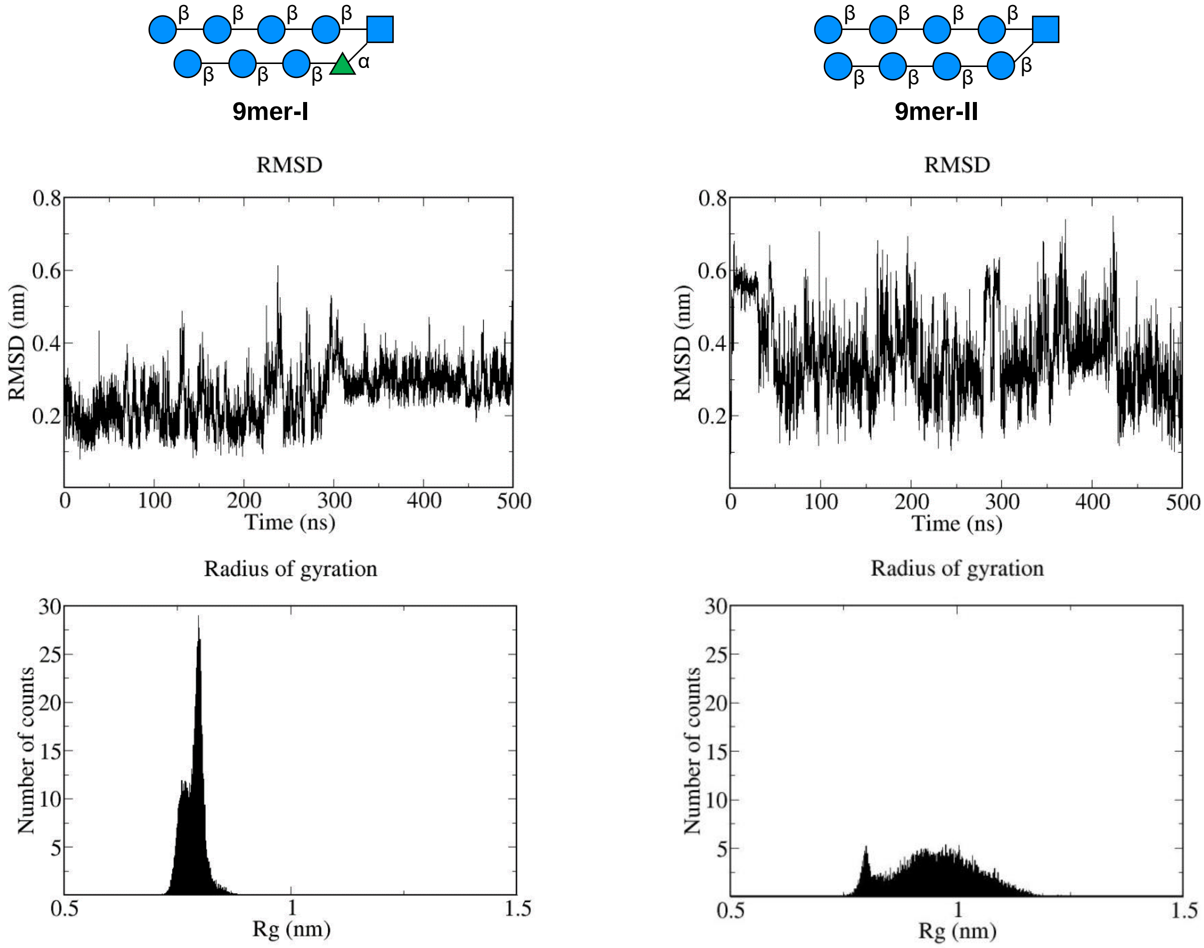

Figure 15 RMSD and RoG plots for **9mer-I** and **9mer-II**.

4.7 Comparison of hairpin with different length

Glycosidic torsional angles were defined as follows: $\Phi = O_5\text{-}C_1\text{-}O\text{-}C_n$ and $\Psi = C_1\text{-}O\text{-}C_n\text{-}C_{(n-1)}$.

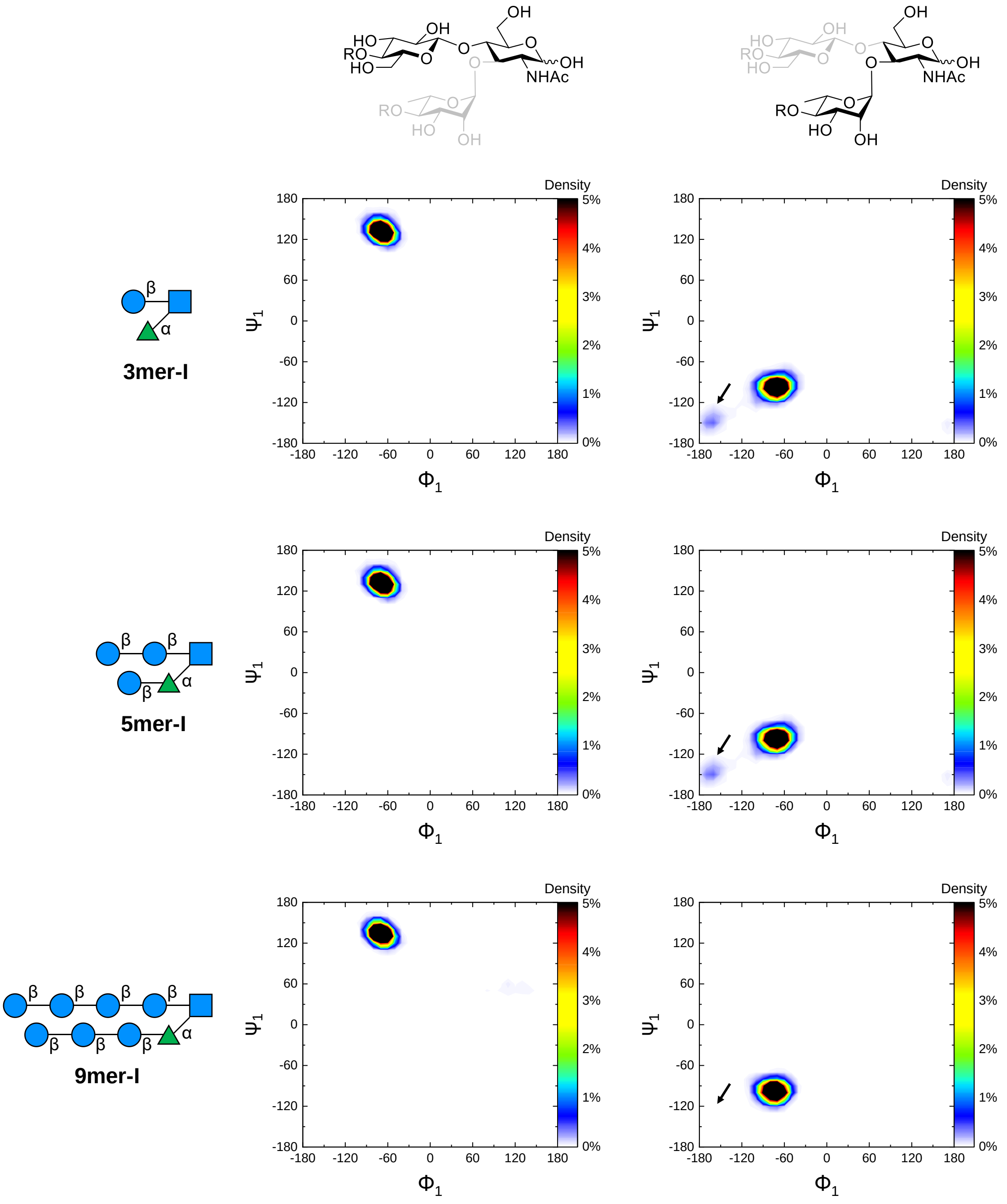

Figure 16 Ramachandran plots for the two glycosidic linkages of the turn unit for **3mer-I**, **5mer-I**, and **9mer-I**. Upon elongation of the hairpin strands, the population of minor conformers decreases (*black arrows*).

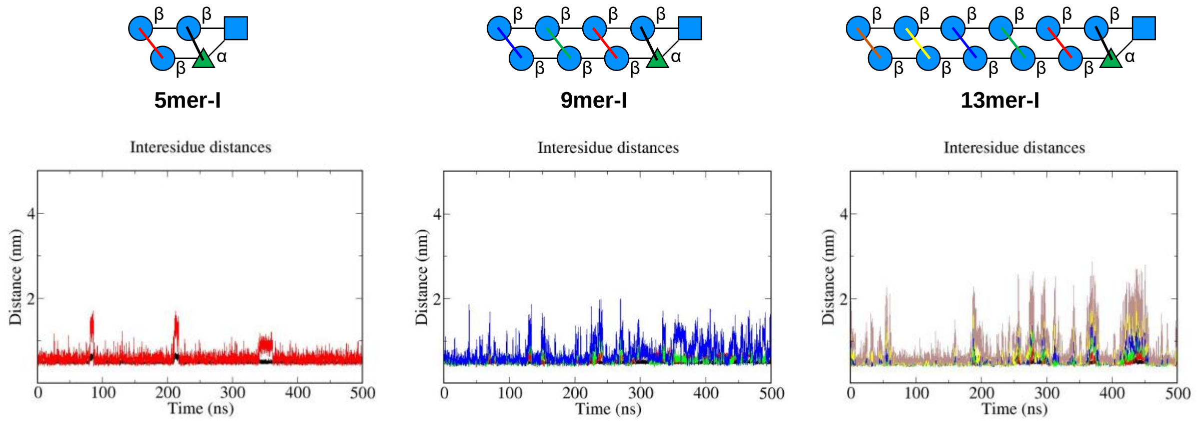

Figure 17 Inter residue plots extracted from MD simulations for **5mer-I**, **9mer-I**, and **13mer-I**.

4.8 Inter-proton distances for 3mer-I

	GlcNAc-1	GlcNAc-2	GlcNAc-3	GlcNAc-4	GlcNAc-5	GlcNAc-6	GlcNAc-6’	GlcNAc-CH_3 Ac
**Rha-1**	4.54	3.71	2.56	4.53	5.13	7.18	6.80	3.83
**Rha-2**	6.23	4.21	4.35	4.94	6.67	8.27	7.74	4.58
**Rha-3**	6.90	4.93	4.60	4.36	6.46	7.70	6.91	6.61
**Rha-4**	8.12	6.99	5.49	6.53	7.65	9.33	8.51	7.34
**Rha-5**	6.12	5.28	3.56	4.00	5.07	6.49	5.61	7.28
**Rha-6 a/b/c**	7.47/7.48/7.49	7.30/7.31/7.32	4.92/4.93/4.94	6.24/6.25/6.26	6.38/6.39/6.40	8.03/8.04/8.05	7.18/7.19/7.20	8.35/8.36/8.37
**Glc-1**	5.88	5.15 Å	4.56	2.51	3.84	3.59	2.53	>9.0
**Glc-2**	6.87	6.57 Å	4.66	4.44	4.88	5.65	4.57	>9.0
**Glc-3**	8.52	7.8 Å	6.83	5.22	6.29	5.89	4.75	>9.0
**Glc-4**	8.76	7.5 Å	6.44	5.42	7.07	7.52	6.38	>9.0
**Glc-5**	7.91	6.32 Å	6.30	3.97	6.24	5.93	4.91	>9.0
**Glc-6/6’**	9.27/8.52	7.1/6.44 Å	7.33/6.42	5.44/4.92	8.02/7.43	8.13/7.89	7.13/6.92	>9.0

Table 1 Average inter-proton distances (in Å) extracted from MD simulations. Cells highlighted in green represent experimentally observed NOEs (see section 5).

	Rha-1	Rha-2	Rha-3	Rha-4	Rha-5	Rha-6 a/b/c
**Glc-1**	6.20	6.56	5.16	6.94	4.08	6.00
**Glc-2**	5.80	6.43	4.77	5.16	2.64	3.55
**Glc-3**	8.13	8.24	6.28	7.47	5.02	6.28
**Glc-4**	6.79	6.39	4.17	5.00	3.44	4.69
**Glc-5**	7.17	6.67	4.79	6.90	4.64	6.70
**Glc-6/6'**	7.38/6.24	6.17/5.02	4.16/2.94	6.21/5.0	4.98/3.96	6.91/5.95

Table 2 Average inter-proton distances (in Å) extracted from MD simulations. Cells highlighted in green represent experimentally observed NOEs (see section 5).

4.9 Inter-proton distances for 3mer-II

	GlcNAc-1	GlcNAc-2	GlcNAc-3	GlcNAc-4	GlcNAc-5	GlcNAc-6	GlcNAc-6'	GlcNAc-CH_3 Ac
**Glc A-1**	5.9	5.02	4.08	2.46	4.02	3.60	4.13	>8
**Glc A-2**	6.73	6.48	4.80	4.17	4.67	4.57	5.01	>8
**Glc A-3**	8.45	7.54	6.50	4.96	6.34	5.51	6.10	>8
**Glc A-4**	8.76	7.86	6.39	5.69	6.96	6.75	7.21	>8
**Glc A-5**	8.00	6.60	5.75	4.40	6.37	5.92	6.39	>8
**Glc A-6/6'**	8.92/8.87	7.47/7.38	6.42/6.41	5.86/5.76	7.64/7.61	7.64/7.57	8.02/7.95	>8
**Glc B-1**	5.07	3.7	2.59	3.9	5.15	6.18	6.43	5.12
**Glc B-2**	6.66	4.8	4.36	4.65	6.51	6.93	7.32	6.40
**Glc B-3**	7.51	6.12	5.25	6.67	7.90	8.94	9.20	5.80
**Glc B-4**	8.17	7.0	5.50	6.43	7.66	8.37	8.69	7.74
**Glc B-5**	6.60	6.08	4.10	6.12	6.61	8.02	8.18	5.94
**Glc B-6/6'**	7.82/7.84	7.47/7.63	5.21/5.31	6.53/6.91	6.96/7.17	8.00/8.40	8.20/8.57	8.14/7.92

Table 3 Average inter-proton distances (in Å) extracted from MD simulations. Cells highlighted in green represent experimentally observed NOEs (see section 5).

	Glc A-1	Glc A-2	Glc A-3	Glc A-4	Glc A-5	Glc A-6/6'
**Glc B-1**	4.62	5.56	6.73	6.25	5.44	5.66
**Glc B-2**	5.00	6.04	6.60	6.02	5.07	5.02
**Glc B-3**	7.11	7.76	8.71	7.81	7.2	6.78
**Glc B-4**	6.17	6.40	7.22	6.00	5.80	5.23
**Glc B-5**	6.32	6.53	7.95	7.01	6.74	6.52
**Glc B-6/6'**	6.07/6.54	5.69/6.21	7.04/7.59	5.90/6.40	6.10/6.66	5.80/6.33

Table 4 Average inter-proton distances (in Å) extracted from MD simulations. Cells highlighted in green represent experimentally observed NOEs (see section 5).

4.10 Inter-proton distances for 3mer-III

	Glc A-1	Glc A-2	Glc A-3	Glc A-4	Glc A-5	Glc A-6	Glc A-6’
**Rha-1**	4.64	3.50	2.60	4.50	5.24	6.80	7.11
**Rha-2**	6.32	4.13	4.41	4.90	6.71	7.63	8.10
**Rha-3**	6.89	4.94	4.57	4.27	6.37	6.70	7.43
**Rha-4**	8.13	6.91	5.42	6.39	7.60	8.33	9.12
**Rha-5**	6.11	5.25	3.41	3.86	4.94	5.42	6.27
**Rha-6 a/b/c**	7.42/7.43/7.44	7.21/7.22/7.23	4.77/4.78/4.79	6.08/6.09/6.1	6.30/6.29/6.28	7.04/7.05/7.06	7.86/7.87/7.88
**Glc B-1**	5.92	5.31	4.51	2.52	3.80	2.66	3.73
**Glc B-2**	6.87	6.61	4.62	4.45	4.87	4.67	5.78
**Glc B-3**	8.53	7.94	6.77	5.22	6.24	4.88	6.04
**Glc B-4**	8.79	7.60	6.37	5.44	7.05	6.49	7.63
**Glc B-5**	7.95	6.50	6.20	4.00	6.19	5.00	6.03
**Glc B-6/6’**	8.64/9.25	6.64/7.25	6.38/7.15	4.99/5.40	7.45/7.92	7.03/7.19	7.97/8.14

Table 5 Average inter-proton distances (in Å) extracted from MD simulations. Cells highlighted in green represent experimentally observed NOEs (see section 5).

	Rha-1	Rha-2	Rha-3	Rha-4	Rha-5	Rha-6 a/b/c
**Glc B-1**	6.23	6.57	5.13	6.90	4.07	5.98
**Glc B-2**	5.98	6.59	4.90	5.39	2.80	3.84
**Glc B-3**	8.21	8.30	6.34	7.56	5.16	6.42
**Glc B-4**	6.92	6.55	4.40	5.26	3.80	5.00
**Glc B-5**	7.14	6.66	4.83	6.88	4.72	6.69
**Glc B-6/6'**	6.32/7.27	5.18/6.13	3.24/4.25	5.22/6.21	4.25/5.10	6.10/6.92

Table 6 Average inter-proton distances (in Å) extracted from MD simulations. Cells highlighted in green represent experimentally observed NOEs (see section 5).

4.11 Inter-proton distances for 5mer-I

	Glc C-1	Glc C-2	Glc C-3	Glc C-4	Glc C-5	Glc C-6	Glc C-6’
**Rha-1**	6.33	7.84	>6	>6	>6	>6	>6
**Rha-2**	5.20	6.94	>6	>6	>6	>6	>6
**Rha-3**	3.75	4.74	>6	>6	>6	>6	>6
**Rha-4**	2.51	4.45	>6	>6	>6	>6	>6
**Rha-5**	4.48*	4.69	>6	>6	>6	>6	>6
**Rha-6 a/b/c**	4.79/4.80/4.81	5.15/5.16/5.17	>6	>6	>6	>6	>6
**Glc A-1**	7.85	6.95	>6	>6	>6	>6	>6
**Glc A-2**	6.26	5.40	>6	>6	>6	>6	>6
**Glc A-3**	7.73	5.90	>6	>6	>6	>6	>6
**Glc A-4**	4.84	3.16	>6	>6	>6	>6	>6
**Glc A-5**	6.98	5.60	>6	>6	>6	>6	>6
**Glc A-6/6’**	4.53/5.34	3.87/4.19	>6	>6	>6	>6	>6
**Glc B-1**	5.51	2.88	>6	>6	>6	>6	>6
**Glc B-2**	7.98	5.42	>6	>6	>6	>6	>6
**Glc B-3**	7.00	4.52	>6	>6	>6	>6	>6
**Glc B-4**	8.70	6.03	>6	>6	>6	>6	>6
**Glc B-5**	6.19	3.80	>6	>6	>6	>6	>6
**Glc B-6/6’**	7.70/8.20	5.48/5.90	>6	>6	>6	>6	>6

Table 7 Average inter-proton distances (in Å) extracted from MD simulations. Cells highlighted in green represent experimentally observed NOEs (see section 5).
*Likely a spin-diffusion artifact.

	Glc B-1	Glc B-2	Glc B-3	Glc B-4	Glc B-5	Glc B-6/6’
**Glc A-1**	5.98	6.94	>5	>7	>5	>7
**Glc A-2**	5.10	6.79	>5	>7	>5	>7
**Glc A-3**	4.25	4.68	>5	>7	>5	>7
**Glc A-4**	2.42	4.48	>5	>7	4.25	>7
**Glc A-5**	4.13	4.81	>5	>7	>5	>7
**Glc A-6/6’**	3.55/3.14	5.40/4.38	>5	>7	>5	>7

Table 8 Average inter-proton distances (in Å) extracted from MD simulations. Cells highlighted in green represent experimentally observed NOEs (see section 5).

	GlcNAc-2	GlcNAc-3	GlcNAc-4	GlcNAc-5	GlcNAc-6	GlcNAc-6’	GlcNAc-CH_3 Ac
**Rha-1**	3.71	2.53	-	-	-	-	3.80
**Rha-5**	-	3.63	-	-	-	-	-
**Glc A-1**	-	-	2.52	3.79	3.55	2.49	-

Table 9 Average inter-proton distances (in Å) extracted from MD simulations for selected residues. Cells highlighted in green represent experimentally observed NOEs (see section 5).

	Glc A-1	Glc A-2	Glc A-3	Glc A-4	Glc A-5	Glc A-6/6’
**Rha-5**	-	2.66	-	-	-	-
**Rha-6 a/b/c**	-	3.48	-	-	-	-

Table 10 Average inter-proton distances (in Å) extracted from MD simulations for selected residues. Cells highlighted in green represent experimentally observed NOEs (see section 5).

4.12 Inter-proton distances for 9mer-I

	Glc A-1	Glc A-2	Glc A-3	Glc A-4	Glc A-5	Glc A-6	Glc A-6'
**Rha-1**	6.26	5.82	>6	6.82	>6	6.13	>6
**Rha-2**	6.66	6.58	>6	6.49	>6	4.93	>6
**Rha-3**	5.20	4.91	>6	4.20	4.80	2.67	4.21
**Rha-4**	6.92	5.21	>6	4.83	>6	4.65	>6
**Rha-5**	3.99	2.58	4.80	3.18	4.50	3.57	4.83
**Rha-6 a/b/c**	5.90/5.91/5.92	3.37/3.38/3.39	>6	4.35/4.36/4.37	>6	5.59/5.60/5.61	>6

Table 11 Average inter-proton distances (in Å) extracted from MD simulations. Cells highlighted in green represent experimentally observed NOEs (see section 5).

	Glc B-1	Glc B-2	Glc B-3	Glc B-4	Glc B-5	Glc B-6/6'
**Glc G-1**	5.09	>6	6.64	>6	6.27	>6
**Glc G-2**	2.60	5.11	4.10	>6	4.01	>6
**Glc G-3**	4.82	>6	5.06	>6	5.44	>6
**Glc G-4**	4.10	>6	4.27	>6	3.63	>6
**Glc G-5**	5.88	>6	6.64	>6	5.99	>6
**Glc G-6/6'**	6.20/6.41	>6	6.81/7.17	>6	5.54/5.87	>6

Table 12 Average inter-proton distances (in Å) extracted from MD simulations. Cells highlighted in green represent experimentally observed NOEs (see section 5).

	Glc C-1	Glc C-2	Glc C-3	Glc C-4	Glc C-5	Glc C-6/6’
**Glc F-1**	>5	>5	>5	>5	>5	>5
**Glc F-2**	>5	>5	>5	>5	>5	>5
**Glc F-3**	>5	>5	>5	>5	>5	>5
**Glc F-4**	>5	>5	>5	>5	>5	>5
**Glc F-5**	>5	4.44	>5	>5	>5	5.43
**Glc F-6/6’**	>5	5.43/5.59	>5	>5	>5	>5

Table 13 Average inter-proton distances (in Å) extracted from MD simulations. Cells highlighted in green represent experimentally observed NOEs (see section 5).

	Glc D-1	Glc D-2	Glc D-3	Glc D-4	Glc D-5	Glc D-6/6’
**Glc E-1**	>5	>6	>7	>7	>6	>7
**Glc E-2**	>5	>6	>7	>7	>6	>7
**Glc E-3**	>5	>6	>7	>7	>6	>7
**Glc E-4**	>5	>6	>7	>7	>6	>7
**Glc E-5**	>5	>6	>7	>7	>6	>7
**Glc E-6/6’**	>5	>6	>7	>7	>6	>7

Table 14 Average inter-proton distances (in Å) extracted from MD simulations. Cells highlighted in green represent experimentally observed NOEs (see section 5).

5 NMR studies

5.1 General materials and methods

^1H, ^{13}C, HSQC, 1D and 2D TOCSY, 1D and 2D ROESY, 2D NOESY NMR spectra were recorded on a Varian 400-MR (400 MHz), Varian 600-NMR (600 MHz), Bruker Biospin AVANCE700 (700 MHz) Bruker AVANCE III 800 (800 MHz) spectrometer. Samples were prepared by dissolving lyophilized samples in D_2O (concentration ≈ 1 - 6 mM). Proton resonances of the oligosaccharides were assigned using a combination of ^1H, 2D COSY, HSQC, 1D and 2D TOCSY. Selective 1D TOCSY (HOHAHA, pulse program: seldigpzs) spectra were recorded using different mixing times to assign all the resonances (d9 = 40, 80, 120, 160, and 200 ms). 2D TOCSY (pulse program: mlevphpp) spectra were recorded using different mixing times (d9 = 80, or 120 ms). Selective 1D t-ROESY (pulse program: selrogp.2) spectra were recorded using different mixing times (p15 = 100, 200, or 300 ms). 2D t-ROESY (pulse program: reosyph.2) and 2D NOESY (pulse program: noesygpphpp) spectra were recorded using different mixing times (p15 = 100, 200, or 300 ms for ROESY and d8 = 600, 800, or 1000 ms for NOESY). Monosaccharide were named as follows: D-glucose (Glc), D-*N*-acetyl glucosamine (GlcNAc), L-rhamnose (Rha). Labelling of protons in a monosaccharides is done as follows: e.g. proton attached to C-1 of Rha is named “Rha-1”. Resonances of residues at the reducing end are additionally labelled with α or β.

5.2 Determination of the non-conventional hydrogen bond

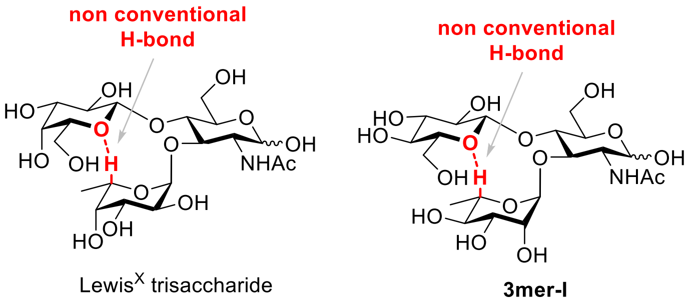

Figure 18 Comparison of a Lewis^X trisaccharide and **3mer-I** to highlight the non-conventional H-bond.[20,21]

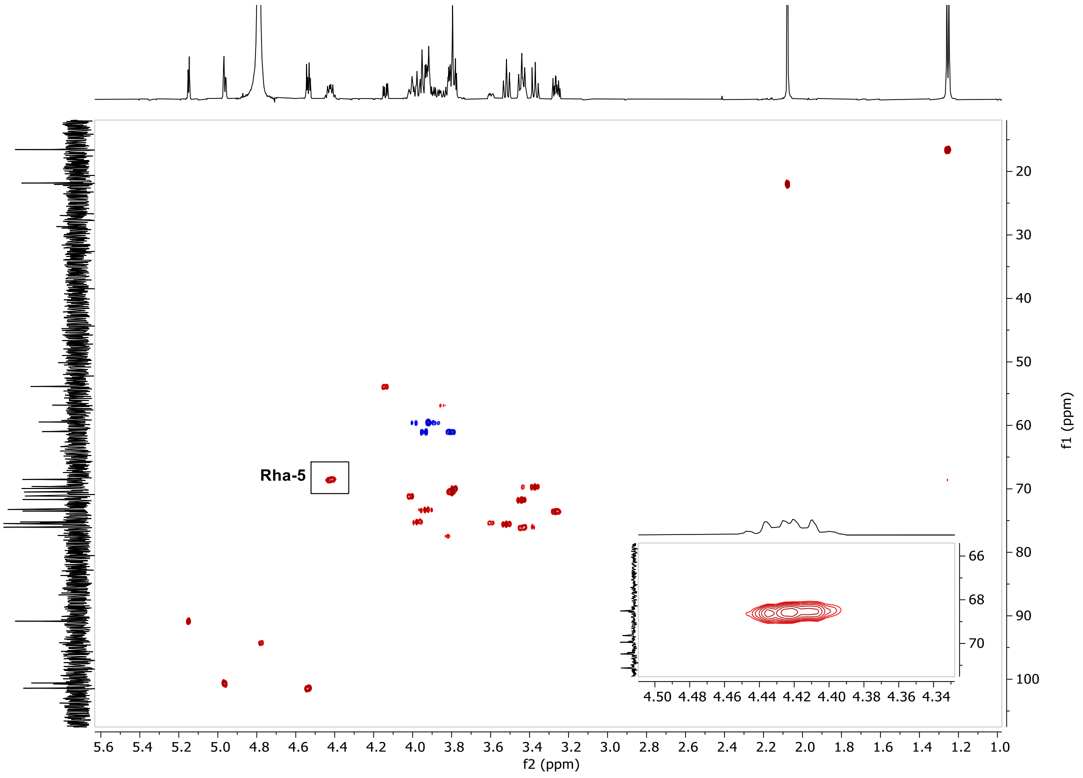

Figure 19 HSQC spectra of **3mer-I** with zoom-in on Rha-5 cross peak.

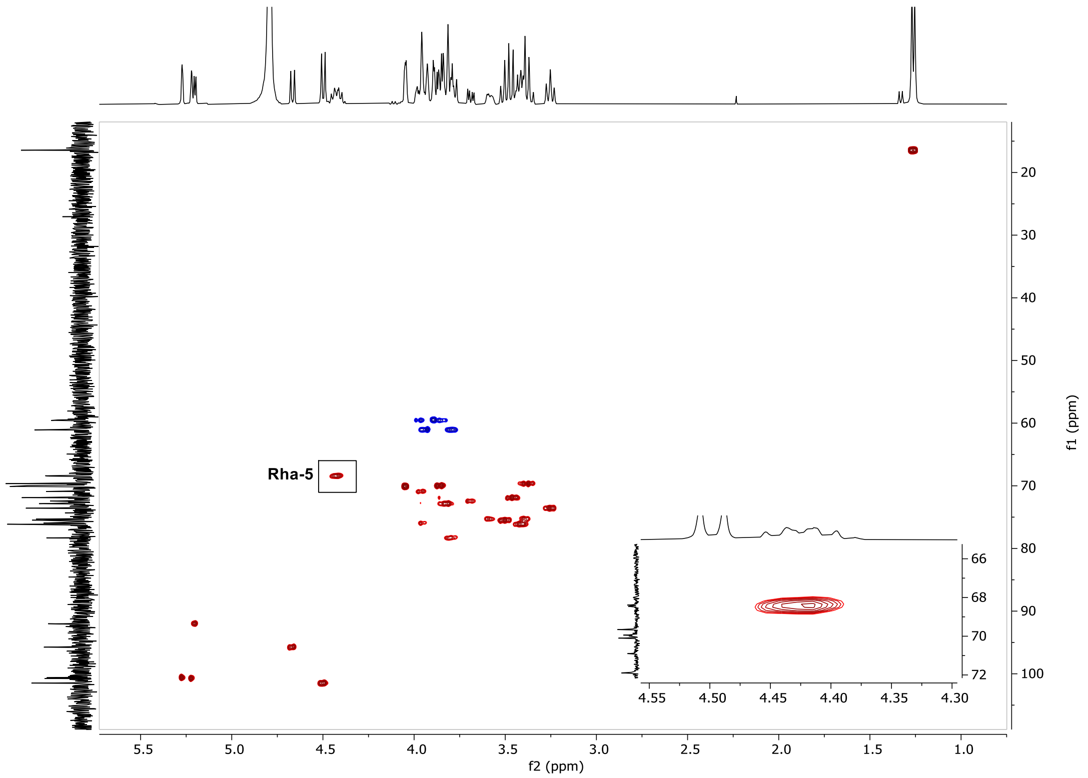

Figure 20 HSQC spectra of **3mer-III** with zoom-in on Rha-5 cross peak.

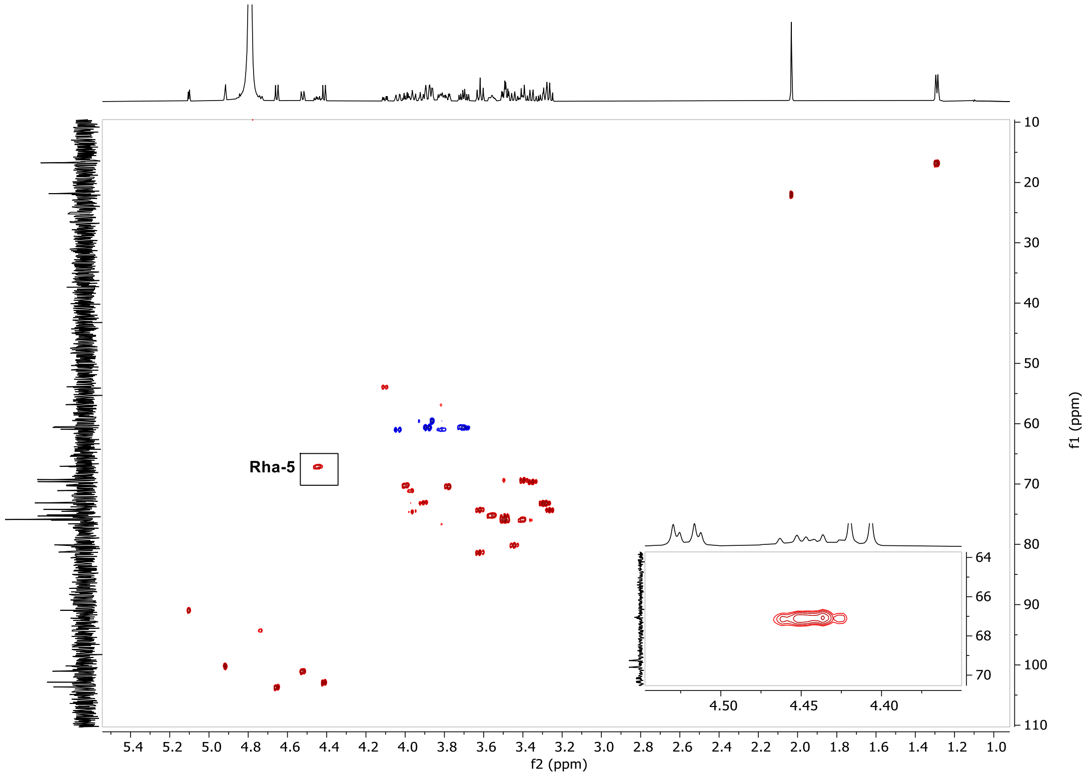

Figure 21 HSQC spectra of **5mer-I** with zoom-in on Rha-5 cross peak.

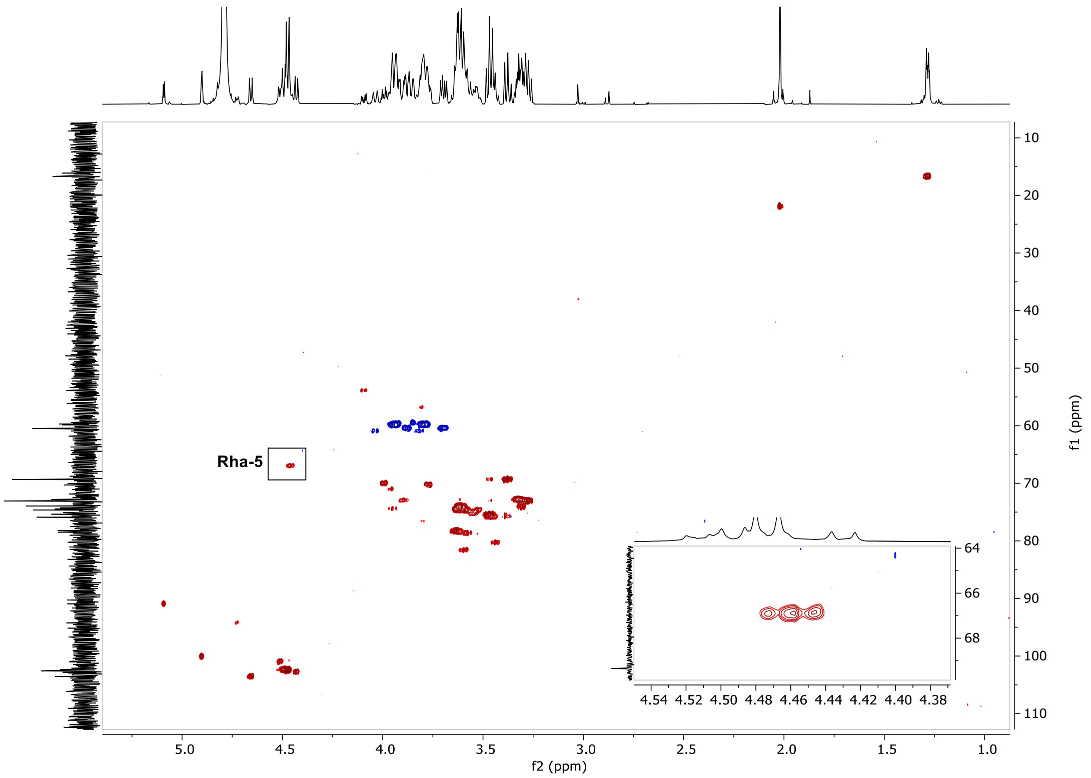

Figure 22 HSQC spectra of **9mer-I** with zoom-in on Rha-5 cross peak.

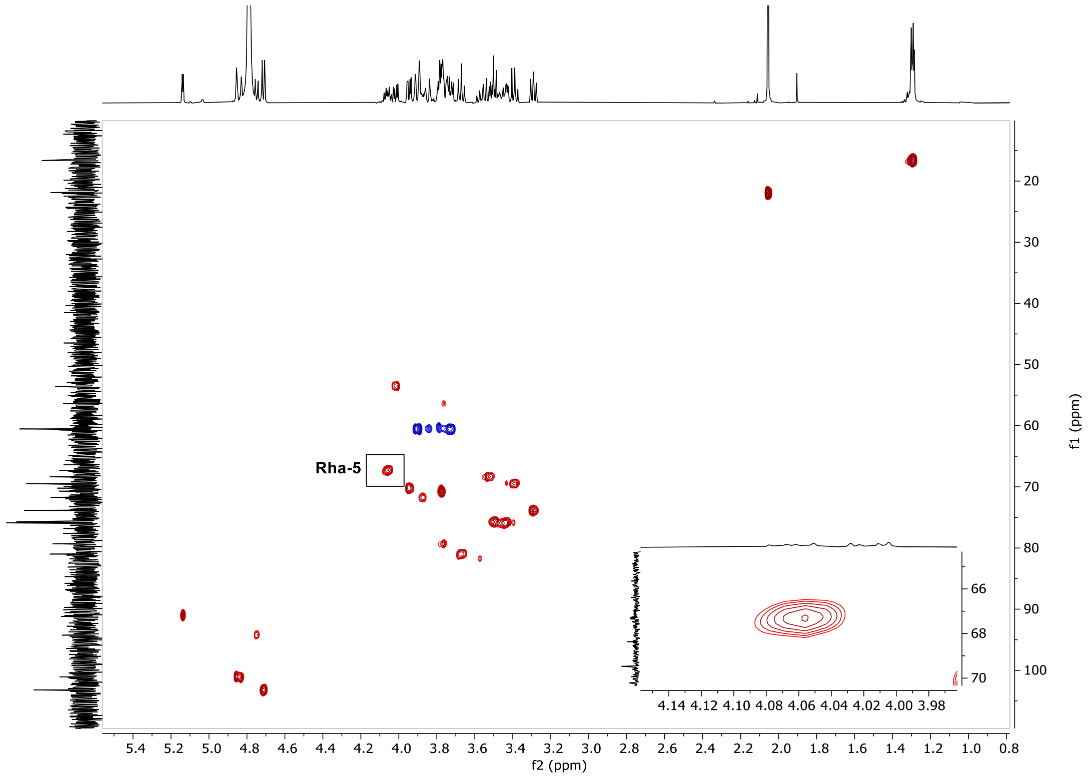

Figure 23 HSQC spectra of **3mer-IV** with zoom-in on Rha-5 cross peak.

Compound	δ Rha-5 (ppm)	Δδ (ppm)*
**3mer-I**	4.42	0.36
**3mer-III**	4.42	0.36
**3mer-IV**	4.06	0
**5mer-I**	4.44	0.38
**9mer-I**	4.46	0.40

Table 15 Comparison of the chemical shifts for Rha-5. ***Δδ** was calculated as follows:

$$\Delta\delta = \delta_{(Rha-5,\boldsymbol{compound})} - \delta_{(Rha-5,\boldsymbol{3mer-IV})}$$

The chemical shift is an indicator for C-H···O hydrogen bond, as previously described for fucosylated motifs such as the Lewis^{X} trisaccharide where the H-5 of Fuc is shifted downfield (Δδ ≈ 0.45 ppm) (Figure 18).[22] An analogous downfield shift (Δδ ≈ 0.3-0.5 ppm) was ascribed to a non-conventional hydrogen bond in galactosaminogalactans analogues.[23] The Rha-5 ^{1}H NMR chemical shift was used to assess the presence of a non-conventional hydrogen bond (C-H···O) involving the H-5 of Rha (Rha-5) and the endocyclic O-5 of the Glc unit. A significant downfield shift (Δδ ≈ 0.3-0.4 ppm, Table 1) was observed for **3mer-I**, **3mer-III**, **5mer-I**, and **9mer-I** (Figures S19, S20, S21, and S22) compared to **3mer-IV** (Figure 23) indicating the presence of a non-conventional hydrogen bond.

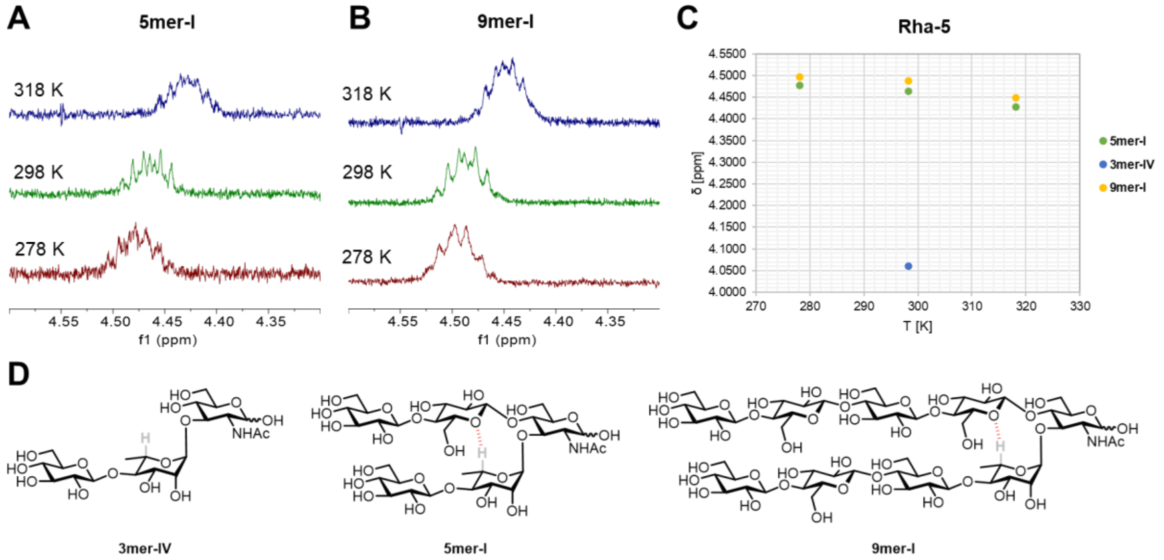

Figure 24 A and B) Temperature dependent ^{1}H NMR analysis of **5mer-I** and **9mer-I**. The chemical shifts for Rha-5 were extracted from selective 1D TOCSY (600 MHz, d9 200 ms, D_2O) at different temperature (*top*). C) Chemical shift of Rha-5 as a function of temperature. A small upfield shift (Δδ = 0.05 ppm) was observed for Rha-5 of **5mer-I** and **9mer-I**. These values remain far from Rha-5 of **3mer-IV**, confirming the stability of the non-conventional hydrogen bond. Negligible variations (Δδ < 0.02 ppm) were detected for all other protons belonging to Glc A, Glc B, Glc C, and Rha monosaccharides. D) Chemical structures of **3mer-IV**, **5mer-I**, and **9mer-I**.

5.3 NMR characterization of 3mer-I

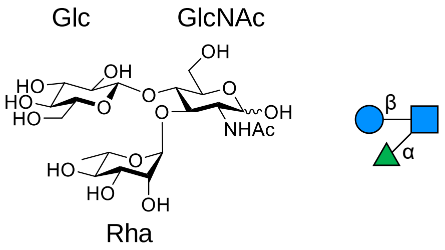

3mer-I

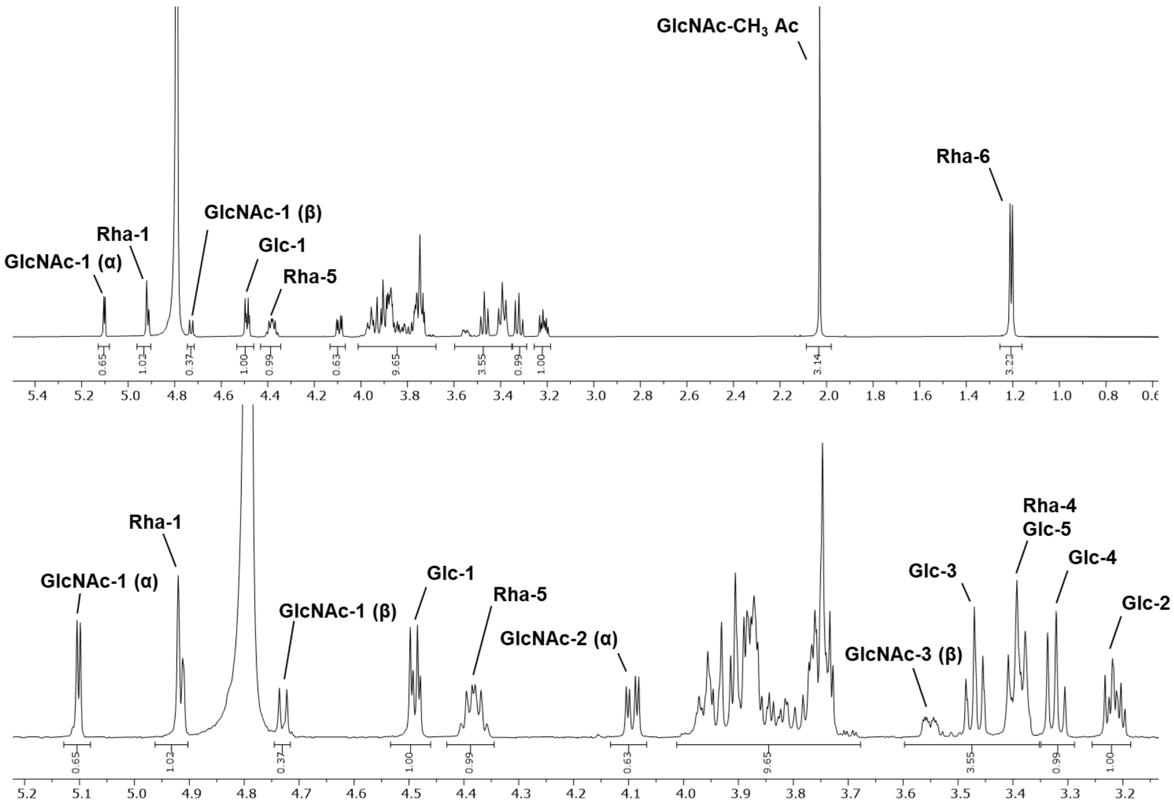

Figure 25 ^{1}H NMR (600 MHz, D_2O) of **3mer-I** with assignments.

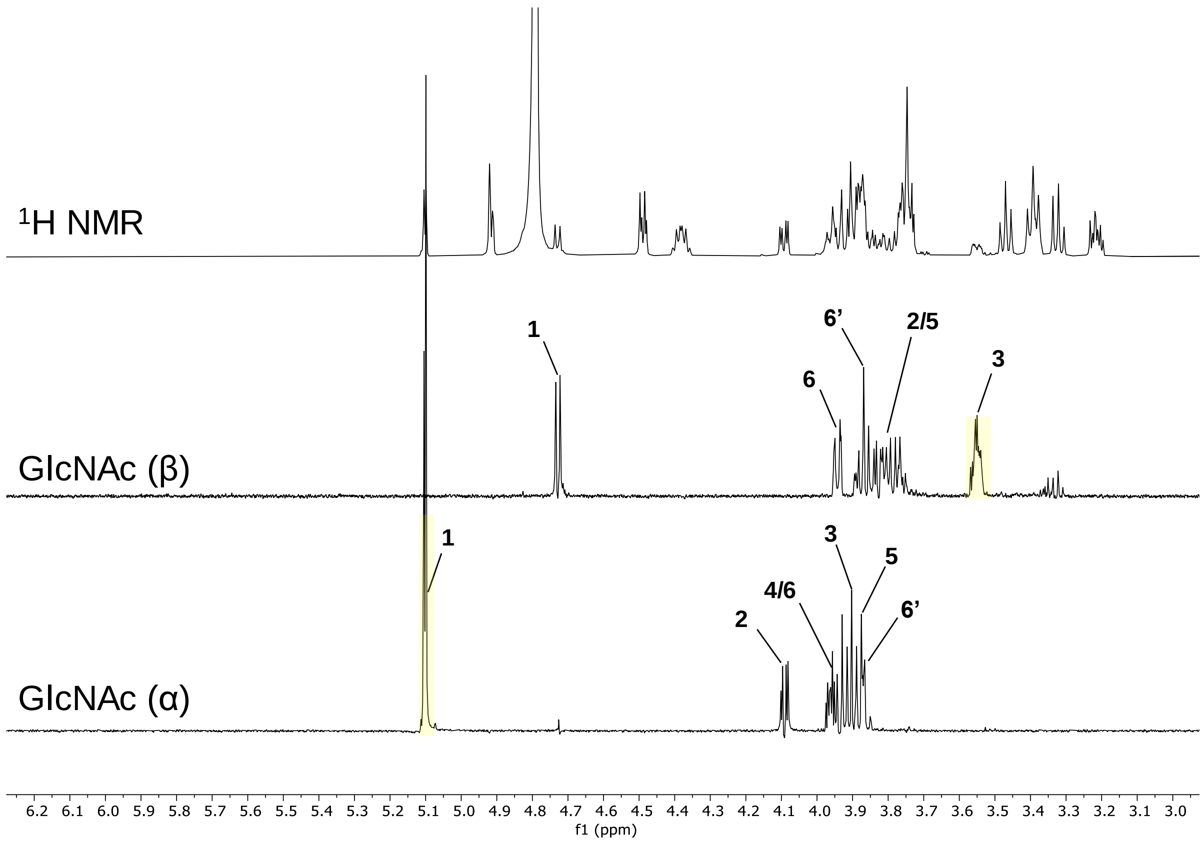

Figure 26 1D TOCSY (700 MHz, d9 200 ms, D_2O) of **3mer-I** with assignments. Resonances chosen for selective excitation are highlighted in yellow.

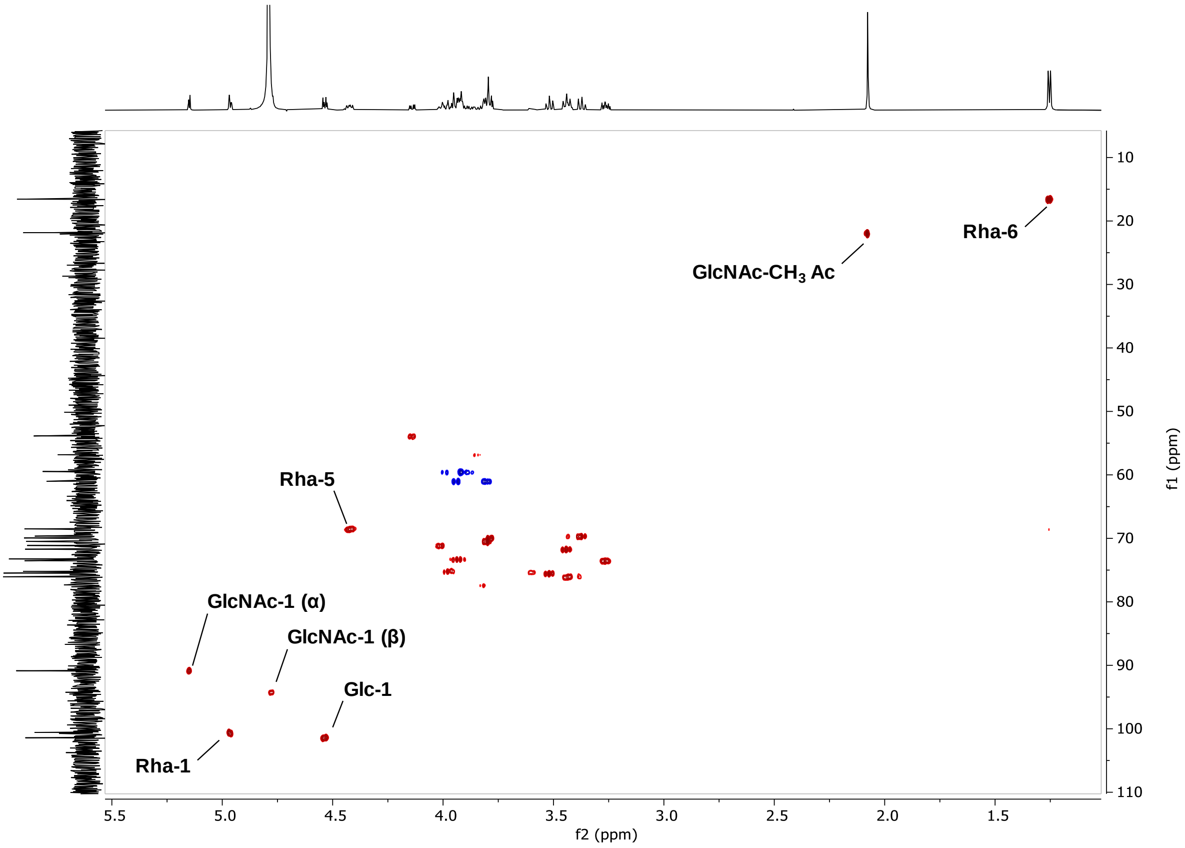

Figure 27 HSQC NMR (D_2O) of **3mer-I** with assignments.

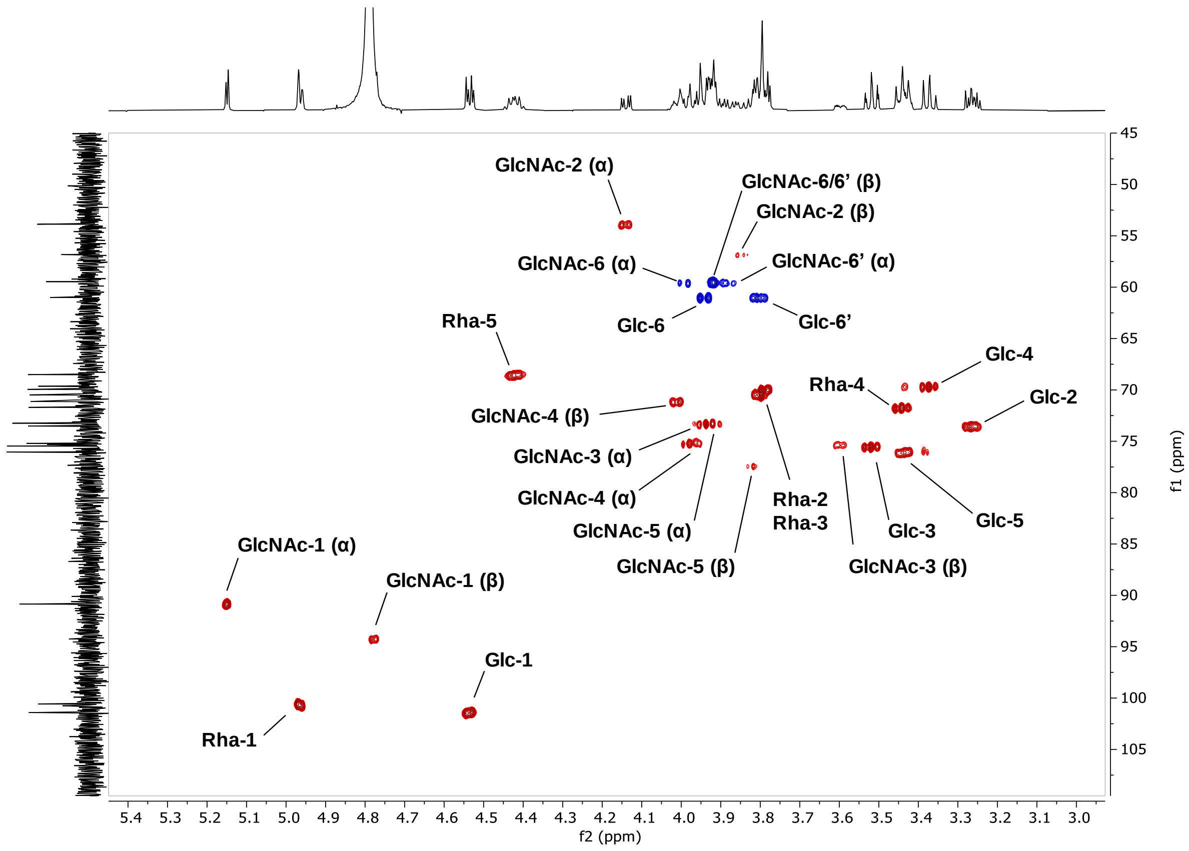

Figure 28 Excerpt of HSQC NMR (D_2O) of **3mer-I** with assignments.

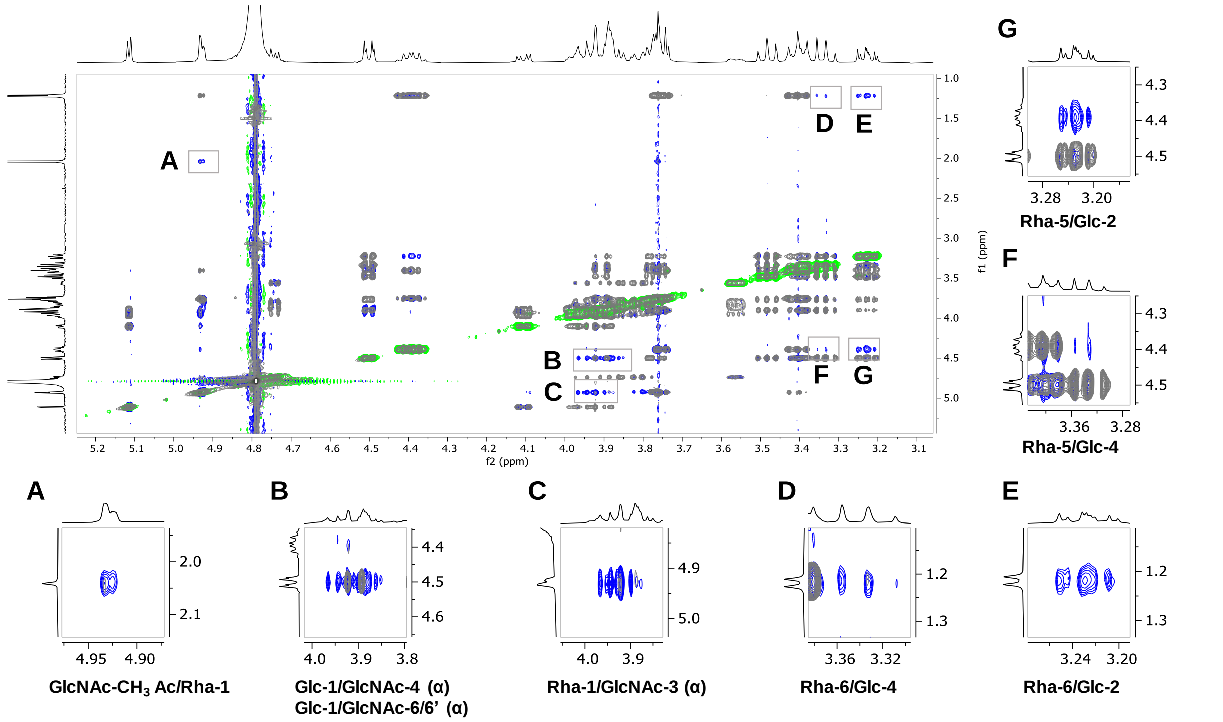

Figure 29 Overimposed 2D NOESY (green-blue, 400 MHz, d8 1000 ms, 295 K, D_2O) of **3mer-I** with assignments and 2D TOCSY (grey, 400 MHz, d9 150 ms, 295 K, D_2O).

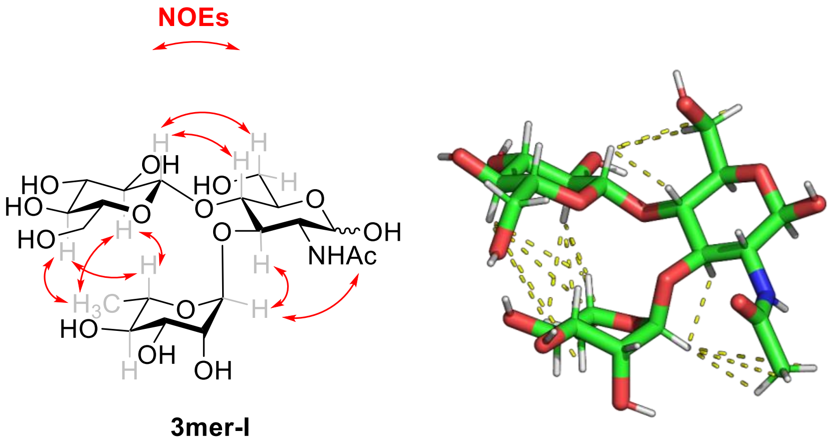

3mer-I

Figure 30 Experimentally observed NOEs (red arrows) and 3D model with NOEs contacts (yellow dashed line).

5.4 NMR characterization of 3mer-II

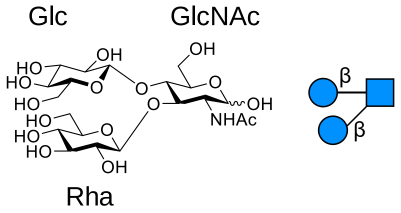

3mer-II

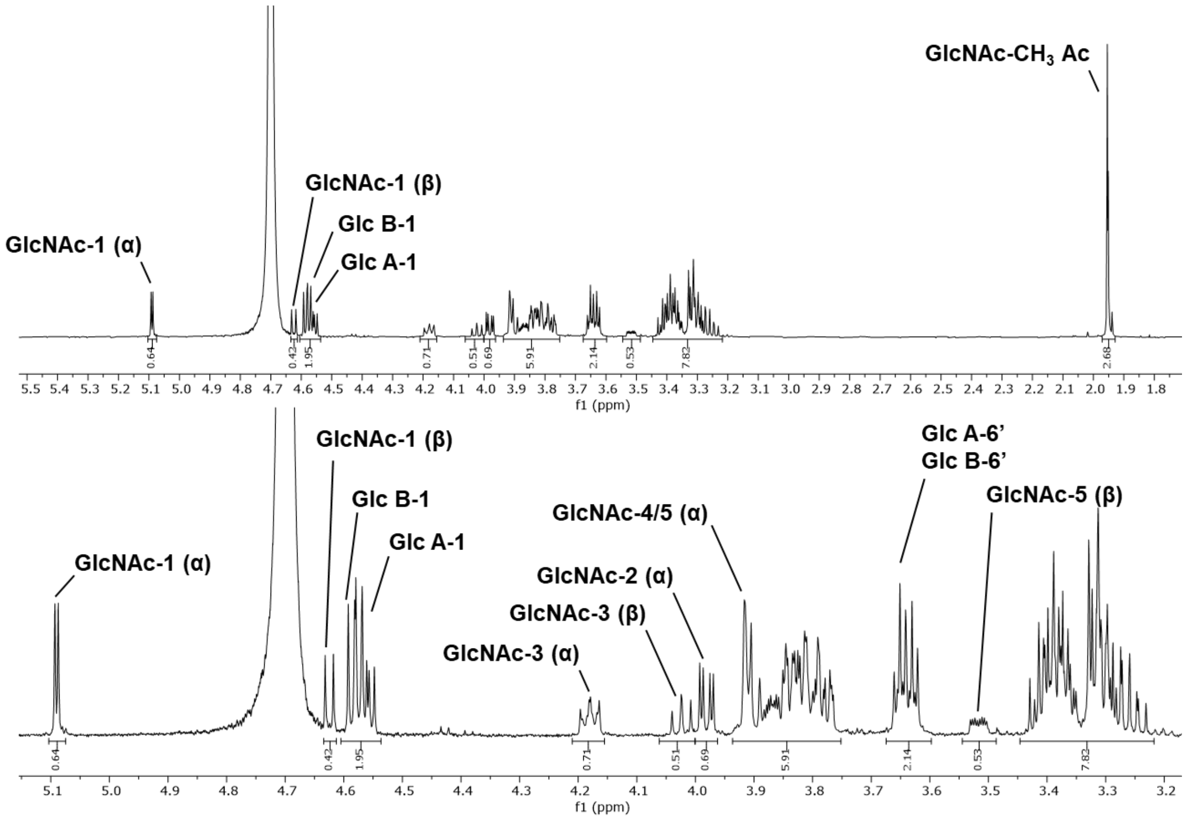

Figure 31 ^{1}H NMR (600 MHz, D_2O) of **3mer-II** with assignments.

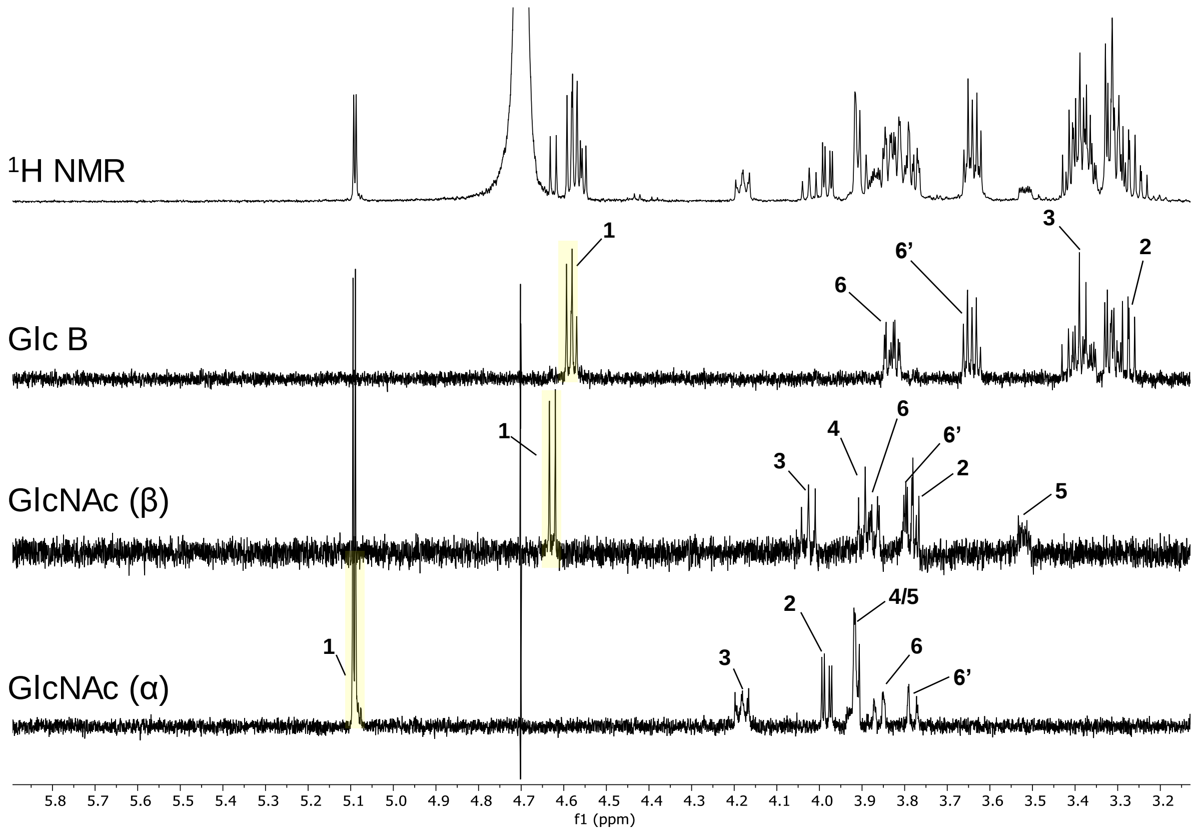

Figure 32 1D TOCSY (600 MHz, d9 200 ms, D_2O) of **3mer-II** with assignments. Resonances chosen for selective excitation are highlighted in yellow.

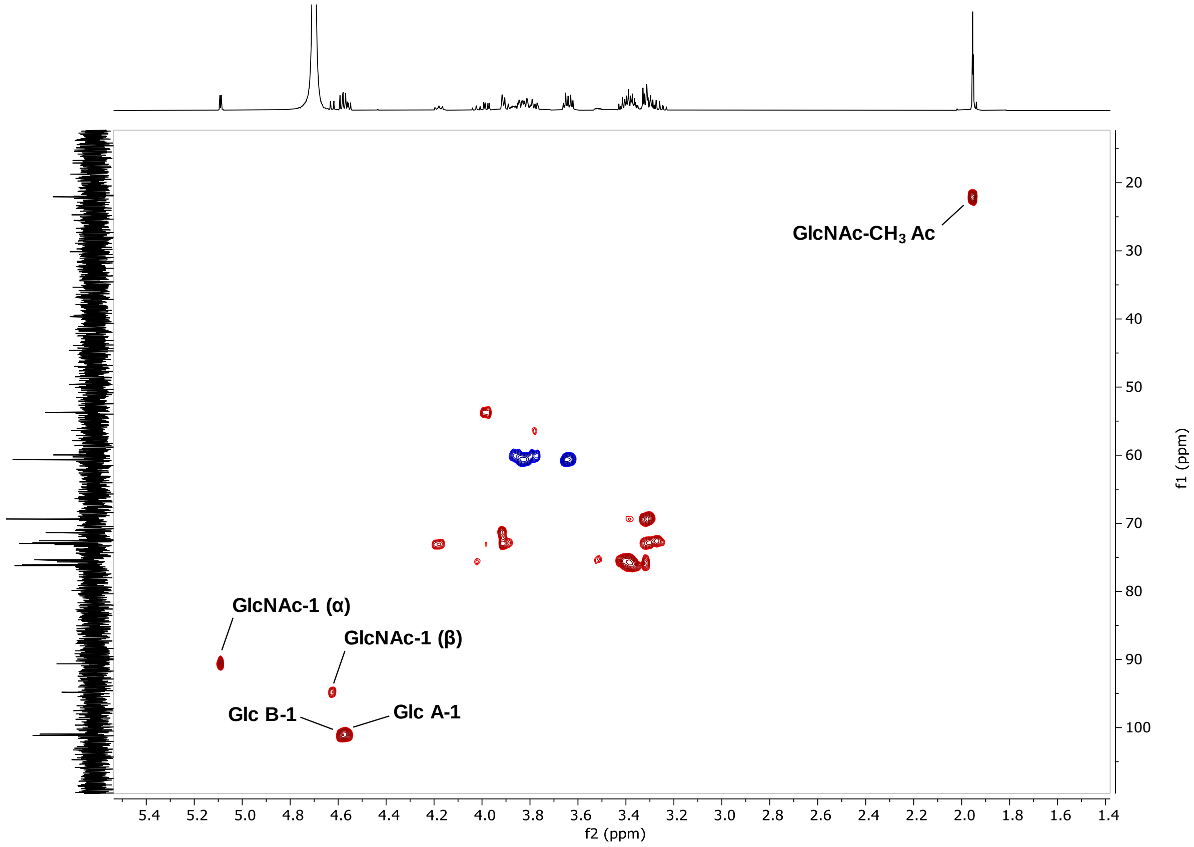

Figure 33 HSQC NMR (D_2O) of **3mer-II** with assignments.

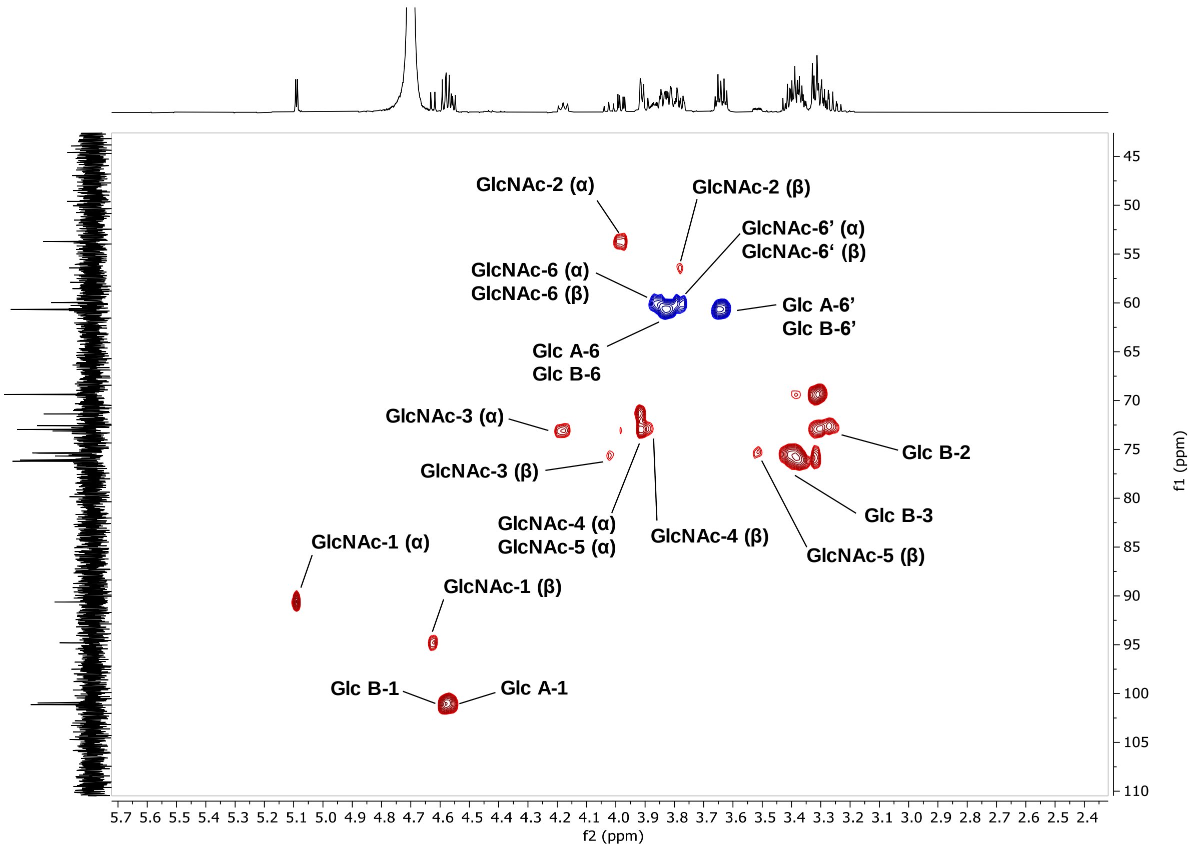

Figure 34 Excerpt of HSQC NMR (D_2O) of **3mer-II** with assignments.

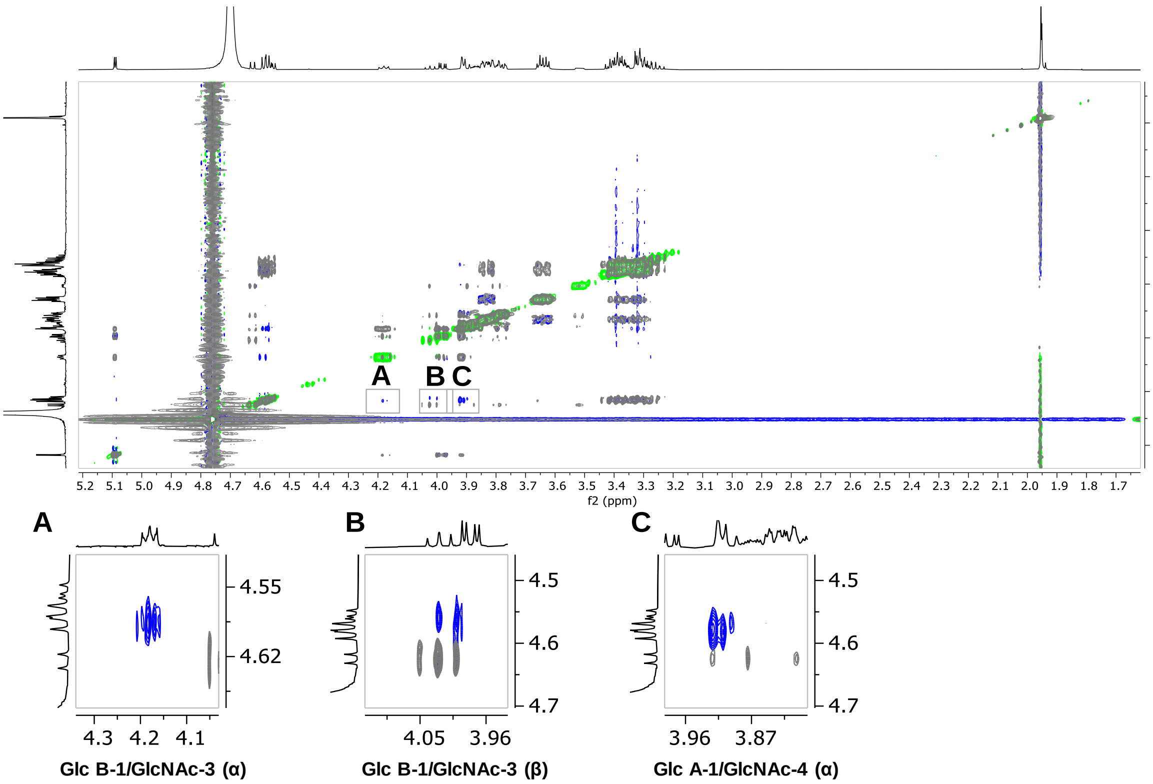

Figure 35 Overimposed 2D NOESY (green-blue, 400 MHz, d8 800 ms, 292 K, D_2O) of **3mer-II** with assignments and 2D TOCSY (grey, 400 MHz, d9 120 ms, 292 K, D_2O).

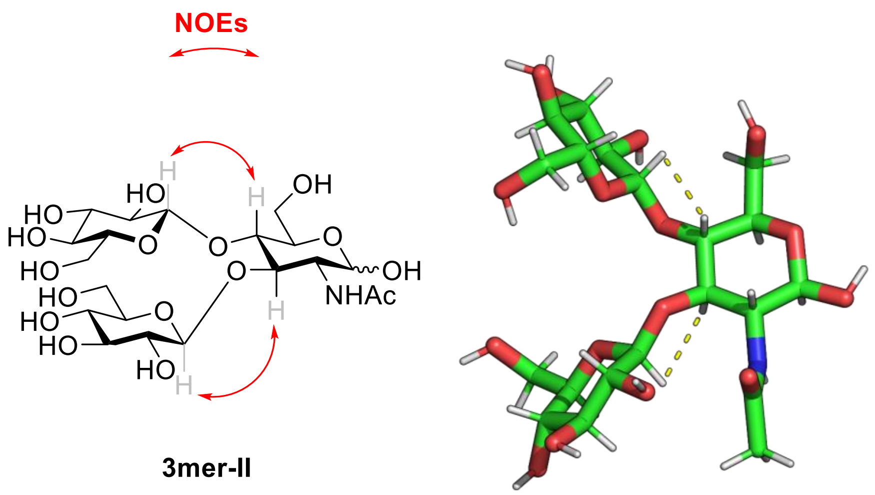

Figure 36 Experimentally observed NOEs (red arrows) and 3D model with NOEs contacts (yellow dashed line).

5.5 NMR characterization of 3mer-III

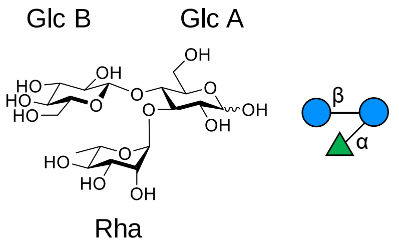

3mer-III

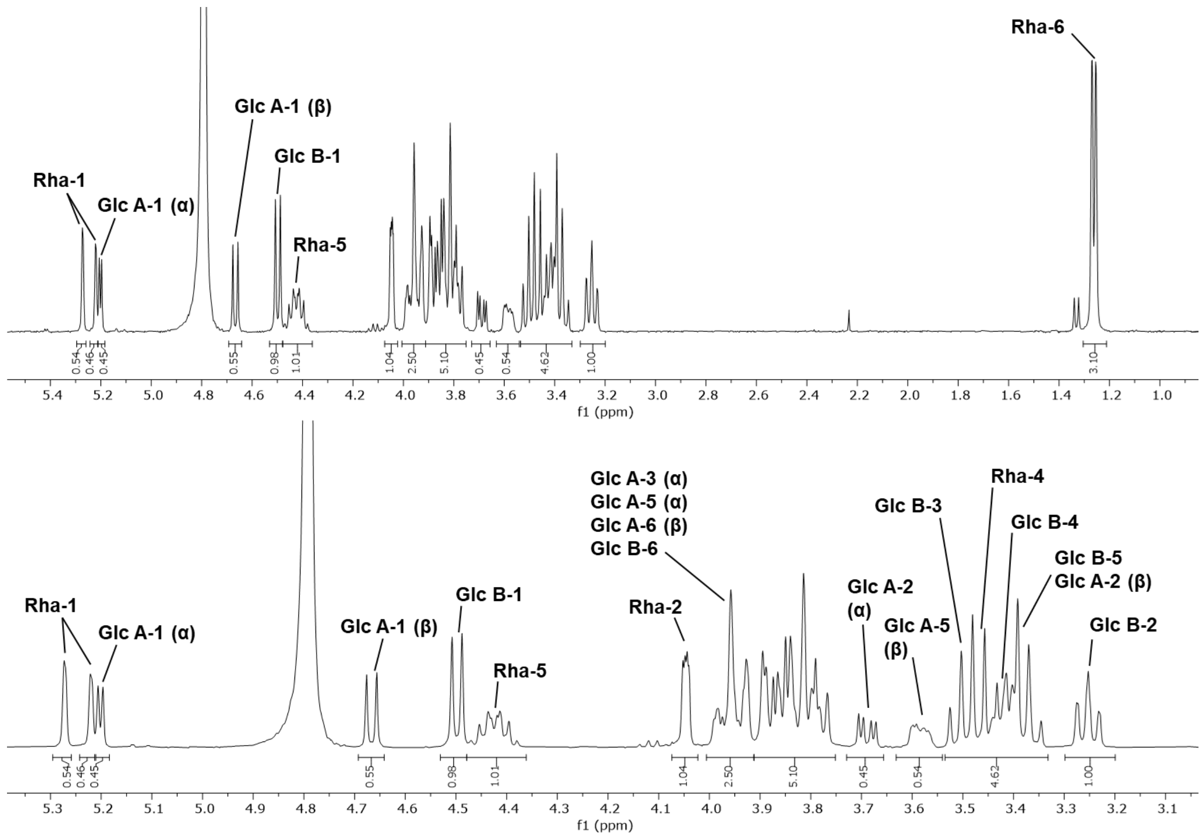

Figure 37 ^{1}H NMR (400 MHz, D_2O) of **3mer-III** with assignments.

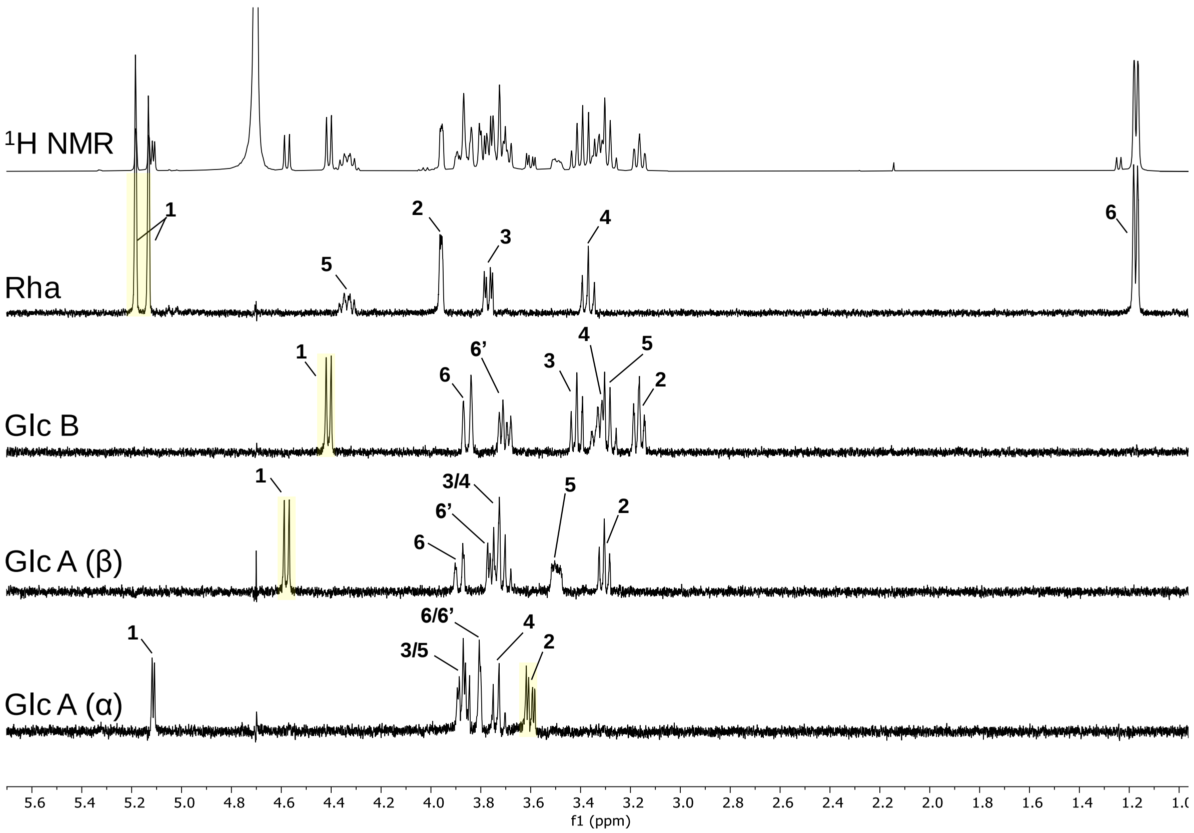

Figure 38 1D TOCSY (400 MHz, d9 200 ms, D_2O) of **3mer-III** with assignments. Resonances chosen for selective excitation are highlighted in yellow.

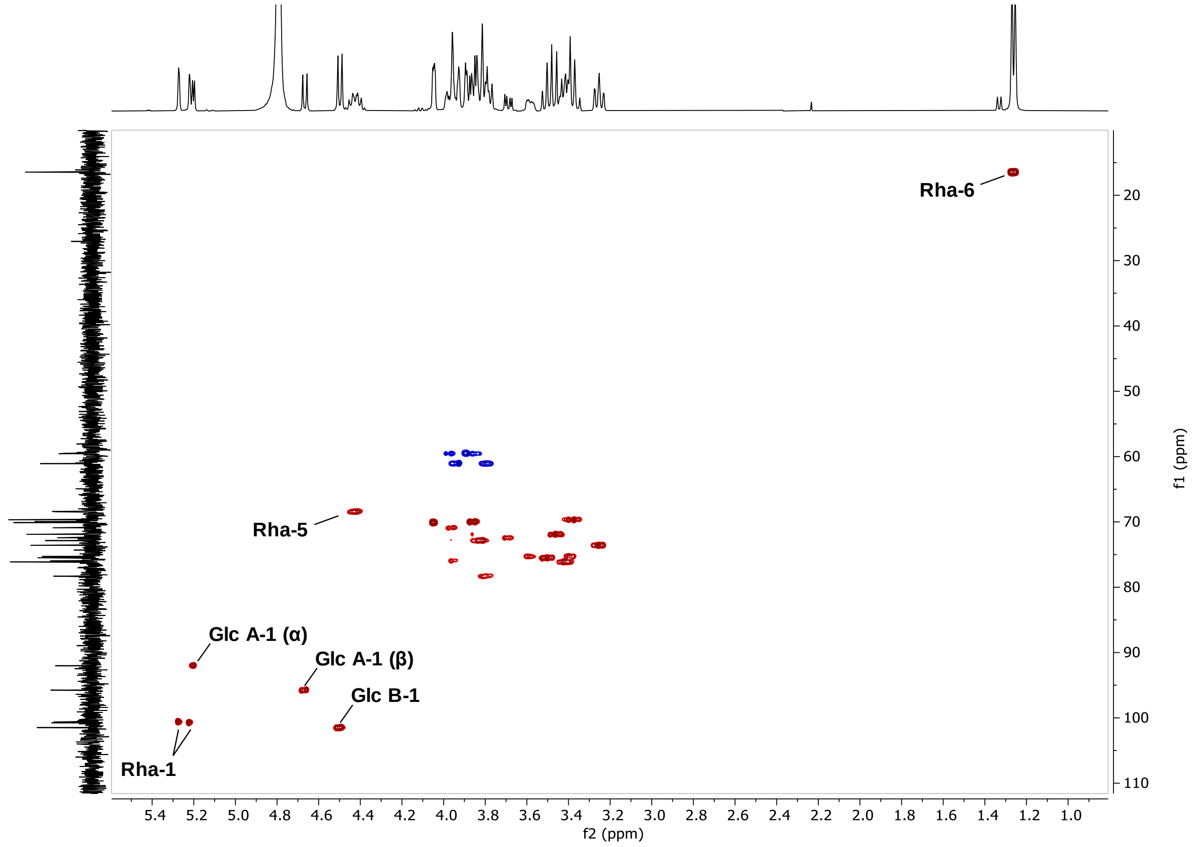

Figure 39 HSQC NMR (D_2O) of **3mer-III** with assignments.

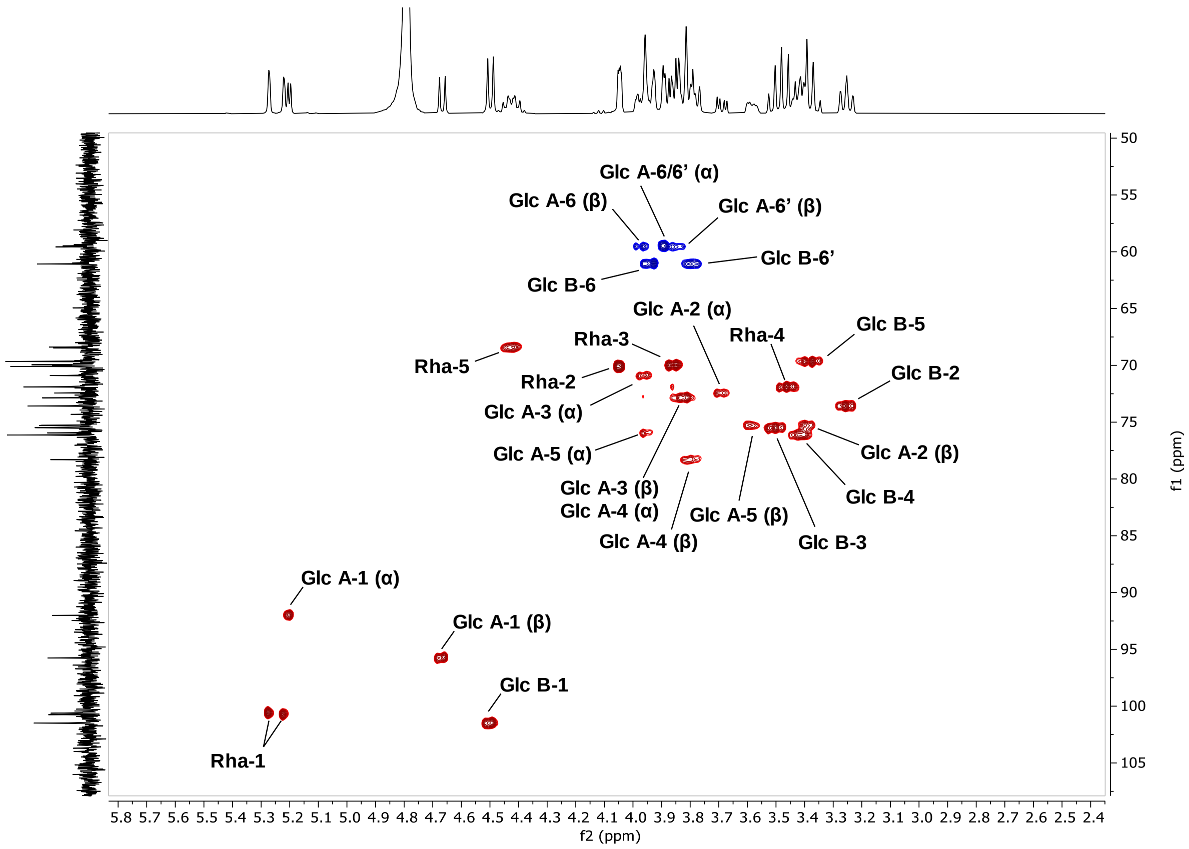

Figure 40 Excerpt of HSQC NMR (D_2O) of **3mer-III** with assignments.

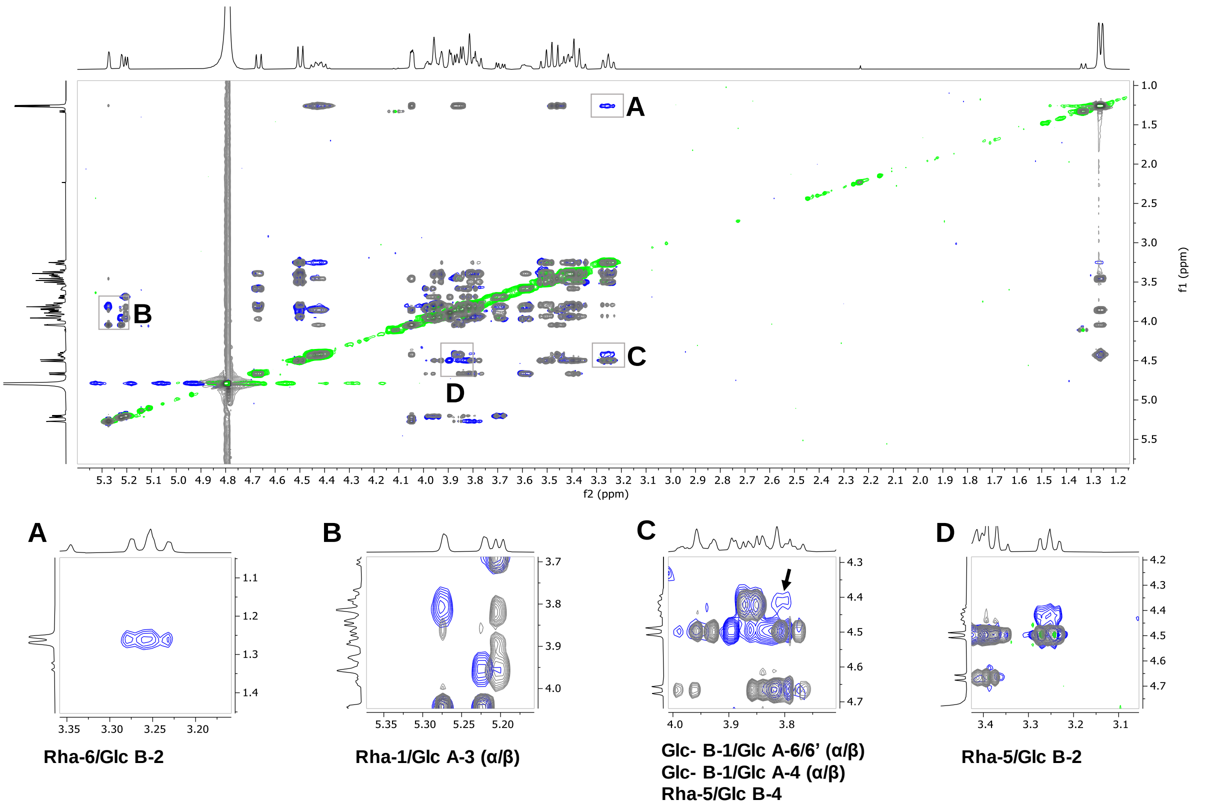

Figure 41 Overimposed 2D NOESY (green-blue, 400 MHz, d8 600 ms, 298 K, D_2O) of **3mer-III** with assignments and 2D TOCSY spectrum (grey, 400 MHz, d9 120 ms, 298 K).

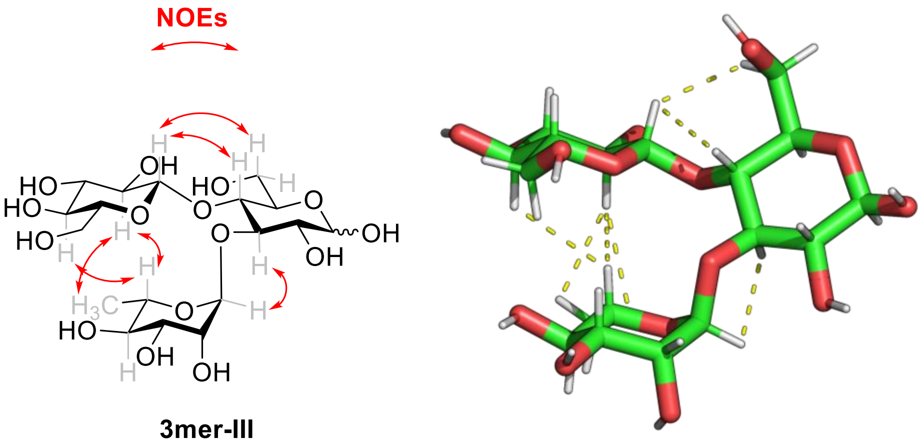

3mer-III

Figure 42 Experimentally observed NOEs (red arrows) and 3D model with NOEs contacts (yellow dashed line).

5.6 NMR characterization of 5mer-I

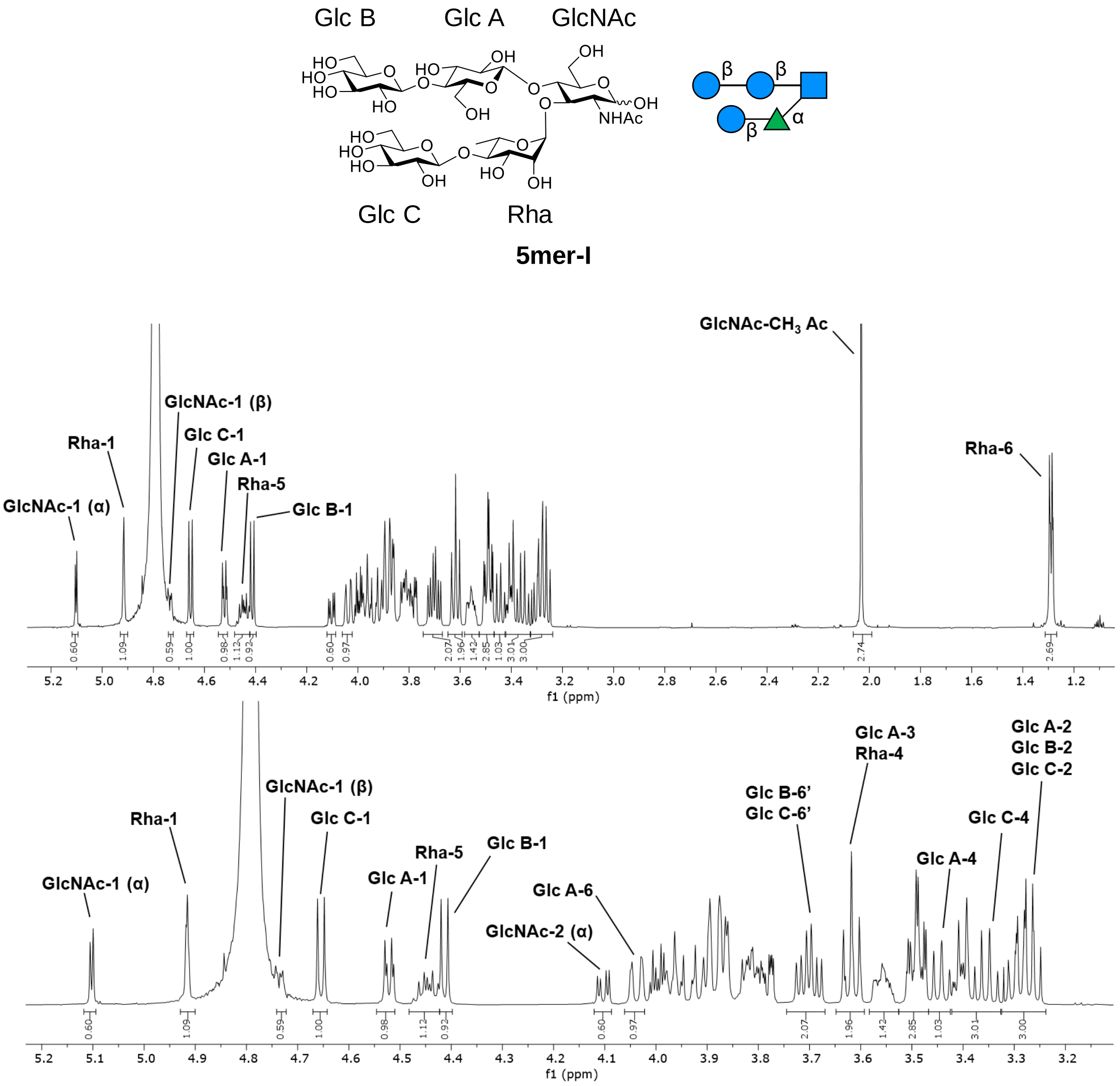

Figure 43 ^{1}H NMR (600 MHz, D_2O) of **5mer-I** with assignments.

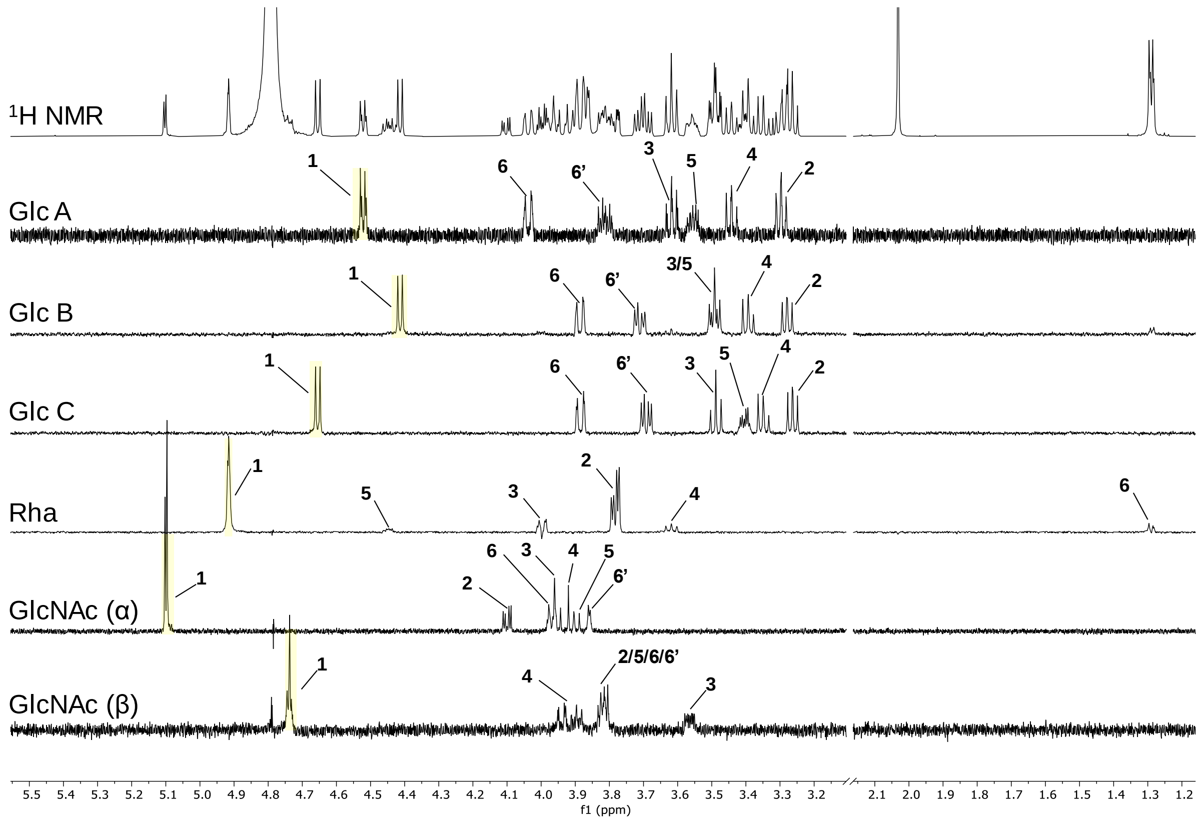

Figure 44 1D TOCSY (600 MHz, d9 200 ms, D_2O) of **5mer-I** with assignments. Resonances chosen for selective excitation are highlighted in yellow.

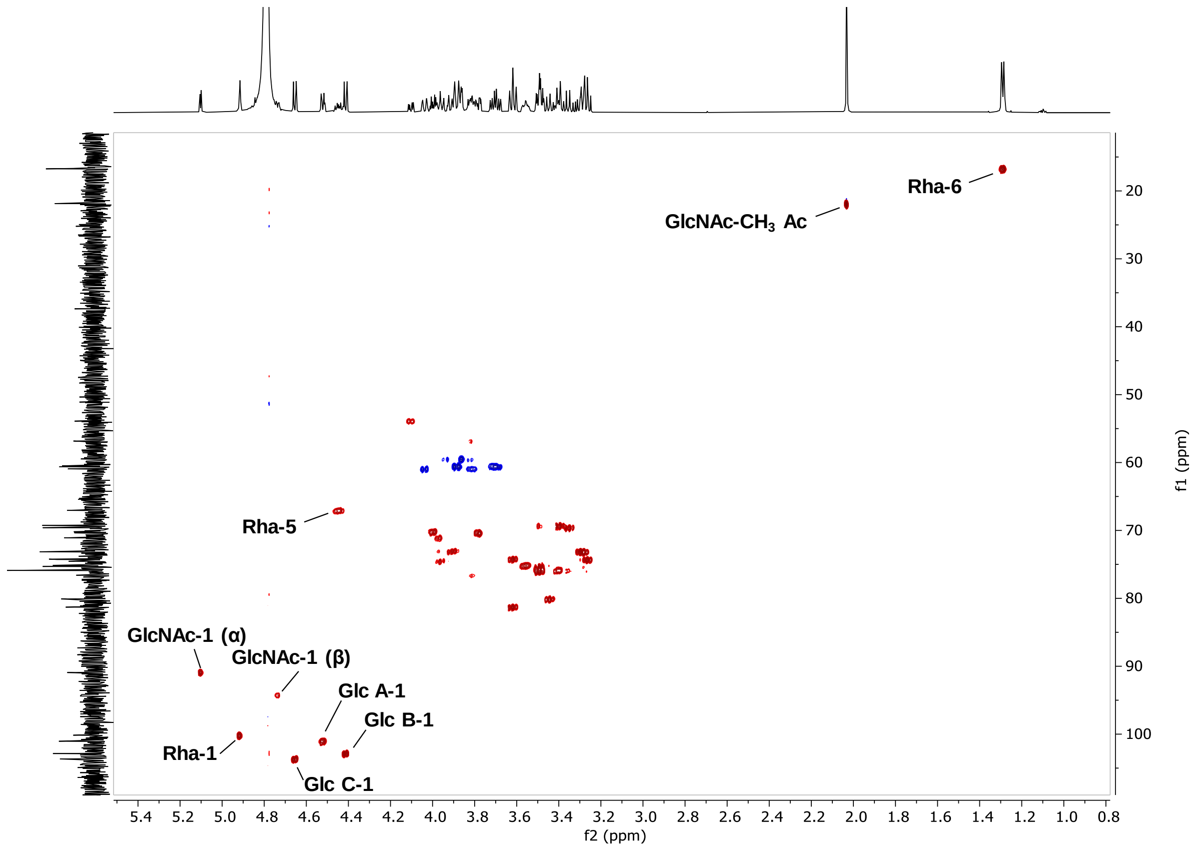

Figure 45 HSQC NMR (D_2O) of **5mer-I** with assignments.

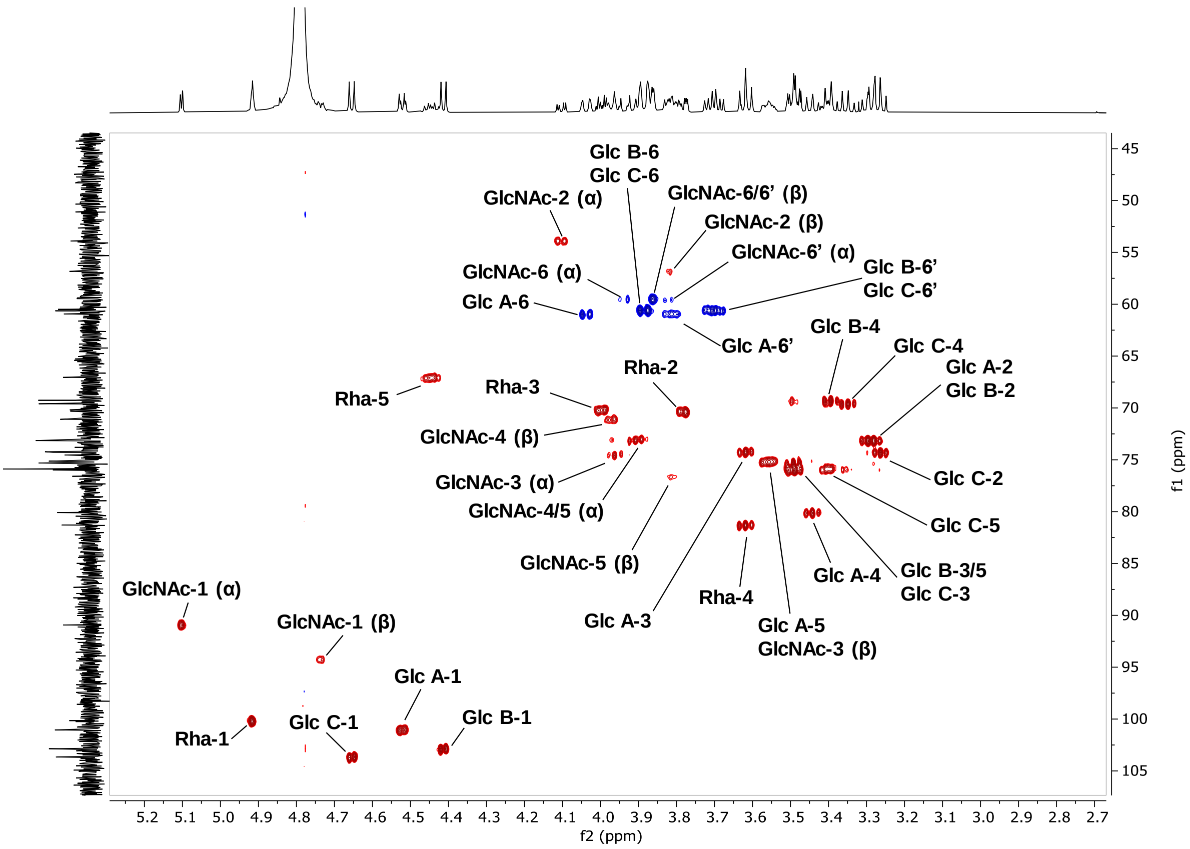

Figure 46 Excerpt of HSQC NMR (D_2O) of 5**mer-I** with assignments.

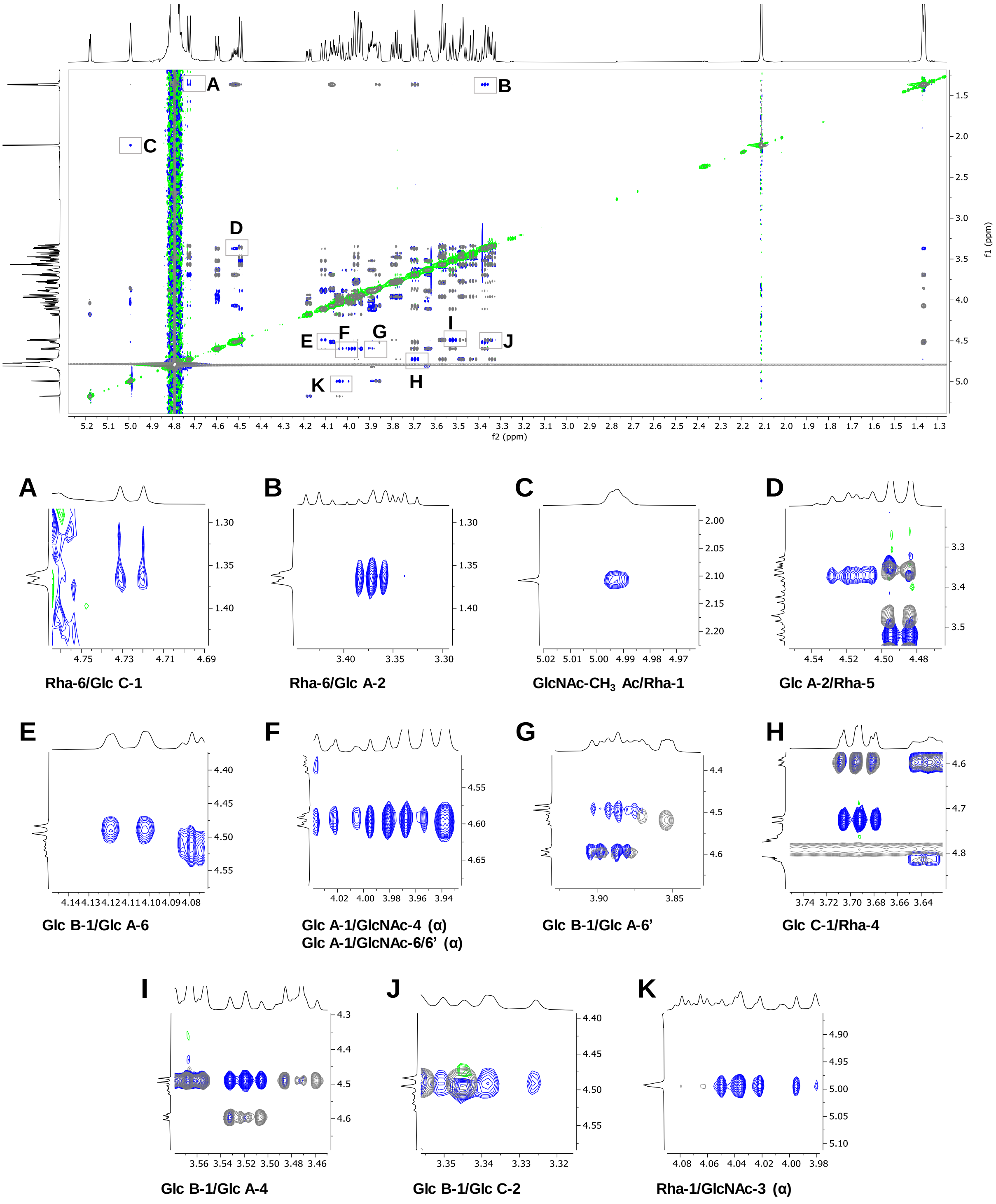

Figure 47 Overimposed 2D ROESY (green-blue, 600 MHz, p15 300 ms, 300 K, D_2O) of **5mer-I** with assignments and 2D TOCSY spectrum (grey, 400 MHz, d9 120 ms, 300 K).

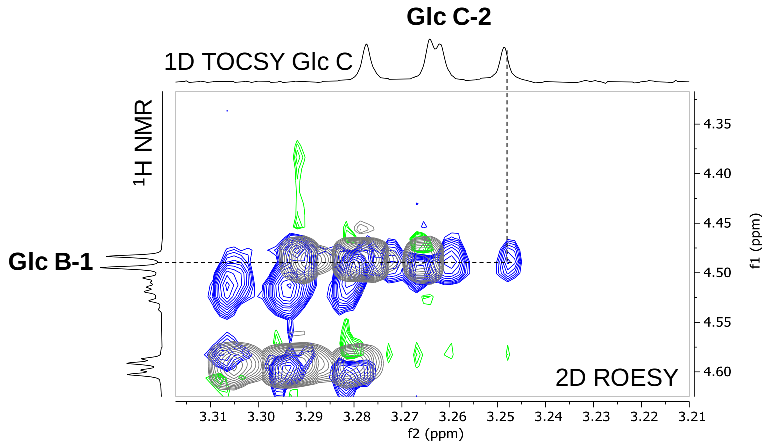

Figure 48 Excerpt of 2D ROESY (green-blue) and 2D TOCSY of **5mer-I** overimposed (grey) (same as **Figure 47**). The horizontal trace presents the 1D TOCSY with selective excitation of Glc C-1. The NOE cross peak is marked with a dashed line.

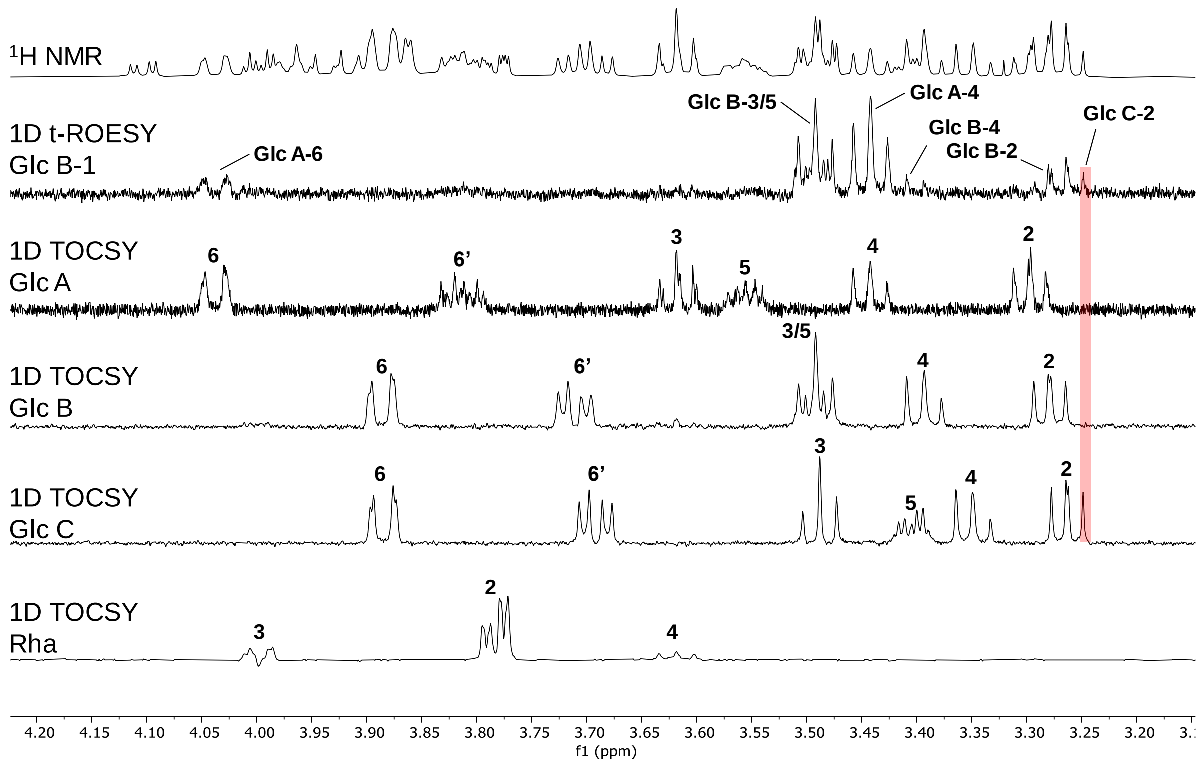

Figure 49 Overlay of 1D t-ROESY (600 MHz, p15 200 ms, 294 K, D_2O) and 1D TOCSY of **5mer-I**. The 1D t-ROESY was obtained by selective excitation of the Glc B-1 resonance (δ 4.41 ppm). The key NOE between Glc B-1/Glc C-2 is highlighted in a red box.

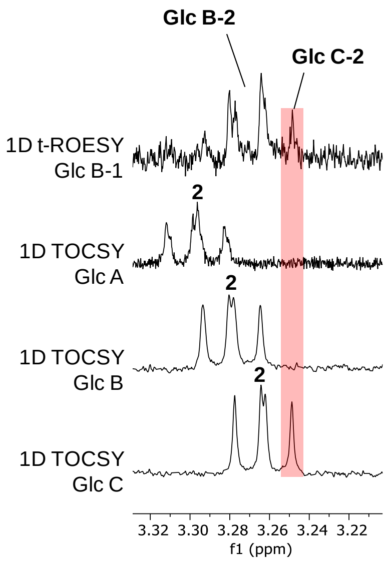

Figure 50 Excerpt of the overlay of 1D t-ROESY (600 MHz, p15 200 ms, 294 K, D_2O) and 1D TOCSY of **5mer-I**. The 1D t-ROESY was obtained by selective excitation of the Glc B-1 resonance.

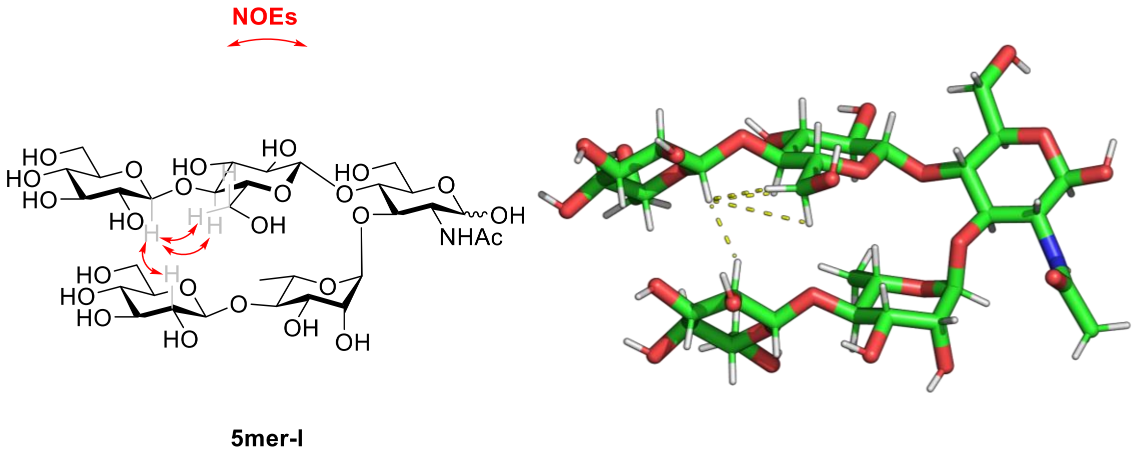

Figure 51 Selected inter-residue NOEs (red arrows) derived from Figure 46 for **5mer-I**.

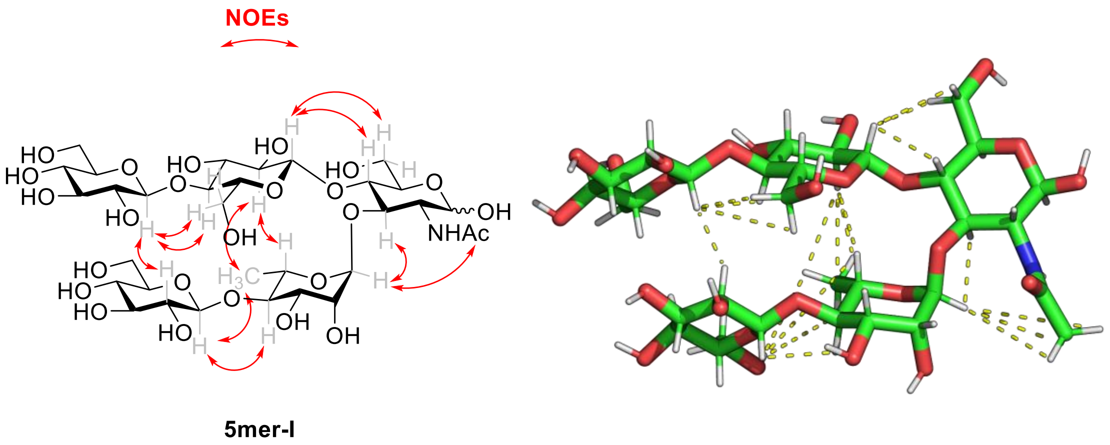

Figure 52 All experimentally observed NOEs (red arrows) and 3D model with NOEs contacts (yellow dashed lines).

5.7 NMR characterization of 9mer-I

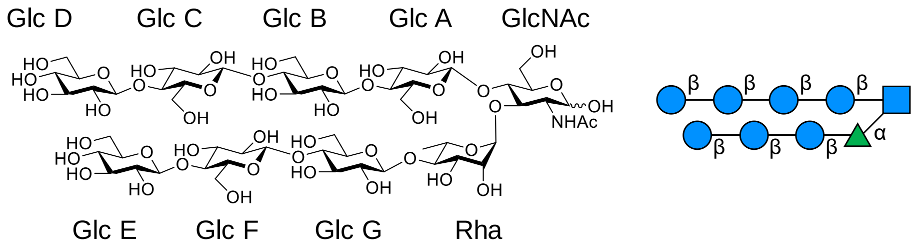

9mer-I

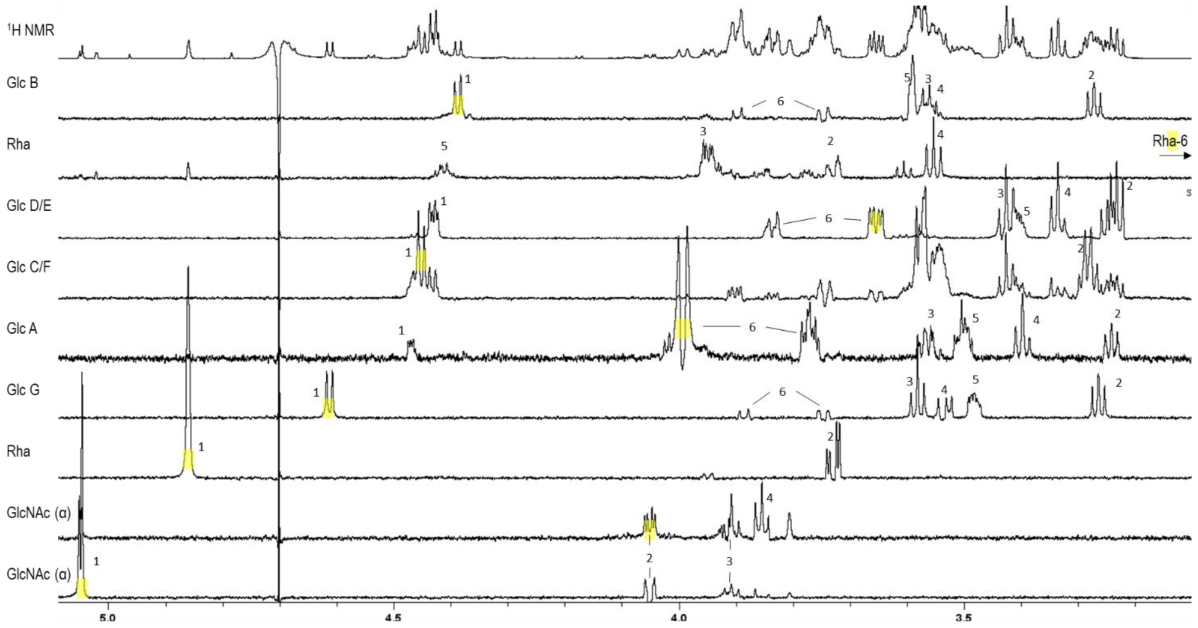

Figure 53 Selective 1D-TOCSY (800 MHz, mixing time 100 ms, D_2O, 298 K) of **9mer-I** with assignments. Resonances chosen for selective excitation are highlighted in yellow.

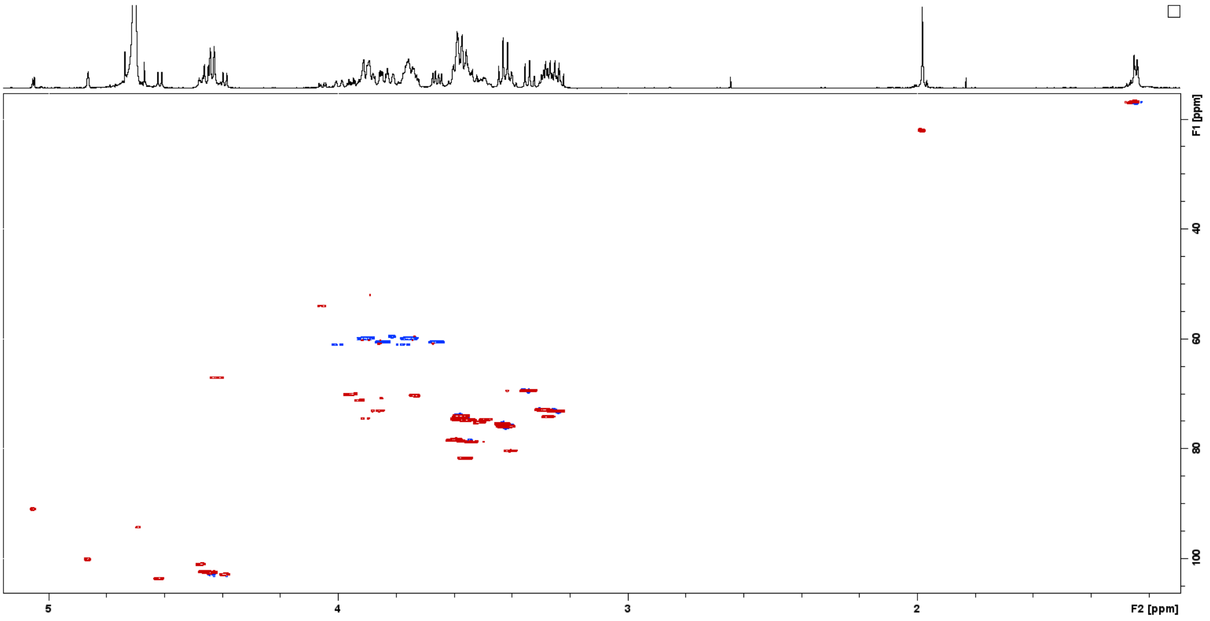

Figure 54 ^1H-^{13}C HSQC (800 MHz, D_2O, 298 K) of **9mer-I**.

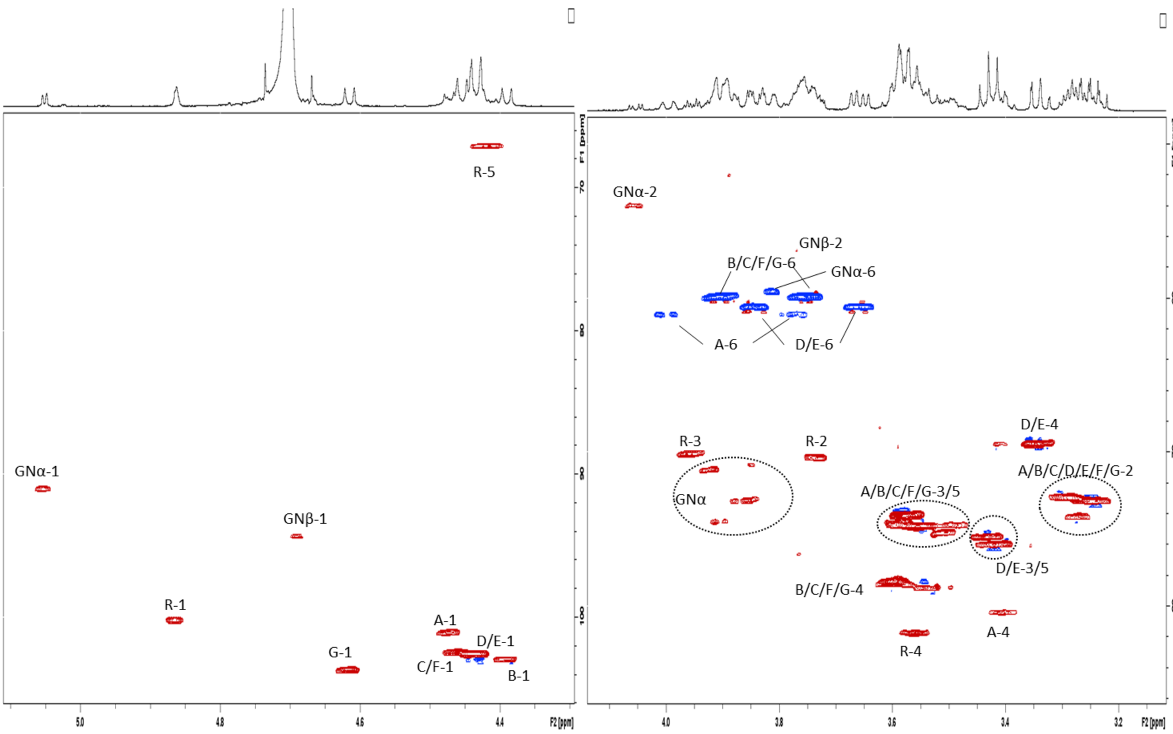

Figure 55 Selected regions of ^1H-^{13}C HSQC (800 MHz, D_2O, 298 K) of **9mer-I** with assignment.

5.8 NMR characterization of 9mer-I-B

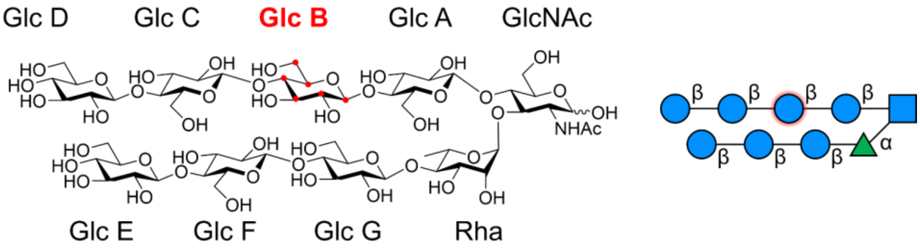

9mer-I-B

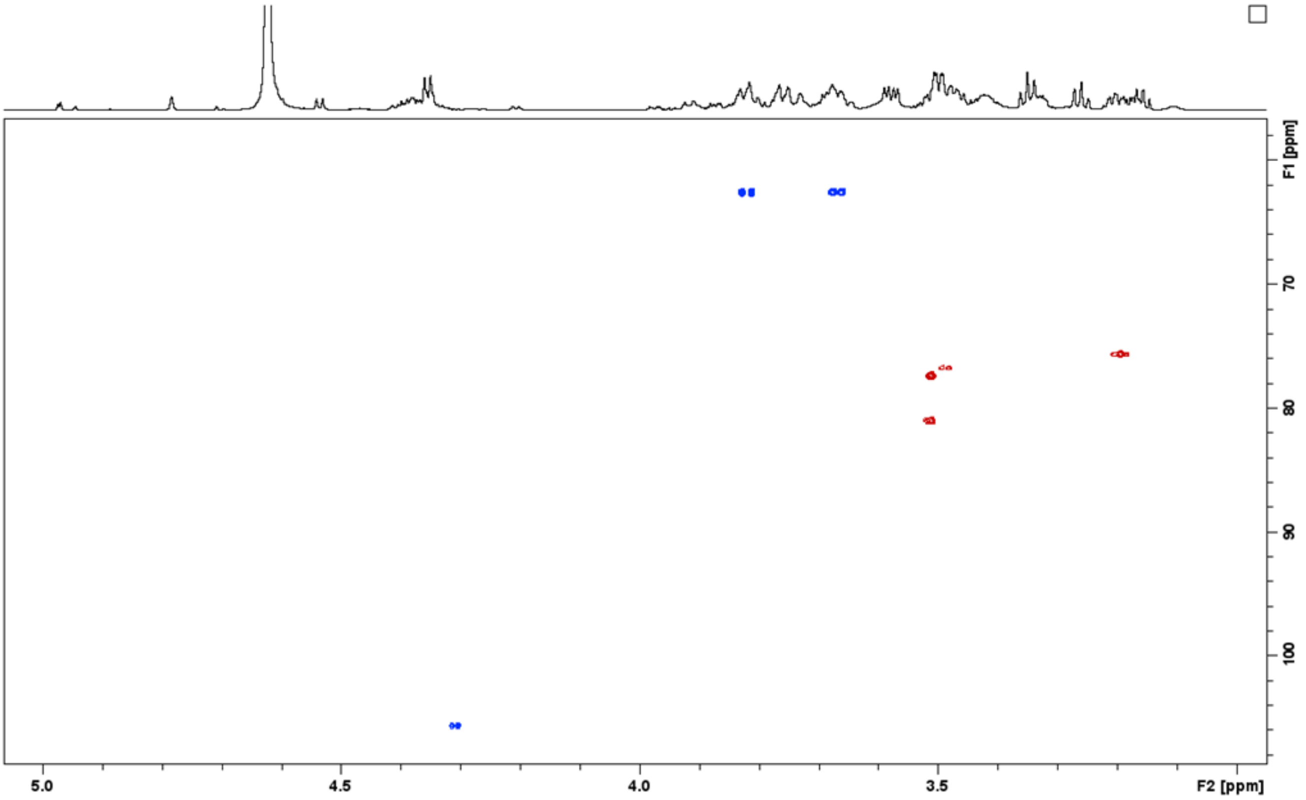

Figure 56 HSQC spectrum of **9mer-I-B** (800 MHz, D_2O, 298 K)**.** Only the cross peaks for the ^{13}C-labeled residue B are visible at this intensity level.

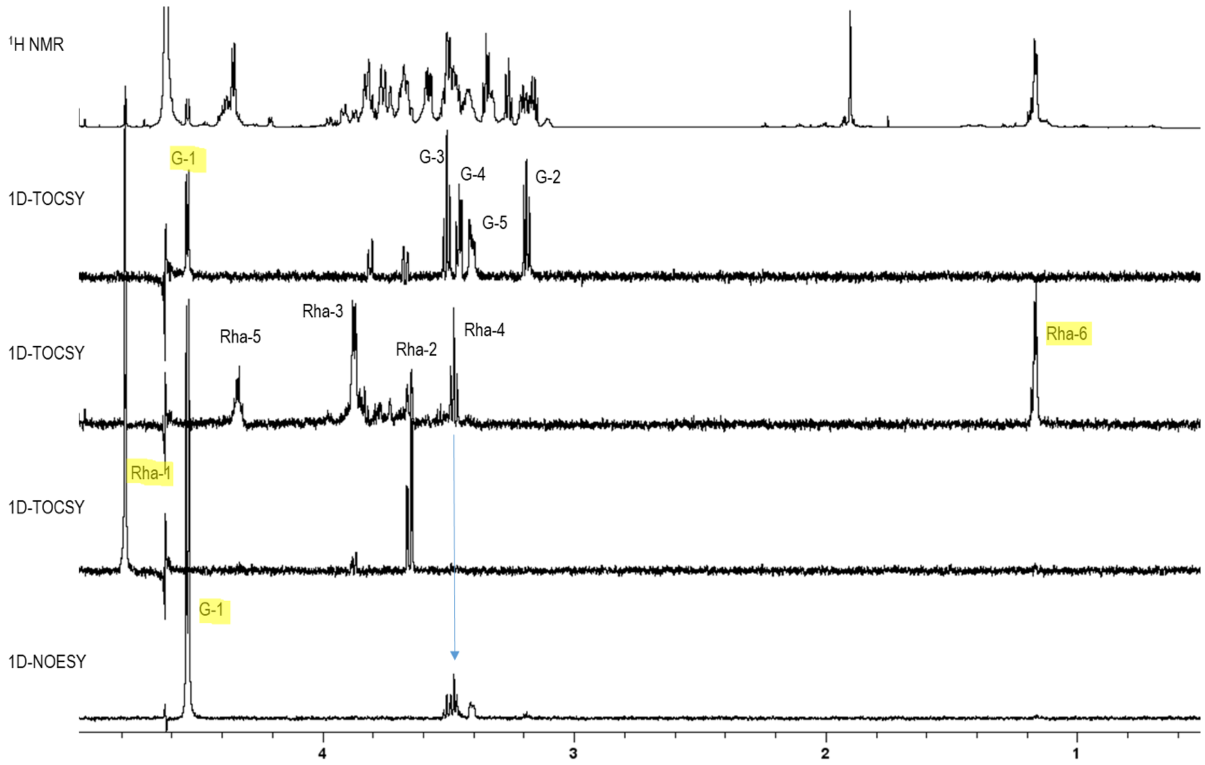

Figure 57 Identification of the G residue for **9mer-I-B** by its glycosylation linkage to Rha-4 position: NOE G-1/Rha-4. Resonances chosen for selective excitation are highlighted in yellow.

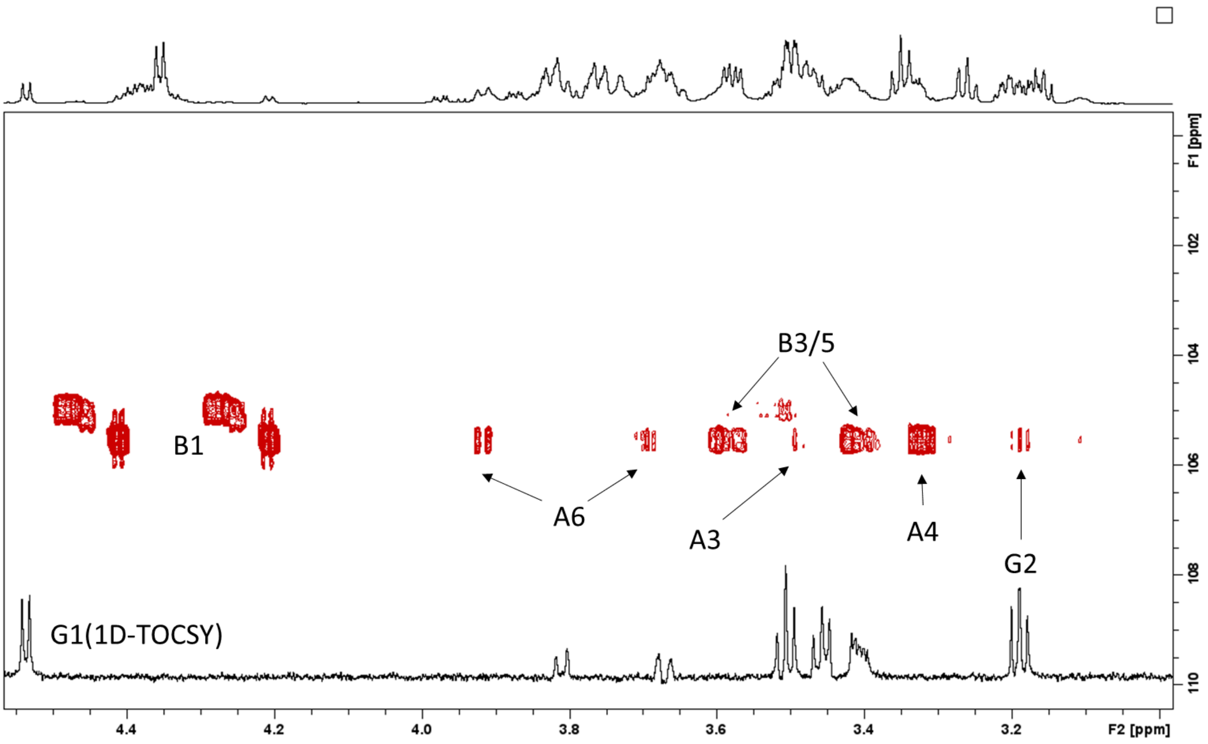

Figure 58 Section of the HSQC-NOESY of **9mer-I-B**, anomeric region. The NOEs from B1 are highlighted. Besides the intra-residue NOEs and those to the vicinal A residue, the inter-chain NOE B-1/G-2 is visible. (Acquisition: TD (F2xF1)=6K x 384 points, SW(F2xF1)=12x60 ppm, 298 K, mixing time 300 ms, D_2O, 800 MHz. Processing: SI(F2xF1)=16K x 2K, QSIN (ssb=2.8/3))

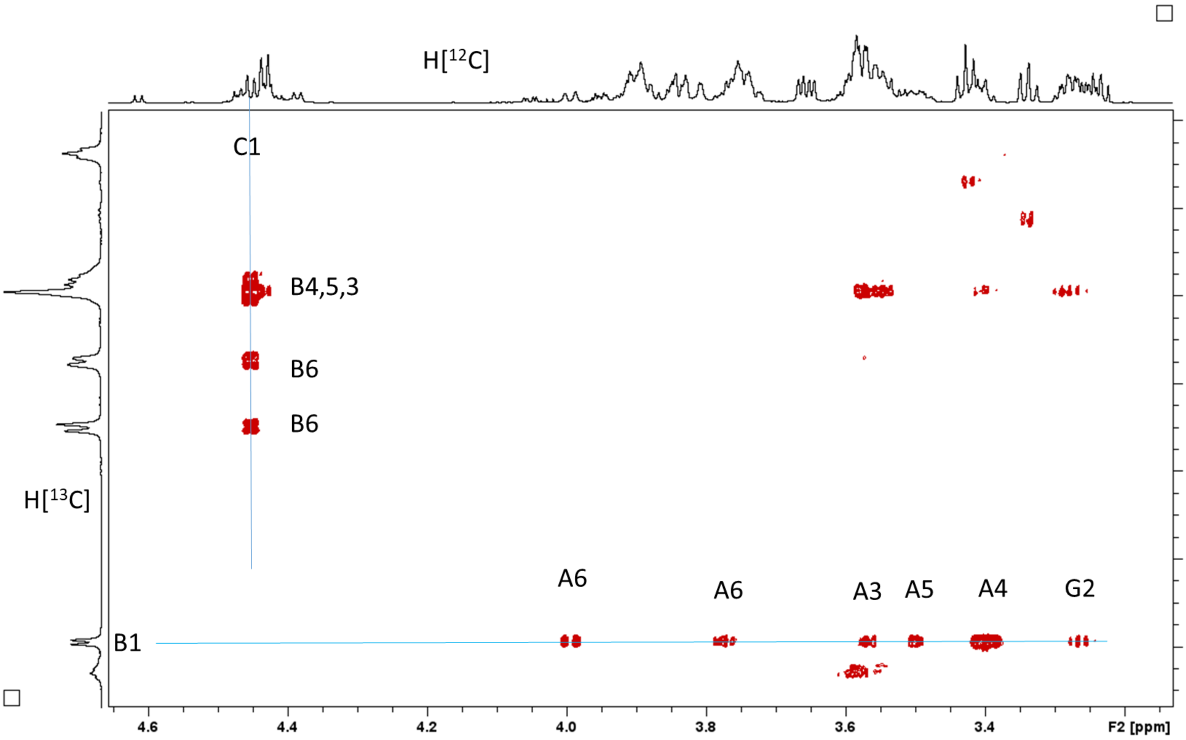

Figure 59 Half-filtered $^1H[^{13}C]$-$^1H[^{12}C]$ NOESY of **9mer-I-B**. The NOEs from B1 (^{13}C-labelled) are highlighted. Besides the inter-residue NOEs to the vicinal A residue, the inter-chain NOE B-1/G-2 is also visible. In this spectrum the intra-residue NOEs are not visible since only NOEs to ^1H-^{12}C protons are observed from the ^1H-^{13}C-labelled B residue. Acquisition: TD (F2xF1)= 4K x 512 points, SW(F2xF1)=8 x 4ppm, 298 K, mixing time 300 ms, D_2O, 800 MHz. Processing: SI(F2xF1)= 4K x 1K, QSIN (ssb=2/2).

5.9 NMR characterization of 9mer-I-F

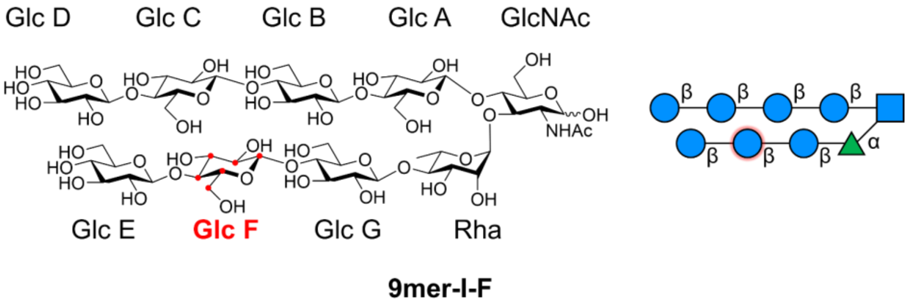

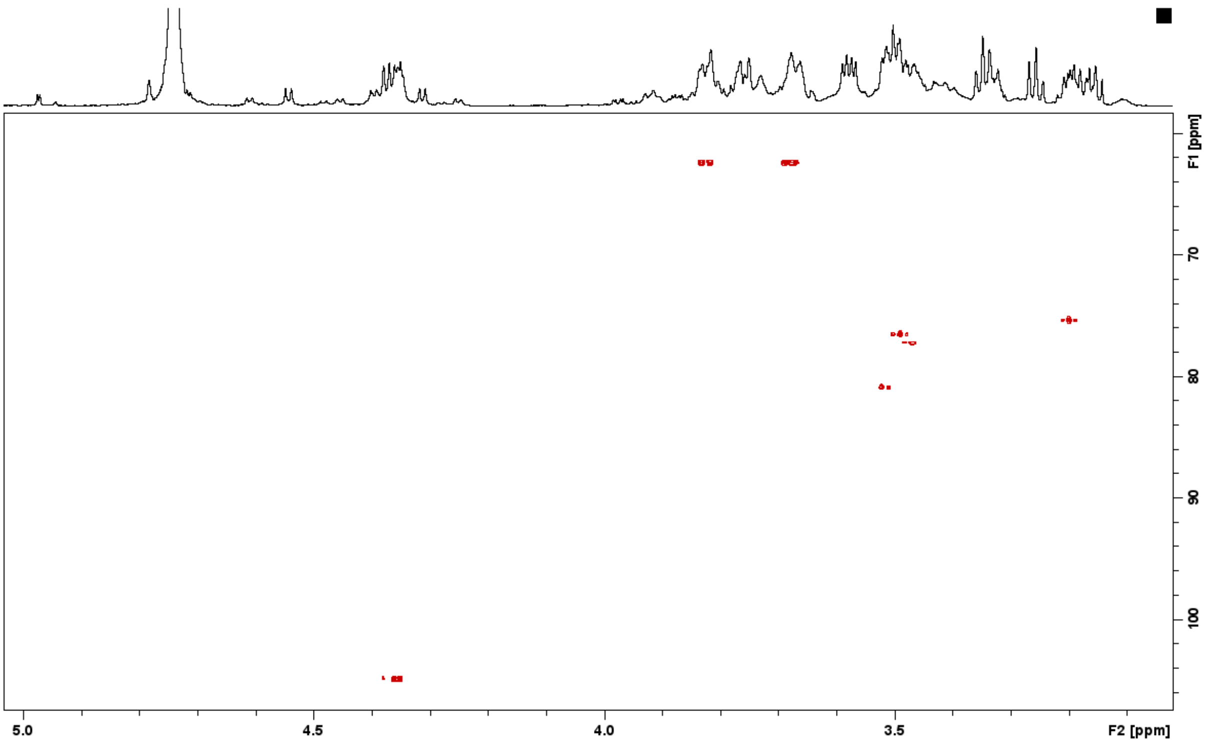

Figure 60 HSQC spectrum of **9mer-I-F** (800 MHz, D_2O, 298 K)**.** Only the cross peaks for the ^{13}C-labeled residue F are visible at this intensity level.

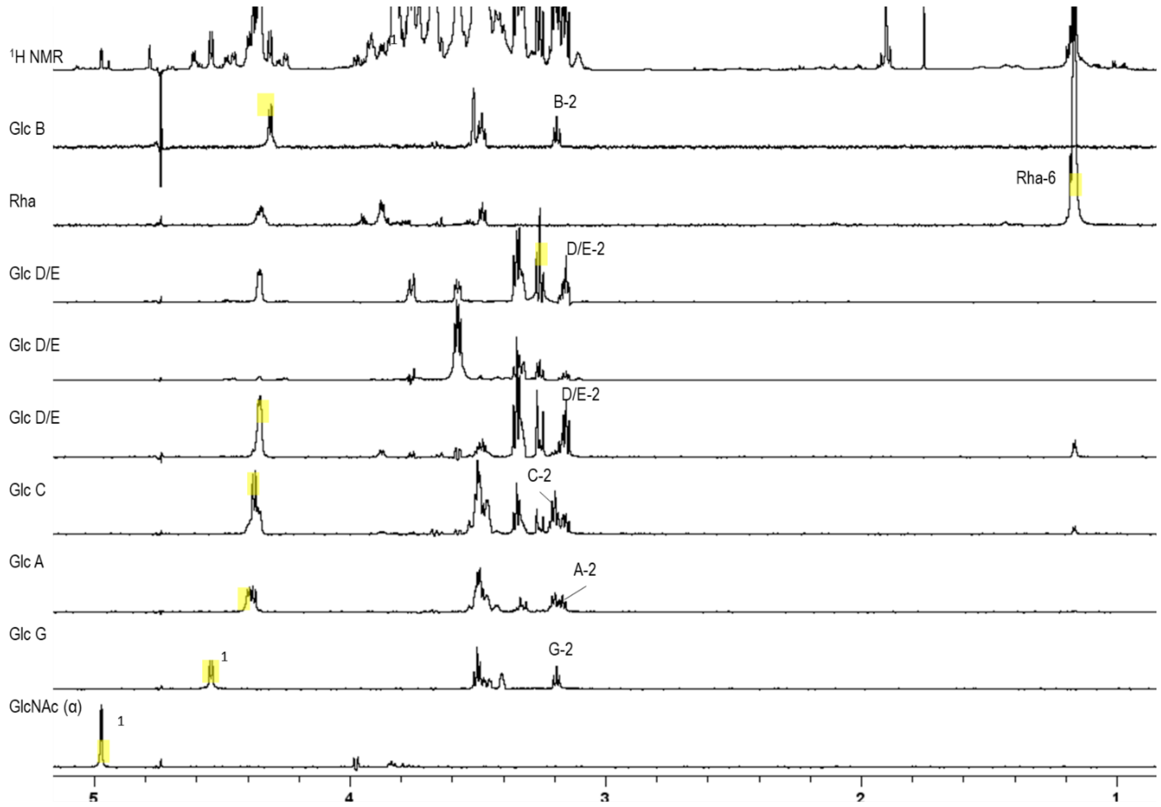

Figure 61 Selective 1D-TOCSY (800 MHz, mixing time 100 ms, D_2O, 288 K) of **9mer-I-F** with some of the key assignments. Those NMR signals chosen for selective excitation are highlighted in yellow.

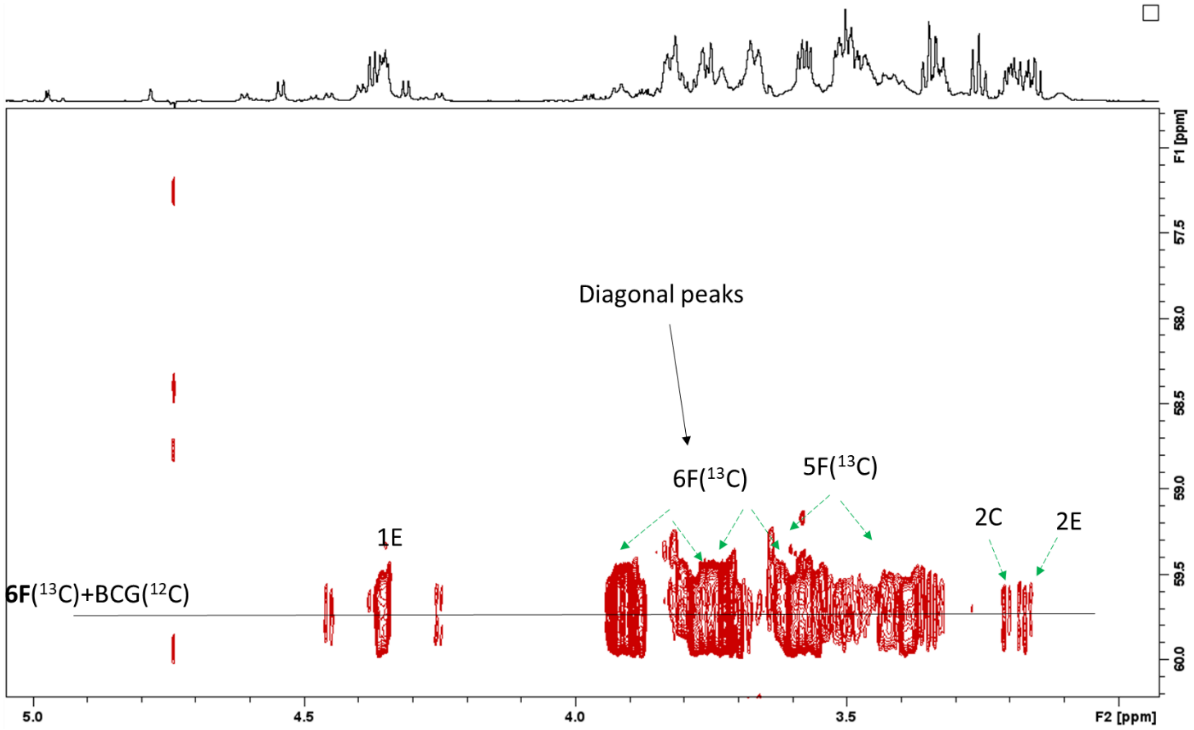

Figure 62 HSQC-NOESY CH2-6 region of **9mer-I-F**. The NOEs from the C-6 belonging to the ^{13}C-labelled F residue are shown. Besides the expected intra-residue NOEs and those to protons belonging to the vicinal E residue, the key F-6/C-2 NOE is also observed. Acquisition: TD (F2xF1)=6K x 384 points, SW(F2xF1)=12x60 ppm, 288 K, mixing time 300 ms, D_2O, 800 MHz. Processing: SI(F2xF1)=16K x 4K, QSIN (ssb(F2xF1)=2.8/3).

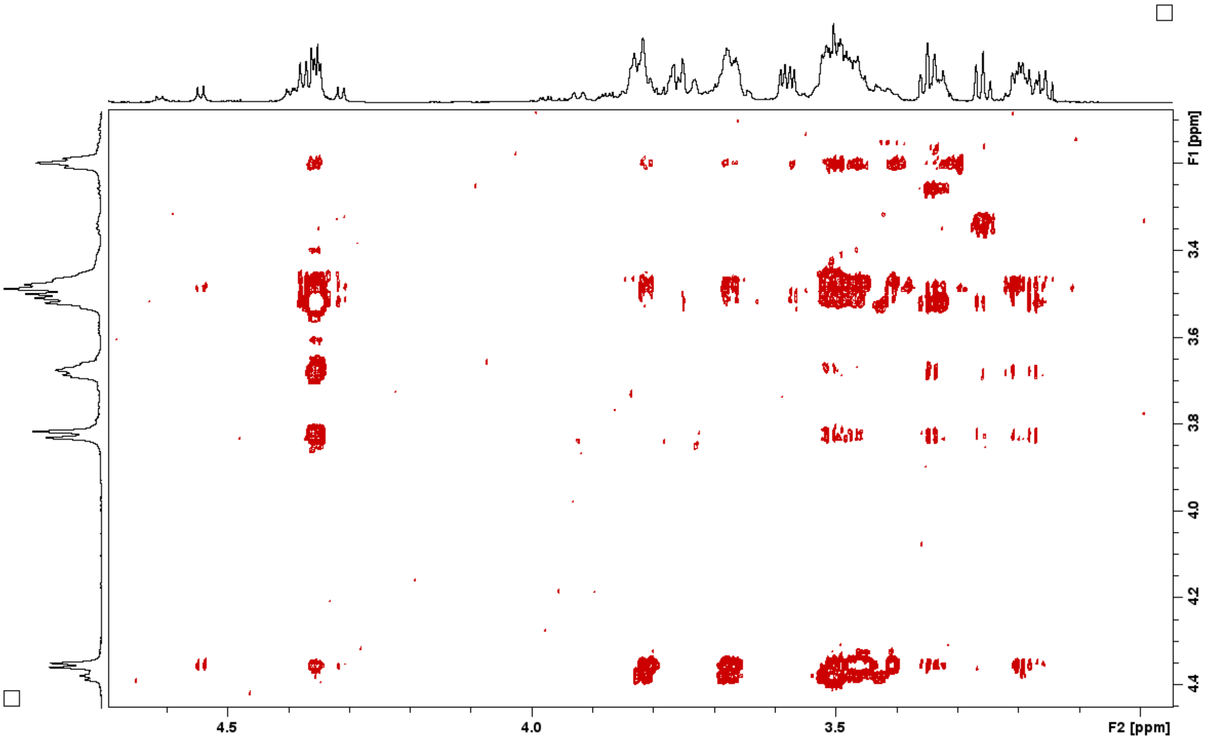

Figure 63 Half-filtered $^1H[^{13}C]$-$^1H[^{12}C]$ NOESY of **9mer-I-F**. Acquisition: TD (F2xF1)=4K x 384 points, SW(F2xF1)=8 x 4ppm, 288 K, mixing time 300 ms, D_2O, 800 MHz. Processing: SI(F2xF1)=4K x 2K, QSIN (ssb(F2xF1)=2/2).

5.10 Diffusion experiments (DOSY)

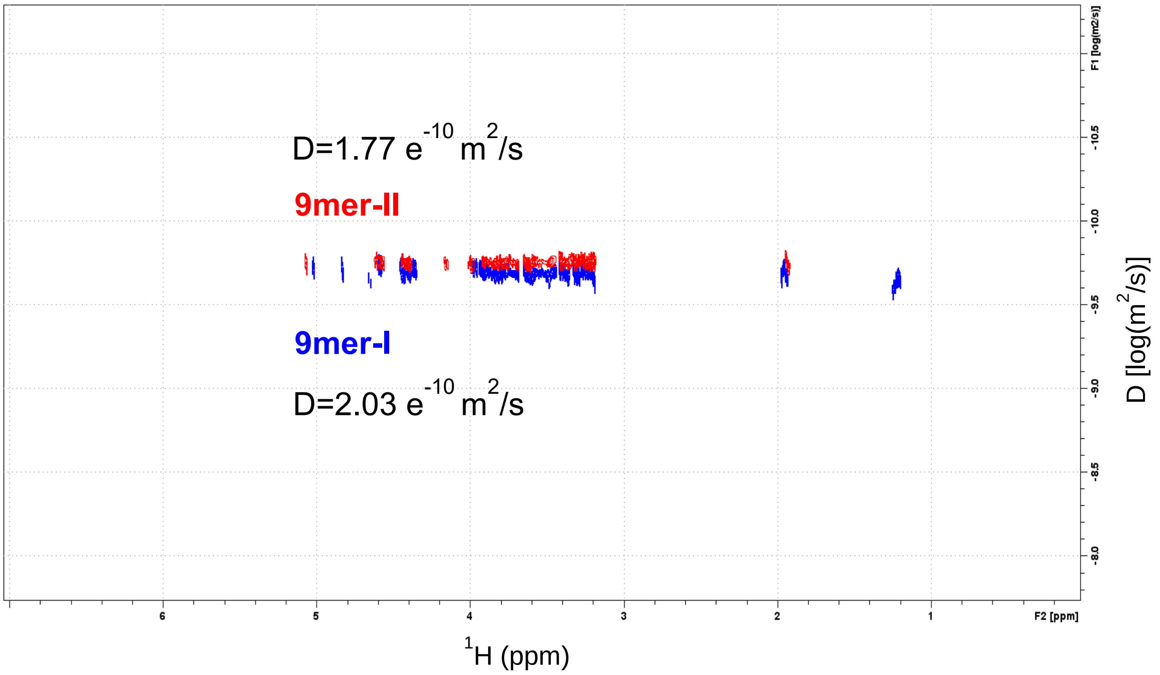

Figure 64 Superimposition of the DOSY spectra acquired for **9mer-I** (*blue*) and **9mer-II** (*red*), (600 MHz, D_2O, 295 K). The calculated diffusion coefficients for both compounds are indicated.

The diffusion coefficients of **9mer-I** and **9mer-II** were measured in 1.5 mM D_2O solutions, at 295 K, in a Bruker AV-III 600 MHz NMR spectrometer equipped with a BBO probe with Z gradients. The standard Bruker pulse sequence *ledbpgppr2s*, with longitudinal eddy current delay (5ms) and bipolar gradient pulses, was applied, using 150 ms of diffusion time Δ (d20) and 2.4 ms of diffusion gradient length δ (2 x p30). The experiments were acquired with 48 gradient increments with increasing gradient strength. The data were analysed with TOPSPIN 4.1.4 software to obtain both the 2D-DOSY spectra (see figure XX) and the diffusion coefficient values using the T1/T2 module included in the software. The final value indicated in the figure for each compound was calculated from the mean of the values obtained for different peaks along the NMR spectra.

Applying the Stokes-Einstein equation (1), it is possible correlate the diffusion coefficient with the hydrodynamic radius of both molecules, and thus with the hydrodynamic volume:

$$D = \frac{k_B \cdot T}{6\pi\eta R} \quad (1)$$

$$\frac{D_{9merI}}{D_{9merII}} = \frac{R_{9merII}}{R_{9merI}} = 1.15 \qquad \Rightarrow \qquad \frac{V_{9merII}}{V_{9merI}} = 1.52$$

As the molecular weights of both compounds are similar, the difference in diffusion coefficients is an indication of the different shapes that the two compounds adopt and of their different spatial volume.

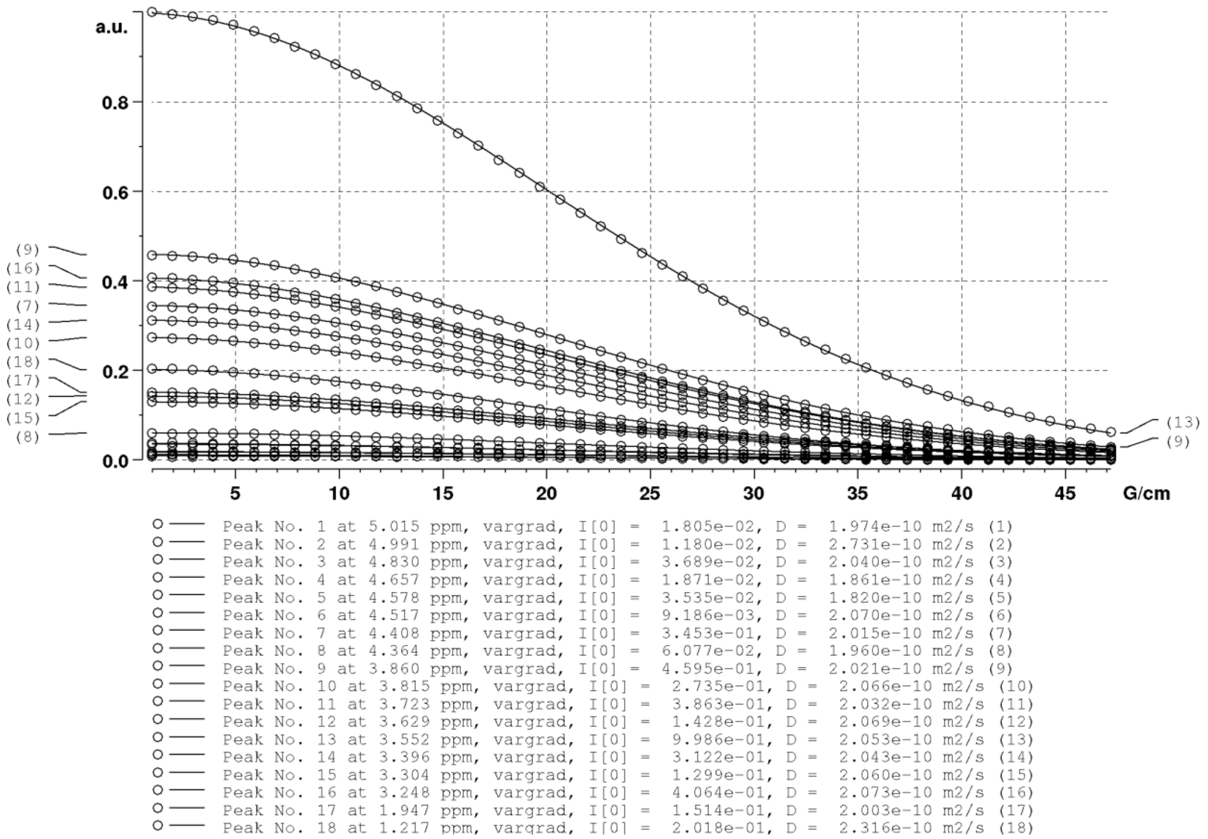

**Mean**	$2.03\ 10^{-10}$ m^2/s
**Standard deviation**	$1.04\ 10^{-11}$ m^2/s

Figure 65 Experimental diffusion data fitting for **9mer-I**. *Peak nº2 was omitted from the mean calculation.

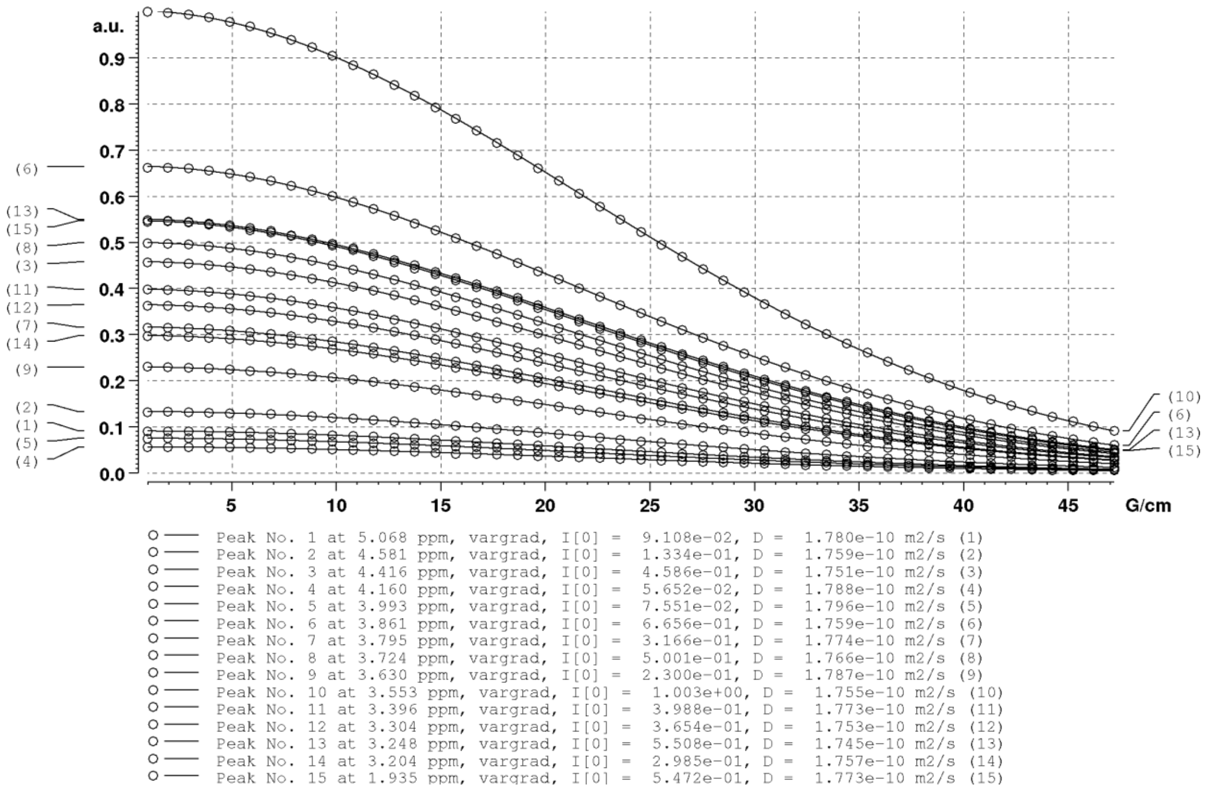

**Mean**	$1.77\ 10^{-10}\ m^2/s$
**Standard deviation**	$1.53\ 10^{-12}\ m^2/s$

Figure 66 Experimental diffusion data fitting for **9mer-II**.

5.11 Degradation of 9mer-I

9mer-I as well as the ^{13}C-labelled analogues (i.e., 9mer-I-B and 9mer-I-F) were stable in D_2O solution at room temperature for ca. one week, after which time degradation was noticed (Figure 67). RP HPLC and MALDI analysis of the degraded sample suggest that hydrolysis at the α-L-Rha-(1,3)-D-GlcNAc glycosidic linkage is occurring. We observed a peak at m/z 673, matching the hydrolyzed fragment (Figure 68) as well as variations in the ^1H NMR spectrum at the corresponding resonances (Figure 67).

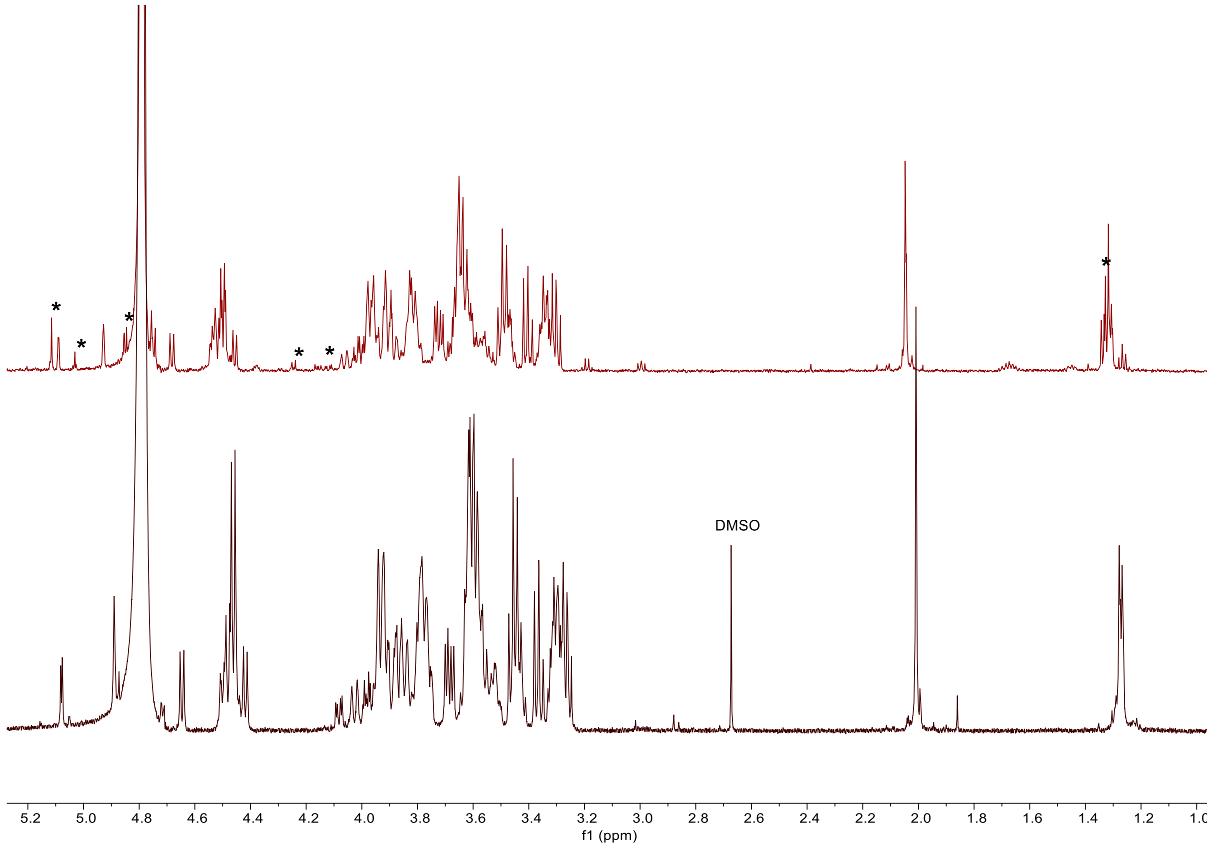

Figure 67 Comparison of ^1H NMR of **9mer-I** taken immediately after synthesis (*bottom*, 600 MHz, D_2O, 292 K) and after keeping the sample at room temperature for ca. 7 days in aqueous solution (*top*, 600 MHz, D_2O, 296 K). Additional peaks belonging to impurities are indicated with *.

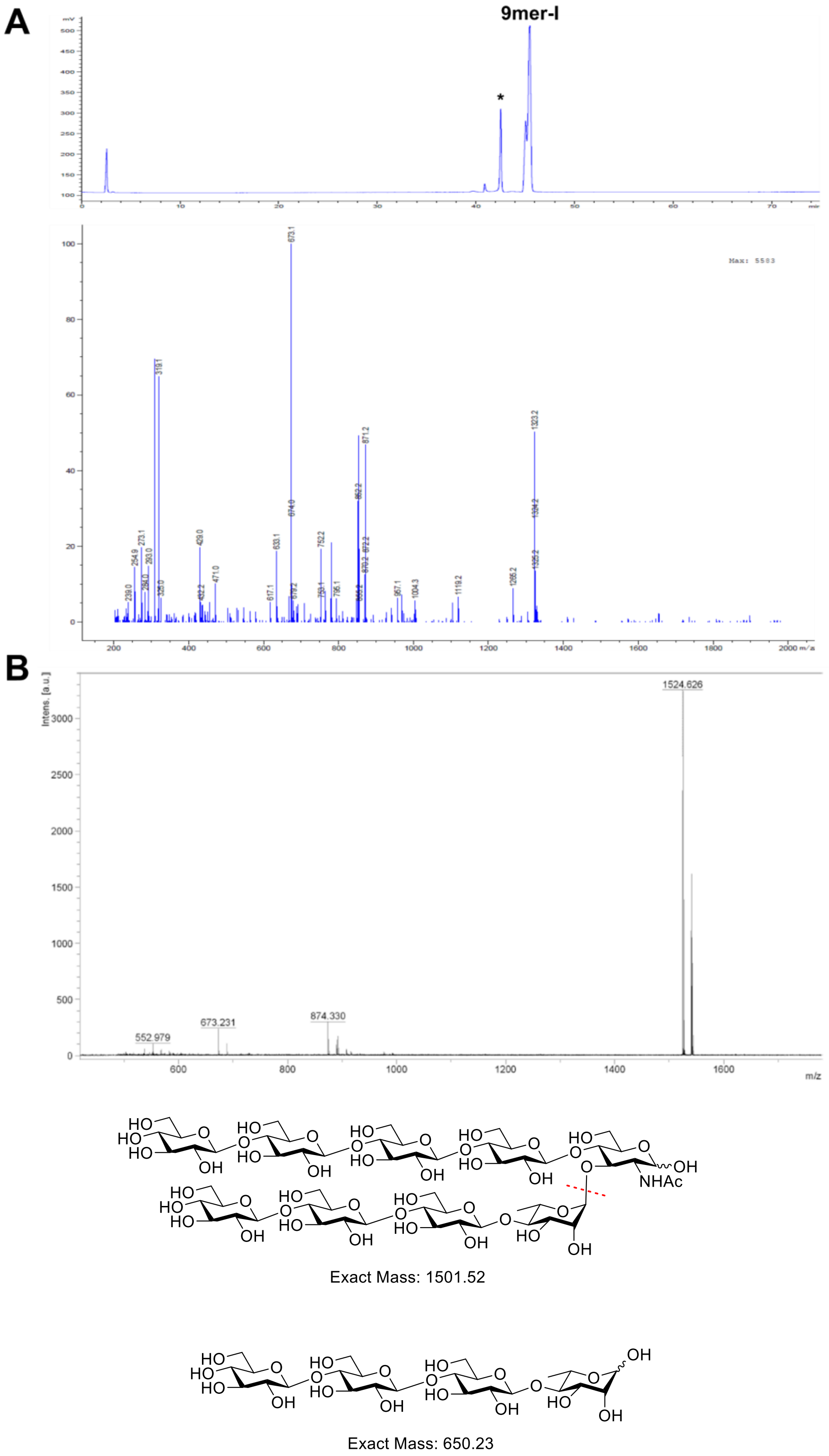

Figure 68 A) RP HPLC chromatogram of degraded sample of **9mer-I** (Hypercarb column, ThermoFisher scientific, 150 x 4.6 mm, 3 μm) flow rate of 0.7 mL/min with H_2O (0.1% formic acid) and ACN as eluents [isocratic (5 min), linear gradient to 50% ACN (60 min), linear gradient to 100% ACN (5 min), isocratic 100% ACN (5 min)] and ESI spectrum of the impurity peak (*). B) MALDI-TOF of the degraded sample of **9mer-I**. The signal at m/z 673 was assigned to $[M+Na]^+$ of the 4mer fragment.

6 References

(1) Dallabernardina, P.; Schuhmacher, F.; Seeberger, P. H.; Pfrengle, F. Mixed-Linkage Glucan Oligosaccharides Produced by Automated Glycan Assembly Serve as Tools To Determine the Substrate Specificity of Lichenase. *Chem. - A Eur. J* **2017**, *23* (13), 3191–3196. https://doi.org/10.1002/chem.201605479.

(2) Le Mai Hoang, K.; Pardo-Vargas, A.; Zhu, Y.; Yu, Y.; Loria, M.; Delbianco, M.; Seeberger, P. H. Traceless Photolabile Linker Expedites the Chemical Synthesis of Complex Oligosaccharides by Automated Glycan Assembly. *J. Am. Chem. Soc.* **2019**, *141* (22), 9079–9086. https://doi.org/10.1021/jacs.9b03769.

(3) Ning, S.; Zhang, L.; Ma, J.; Chen, L.; Zeng, G.; Yang, C.; Zhou, Y.; Guo, X.; Deng, X. Modular and Scalable Synthesis of Nematode Pheromone Ascarosides: Implications in Eliciting Plant Defense Response. *Org. Biomol. Chem.* **2020**, *18* (26), 4956–4961. https://doi.org/10.1039/d0ob00652a.

(4) Delbianco, M.; Kononov, A.; Poveda, A.; Yu, Y.; Diercks, T.; Jiménez-Barbero, J.; Seeberger, P. H. Well-Defined Oligo- and Polysaccharides as Ideal Probes for Structural Studies. *J. Am. Chem. Soc.* **2018**, *140* (16), 5421–5426. https://doi.org/10.1021/jacs.8b00254.

(5) Mo, K.-F.; Li, H.; Mague, J. T.; Ensley, H. E. Synthesis of the β-1,3-Glucan, Laminarahexaose: NMR and Conformational Studies. *Carbohydr. Res.* **2009**, *344* (4), 439–447. https://doi.org/10.1016/j.carres.2008.12.014.

(6) Gude, M.; Ryf, J.; White, P. D. An Accurate Method for the Quantitation of Fmoc-Derivatized Solid Phase Supports. *Lett. Pept. Sci.* **2002**, *9* (4), 203–206. https://doi.org/doi.org/10.1023/A:1024148619149.

(7) Hurevich, M.; Kandasamy, J.; Ponnappa, B. M.; Collot, M.; Kopetzki, D.; McQuade, D. T.; Seeberger, P. H. Continuous Photochemical Cleavage of Linkers for Solid-Phase Synthesis. *Org. Lett.* **2014**, *16* (6), 1794–1797. https://doi.org/10.1021/ol500530q.

(8) Ohara, K.; Lin, C.-C.; Yang, P.-J.; Hung, W.-T.; Yang, W.-B.; Cheng, T.-J. R.; Fang, J.-M.; Wong, C.-H. Synthesis and Bioactivity of β-(1→4)-Linked Oligomannoses and Partially Acetylated Derivatives. *J. Org. Chem.* **2013**, *78* (13), 6390–6411. https://doi.org/10.1021/jo4005266.

(9) Van Der Spoel, D.; Lindahl, E.; Hess, B.; Groenhof, G.; Mark, A. E.; Berendsen, H. J. C. GROMACS: Fast, Flexible, and Free. *J. Comput. Chem.* **2005**, *26* (16), 1701–1718. https://doi.org/10.1002/jcc.20291.

(10) Kirschner, K. N.; Yongye, A. B.; Tschampel, S. M.; González-Outeiriño, J.; Daniels, C. R.; Foley, B. L.; Woods, R. J. GLYCAM06: A Generalizable Biomolecular Force Field. Carbohydrates. *J. Comput. Chem.* **2008**, *29* (4), 622–655. https://doi.org/10.1002/jcc.20820.

(11) Sauter, J.; Grafmüller, A. Predicting the Chemical Potential and Osmotic Pressure of Polysaccharide Solutions by Molecular Simulations. *J. Chem. Theory Comput.* **2016**, *12* (9), 4375–4384. https://doi.org/10.1021/acs.jctc.6b00295.

(12) Mahoney, M. W.; Jorgensen, W. L. A Five-Site Model for Liquid Water and the Reproduction of the Density Anomaly by Rigid, Nonpolarizable Potential Functions. *J. Chem. Phys.* **2000**, *112* (20), 8910–8922. https://doi.org/10.1063/1.481505.

(13) Hoover, W. G. Canonical Dynamics: Equilibrium Phase-Space Distributions. *Phys. Rev. A* **1985**, *31* (3), 1695–1697. https://doi.org/10.1103/PhysRevA.31.1695.

(14) Nosé, S. A Unified Formulation of the Constant Temperature Molecular Dynamics Methods. *J. Chem. Phys.* **1984**, *81* (1), 511–519. https://doi.org/10.1063/1.447334.

(15) Parrinello, M.; Rahman, A. Crystal Structure and Pair Potentials: A Molecular-Dynamics Study.

Phys. Rev. Lett. **1980**, *45* (14), 1196–1199. https://doi.org/10.1103/PhysRevLett.45.1196.

(16) Parrinello, M.; Rahman, A. Polymorphic Transitions in Single Crystals: A New Molecular Dynamics Method. *J. Appl. Phys.* **1981**, *52* (12), 7182–7190. https://doi.org/10.1063/1.328693.

(17) Darden, T.; York, D.; Pedersen, L. Particle Mesh Ewald: An N ·log(N) Method for Ewald Sums in Large Systems. *J. Chem. Phys.* **1993**, *98* (12), 10089–10092. https://doi.org/10.1063/1.464397.

(18) Hess, B.; Bekker, H.; Berendsen, H. J. C.; Fraaije, J. G. E. M. LINCS: A Linear Constraint Solver for Molecular Simulations. *J. Comput. Chem.* **1997**, *18* (12), 1463–1472. https://doi.org/10.1002/(SICI)1096-987X(199709)18:12<1463::AID-JCC4>3.0.CO;2-H.

(19) Miyamoto, S.; Kollman, P. A. Settle: An Analytical Version of the SHAKE and RATTLE Algorithm for Rigid Water Models. *J. Comput. Chem.* **1992**, *13* (8), 952–962. https://doi.org/10.1002/jcc.540130805.

(20) Battistel, M. D.; Azurmendi, H. F.; Frank, M.; Freedberg, D. I. Uncovering Nonconventional and Conventional Hydrogen Bonds in Oligosaccharides through NMR Experiments and Molecular Modeling: Application to Sialyl Lewis-X. *J. Am. Chem. Soc.* **2015**, *137* (42), 13444–13447. https://doi.org/10.1021/jacs.5b03824.

(21) Zierke, M.; Smieško, M.; Rabbani, S.; Aeschbacher, T.; Cutting, B.; Allain, F. H.-T.; Schubert, M.; Ernst, B. Stabilization of Branched Oligosaccharides: Lewis x Benefits from a Nonconventional C–H···O Hydrogen Bond. *J. Am. Chem. Soc.* **2013**, *135* (36), 13464–13472. https://doi.org/10.1021/ja4054702.

(22) Aeschbacher, T.; Zierke, M.; Smieško, M.; Collot, M.; Mallet, J.; Ernst, B.; Allain, F. H. -T.; Schubert, M. A Secondary Structural Element in a Wide Range of Fucosylated Glycoepitopes. *Chem. - A Eur. J* **2017**, *23* (48), 11598–11610. https://doi.org/10.1002/chem.201701866.

(23) Zhang, Y.; Gómez-Redondo, M.; Jiménez-Osés, G.; Arda, A.; Overkleeft, H. S.; Marel, G. A.; Jiménez-Barbero, J.; Codée, J. D. C. Synthesis and Structural Analysis of Aspergillus Fumigatus Galactosaminogalactans Featuring A-Galactose, A-Galactosamine and A- N -Acetyl Galactosamine Linkages. *Angew. Chem., Int. Ed.* **2020**, *59* (31), 12746–12750. https://doi.org/10.1002/anie.202003951.
